# Supplementary material for: Copper-Free Halodediazoniation of Arenediazonium Tetrafluoroborates in Deep Eutectic Solvents-like Mixtures
Source: Molecules. 2022 Mar 15;27(6):1909. doi: 10.3390/molecules27061909 (PMC8950527; doi:10.3390/molecules27061909)

Supplementary Material

Copper-free halodediazoniation of Arenediazonium Tetrafluoroborates in Deep Eutectic solvents-like mixtures.

Giovanni Ghigo, Matteo Bonomo, Achille Antenucci, Chiara Reviglio, and Stefano Dughera

INDEX

Computational method.	2
**Table S1.** Calculated absolute and relative energies for the solvation of **1a** in glycerol and for the N_2-scrambling in **1a'**.	3
**Table S2.** Calculated absolute and relative energies for the solvation of **1f** in glycerol.	3
**Table S3.** Calculated absolute and relative energies for the reactions of **1a'** in glycerol.	4
**Table S4.** Calculated absolute and relative energies for the reactions of **1f'** in glycerol.	8
**Table S5.** Calculated absolute and relative energies for the intramolecular fluorination of **1a**.	10
Pictures and Cartesian coordinates for the solvation of **1a** in glycerol and N_2-scrambling.	11
Pictures and Cartesian coordinates for the solvation of **1f** in glycerol.	13
Pictures and Cartesian coordinates for the reactions of **1a'**.	15
Pictures and Cartesian coordinates for the reactions of **1f'**.	68
Pictures and Cartesian coordinates of the structures for the intramolecular fluorination of **1a**.	92
Physical and NMR data of aryl halides **2**, **4**, and **5**.	94
References.	96
NMR spectra of aryl halides **2**, **4**, and **5**.	97
NMR spectra of glycerol/KBr (6:1) solvent system.	120
NMR spectra of recovered glycerol/KBr (6:1) solvent system after the fifth run.	121
DSC of Gly and Gly/KBr 6:1 solvent systems.	122

Computational method.

The structures of the reactants, intermediates and transition states have been optimized by using the density functional method (DFT)[1] with the functional M06[2,3] and the basis sets 6–311+G(d,p). Then, the electronic energy values where refined by single-point calculations with the basis set 6–311++G (3df,2pd).[4–6] The nature of the critical points was characterized by using vibrational analysis[7] which also furnished the Zero Point Energies (ZPE) and entropies for the calculations of the Free Energies. These have been converted from the gas phase to the 1 M standard state at 1 atm and 298.15 K[8] and used to calculate the rate constant with the Eyring equation.[9] The solvent effects (glycerol, because at the present a method to mimic DES is not available) were introduced in all calculations using the Polarized Continuum Method (PCM).[10] The following parameter were used: $\varepsilon = 42.5$, $\varepsilon_{inf} = n^2 = (1.4746)^2 = 2.174$. The calculations were performed by the quantum package Gaussian 16-A.03[11] The figures were obtained using the graphical program Molden.[12]

References:

1. R.G. Parr, Density Functional Theory of Atoms and Molecules, in: Horizons Quantum Chem., Springer Netherlands, 1980: pp. 5–15. doi:10.1007/978-94-009-9027-2_2.

2. Y. Zhao, D.G. Truhlar, *Theor. Chem. Acc.*, **2008**, *120*, 215–24.

3. Y. Zhao, D.G. Truhlar, *Acc. Chem. Res.*, **2008**, *41*,157–167

4. A.D. McLean, G.S. Chandler, *J. Chem. Phys.,* **1980***, 72*, 5639–5648,

5. T. Clark, J. Chandrasekhar, G.W. Spitznagel, P.V.R. Schleyer, *J. Comput. Chem.,* **1983**, 4 294–301

6. M.J. Frisch, J.A. Pople, J.S. Binkley, *J. Chem. Phys.,* **1984***,* 80, 3265–3269,.

7. J. Foresman, A. Frisch, Exploring chemistry with electronic structure methods, 1996, Gaussian Inc, Pittsburgh, PA, 1996, http://gaussian.com/expchem3/(accessed June 4, 2021).

8. R.F. Ribeiro, A.V. Marenich, C.J. Cramer, D.G. Truhlar, *J. Phys. Chem. B.*, **2011**, *115*, 14556–14562.

9. K.J. Laidler, M.C. King, *J. Phys. Chem.*, **1983**, *87*, 2657–2664,

10. J. Tomasi, B. Mennucci, R. Cammi, *Chem. Rev.*, **2005**, *105*, 2999–3093.

11. D.J. Frisch, M. J.; Trucks, G. W.; Schlegel, H. B.; Scuseria, G. E.; Robb, M. A.; Cheeseman, J. R.; Scalmani, G.; Barone, V.; Petersson, G. A.; Nakatsuji, H.; Li, X.; Caricato, M.; Marenich, A. V.; Bloino, J.; Janesko, B. G.; Gomperts, R.; Mennucci, B.; Hratch, Gaussian 16, Revision A.03, (2016).

12. G. Schaftenaar, J.H. Noordik, *J. Comput. Aided. Mol. Des.*, **2000**, *14*, 123–134.

Table S1. Calculated absolute and relative (in kcal mol^{-1}) energies for the solvation of 4-nitrobenzendiazonium tetrafluoborate **1a** in glycerol and for the N_2-scrambling in 4-nitrobenzendiazonium **1a'** .

DiazoCation TFB Solvation		E /au[a]	ΔE	E^{0K} /au	ΔE^{0K}	G^{298K} /au	Δn	ΔG^{298K}	E(SP) /au[b]	G(SP) /au[c]	ΔG^{298K}
$4\text{-}O_2N\text{-}C_6H_4\text{-}NN^+\ BF_4^-$ (**ArN_2BF_4**)	**1a**	-969.84450	**0.00**	-969.72895	**0.00**	-969.77395	**0**	**0.00**	-969.91608	-969.84553	**0.00**
$4\text{-}O_2N\text{-}C_6H_4\text{-}NN^+$ (**ArN_2^+**)	**1a'**	-545.20953		-545.10899		-545.14441			-545.25439	-545.18928	
BF_4^-		-424.62153		-424.60780		-424.63306			-424.64862	-424.66014	
**ArN_2^+ + BF_4^-**		-969.83106	**8.44**	-969.71679	**7.63**	-969.77747	**1**	**-0.31**	-969.90301	-969.84942	**-0.54**
**DiazoCation N2-Scrambling**		E /au[a]	ΔE	E^{0K} /au	ΔE^{0K}	G^{298K} /au	Δn	ΔG^{298K}	E(SP) /au[b]	G(SP) /au[c]	ΔG^{298K}
$4\text{-}O_2N\text{-}C_6H_4\text{-}NN^+$ (**ArN_2^+**)	**1a'**	-545.20953	**0.00**	-545.10899	**0.00**	-545.14441		**0.00**	-545.25439	-545.18928	**0.00**
$TS_{N2\text{-}Scramble}$		-545.15124	**36.58**	-545.05804	**31.98**	-545.09564		**30.61**	-545.19604	-545.14044	**30.64**

[a] M06/6-311+G(d,p); [b] SP M06/6-311++G(3df,2pd); [c] SP enegies combined with thermal corrections calculated with 6-311+G(d,p).

Table S2. Calculated absolute and relative (in kcal mol^{-1}) energies for the solvation of 4-methoxybenzendiazonium tetrafluoborate **1f** in glycerol.

DiazoCation TFB Solvation		E /au[a]	ΔE	E^{0K} /au	ΔE^{0K}	G^{298K} /au	Δn	ΔG^{298K}	E(SP) /au[b]	G(SP) /au[c]	ΔG^{298K}
$4\text{-}CH_3O\text{-}C_6H_4\text{-}NN^+\ BF_4^-$ (**ArN_2BF_4**)	**1f**	-879.88724	**0.00**	-879.74145	**0.00**	-879.78486	**0**	**0.00**	-879.95307	-879.85069	**0.00**
$4\text{-}CH_3O\text{-}C_6H_4\text{-}NN^+$ (**ArN_2^+**)	**1f'**	-455.25492		-455.12412		-455.15778			-455.29414	-455.19700	
BF_4^-		-424.62153		-424.60780		-424.63306			-424.64862	-424.66014	
**ArN_2^+ + BF_4^-**		-879.87645	**6.77**	-879.73191	**5.98**	-879.79083	**1**	**-1.85**	-879.94276	-879.85714	**-2.15**

[a] M06/6-311+G(d,p); [b] SP M06/6-311++G(3df,2pd); [c] SP enegies combined with thermal corrections calculated with 6-311+G(d,p).

Table S3. Calculated absolute and relative (in kcal mol^{-1}) energies for the reactions of solvated 4-nitrobenzendiazonium **1a'** in glycerol.

**S_N to Fluoride - GL-solvated**		E /au[a]	ΔE	E^{0K} /au	$ΔE^{0K}$	G^{298K} /au	Δn	$ΔG^{298K}$	E(SP) /au[b]	G(SP) /au[c]	$ΔG^{298K}$
Cpl [$ArN_2^+(GL)_3$]	**03**	-1579.38516		-1578.92466		-1578.99645			-1579.49787	-1579.10916	
Cpl [F^- $(GL)_2$]	**04**	-789.45775		-789.21727		-789.26535			-789.50581	-789.31341	
**[$ArN_2^+(GL)_3$] + [$F^-(GL)_2$]**		-2368.84291	**0.00**	-2368.14193	**0.00**	-2368.26180	**0**	**0.00**	-2369.00369	-2368.42257	**0.00**
Cpl [$ArN_2^+(GL)_2$ * $F^-(GL)_2$]	**05**	-2024.14607		-2023.56151		-2023.64491			-2024.28093	-2023.77978	
GL	**01**	-344.70993		-344.59196		-344.62307			-344.73430	-344.64744	
**Cpl [$ArN_2^+(GL)_2$ * $F^-(GL)_2$] + GL**		-2368.85599	**-8.21**	-2368.15348	**-7.24**	-2368.26799	**0**	**-3.88**	-2369.01522	-2368.42721	**-2.91**
$TS_{SN2'}$ 4-O_2N-C_6H_4-NN^+ F^- $(GL)_4$	**06**	-2024.10221		-2023.52449		-2023.61257			-2024.23689	-2023.74724	
**$TS_{SN2'}$ + GL**		-2368.81214	**19.31**	-2368.11645	**15.99**	-2368.23565	**0**	**16.41**	-2368.97118	-2368.39468	**17.50**
Cpl [Ar-F * N_2 * $(GL)_4$]	**07**	-2024.20539		-2023.62520		-2023.71214			-2024.34369	-2023.85044	
**Cpl [Ar-F * N_2 * $(GL)_4$] + GL**		-2368.91532	**-45.43**	-2368.21717	**-47.21**	-2368.33521	**0**	**-46.07**	-2369.07799	-2368.49788	**-47.26**
Ar-F	**08**	-535.83110		-535.73704		-535.76976			-535.87081	-535.80948	
Cpl $(GL)_2$	**02**	-689.43303		-689.19471		-689.23917			-689.48016	-689.28629	
**Ar-F + N_2 + 2$(GL)_2$ + GL**		-2368.89809	**-34.63**	-2368.20383	**-38.84**	-2368.37500	**3**	**-65.35**	-2369.06434	-2368.54125	**-68.79**
**S_{RN} to Fluoride - GL-solvated**		E /au[a]	ΔE	E^{0K} /au	$ΔE^{0K}$	G^{298K} /au	Δn	$ΔG^{298K}$	E(SP) /au[b]	G(SP) /au[c]	$ΔG^{298K}$
^{2}4-O_2N-C_6H_4* (**Ar***)	**r00**	-435.903784		-435.814576		-435.846742			-435.936871	-435.879829	
**Cpl ^{2}[Ar * $F^-(GL)_2$]**	**r01**	-1225.373093	**0.00**	-1225.042504	**0.00**	-1225.104955	**0**	**0.00**	-1225.453287	-1225.185149	**0.00**
**TS_{CX} to Cpl ^{2}[Ar-F * $(GL)_2$]*$^-$**	**r02**	-1225.353878	**12.06**	-1225.024289	**11.43**	-1225.083646	**0**	**13.37**	-1225.433739	-1225.163507	**13.58**
**Cpl ^{2}[Ar-F * $(GL)_2$]*$^-$**	**r03**	-1225.403106	**-18.83**	-1225.071538	**-18.22**	-1225.130481	**0**	**-16.02**	-1225.485645	-1225.213020	**-17.49**
**S_N to Chloride - GL-solvated**		E /au[a]	ΔE	E^{0K} /au	$ΔE^{0K}$	G^{298K} /au	Δn	$ΔG^{298K}$	E(SP) /au[b]	G(SP) /au[c]	$ΔG^{298K}$
Cpl [Cl^- $(GL)_2$]	**09**	-1149.81599		-1149.57797		-1149.63088			-1149.86587	-1149.68076	

**[ArN$_2^+$(GL)$_3$] + [Cl$^-$(GL)$_2$]**		-2729.20115	**0.00**	-2728.50263	**0.00**	-2728.62732	**0**	**0.00**	-2729.36374	-2728.78992	**0.00**
Cpl [ArN$_2^+$(GL)$_2$ * Cl$^-$(GL)$_2$]	**10**	-2384.50732		-2383.92665		-2384.01152			-2384.64375	-2384.14796	
**Cpl [ArN$_2^+$(GL)$_2$ * Cl$^-$(GL)$_2$] + GL**		-2729.21725	**-10.10**	-2728.51862	**-10.03**	-2728.63460	**0**	**-4.56**	-2729.37805	-2728.79540	**-3.44**
TS$_{SN2'}$ 4-O$_2$N-C$_6$H$_4$-NN$^+$ Cl$^-$ (GL)$_4$	**11**	-2384.45927		-2383.88426		-2383.97392			-2384.59501	-2384.10967	
**TS$_{SN2'}$ + GL**		-2729.16920	**20.05**	-2728.47623	**16.56**	-2728.59699	**0**	**19.03**	-2729.32931	-2728.75710	**20.59**
Cpl [Ar-Cl * N$_2$ * (GL)$_4$]	**12**	-2384.56719		-2383.98638		-2384.07193			-2384.70503	-2384.20977	
**Cpl [Ar-Cl * N$_2$ * (GL)$_4$] + GL**		-2729.27712	**-47.67**	-2728.57834	**-47.51**	-2728.69500	**0**	**-42.47**	-2729.43933	-2728.85721	**-42.23**
Ar-Cl	**13**	-896.18290		-896.09026		-896.12467			-896.22188	-896.16365	
**Ar-Cl + N$_2$ + 2(GL)$_2$ + GL**		-2729.24990	**-30.59**	-2728.55706	**-34.16**	-2728.72991	**3**	**-58.69**	-2729.41541	-2728.89542	**-60.52**

**S$_{RN}$ to Choride - GL-solvated**		E /au[a]	ΔE	E^{0K} /au	ΔE^{0K}	G^{298K} /au	Δn	ΔG^{298K}	E(SP) /au[b]	G(SP) /au[c]	ΔG^{298K}
**Cpl ^{2}[Ar * Cl$^-$(GL)$_2$]**	**r04**	-1585.736252	**0.00**	-1585.407328	**0.00**	-1585.470276	**0**	**0.00**	-1585.816819	-1585.550843	**0.00**
TS$_{CX}$ to **Cpl ^{2}[Ar-Cl * (GL)$_2$]$^{*-}$**	**r05**	-1585.720558	**9.85**	-1585.393238	**8.84**	-1585.455526	**0**	**9.26**	-1585.801746	-1585.536714	**8.87**
**Cpl ^{2}[Ar-Cl * (GL)$_2$]$^{*-}$**	**r06**	-1585.757227	**-13.16**	-1585.427052	**-12.38**	-1585.487314	**0**	**-10.69**	-1585.839708	-1585.569796	**-11.89**

**S$_N$ to Bromide - GL-solvated**		E /au[a]	ΔE	E^{0K} /au	ΔE^{0K}	G^{298K} /au	Δn	ΔG^{298K}	E(SP) /au[b]	G(SP) /au[c]	ΔG^{298K}
Cpl [Br$^-$ (GL)$_2$]	**14**	-3263.63865		-3263.40138		-3263.45558			-3263.68856	-3263.50549	
**[ArN$_2^+$(GL)$_3$] + [Br$^-$(GL)$_2$]**		-4843.02381	**0.00**	-4842.32604	**0.00**	-4842.45202	**0**	**0.00**	-4843.18644	-4842.61465	**0.00**
Cpl [ArN$_2^+$(GL)$_2$ * Br$^-$(GL)$_2$]	**15**	-4498.32923		-4497.74880		-4497.83600			-4498.46590	-4497.97267	
**Cpl [ArN$_2^+$(GL)$_2$ * Br$^-$(GL)$_2$] + GL**		-4843.03916	**-9.63**	-4842.34076	**-9.24**	-4842.45908	**0**	**-4.43**	-4843.20020	-4842.62011	**-3.43**
TS$_{SN2'}$ 4-O$_2$N-C$_6$H$_4$-NN$^+$ Br$^-$ (GL)$_4$	**16**	-4498.28147		-4497.70686		-4497.79703			-4498.41768	-4497.93324	
**TS$_{SN2'}$ + GL**		-4842.99140	**20.34**	-4842.29882	**17.08**	-4842.42010	**0**	**20.03**	-4843.15197	-4842.58068	**21.32**
Cpl [Ar-Br * N$_2$ * (GL)$_4$]	**17**	-4498.39144		-4497.81170		-4497.89954			-4498.52739	-4498.03549	
**Cpl [Ar-Br * N$_2$ * (GL)$_4$] + GL**		-4843.10137	**-48.67**	-4842.40366	**-48.71**	-4842.52261	**0**	**-44.29**	-4843.26169	-4842.68293	**-42.84**

Ar-Br	18	-3010.00780		-3009.91576		-3009.95053			-3010.04459	-3009.98732	
**Ar-Br + N_2 + $2(GL)_2$ + GL**		-4843.07479	**-31.99**	-4842.38256	**-35.47**	-4842.55577	**3**	**-59.42**	-4843.23812	-4842.71910	**-59.86**

**S_{RN} to Bromide - GL-solvated**		E /au[a]	ΔE	E^{0K} /au	$ΔE^{0K}$	G^{298K} /au	Δn	$ΔG^{298K}$	E(SP) /au[b]	G(SP) /au[c]	$ΔG^{298K}$
**Cpl ^{2}[Ar * $Br^-$$(GL)_2$]**	**r07**	-3699.560567	**0.00**	-3699.232005	**0.00**	-3699.296747	**0**	**0.00**	-3699.641113	-3699.377292	**0.00**
TS_{CX} to **Cpl ^{2}[Ar-Br * $(GL)_2$]$^{*-}$**	**r08**	-3699.548364	**7.66**	-3699.221592	**6.53**	-3699.284155	**0**	**7.90**	-3699.628806	-3699.364597	**7.97**
**Cpl ^{2}[Ar-Br * $(GL)_2$]$^{*-}$**	**r09**	-3699.582395	**-13.70**	-3699.253030	**-13.19**	-3699.313671	**0**	**-10.62**	-3699.662525	-3699.393801	**-10.36**

**S_N to Iodide - GL-solvated**		E /au[a]	ΔE	E^{0K} /au	$ΔE^{0K}$	G^{298K} /au	Δn	$ΔG^{298K}$	E(SP) /au[b]	G(SP) /au[c]	$ΔG^{298K}$
Cpl [I^- $(GL)_2$]	19	-7608.88671		-7608.65016		-7608.70395			-7608.93722	-7608.75447	
**[$ArN_2^+$$(GL)_3$] + [$I^-$$(GL)_2$]**		-9188.27187	**0.00**	-9187.57482	**0.00**	-9187.70040	**0**	**0.00**	-9188.43510	-9187.86363	**0.00**
Cpl [$ArN_2^+$$(GL)_2$ * $I^-$$(GL)_2$]	20	-8843.58033		-8843.00070		-8843.08886			-8843.71746	-8843.22599	
**Cpl [$ArN_2^+$$(GL)_2$ * $I^-$$(GL)_2$] + GL**		-9188.29026	**-11.54**	-9187.59266	**-11.20**	-9187.71193	**3**	**-1.55**	-9188.45175	-9187.87343	**-0.46**
$TS_{SN2'}$ 4-O_2N-C_6H_4-NN^+ I^- $(GL)_4$	21	-8843.53053		-8842.95620		-8843.04772			-8843.66743	-8843.18462	
$TS_{SN2'}$ **+ GL**		-9188.24046	**19.71**	-9187.54816	**16.73**	-9187.67080	**0**	**18.57**	-9188.40172	-9187.83206	**19.81**
Cpl [Ar-I * N_2 * $(GL)_4$]	22	-8843.64226		-8843.06314		-8843.15182			-8843.78018	-8843.28973	
**Cpl [Ar-I * N_2 * $(GL)_4$] + GL**		-9188.35219	**-50.40**	-9187.65511	**-50.38**	-9187.77489	**3**	**-41.06**	-9188.51447	-9187.93717	**-40.47**
Ar-I	23	-7355.25798		-7355.16638		-7355.20270			-7355.29675	-7355.24147	
**Ar-I + N_2 + $2(GL)_2$ + GL**		-9188.32498	**-33.33**	-9187.63318	**-36.62**	-9187.80794	**3**	**-61.80**	-9188.49028	-9187.97324	**-63.10**

**S_{RN} to Iodide - GL-solvated**		E /au[a]	ΔE	E^{0K} /au	$ΔE^{0K}$	G^{298K} /au	Δn	$ΔG^{298K}$	E(SP) /au[b]	G(SP) /au[c]	$ΔG^{298K}$
**Cpl ^{2}[Ar * $I^-$$(GL)_2$]**	**r10**	-8044.810728	**0.00**	-8044.482783	**0.00**	-8044.547981	**0**	**0.00**	-8044.891853	-8044.629106	**0.00**
TS_{CX} to **Cpl ^{2}[Ar-I * $(GL)_2$]$^{*-}$**	**r11**	-8044.804431	**3.95**	-8044.477387	**3.39**	-8044.541102	**0**	**4.32**	-8044.885433	-8044.622104	**4.39**
**Cpl ^{2}[Ar-I * $(GL)_2$]$^{*-}$**	**r12**	-8044.833271	**-14.15**	-8044.504746	**-13.78**	-8044.566633	**0**	**-11.70**	-8044.915640	-8044.649002	**-12.48**

**Solvolysis**		E /au[a]	**ΔE**	E^{0K} /au	**ΔE^{0K}**	G^{298K} /au	**Δn**	**ΔG^{298K}**	E(SP) /au[b]	G(SP) /au[c]	**ΔG^{298K}**
**Cpl [$ArN_2^+(GL)_3$]**	**03**	-1579.38516	**0.00**	-1578.92466	**0.00**	-1578.99645	**0**	**0.00**	-1579.49787	-1579.10916	**0.00**
**TS_{Solv} [ArN_2^+ * 3 GL]**	**24**	-1579.34095	**27.74**	-1578.88588	**24.34**	-1578.95862	**0**	**23.74**	-1579.45197	-1579.06964	**24.80**
[Ar-O-$C_3H_7O_2$ * H^+GL * GL * N_2]		-1579.44077	**-34.90**								
**[Ar-O-$C_3H_7O_2$ * H^+GL * GL * N_2]**	**25**	-1579.44077	**-34.90**	-1578.97995	**-34.70**	-1579.04857	**0**	**-32.71**	-1579.55269	-1579.16049	**-32.21**
Ar-O-$CH_2CH(OH)CH_2OH$	**26**	-780.10171		-779.90096		-779.94395			-780.16000	-780.00224	
Cpl [GL * H^+GL]	**27**	-689.85037		-689.59938		-689.64410			-689.89837	-689.69210	
**Ar-O-$C_3H_7O_2$ + GL*H^+GL + N_2**		-1579.44308	**-36.34**	-1578.98575	**-38.33**	-1579.09189	**2**	**-56.10**	-1579.55729	-1579.20610	**-57.04**

[a] M06/6-311+G(d,p); [b] SP M06/6-311++G(3df,2pd); [c] SP enegies combined with thermal corrections calculated with 6-311+G(d,p).

Table S4. Calculated absolute and relative (in kcal mol^{-1}) energies for the reactions of solvated 4- methoxybenzendiazonium **1f'** in glycerol.

**S_N to Fluoride - GL-solvated**		E /au[a]	ΔE	E^{0K} /au	$ΔE^{0K}$	G^{298K} /au	Δn	$ΔG^{298K}$	E(SP) /au[b]	G(SP) /au[c]	$ΔG^{298K}$
Cpl [$ArN_2^+(GL)_3$]	**28**	-1489.423714		-1488.933633		-1489.004956			***-1489.531352***	-1489.112594	
Cpl [F^- $(GL)_2$]	**04**	-789.457753		-789.217273		-789.265350			-789.505814	-789.313411	
**[$ArN_2^+(GL)_3$] + [$F^-(GL)_2$]**		-2278.881467	**0.00**	-2278.150906	**0.00**	-2278.270306	**0**	**0.00**	-2279.037166	-2278.426005	**0.00**
Cpl [$ArN_2^+(GL)_2$ * $F^-(GL)_2$]	**29**	-1934.183523		-1933.570448		-1933.654427			-1934.314387	-1933.785291	
GL	**01**	-344.709929		-344.591964		-344.623072			-344.734295	-344.647438	
**Cpl [$ArN_2^+(GL)_2$ * $F^-(GL)_2$] + GL**		-2278.893453	**-7.52**	-2278.162412	**-7.22**	-2278.277499	**0**	**-4.51**	-2279.048683	-2278.432729	**-4.22**
$TS_{SN2'}$ 4-CH_3O-C_6H_4-NN^+ F^- $(GL)_4$	**30**	-1934.130889		-1933.523467		-1933.607838			-1934.260054	-1933.737003	
**$TS_{SN2'}$ + GL**		-2278.840818	**25.51**	-2278.115431	**22.26**	-2278.230910	**0**	**24.72**	-2278.994349	-2278.384441	**26.08**
Cpl [Ar-F * N_2 * $(GL)_4$]	**31**	-1934.233902		-1933.621677		-1933.707743			-1934.365647	-1933.839488	
**Cpl [Ar-F * N_2 * $(GL)_4$] + GL**		-2278.943832	**-39.13**	-2278.213641	**-39.37**	-2278.330815	**0**	**-37.97**	-2279.099942	-2278.486925	**-38.23**
Ar-F	**32**	-445.851116		-445.727490		-445.759886			-445.884539	-445.793309	
Cpl $(GL)_2$	**02**	-689.433030		-689.194711		-689.239166			-689.480155	-689.286292	
**Ar-F + N_2 + $2(GL)_2$ + GL**		-2278.918110	**-22.99**	-2278.194282	**-27.22**	-2278.365126	**3**	**-53.82**	-2279.078066	-2278.525081	**-56.49**

**S_N to Chloride - GL-solvated**		E /au[a]	ΔE	E^{0K} /au	$ΔE^{0K}$	G^{298K} /au	Δn	$ΔG^{298K}$	E(SP) /au[b]	G(SP) /au[c]	$ΔG^{298K}$
Cpl [Cl^- $(GL)_2$]	**09**	-1149.815987		-1149.577965		-1149.630877			-1149.865868	-1149.680758	
**[$ArN_2^+(GL)_3$] + [$Cl^-(GL)_2$]**		-2639.239700	**0.00**	-2638.511598	**0.00**	-2638.635833	**0**	**0.00**	-2639.397220	-2638.793352	**0.00**
Cpl [$ArN_2^+(GL)_2$ * $Cl^-(GL)_2$]	**33**	-2294.545734		-2293.934937		-2294.020757			-2294.677156	-2294.152179	
**Cpl [$ArN_2^+(GL)_2$ * $Cl^-(GL)_2$] + GL**		-2639.255664	**-10.02**	-2638.526901	**-9.60**	-2638.643829	**0**	**-5.02**	-2639.411451	-2638.799616	**-3.93**
$TS_{SN2'}$ 4-CH_3O-C_6H_4-NN^+ Cl^- $(GL)_4$	**34**	-2294.491712		-2293.887190		-2293.975233			-2294.621614	-2294.105135	
**$TS_{SN2'}$ + GL**		-2639.201641	**23.88**	-2638.479154	**20.36**	-2638.598305	**0**	**23.55**	-2639.355909	-2638.752573	**25.59**
Cpl [Ar-Cl * N_2 * $(GL)_4$]	**35**	-2294.591780		-2293.981456		-2294.066755			-2294.723184	-2294.198159	
**Cpl [Ar-Cl * N_2 * $(GL)_4$] + GL**		-2639.301709	**-38.91**	-2638.573420	**-38.79**	-2638.689827	**0**	**-33.88**	-2639.457479	-2638.845597	**-32.78**
Ar-Cl	**36**	-806.204816		-806.082520		-806.115843			-806.237826	-806.148853	

**Ar-Cl + N_2 + $2(GL)_2$ + GL**		-2639.271809	**-20.15**	-2638.549312	**-23.67**	-2638.721083	3	**-47.81**	-2639.431352	-2638.880626	**-49.08**

**S_N to Bromide - GL-solvated**		E /au[a]	ΔE	E^{0K} /au	$ΔE^{0K}$	G^{298K} /au	Δn	$ΔG^{298K}$	E(SP) /au[b]	G(SP) /au[c]	$ΔG^{298K}$
Cpl [Br^- $(GL)_2$]	14	-3263.638651		-3263.401376		-3263.455579			-3263.688563	-3263.505491	
**[$ArN_2^+(GL)_3$] + [$Br^-(GL)_2$]**		-4753.062364	**0.00**	-4752.335009	**0.00**	-4752.460535	0	**0.00**	-4753.219914	-4752.618085	**0.00**
Cpl [$ArN_2^+(GL)_2$ * $Br^-(GL)_2$]	37	-4408.367642		-4407.757246		-4407.843165			-4408.499305	-4407.974828	
**Cpl [$ArN_2^+(GL)_2$ * $Br^-(GL)_2$] + GL**		-4753.077571	**-9.54**	-4752.349210	**-8.91**	-4752.466237	0	**-3.58**	-4753.233600	-4752.622266	**-2.62**
$TS_{SN2'}$ 4-CH_3O-C_6H_4-NN^+ Br^- $(GL)_4$	38	-4408.313406		-4407.709238		-4407.798576			-4408.443813	-4407.928982	
**$TS_{SN2'}$ + GL**		-4753.023335	**24.49**	-4752.301202	**21.21**	-4752.421648	0	**24.40**	-4753.178108	-4752.576420	**26.15**
Cpl [Ar-Br * N_2 * $(GL)_4$]	39	-4408.416511		-4407.806832		-4407.893066			-4408.546165	-4408.022719	
**Cpl [Ar-Br * N_2 * $(GL)_4$] + GL**		-4753.126441	**-40.21**	-4752.398796	**-40.03**	-4752.516138	0	**-34.89**	-4753.280460	-4752.670157	**-32.68**
Ar-Br	40	-2920.030083		-2919.908415		-2919.942837			-2920.061058	-2919.973812	
**Ar-Br + N_2 + $2(GL)_2$ + GL**		-4753.097077	**-21.78**	-4752.375207	**-25.22**	-4752.548077	3	**-49.25**	-4753.254585	-4752.705585	**-49.22**

**Solvolysis**		E /au[a]	ΔE	E^{0K} /au	$ΔE^{0K}$	G^{298K} /au	Δn	$ΔG^{298K}$	E(SP) /au[b]	G(SP) /au[c]	$ΔG^{298K}$
**Cpl [$ArN_2^+(GL)_3$]**	28	-1489.423714	**0.00**	-1488.933633	**0.00**	-1489.004956	0	**0.00**	-1489.531352	-1489.112594	**0.00**
**TS_{NN} [ArN_2^+ * 3 GL]**	41	-1489.370850	**33.17**	-1488.886676	**29.47**	-1488.958969	0	**28.86**	-1489.476488	-1489.064606	**30.11**
[Ar-O-$C_3H_7O_2$ * H^+GL * GL * N_2]	42										
**[Ar-O-$C_3H_7O_2$ * H^+GL * GL * N_2]**		-1489.458946	**-22.11**	-1488.970718	**-23.27**	-1489.040430	0	**-22.26**	-1489.565127	-1489.146611	**-21.35**
Ar-O-$CH_2CH(OH)CH_2OH$	43	-690.115690		-689.885680		-689.928317			-690.167858	-689.980485	
Cpl [GL * H^+GL]	27	-689.850366		-689.599379		-689.644103			-689.898367	-689.692103	
**Ar-O-$C_3H_7O_2$ + GL*H^+GL + N_2**		-1489.457062	**-20.93**	-1488.970465	**-23.11**	-1489.076256	2	**-40.95**	-1489.565145	-1489.184340	**-41.23**

[a] M06/6-311+G(d,p); [b] SP M06/6-311++G(3df,2pd); [c] SP enegies combined with thermal corrections calculated with 6-311+G(d,p).

Table S5. Calculated absolute and relative (in kcal mol^{-1}) energies for the intramolecular fluorination in 4-nitrobenzendiazonium tetrafluoborate **1a** in glycerol**.**

**Intramolecular S_N to Fluoride**		E /au[a]	**ΔE**	E^{0K} /au	**ΔE^{0K}**	G^{298K} /au	**Δn**	**ΔG^{298K}**	E(SP) /au[b]	G(SP) /au[c]	**ΔG^{298K}**
4-O_2N-C_6H_4-NN^+ BF_4^- (**ArN_2 BF_4**)	**1a**	-969.844503	**0.00**	-969.728945	**0.00**	-969.773953	**0**	**0.00**	-969.916085	-969.845535	**0.00**
TS$_{ipso\text{-}Sst}$ 4-O_2N-C_6H_4-NN^+ BF_4^-	**44**	-969.795883	**30.51**	-969.686026	**26.93**	-969.732983	**0**	**25.71**	-969.866065	-969.803165	**26.59**
Cpl [*ipso*-Sst * N_2]	**45**	-969.881106	**-22.97**	-969.766834	**-23.78**	-969.816319	**0**	**-26.59**	-969.954860	-969.890073	**-27.95**
ipso-Sst 4-O_2N-C_6H_4-F---BF_3	**46**	-860.388067		-860.280793		-860.325503			-860.454154	-860.391591	
N_2		-109.491005		-109.485406		-109.503836			-109.498921	-109.511751	
ipso-Sst + N_2		-969.879072	**-21.69**	-969.766199	**-23.38**	-969.829339	**1**	**-32.86**	-969.953075	-969.903342	**-34.38**

[a] M06/6-311+G(d,p); [b] SP M06/6-311++G(3df,2pd); [c] SP enegies combined with thermal corrections calculated with 6-311+G(d,p).

Pictures and **Cartesian coordinates** for the solvation of **1a** in glycerol and N_2-scrambe (**Table 1**).

1a 4-NO2-C6H4-N2(+) * BF4(-)

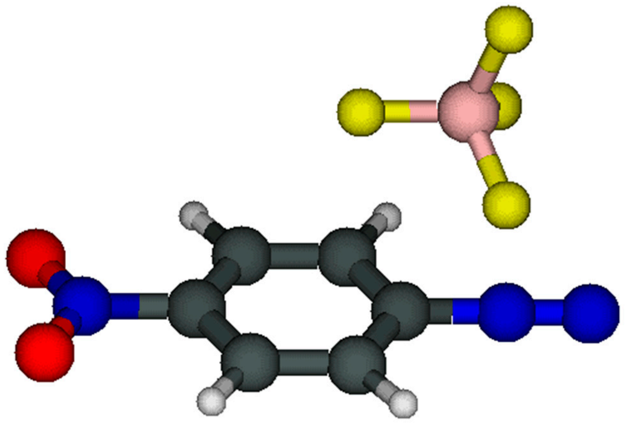

1	6	0	-0.000000	0.000000	-0.000000
2	6	0	-0.000000	0.000000	1.391963
3	6	0	1.230059	0.000000	2.016686
4	6	0	2.370630	-0.000647	1.230475
5	6	0	2.357142	0.001167	-0.154749
6	6	0	1.138318	0.001183	-0.801118
7	1	0	-0.927725	-0.014453	1.949516
8	1	0	1.308452	-0.004131	3.096079
9	7	0	3.690460	-0.008596	1.915528
10	1	0	3.284967	-0.002086	-0.711869
11	1	0	1.060346	-0.012384	-1.880695
12	7	0	-1.231335	-0.010247	-0.639130
13	7	0	-2.206184	-0.011675	-1.145131
14	8	0	4.678892	-0.010357	1.216866
15	8	0	3.687983	-0.011261	3.125949
16	9	0	-1.583221	-2.502642	0.461889
17	5	0	-0.631797	-3.180986	-0.329683
18	9	0	-0.532326	-2.501570	-1.562746
19	9	0	0.608290	-3.145975	0.314009
20	9	0	-1.036394	-4.494380	-0.540387

1a' 4-NO2-C6H4-N2(+)

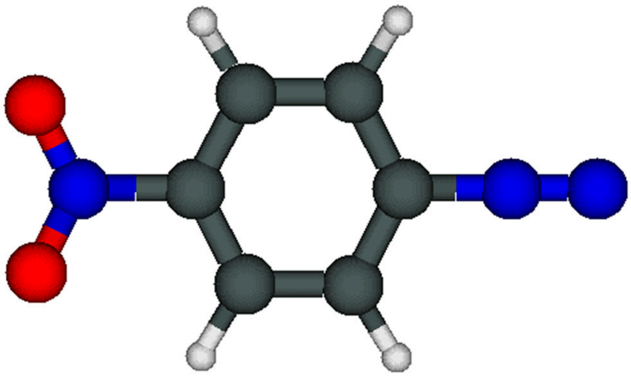

1	6	0	-0.000011	-0.000051	0.000690
2	6	0	-0.000030	0.000110	1.394548
3	6	0	1.229548	-0.000078	2.020414
4	6	0	2.371193	0.002967	1.235995
5	6	0	2.359317	0.005275	-0.149095

6	6	0	1.141996	0.001875	-0.798674
7	1	0	-0.926226	-0.000534	1.954799
8	1	0	1.305929	-0.002189	3.100021
9	7	0	3.690953	0.001476	1.924094
10	1	0	3.287773	0.008051	-0.705335
11	1	0	1.072655	0.000685	-1.878916
12	7	0	-1.231483	-0.003143	-0.639504
13	7	0	-2.206486	-0.006094	-1.147245
14	8	0	4.677723	0.076336	1.228338
15	8	0	3.684988	-0.075345	3.131449

TS_N2-Scramble in 4-NO2-C6H4-N2(+)

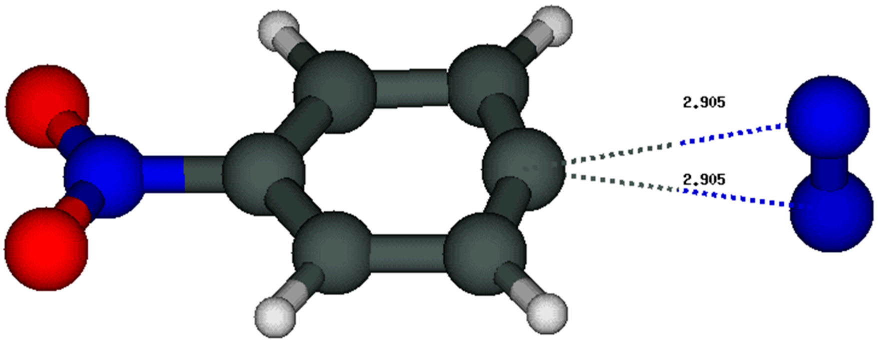

1	6	0	-0.000000	0.000000	0.000000
2	6	0	-0.000000	-1.271547	-0.356357
3	6	0	-0.000000	-1.220985	-1.768310
4	1	0	-0.000000	-2.179277	0.232714
5	1	0	-0.000000	-2.163108	-2.308344
6	6	0	-0.000000	1.271547	-0.356357
7	6	0	-0.000000	1.220985	-1.768310
8	1	0	-0.000000	2.179277	0.232714
9	1	0	-0.000000	2.163108	-2.308344
10	6	0	-0.000000	0.000000	-2.424055
11	7	0	0.000000	0.000000	-3.904010
12	7	0	0.547926	-0.000000	2.853258
13	7	0	-0.547926	-0.000000	2.853258
14	8	0	0.000000	-1.077055	-4.453435
15	8	0	0.000000	1.077055	-4.453435

Pictures and **Cartesian coordinates** of the structures related to the solvation of **1f** (**Table 2**).

1f 4-CH3O-C6H4-N2(+) * BF4(-)

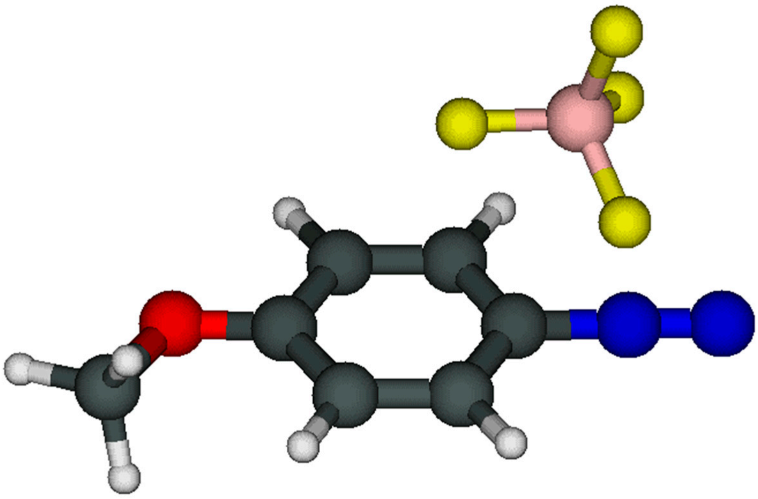

1	6	0	-0.000706	0.017627	0.045256
2	6	0	0.008559	0.083485	1.443053
3	6	0	1.217833	0.058344	2.089465
4	6	0	2.404122	-0.036407	1.341653
5	6	0	2.365473	-0.097482	-0.065350
6	6	0	1.171194	-0.075533	-0.724528
7	1	0	-0.923307	0.144505	1.992297
8	1	0	1.240572	0.106063	3.170296
9	8	0	3.611950	-0.074638	1.876211
10	1	0	3.302290	-0.170034	-0.605209
11	1	0	1.114198	-0.135202	-1.804497
12	7	0	-1.199494	0.029674	-0.594085
13	7	0	-2.173113	0.048188	-1.117168
14	9	0	-1.750494	-2.446328	0.590014
15	5	0	-0.804331	-3.183450	-0.148589
16	9	0	-0.673841	-2.592884	-1.420849
17	9	0	0.428991	-3.142533	0.509543
18	9	0	-1.234641	-4.503806	-0.282271
19	6	0	3.759747	-0.034925	3.294671
20	1	0	4.829689	-0.088318	3.482742
21	1	0	3.361809	0.899939	3.698928
22	1	0	3.261677	-0.890799	3.758396

1f' 4-CH3O-C6H4-N2(+)

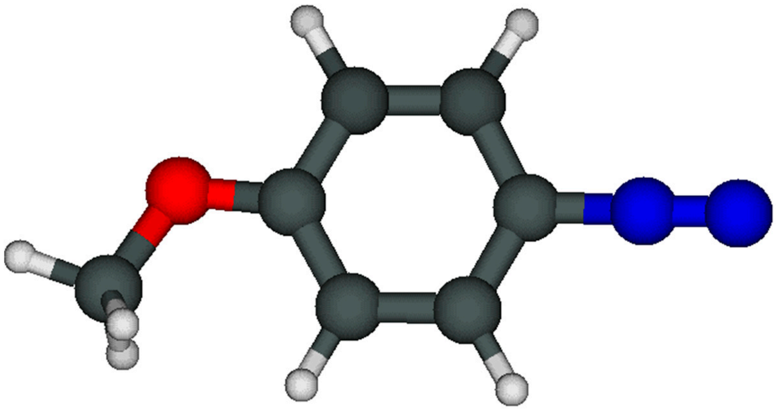

1	6	0	0.002241	-0.019695	-0.001708
2	6	0	-0.002007	-0.022381	1.400513
3	6	0	1.201422	-0.038482	2.056341
4	6	0	2.398635	-0.051824	1.317458
5	6	0	2.373548	-0.048709	-0.092502
6	6	0	1.186308	-0.032770	-0.763205
7	1	0	-0.938190	-0.011908	1.945844
8	1	0	1.212894	-0.040704	3.138337
9	8	0	3.599442	-0.067643	1.860243
10	1	0	3.316997	-0.059261	-0.625388
11	1	0	1.143277	-0.030098	-1.845721
12	7	0	-1.189615	-0.003740	-0.653434
13	7	0	-2.158502	0.009340	-1.188134
14	6	0	3.738016	-0.072090	3.281637
15	1	0	4.808198	-0.085639	3.473954
16	1	0	3.297222	0.830173	3.713485
17	1	0	3.275133	-0.964901	3.710009

Pictures and **Cartesian coordinates** for the reaction of **1a'** in glycerol (see labels in **Table 3**).

01 GL

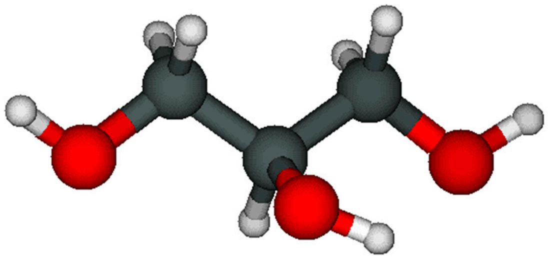

1	6	0	-0.006534	-0.000432	-0.003416
2	6	0	-0.002604	-0.000160	1.500712
3	6	0	1.402874	0.008849	-0.550815
4	8	0	-0.697482	-1.149048	-0.455918
5	1	0	-0.524408	0.909778	-0.353780
6	8	0	-1.318065	0.232367	1.959199
7	1	0	0.376231	-0.971965	1.852026
8	1	0	0.685272	0.782495	1.854720
9	8	0	1.288133	-0.079616	-1.959117
10	1	0	1.917921	0.931122	-0.248195
11	1	0	1.957726	-0.851361	-0.147394
12	1	0	-0.515511	-1.223950	-1.400580
13	1	0	2.136549	-0.324388	-2.336402
14	1	0	-1.329503	0.160190	2.916890

02 (GL)2

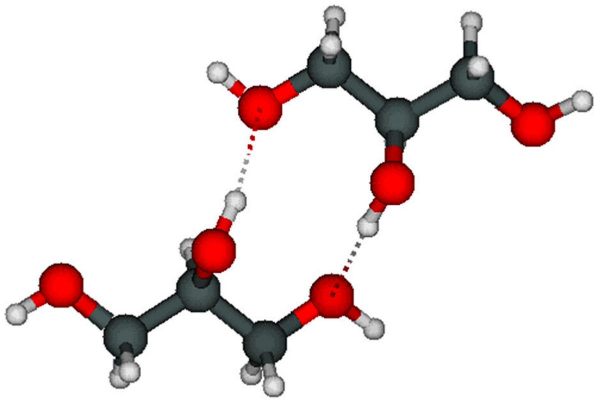

1	1	0	0.000000	-0.000000	0.000000
2	8	0	0.000000	-0.000000	0.972771
3	6	0	1.340392	-0.000000	1.414053
4	6	0	1.813752	1.409710	1.690875
5	6	0	1.448838	-0.840021	2.662938
6	1	0	1.988842	-0.440165	0.638446
7	8	0	1.733074	2.150790	0.482518
8	1	0	1.177615	1.862625	2.466390
9	1	0	2.848629	1.390116	2.057128
10	1	0	2.087275	3.032473	0.629676
11	8	0	0.381891	-0.265562	-1.788231
12	6	0	-0.103773	0.553843	-2.841117
13	6	0	0.218997	1.993485	-2.507615
14	1	0	0.372061	0.275558	-3.790648
15	1	0	-1.192985	0.439781	-2.949971
16	8	0	-0.409557	2.381424	-1.305162

17	1	0	1.312536	2.091726	-2.405904
18	6	0	-0.242381	2.901678	-3.620851
19	1	0	0.223244	2.256187	-0.577027
20	1	0	0.238749	-1.188653	-2.016035
21	8	0	0.320406	4.182296	-3.423873
22	1	0	0.065823	2.480735	-4.589459
23	1	0	-1.342356	2.943632	-3.604015
24	1	0	-0.057759	4.788751	-4.065425
25	1	0	2.429526	-0.673728	3.133011
26	1	0	0.673037	-0.512914	3.372256
27	8	0	1.279954	-2.197909	2.312089
28	1	0	1.238296	-2.722474	3.115509

03 Cpl 4-NO2-C6H4-N2(+) * 3 GL

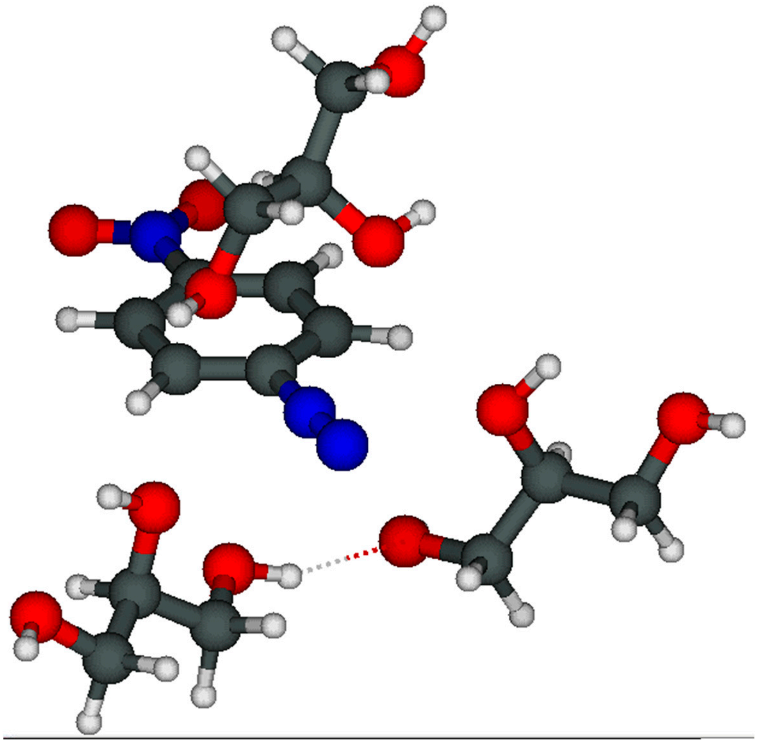

1	6	0	-0.014514	-0.095750	-0.042474
2	6	0	-0.046714	-0.159011	1.345852
3	6	0	1.170488	-0.125215	1.997836
4	6	0	2.326728	-0.025606	1.239918
5	6	0	2.340950	0.050631	-0.144295
6	6	0	1.135475	0.015770	-0.816890
7	1	0	-1.000113	-0.221326	1.859911
8	1	0	1.228475	-0.172434	3.077478
9	7	0	3.627986	0.007391	1.953747
10	1	0	3.278497	0.133571	-0.678477
11	1	0	1.054255	0.069735	-1.897313
12	7	0	-1.225934	-0.166898	-0.718004
13	7	0	-2.169230	-0.242118	-1.272641
14	8	0	4.628146	0.154537	1.285946
15	8	0	3.607609	-0.115184	3.158893
16	8	0	-1.717482	2.325846	0.598482
17	6	0	-3.119277	2.377670	0.709743
18	6	0	-3.543281	1.474519	1.836925
19	1	0	-3.471012	3.400285	0.916541

20	1	0	-3.608167	2.039553	-0.219464
21	6	0	-5.018447	1.609104	2.131042
22	8	0	-3.238147	0.134535	1.479154
23	1	0	-2.973357	1.749715	2.741749
24	8	0	-5.316461	0.653675	3.132097
25	1	0	-5.240950	2.630196	2.469560
26	1	0	-5.594646	1.410026	1.215092
27	1	0	-3.726987	-0.429801	2.091947
28	1	0	-6.268282	0.545064	3.199989
29	1	0	-1.438729	2.663397	-0.268985
30	8	0	-1.676378	-2.686265	0.587582
31	6	0	-1.148719	-3.847285	-0.023434
32	6	0	0.105338	-3.465277	-0.760468
33	1	0	-0.905824	-4.615424	0.725309
34	1	0	-1.864479	-4.278185	-0.738976
35	6	0	0.822051	-4.680323	-1.301283
36	8	0	-0.242836	-2.590413	-1.819359
37	1	0	0.783781	-2.943505	-0.059193
38	8	0	1.914108	-4.191334	-2.057694
39	1	0	1.158266	-5.317600	-0.472355
40	1	0	0.134815	-5.260790	-1.934363
41	1	0	2.269188	-4.897052	-2.604022
42	1	0	-2.527572	-2.898529	0.979428
43	8	0	-0.624107	2.612089	-1.931983
44	6	0	-1.227687	2.524893	-3.212364
45	6	0	-0.501103	1.562492	-4.118113
46	1	0	-2.250755	2.166659	-3.047818
47	1	0	-1.287031	3.512323	-3.688477
48	6	0	-1.117758	1.541519	-5.497828
49	1	0	0.554816	1.876427	-4.208667
50	8	0	-0.560545	0.271869	-3.536247
51	1	0	-0.346388	-0.357634	-4.237726
52	8	0	-0.441509	0.530891	-6.220701
53	1	0	-2.190737	1.313597	-5.413865
54	1	0	-1.004571	2.524677	-5.974396
55	1	0	-0.937793	0.314690	-7.014208
56	1	0	0.230378	3.049771	-2.008873
57	1	0	0.504879	-2.594745	-2.431816

04 Cpl F(-) * 2 GL

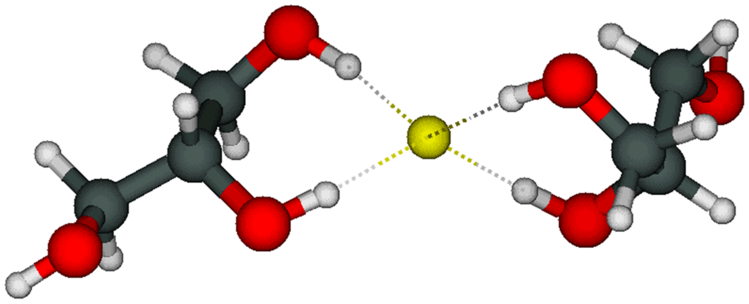

1	9	0	0.215701	-0.135157	-0.065809
2	1	0	0.091693	-0.041022	1.604364
3	8	0	0.225711	0.007809	2.576007
4	6	0	1.324415	-0.806075	2.914314
5	6	0	2.607214	-0.459066	2.174190
6	1	0	1.492521	-0.685986	3.991714
7	1	0	1.106159	-1.870924	2.732546

8	6	0	2.917893	1.019210	2.273546
9	8	0	2.598626	-0.906463	0.833867
10	1	0	1.770969	-0.596941	0.405271
11	1	0	3.425183	-1.003393	2.670353
12	8	0	4.259422	1.227578	1.870029
13	1	0	4.419708	2.172424	1.809908
14	1	0	2.762997	1.353207	3.311582
15	1	0	2.222389	1.581240	1.633791
16	1	0	-0.333821	1.063407	-1.090333
17	8	0	-0.781292	1.633478	-1.753161
18	6	0	-1.037515	0.870453	-2.906051
19	6	0	-1.886122	-0.363107	-2.639644
20	1	0	-0.099934	0.545186	-3.390367
21	1	0	-1.563058	1.525649	-3.611621
22	8	0	-1.163813	-1.370632	-1.966534
23	1	0	-0.687249	-0.980341	-1.202027
24	1	0	-2.758892	-0.048324	-2.036974
25	6	0	-2.393971	-0.938828	-3.937818
26	8	0	-3.306088	-1.982666	-3.657251
27	1	0	-3.575762	-2.386186	-4.485906
28	1	0	-1.536266	-1.309864	-4.521015
29	1	0	-2.881301	-0.141043	-4.517482

----

05 Cpl 4-NO2-C6H4-N2(+) * F(-) * 4 GL

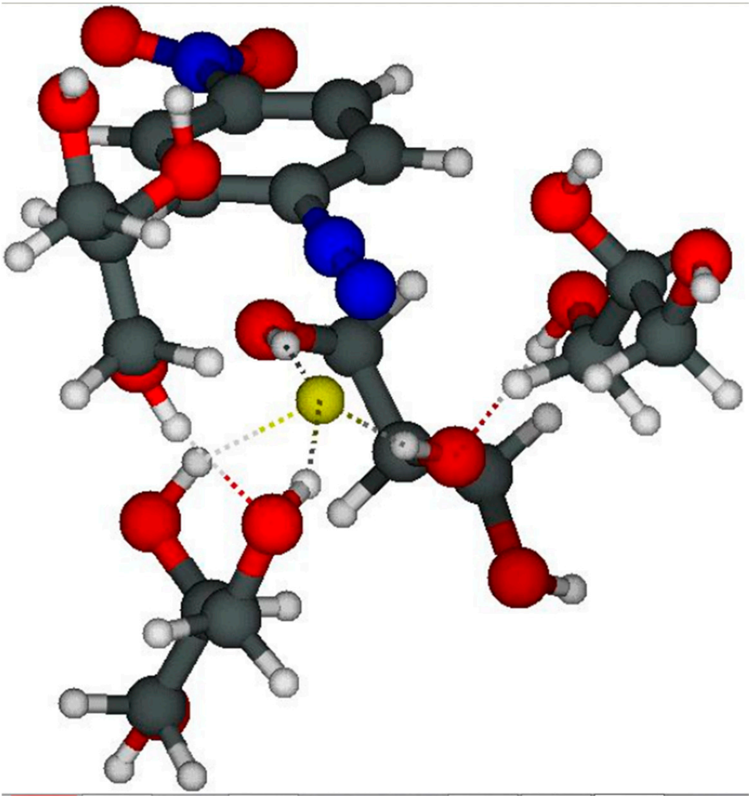

1	6	0	-0.057383	-0.079434	-0.076270
2	6	0	-0.033853	0.034644	1.311365
3	6	0	1.206494	-0.013508	1.915276
4	6	0	2.325971	-0.160067	1.114319
5	6	0	2.288500	-0.227673	-0.269060
6	6	0	1.059965	-0.200655	-0.896865
7	1	0	-0.960527	0.115968	1.871834
8	1	0	1.304866	0.036972	2.992425

9	7	0	3.638112	-0.311839	1.779536
10	1	0	3.204076	-0.340482	-0.835365
11	1	0	0.933066	-0.314255	-1.967781
12	9	0	-0.794243	-2.743766	-0.185315
13	7	0	-1.311423	-0.157225	-0.665195
14	7	0	-2.329509	-0.229586	-1.069082
15	8	0	4.631958	-0.134575	1.108704
16	8	0	3.632979	-0.619575	2.951907
17	8	0	-0.533104	-1.302387	-3.341658
18	6	0	-1.277109	-0.465759	-4.194659
19	6	0	-0.661328	0.908424	-4.189465
20	1	0	-1.286054	-0.849341	-5.226711
21	1	0	-2.325522	-0.388281	-3.860712
22	6	0	-1.292715	1.805926	-5.226839
23	8	0	-0.824951	1.468287	-2.895687
24	1	0	0.415833	0.811559	-4.414035
25	1	0	-0.590146	2.401906	-2.965631
26	1	0	-1.074090	-2.082741	-3.107263
27	8	0	-0.691936	3.079041	-5.077184
28	1	0	-1.120797	1.395936	-6.231575
29	1	0	-2.377395	1.860434	-5.051260
30	1	0	-1.213074	3.736946	-5.544007
31	1	0	-1.069600	-3.701514	1.119299
32	8	0	-1.125657	-4.125660	2.006930
33	6	0	0.132668	-4.683442	2.349528
34	6	0	1.216436	-3.619365	2.400542
35	6	0	-0.010095	-5.360469	3.688474
36	1	0	0.419420	-5.438190	1.595428
37	1	0	2.129483	-4.045906	2.831464
38	8	0	1.558850	-3.115276	1.132274
39	1	0	0.878622	-2.808698	3.073899
40	1	0	0.741900	-2.849698	0.666616
41	1	0	0.980798	-5.694873	4.027882
42	1	0	-0.388371	-4.624950	4.417236
43	8	0	-0.897073	-6.452128	3.561021
44	1	0	-1.023795	-6.848501	4.426754
45	1	0	-1.848720	-3.125137	-1.432638
46	8	0	-2.176296	-3.282872	-2.346501
47	6	0	-1.913522	-4.623668	-2.717814
48	6	0	-0.774861	-5.243474	-1.922123
49	1	0	-2.816918	-5.237668	-2.591047
50	1	0	-1.654410	-4.626093	-3.786314
51	6	0	-0.431137	-6.599119	-2.480851
52	8	0	0.380175	-4.432321	-1.953513
53	1	0	-1.105170	-5.378047	-0.875972
54	1	0	0.202982	-3.686537	-1.352074
55	8	0	0.397031	-7.277861	-1.558924
56	1	0	-1.359077	-7.160314	-2.666756
57	1	0	0.079018	-6.458867	-3.446294
58	1	0	0.669477	-8.113954	-1.945365
59	8	0	-2.163412	-1.764404	3.030537
60	6	0	-3.287226	-1.792195	2.182931
61	6	0	-3.971124	-0.452737	2.211575
62	1	0	-2.990169	-2.020659	1.144365
63	1	0	-4.008206	-2.563551	2.497573
64	6	0	-5.287572	-0.485812	1.471526
65	1	0	-4.160956	-0.172113	3.261418
66	8	0	-3.107798	0.505125	1.615935
67	1	0	-3.618723	1.317647	1.512185

68	8	0	-5.798101	0.834160	1.499398
69	1	0	-5.116980	-0.819298	0.436303
70	1	0	-5.974019	-1.192643	1.957689
71	1	0	-6.514309	0.915448	0.864685
72	1	0	-1.683579	-2.599616	2.875957

----

06 TS_SN 4-NO2-C6H4-N2(+) * F(-) * 4 GL

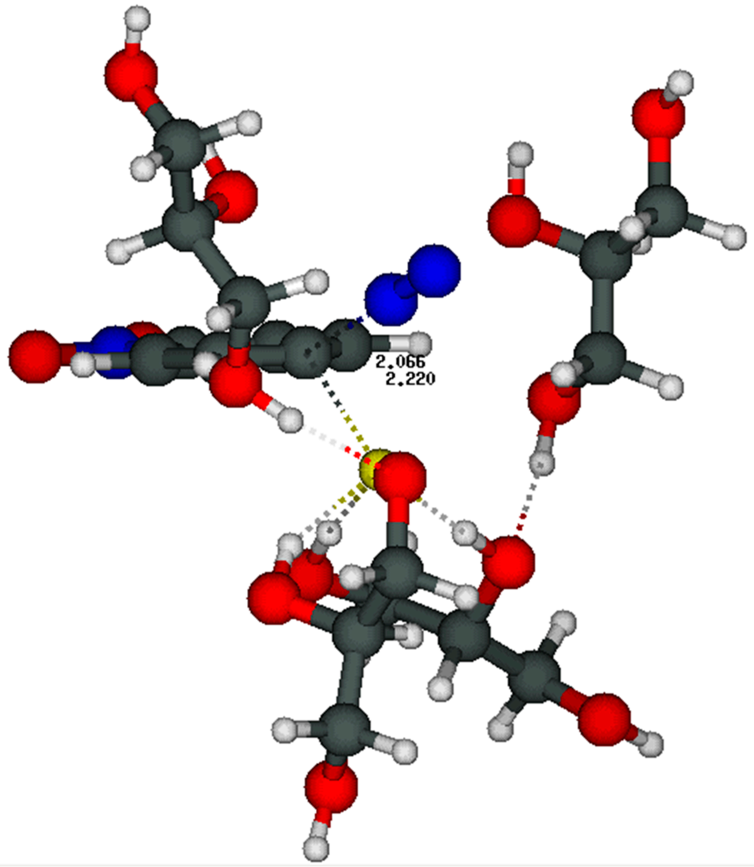

1	6	0	0.000000	0.000000	0.000000
2	6	0	0.000000	0.000000	1.342492
3	6	0	1.319076	0.000000	1.805613
4	6	0	2.368305	0.014248	0.897582
5	6	0	2.188448	0.021072	-0.479530
6	6	0	0.894205	0.021843	-1.002652
7	1	0	-0.881057	-0.038705	1.984550
8	1	0	1.510614	-0.018120	2.872051
9	7	0	3.744749	0.016695	1.421876
10	1	0	3.040159	0.026495	-1.149156
11	1	0	0.626443	0.025689	-2.060464
12	9	0	-1.334136	-1.691973	-0.532626
13	7	0	-1.774682	0.778577	-0.716918
14	7	0	-2.700314	1.204729	-1.112868
15	8	0	4.653237	0.042954	0.617942
16	8	0	3.888863	-0.006751	2.626510
17	8	0	-0.629269	-0.146544	-3.655135
18	6	0	-1.125256	1.021591	-4.267218
19	6	0	-0.178857	2.162598	-4.001856
20	1	0	-1.224488	0.884870	-5.355148
21	1	0	-2.119569	1.284238	-3.872197
22	6	0	-0.526964	3.374985	-4.832921
23	8	0	-0.235590	2.481557	-2.622466

24	1	0	0.843634	1.837273	-4.265209
25	1	0	0.246333	3.309855	-2.509804
26	1	0	-1.372748	-0.752976	-3.466534
27	8	0	0.374320	4.397849	-4.451128
28	1	0	-0.434016	3.136520	-5.901491
29	1	0	-1.566942	3.671312	-4.628982
30	1	0	0.062456	5.242536	-4.785105
31	1	0	-2.090200	-2.720558	0.589922
32	8	0	-2.389533	-3.232001	1.370451
33	6	0	-1.473790	-4.283197	1.649978
34	6	0	-0.070450	-3.749700	1.878162
35	6	0	-1.973789	-5.012286	2.870574
36	1	0	-1.448395	-4.986296	0.799040
37	1	0	0.567119	-4.551212	2.266916
38	8	0	0.540854	-3.278850	0.700081
39	1	0	-0.116681	-2.958372	2.648756
40	1	0	-0.055023	-2.641617	0.270155
41	1	0	-1.218812	-5.749901	3.178039
42	1	0	-2.093588	-4.287392	3.692610
43	8	0	-3.200052	-5.639462	2.561545
44	1	0	-3.540183	-6.059794	3.355441
45	1	0	-2.474306	-1.683928	-1.861613
46	8	0	-2.760306	-1.687682	-2.796733
47	6	0	-2.826847	-3.026948	-3.254118
48	6	0	-1.821409	-3.928119	-2.555260
49	1	0	-3.838995	-3.431537	-3.111609
50	1	0	-2.618493	-3.010940	-4.332441
51	6	0	-1.798362	-5.288945	-3.199151
52	8	0	-0.520823	-3.379189	-2.607070
53	1	0	-2.129221	-4.056105	-1.501519
54	1	0	-0.493566	-2.675072	-1.941035
55	8	0	-1.116441	-6.184566	-2.345835
56	1	0	-2.831570	-5.621761	-3.379202
57	1	0	-1.295187	-5.204067	-4.174556
58	1	0	-1.030356	-7.031541	-2.790647
59	8	0	-2.543686	-0.966239	2.946985
60	6	0	-3.637596	-0.376240	2.281135
61	6	0	-3.672595	1.094479	2.596419
62	1	0	-3.545415	-0.509108	1.189400
63	1	0	-4.589388	-0.832901	2.592739
64	6	0	-4.904606	1.753739	2.022279
65	1	0	-3.683604	1.220358	3.692997
66	8	0	-2.503553	1.690138	2.060763
67	1	0	-2.619727	2.645682	2.126605
68	8	0	-4.800157	3.132732	2.324131
69	1	0	-4.933337	1.591580	0.934105
70	1	0	-5.808248	1.312543	2.465469
71	1	0	-5.434039	3.625287	1.797017
72	1	0	-2.446803	-1.865216	2.578316

----

07 Cpl 4-NO2-C6H4-F * N2 * 4 GL

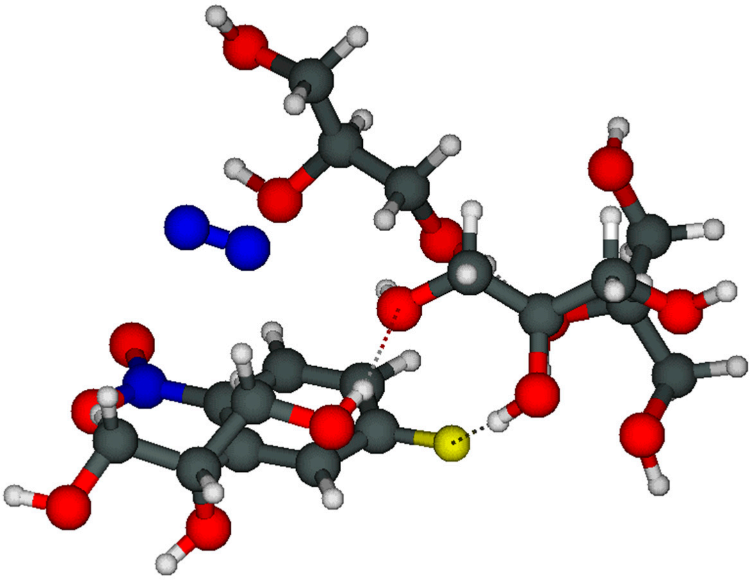

1	6	0	0.115634	-0.604877	-0.372709
2	6	0	-0.040052	-0.218577	0.945023
3	6	0	1.000844	0.450890	1.563140
4	6	0	2.157663	0.693224	0.836644
5	6	0	2.306815	0.293457	-0.484618
6	6	0	1.262599	-0.367860	-1.106157
7	1	0	-0.959763	-0.421252	1.482989
8	1	0	0.891192	0.814018	2.578968
9	7	0	3.242016	1.448107	1.470940
10	1	0	3.211790	0.507708	-1.041895
11	1	0	1.332222	-0.662196	-2.148245
12	9	0	-0.912759	-1.232735	-0.974867
13	7	0	0.591757	3.303469	0.015936
14	7	0	1.338431	4.074240	0.232834
15	8	0	4.224581	1.710556	0.801618
16	8	0	3.099230	1.789567	2.630194
17	8	0	0.782225	1.150655	-4.002431
18	6	0	1.755178	2.066260	-3.562025
19	6	0	3.118822	1.636150	-4.034689
20	1	0	1.555100	3.079672	-3.946254
21	1	0	1.765135	2.128105	-2.461651
22	6	0	4.159990	2.672511	-3.679864
23	8	0	3.447673	0.396451	-3.432438
24	1	0	3.094525	1.519789	-5.132421
25	1	0	4.382901	0.243725	-3.614375
26	1	0	0.004028	1.278617	-3.436408
27	8	0	5.407707	2.159479	-4.108613
28	1	0	3.928042	3.626996	-4.172485
29	1	0	4.154482	2.830334	-2.589329
30	1	0	6.118148	2.663231	-3.703836
31	1	0	-2.837273	-1.247386	0.217641
32	8	0	-3.375603	-0.679449	0.789642
33	6	0	-4.732535	-1.029938	0.536657
34	6	0	-4.829655	-2.523547	0.324296
35	6	0	-5.571883	-0.573823	1.696148
36	1	0	-5.071844	-0.520665	-0.380489
37	1	0	-5.842211	-2.788297	-0.008747
38	8	0	-3.858161	-2.844253	-0.654970
39	1	0	-4.621017	-3.048467	1.268375

40	1	0	-3.701454	-3.791866	-0.657151
41	1	0	-6.596573	-0.952305	1.564098
42	1	0	-5.167199	-1.006208	2.624731
43	8	0	-5.542026	0.837484	1.736031
44	1	0	-5.998316	1.135990	2.527038
45	1	0	-1.166917	1.873018	-1.194630
46	8	0	-1.210167	1.810989	-2.154341
47	6	0	-2.551163	1.951094	-2.598655
48	6	0	-3.281239	0.622553	-2.570298
49	1	0	-3.083700	2.700277	-2.000338
50	1	0	-2.497966	2.312461	-3.633273
51	6	0	-4.666586	0.783546	-3.140337
52	8	0	-2.587096	-0.340498	-3.340934
53	1	0	-3.358314	0.280505	-1.523532
54	1	0	-1.789370	-0.588633	-2.860555
55	8	0	-5.413645	-0.389815	-2.899869
56	1	0	-5.142574	1.658458	-2.672112
57	1	0	-4.577412	0.984887	-4.219242
58	1	0	-6.272772	-0.299862	-3.320263
59	8	0	-2.421776	0.655961	3.061605
60	6	0	-2.317996	1.964919	2.556353
61	6	0	-1.659503	2.852100	3.578252
62	1	0	-1.709021	1.987107	1.634158
63	1	0	-3.311819	2.370127	2.310244
64	6	0	-1.733561	4.304236	3.164637
65	1	0	-2.187558	2.733723	4.540581
66	8	0	-0.306949	2.456741	3.726652
67	1	0	0.133504	3.158295	4.222005
68	8	0	-1.011744	5.039561	4.135612
69	1	0	-1.284223	4.426011	2.167353
70	1	0	-2.782718	4.627087	3.115790
71	1	0	-0.826269	5.920000	3.800103
72	1	0	-2.865197	0.121964	2.380760

08 4-NO2-C6H4-F

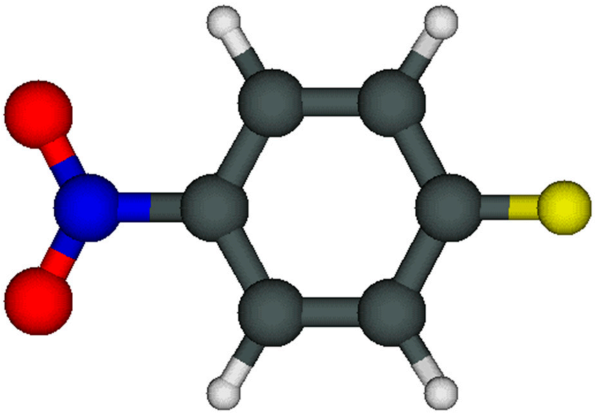

1	6	0	0.040893	0.000000	0.023609
2	6	0	0.004378	0.000000	1.407130
3	6	0	1.201562	-0.000000	2.097757
4	6	0	2.389290	-0.000000	1.379457
5	6	0	2.417492	0.000000	-0.008296
6	6	0	1.220799	0.000000	-0.699774
7	1	0	-0.948960	-0.000000	1.922232
8	1	0	1.222032	-0.000000	3.180318

9	7	0	3.657729	-0.000000	2.111791
10	1	0	3.365253	0.000000	-0.531848
11	1	0	1.190221	0.000000	-1.782940
12	9	0	-1.113879	0.000000	-0.643098
13	8	0	4.689887	-0.000000	1.467132
14	8	0	3.615518	-0.000000	3.327995

r00 4-NO2-C6H4 Rad.

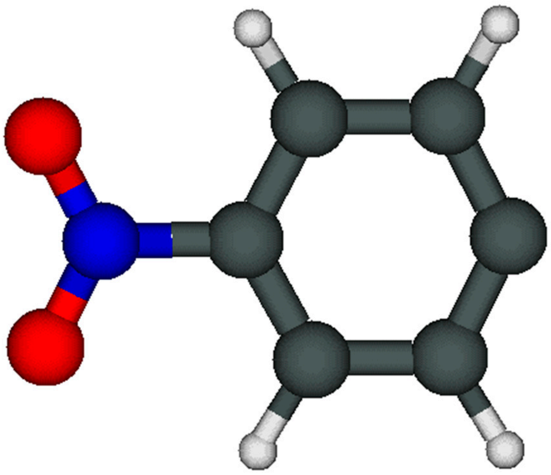

1	6	0	-0.000000	0.000000	0.000000
2	6	0	-0.000000	0.000000	1.371017
3	6	0	1.243598	0.000000	1.993689
4	6	0	2.383855	-0.000000	1.199653
5	6	0	2.342121	-0.000000	-0.189209
6	6	0	1.101059	-0.000000	-0.816919
7	1	0	-0.912869	0.000000	1.957178
8	1	0	1.334105	0.000000	3.073482
9	7	0	3.696377	-0.000000	1.860168
10	1	0	3.263228	-0.000000	-0.759916
11	1	0	1.027871	-0.000000	-1.899305
12	8	0	4.689499	-0.000000	1.157998
13	8	0	3.724217	-0.000000	3.076128

r01 Cpl 4-NO2-C6H4 Rad. F(-) * 2 GL

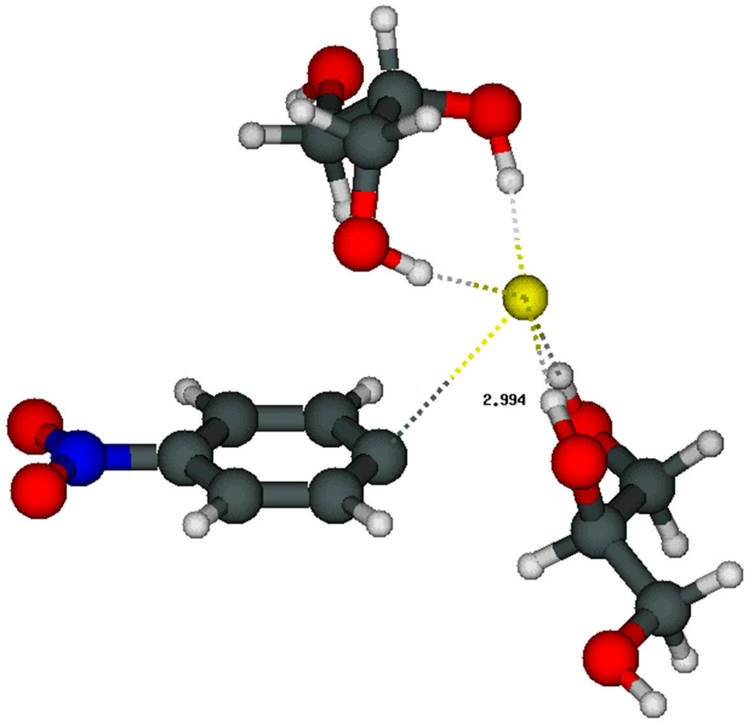

1	9	0	0.179110	-0.041148	-0.304783
2	1	0	-0.025966	-0.521877	1.315221
3	8	0	0.056065	-0.768683	2.261369
4	6	0	1.405163	-1.071810	2.529988
5	6	0	2.380188	0.028742	2.141661
6	1	0	1.482931	-1.250235	3.609645
7	1	0	1.718815	-1.997457	2.020446
8	6	0	1.938594	1.371914	2.683448
9	8	0	2.618811	0.076666	0.749567
10	1	0	1.753938	0.093545	0.284160
11	1	0	3.347513	-0.215061	2.606709
12	8	0	3.029829	2.271547	2.607238
13	1	0	2.717304	3.154549	2.818705
14	1	0	1.597809	1.252558	3.724454
15	1	0	1.085126	1.735624	2.093214
16	1	0	-0.412811	1.188319	-1.277953
17	8	0	-0.893548	1.776685	-1.900902
18	6	0	-1.610148	0.972940	-2.805546
19	6	0	-2.650083	0.090099	-2.134614
20	1	0	-0.932969	0.327991	-3.393241
21	1	0	-2.112174	1.649950	-3.507415
22	8	0	-2.065944	-0.973228	-1.414461
23	1	0	-1.271319	-0.654960	-0.934812
24	1	0	-3.241080	0.733448	-1.453561
25	6	0	-3.591671	-0.495204	-3.156323
26	8	0	-4.642164	-1.161827	-2.482615
27	1	0	-5.205027	-1.587130	-3.134220
28	1	0	-3.032212	-1.190816	-3.801440
29	1	0	-3.985791	0.315074	-3.787476
30	6	0	-2.275475	1.169512	0.909978
31	6	0	-1.681149	2.270837	1.471726
32	6	0	-2.027176	2.570985	2.783208
33	6	0	-2.942794	1.749641	3.432193
34	6	0	-3.533282	0.645357	2.827857

35	6	0	-3.186928	0.342680	1.516688
36	1	0	-0.967081	2.880694	0.926695
37	1	0	-1.599330	3.420942	3.302000
38	7	0	-3.307854	2.066911	4.817126
39	1	0	-4.244300	0.042191	3.380021
40	1	0	-3.615770	-0.513207	1.003705
41	8	0	-4.098271	1.335156	5.384035
42	8	0	-2.802897	3.046692	5.333916

r02 TS_CX

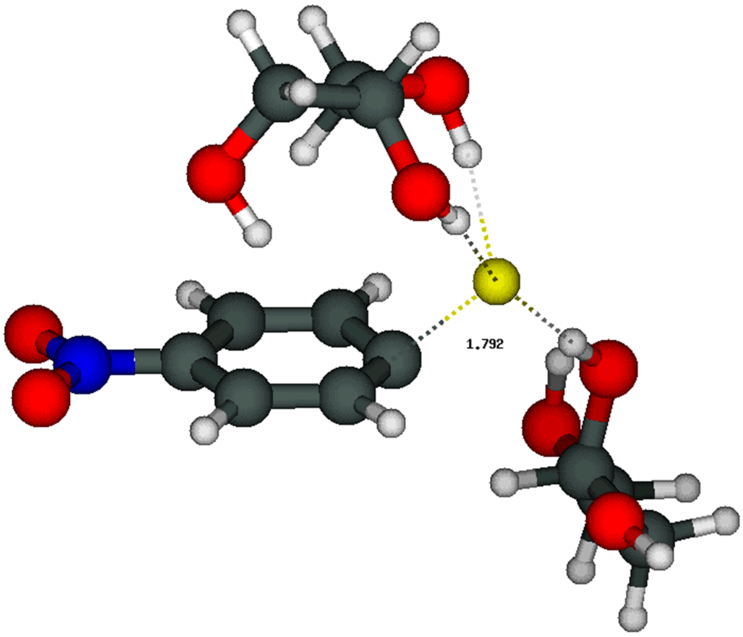

1	9	0	-0.146903	0.669444	0.548151
2	1	0	0.054362	-0.312262	4.529717
3	8	0	0.863734	-0.460835	5.037392
4	6	0	1.554958	-1.503447	4.379088
5	6	0	1.567670	-1.305564	2.877119
6	1	0	2.578028	-1.520771	4.769452
7	1	0	1.093882	-2.477414	4.602244
8	6	0	2.332333	-0.057820	2.474891
9	8	0	0.206492	-1.274690	2.477728
10	1	0	0.076300	-0.680723	1.718498
11	1	0	2.072727	-2.172464	2.417115
12	8	0	2.467073	0.078252	1.076494
13	1	0	1.615987	0.385420	0.720504
14	1	0	3.343509	-0.128998	2.892904
15	1	0	1.855176	0.832579	2.911760
16	1	0	0.323284	4.017547	-2.041993
17	8	0	-0.282846	4.764969	-2.127364
18	6	0	-1.546998	4.199363	-2.393259
19	6	0	-1.806154	3.011917	-1.492820
20	1	0	-1.615911	3.874431	-3.445390
21	1	0	-2.301769	4.974845	-2.230683
22	8	0	-0.720692	2.126092	-1.687890
23	1	0	-0.619547	1.550689	-0.907173
24	1	0	-1.819552	3.350877	-0.442689
25	6	0	-3.128817	2.359493	-1.807680
26	8	0	-3.368759	1.357578	-0.838945
27	1	0	-4.141547	0.850431	-1.099574

28	1	0	-3.081460	1.928969	-2.819542
29	1	0	-3.923730	3.121646	-1.796338
30	6	0	-0.649273	2.039583	1.588070
31	6	0	0.412981	2.926665	1.747451
32	6	0	0.625732	3.468672	2.994737
33	6	0	-0.177302	3.061499	4.073106
34	6	0	-1.198572	2.111353	3.897258
35	6	0	-1.414532	1.567747	2.652129
36	1	0	1.024849	3.223354	0.898892
37	1	0	1.391108	4.219315	3.155958
38	7	0	0.040705	3.620686	5.358595
39	1	0	-1.818012	1.836204	4.743980
40	1	0	-2.193103	0.826608	2.494721
41	8	0	-0.653241	3.232188	6.305382
42	8	0	0.921917	4.476339	5.498977

----

r03 Cpl 4-NO2-C6H4-F(*-) * 2 GL

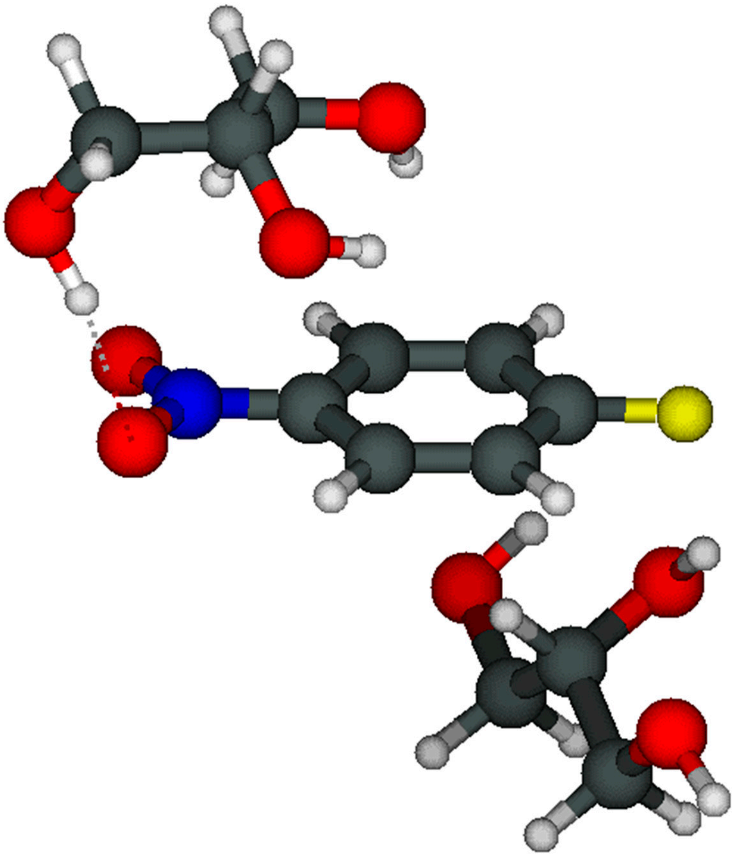

1	9	0	-0.894213	-0.382207	-0.006469
2	1	0	-0.044763	-0.464913	7.040517
3	8	0	0.769349	-0.562494	7.577540
4	6	0	1.525659	-1.680963	7.188292
5	6	0	1.669965	-1.855388	5.692823
6	1	0	2.524200	-1.563117	7.627249
7	1	0	1.101606	-2.614541	7.591743
8	6	0	2.142993	-0.595279	4.998352
9	8	0	0.418462	-2.272196	5.179461
10	1	0	0.411899	-2.060912	4.236658
11	1	0	2.421507	-2.647359	5.521499
12	8	0	2.137087	-0.839023	3.598013
13	1	0	1.774691	-0.066900	3.148816

14	1	0	3.156868	-0.349560	5.345368
15	1	0	1.485577	0.247260	5.245027
16	1	0	-1.543855	4.026793	0.307604
17	8	0	-2.187674	4.381871	0.932160
18	6	0	-3.430939	3.801985	0.604026
19	6	0	-3.300267	2.314205	0.391331
20	1	0	-3.848970	4.258191	-0.308470
21	1	0	-4.119373	4.005961	1.429757
22	8	0	-2.368759	2.152996	-0.667180
23	1	0	-2.176898	1.212742	-0.764579
24	1	0	-2.896752	1.855057	1.311318
25	6	0	-4.618385	1.663705	0.058336
26	8	0	-4.369155	0.280363	-0.102521
27	1	0	-5.147035	-0.140385	-0.476791
28	1	0	-5.016442	2.104069	-0.868061
29	1	0	-5.338132	1.850688	0.869073
30	6	0	-0.874037	-0.022188	1.302102
31	6	0	-0.160434	1.102685	1.673391
32	6	0	-0.171412	1.494986	3.001869
33	6	0	-0.894666	0.746633	3.951277
34	6	0	-1.608353	-0.394298	3.539643
35	6	0	-1.596208	-0.776293	2.211739
36	1	0	0.381750	1.669790	0.923319
37	1	0	0.368374	2.376173	3.326801
38	7	0	-0.879288	1.120531	5.290572
39	1	0	-2.156327	-0.963202	4.279885
40	1	0	-2.146610	-1.647248	1.871454
41	8	0	-1.468118	0.376559	6.161532
42	8	0	-0.256683	2.177592	5.642573

----

09 Cpl Cl(-) * 2 GL

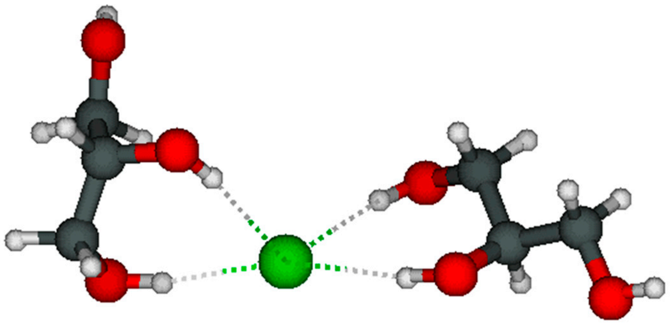

1	17	0	0.344899	-0.420987	-0.129403
2	1	0	-0.062032	-0.337535	2.052805
3	1	0	1.807927	0.157892	-1.730758
4	1	0	-1.504553	-1.429149	0.704637
5	1	0	0.150459	1.639398	-1.008697
6	8	0	-0.319394	-0.469979	2.981526
7	6	0	-1.725082	-0.485478	3.083504
8	6	0	-2.361536	-1.716499	2.460513
9	1	0	-1.957936	-0.461695	4.154302
10	1	0	-2.170571	0.412128	2.623683
11	8	0	-2.386317	-1.651283	1.047633
12	1	0	-1.787777	-2.600555	2.792051
13	6	0	-3.785292	-1.868687	2.933371
14	8	0	2.471736	0.583137	-2.300084

15	6	0	1.878964	1.659423	-2.995793
16	6	0	1.336600	2.764053	-2.103494
17	1	0	2.662248	2.077527	-3.638576
18	1	0	1.063351	1.313605	-3.648776
19	8	0	0.074042	2.448605	-1.544899
20	6	0	2.339848	3.167877	-1.045450
21	1	0	1.157260	3.637963	-2.746430
22	8	0	-4.297886	-3.099921	2.466332
23	1	0	-3.801459	-1.829847	4.032270
24	1	0	-4.378542	-1.022173	2.552909
25	1	0	-5.222347	-3.163834	2.718545
26	8	0	1.931262	4.399249	-0.481260
27	1	0	2.397641	2.381814	-0.278130
28	1	0	3.335341	3.254970	-1.507966
29	1	0	2.487655	4.590536	0.277679

----

10 Cpl 4-NO2-C6H4-N2(+) * Cl(-) * 4 GL

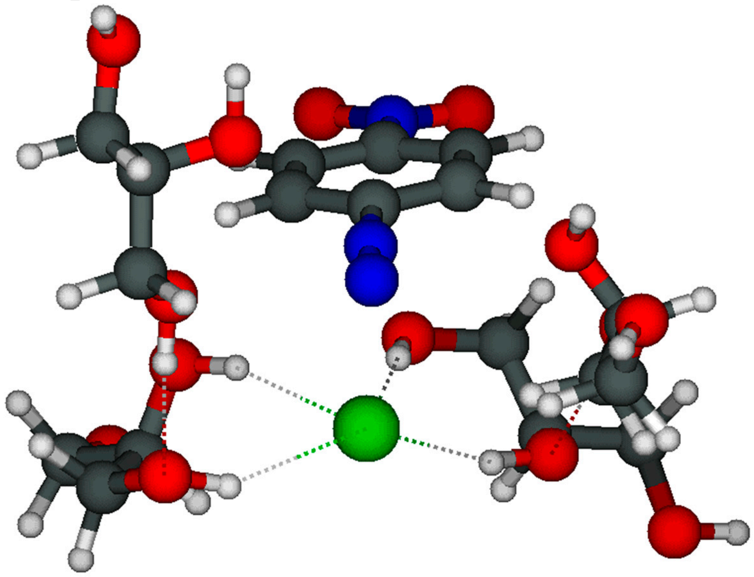

1	6	0	-0.109113	-0.051805	-0.067179
2	6	0	-0.062339	-0.020909	1.324011
3	6	0	1.191524	-0.016191	1.903520
4	6	0	2.302205	-0.056678	1.078049
5	6	0	2.238257	-0.042819	-0.306774
6	6	0	0.998778	-0.041914	-0.911971
7	1	0	-0.977681	-0.043816	1.906804
8	1	0	1.305732	-0.010282	2.980326
9	7	0	3.638763	-0.168052	1.711306
10	1	0	3.146034	-0.072134	-0.895507
11	1	0	0.859796	-0.086957	-1.988254
12	17	0	-0.337165	-3.330821	-0.534335
13	7	0	-1.366426	-0.198591	-0.637369
14	7	0	-2.377546	-0.357262	-1.034828
15	8	0	4.605318	-0.174100	0.981650
16	8	0	3.677027	-0.263451	2.918777
17	8	0	-0.496603	-0.679320	-3.455511
18	6	0	-1.372970	0.096347	-4.233202
19	6	0	-1.087566	1.549668	-3.958404
20	1	0	-1.239271	-0.095026	-5.308271
21	1	0	-2.426570	-0.113671	-3.982545
22	6	0	-1.905365	2.454433	-4.848902

23	8	0	-1.378916	1.813630	-2.594744
24	1	0	-0.016169	1.738821	-4.146563
25	1	0	-1.374471	2.773551	-2.493130
26	1	0	-0.563975	-1.631744	-3.663986
27	8	0	-1.615063	3.781534	-4.450347
28	1	0	-1.639889	2.283571	-5.901374
29	1	0	-2.973988	2.229758	-4.715845
30	1	0	-2.269616	4.378558	-4.821065
31	1	0	-0.083910	-4.298603	1.438354
32	8	0	-0.031972	-4.382823	2.408318
33	6	0	1.314127	-4.566967	2.828787
34	6	0	2.141564	-3.316707	2.594009
35	6	0	1.283734	-4.907463	4.297315
36	1	0	1.763752	-5.405997	2.274303
37	1	0	3.119584	-3.426926	3.074778
38	8	0	2.394528	-3.048612	1.233677
39	1	0	1.626994	-2.466090	3.078372
40	1	0	1.554965	-3.015191	0.742668
41	1	0	2.312569	-4.910242	4.684609
42	1	0	0.723662	-4.121231	4.829963
43	8	0	0.674271	-6.168909	4.463739
44	1	0	0.599909	-6.354170	5.403351
45	1	0	-0.479335	-3.717194	-2.806939
46	8	0	-0.378669	-3.421911	-3.728807
47	6	0	0.874066	-3.838998	-4.243167
48	6	0	1.973294	-3.870331	-3.194111
49	1	0	0.785023	-4.833630	-4.702663
50	1	0	1.142383	-3.124831	-5.033450
51	6	0	3.297181	-4.177162	-3.845406
52	8	0	2.081073	-2.635605	-2.515638
53	1	0	1.752074	-4.671977	-2.467921
54	1	0	1.384919	-2.622225	-1.838070
55	8	0	4.233595	-4.510980	-2.842611
56	1	0	3.167701	-5.005467	-4.557941
57	1	0	3.618885	-3.290193	-4.412700
58	1	0	5.097300	-4.618653	-3.248780
59	8	0	-1.542197	-2.139202	3.139671
60	6	0	-2.665052	-2.453000	2.347839
61	6	0	-3.670259	-1.337487	2.431476
62	1	0	-2.373264	-2.598067	1.292847
63	1	0	-3.146058	-3.383490	2.689132
64	6	0	-4.957565	-1.703070	1.731117
65	1	0	-3.888146	-1.129284	3.492594
66	8	0	-3.111693	-0.177973	1.829166
67	1	0	-3.819691	0.475435	1.768015
68	8	0	-5.797794	-0.566881	1.814065
69	1	0	-4.740160	-1.955323	0.681607
70	1	0	-5.414703	-2.577431	2.214523
71	1	0	-6.534395	-0.667017	1.206074
72	1	0	-0.905831	-2.867041	3.015265

----

11 TS_SN 4-NO2-C6H4-N2(+) * Cl(-) * 4 GL

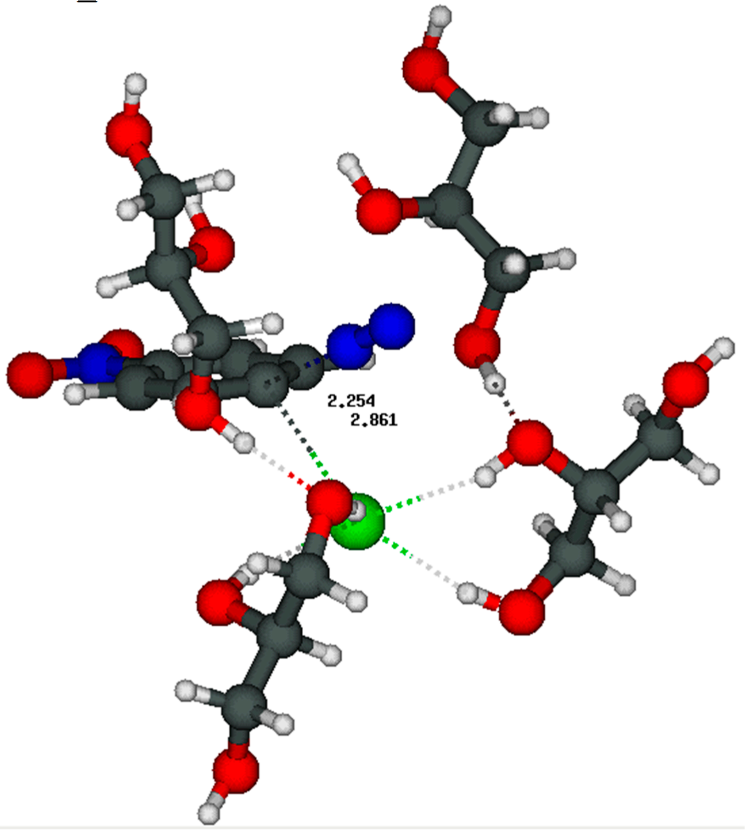

1	6	0	0.050340	0.037849	-0.020793
2	6	0	0.057505	0.008578	1.312113
3	6	0	1.396154	-0.004893	1.725264
4	6	0	2.409086	0.039066	0.775154
5	6	0	2.178492	0.102318	-0.592708
6	6	0	0.861731	0.119827	-1.074685
7	1	0	-0.826875	-0.004493	1.955609
8	1	0	1.626183	-0.049064	2.783546
9	7	0	3.805749	0.031955	1.245751
10	1	0	3.002319	0.149875	-1.295453
11	1	0	0.555450	0.212946	-2.122294
12	17	0	-1.669125	-2.180221	-0.577225
13	7	0	-1.941008	0.862007	-0.680332
14	7	0	-2.902486	1.286257	-0.984123
15	8	0	4.681397	0.072999	0.407475
16	8	0	3.994848	-0.013853	2.443026
17	8	0	-0.529311	0.520475	-3.695058
18	6	0	-1.016607	1.806471	-4.007596
19	6	0	-0.093970	2.851028	-3.435839
20	1	0	-1.079059	1.943633	-5.097982
21	1	0	-2.025426	1.956664	-3.593255
22	6	0	-0.419995	4.224923	-3.973000
23	8	0	-0.213665	2.836141	-2.024399
24	1	0	0.942434	2.597339	-3.723162
25	1	0	0.244296	3.620932	-1.699510
26	1	0	-1.279481	-0.104762	-3.634720
27	8	0	0.447819	5.131207	-3.317681
28	1	0	-0.274190	4.245913	-5.061848
29	1	0	-1.472504	4.461728	-3.755501
30	1	0	0.140876	6.029986	-3.460193
31	1	0	-3.823705	-1.559124	0.077843

32	8	0	-4.404880	-1.134226	0.730579
33	6	0	-5.226676	-2.115724	1.357650
34	6	0	-4.408798	-3.310319	1.812252
35	6	0	-5.914627	-1.458549	2.526362
36	1	0	-5.988649	-2.460784	0.640652
37	1	0	-5.046730	-3.975467	2.405137
38	8	0	-3.910972	-4.065424	0.731515
39	1	0	-3.591543	-2.960636	2.464558
40	1	0	-3.111866	-3.639354	0.381013
41	1	0	-6.496074	-2.221686	3.063681
42	1	0	-5.153675	-1.066094	3.220477
43	8	0	-6.747185	-0.425091	2.046504
44	1	0	-7.147140	0.022465	2.796570
45	1	0	-2.684393	-1.531424	-2.539900
46	8	0	-2.685437	-1.161069	-3.439861
47	6	0	-2.600352	-2.210233	-4.390013
48	6	0	-1.718099	-3.359643	-3.928731
49	1	0	-3.602206	-2.593058	-4.628679
50	1	0	-2.181238	-1.768223	-5.304114
51	6	0	-1.513584	-4.328720	-5.064646
52	8	0	-0.454911	-2.900973	-3.491390
53	1	0	-2.224044	-3.891939	-3.105444
54	1	0	-0.546276	-2.629012	-2.564738
55	8	0	-0.982267	-5.532010	-4.552229
56	1	0	-2.475623	-4.503369	-5.569413
57	1	0	-0.828838	-3.867543	-5.792914
58	1	0	-0.759384	-6.109851	-5.286326
59	8	0	-2.699182	0.130668	2.484616
60	6	0	-3.293888	1.395318	2.684501
61	6	0	-2.224511	2.433081	2.903737
62	1	0	-3.898514	1.682739	1.810522
63	1	0	-3.960168	1.376634	3.561470
64	6	0	-2.815429	3.740990	3.376046
65	1	0	-1.528714	2.061687	3.678097
66	8	0	-1.525062	2.622723	1.687104
67	1	0	-0.977106	3.408603	1.803429
68	8	0	-1.740498	4.657021	3.472993
69	1	0	-3.563055	4.087823	2.646919
70	1	0	-3.311527	3.600356	4.346297
71	1	0	-2.084983	5.550488	3.545493
72	1	0	-3.270119	-0.383503	1.880488

IRC from TS_SN 4-NO2-C6H4-N2(+) * Cl(-) * 4

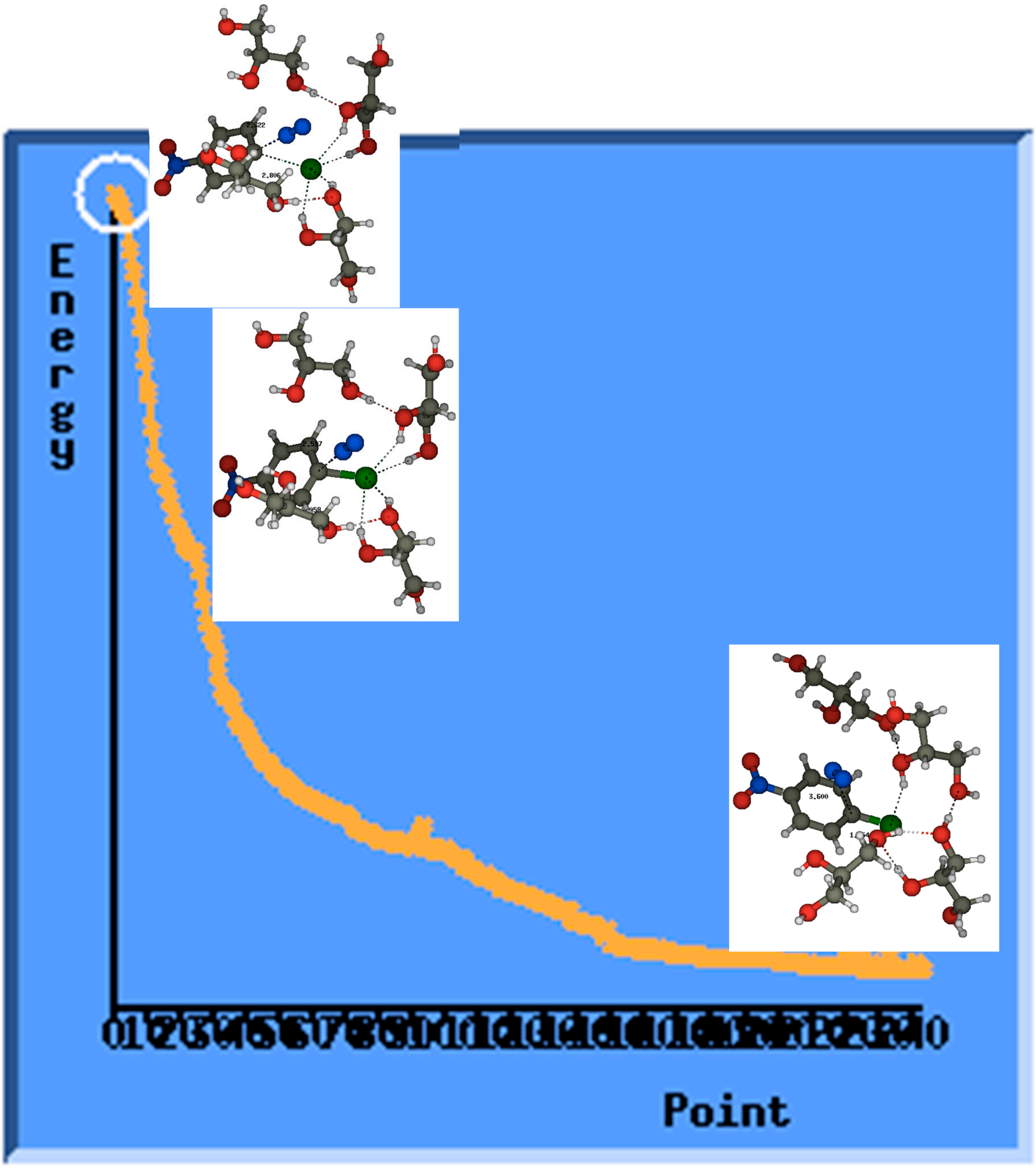

12 Cpl 4-NO2-C6H4-Cl * N2 * 4 GL

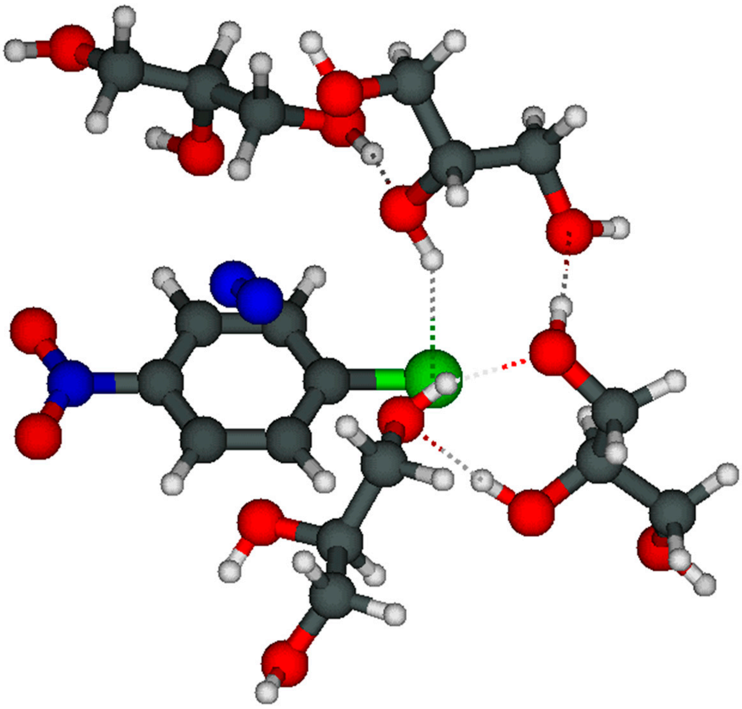

1	6	0	0.316014	0.560702	-1.627424
2	6	0	1.681891	0.363015	-1.768038
3	6	0	2.542052	1.399423	-1.449725
4	6	0	2.005853	2.597365	-1.003191
5	6	0	0.640061	2.791898	-0.857274
6	6	0	-0.220226	1.756748	-1.173852
7	1	0	2.079887	-0.591607	-2.097502
8	1	0	3.612835	1.251793	-1.535597
9	7	0	2.918009	3.689211	-0.648557
10	1	0	0.251814	3.729575	-0.479918
11	1	0	-1.288118	1.867102	-1.028250
12	17	0	-0.768326	-0.756254	-2.022608
13	7	0	1.093376	1.006600	1.888123
14	7	0	0.225304	0.995824	2.554908
15	8	0	2.433567	4.741098	-0.273927
16	8	0	4.114917	3.489574	-0.743046
17	8	0	-2.274699	0.524157	0.873773
18	6	0	-3.068106	1.346117	1.704500
19	6	0	-3.266837	2.690635	1.056635
20	1	0	-4.055517	0.886208	1.870966
21	1	0	-2.586403	1.487520	2.684353
22	6	0	-4.277519	3.514276	1.822326
23	8	0	-2.024206	3.366340	1.007886
24	1	0	-3.646650	2.536295	0.029642
25	1	0	-2.226869	4.290595	0.815210
26	1	0	-2.459947	-0.402552	1.115171
27	8	0	-4.318821	4.784510	1.198350
28	1	0	-5.259756	3.022664	1.796509
29	1	0	-3.956775	3.602993	2.871034
30	1	0	-4.757360	5.413083	1.777003
31	1	0	-0.113485	-1.860021	0.038338
32	8	0	0.612299	-2.021896	0.658311
33	6	0	0.192744	-3.000262	1.598232
34	6	0	-0.346305	-4.217872	0.882895
35	6	0	1.369623	-3.364549	2.464869

36	1	0	-0.598091	-2.573409	2.238924
37	1	0	-0.690826	-4.963928	1.610038
38	8	0	-1.418694	-3.772244	0.063100
39	1	0	0.445669	-4.663198	0.264219
40	1	0	-1.666344	-4.457621	-0.564322
41	1	0	1.102123	-4.241636	3.072289
42	1	0	2.213865	-3.648351	1.815668
43	8	0	1.695432	-2.258165	3.275142
44	1	0	2.485250	-2.466788	3.780817
45	1	0	-2.671660	-2.676655	0.764201
46	8	0	-3.316511	-2.004789	1.055711
47	6	0	-4.472628	-2.142730	0.244977
48	6	0	-4.273549	-1.585855	-1.154615
49	1	0	-4.763907	-3.200963	0.186765
50	1	0	-5.283689	-1.594731	0.739650
51	6	0	-5.443675	-1.947321	-2.034204
52	8	0	-4.150702	-0.180204	-1.149726
53	1	0	-3.363717	-2.049646	-1.582562
54	1	0	-3.378970	0.077634	-0.615859
55	8	0	-5.144789	-1.587260	-3.366632
56	1	0	-5.633791	-3.027364	-1.951888
57	1	0	-6.336349	-1.415646	-1.668950
58	1	0	-5.917625	-1.755228	-3.911638
59	8	0	3.076279	-2.489784	-0.550794
60	6	0	3.821315	-1.590826	0.234965
61	6	0	5.149033	-1.318128	-0.421596
62	1	0	3.276437	-0.641771	0.367283
63	1	0	4.010847	-2.001008	1.241671
64	6	0	6.012755	-0.440456	0.454233
65	1	0	5.669079	-2.279246	-0.580839
66	8	0	4.930646	-0.685694	-1.670582
67	1	0	5.792782	-0.374987	-1.972879
68	8	0	7.212067	-0.205820	-0.261227
69	1	0	5.482601	0.504585	0.650876
70	1	0	6.209711	-0.938103	1.414066
71	1	0	7.682231	0.532346	0.134226
72	1	0	2.156186	-2.434723	-0.238693

13 4-NO2-C6H4-Cl

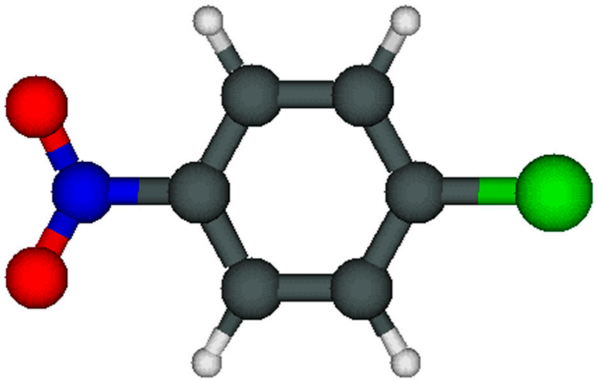

1	6	0	0.000000	0.000000	0.000000
2	6	0	0.000000	0.000000	1.386889
3	6	0	1.211242	0.000000	2.053706
4	6	0	2.388174	0.000000	1.315724
5	6	0	2.383318	0.000000	-0.073323
6	6	0	1.172473	0.000000	-0.740827

7	1	0	-0.936262	0.000000	1.931024
8	1	0	1.245861	0.000000	3.136872
9	17	0	3.910659	0.000000	2.155474
10	1	0	3.317443	0.000000	-0.622671
11	1	0	1.132593	0.000000	-1.822970
12	7	0	-1.284318	0.000000	-0.708557
13	8	0	-1.264645	-0.000002	-1.924754
14	8	0	-2.302584	-0.000002	-0.043328

----

r04 Cpl 4-NO2-C6H4 Rad. Cl(-) * 2 GL

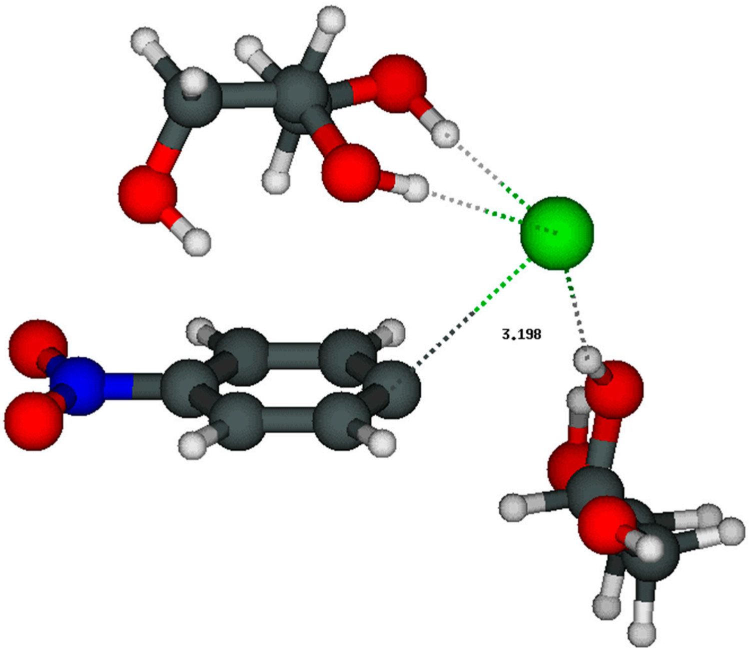

1	17	0	-0.147781	-0.347206	-0.155846
2	1	0	0.002086	-0.006368	4.795456
3	8	0	0.816625	0.024156	5.314019
4	6	0	1.623985	-1.027716	4.820968
5	6	0	1.662668	-1.034600	3.306833
6	1	0	2.630672	-0.890106	5.227946
7	1	0	1.248584	-2.001048	5.170244
8	6	0	2.323265	0.214107	2.752547
9	8	0	0.310066	-1.169896	2.901174
10	1	0	0.198419	-0.957581	1.956255
11	1	0	2.253106	-1.906940	2.976873
12	8	0	2.596973	0.142746	1.370735
13	1	0	1.761122	0.128280	0.869739
14	1	0	3.288332	0.346170	3.256235
15	1	0	1.702071	1.090180	2.990921
16	1	0	0.271352	4.304089	-1.957765
17	8	0	-0.339349	5.051257	-1.982332
18	6	0	-1.611452	4.507208	-2.258036
19	6	0	-1.883015	3.298442	-1.392772
20	1	0	-1.691018	4.215346	-3.318651
21	1	0	-2.354562	5.287033	-2.065686
22	8	0	-0.813270	2.406509	-1.631330
23	1	0	-0.853962	1.639530	-1.030090
24	1	0	-1.882235	3.611363	-0.333414
25	6	0	-3.220757	2.676335	-1.700608
26	8	0	-3.451338	1.660731	-0.743551

27	1	0	-4.197247	1.125580	-1.025644
28	1	0	-3.199292	2.264661	-2.720704
29	1	0	-4.003993	3.449083	-1.656907
30	6	0	-0.862042	2.253301	1.563262
31	6	0	0.208923	3.098154	1.710090
32	6	0	0.512567	3.511032	3.002635
33	6	0	-0.282650	3.055938	4.047788
34	6	0	-1.369645	2.208604	3.860442
35	6	0	-1.666026	1.783807	2.571579
36	1	0	0.801014	3.429533	0.861416
37	1	0	1.348284	4.170486	3.205741
38	7	0	0.051916	3.470301	5.413942
39	1	0	-1.957961	1.887300	4.712854
40	1	0	-2.492371	1.105988	2.380998
41	8	0	-0.705499	3.151288	6.311370
42	8	0	1.073218	4.110063	5.584609

r05 TS_CX

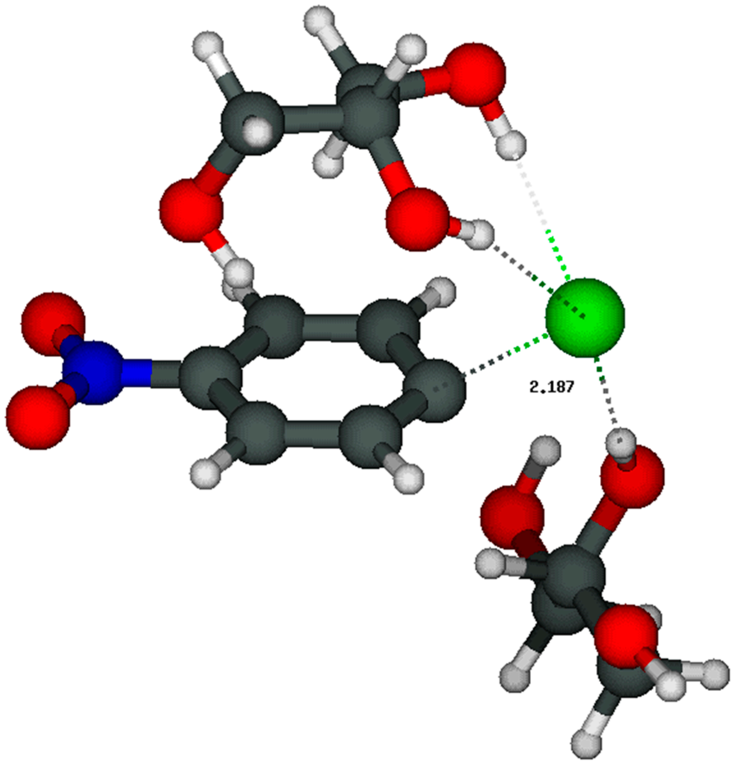

1	17	0	0.399194	-0.821515	-0.372266
2	1	0	-0.397812	0.499149	4.046133
3	8	0	0.222596	0.567592	4.784101
4	6	0	0.801510	-0.712085	4.941499
5	6	0	1.224936	-1.305649	3.615325
6	1	0	1.669639	-0.602499	5.599525
7	1	0	0.096588	-1.403041	5.427485
8	6	0	2.290240	-0.470360	2.931627
9	8	0	0.033859	-1.411874	2.849014
10	1	0	0.233045	-1.462264	1.902609
11	1	0	1.646653	-2.308228	3.800637
12	8	0	2.819990	-1.097946	1.780241
13	1	0	2.233279	-0.936799	1.026228
14	1	0	3.121647	-0.334564	3.633397
15	1	0	1.891737	0.525411	2.689636

16	1	0	1.593989	3.040598	-3.042732
17	8	0	1.085933	3.840054	-3.230326
18	6	0	-0.155641	3.402919	-3.737348
19	6	0	-0.700662	2.257869	-2.916639
20	1	0	-0.058244	3.078669	-4.787032
21	1	0	-0.844740	4.252496	-3.709958
22	8	0	0.290957	1.245399	-2.954692
23	1	0	0.142088	0.630666	-2.220216
24	1	0	-0.839265	2.594292	-1.875070
25	6	0	-2.020969	1.759438	-3.445969
26	8	0	-2.491769	0.764903	-2.557436
27	1	0	-3.260461	0.337729	-2.943458
28	1	0	-1.871500	1.349929	-4.456411
29	1	0	-2.728827	2.599613	-3.516006
30	6	0	0.026891	1.237070	0.266365
31	6	0	1.198713	1.975497	0.412991
32	6	0	1.261441	2.928804	1.406600
33	6	0	0.173376	3.088475	2.277525
34	6	0	-0.976958	2.293316	2.152090
35	6	0	-1.034910	1.337174	1.161630
36	1	0	2.033571	1.836044	-0.270228
37	1	0	2.126999	3.573029	1.512420
38	7	0	0.235365	4.070547	3.303169
39	1	0	-1.814024	2.460018	2.821773
40	1	0	-1.911605	0.703026	1.058304
41	8	0	-0.724975	4.192486	4.071987
42	8	0	1.248597	4.772255	3.399284

----

r06 Cpl 4-NO2-C6H4-Cl(*-) * 2 GL

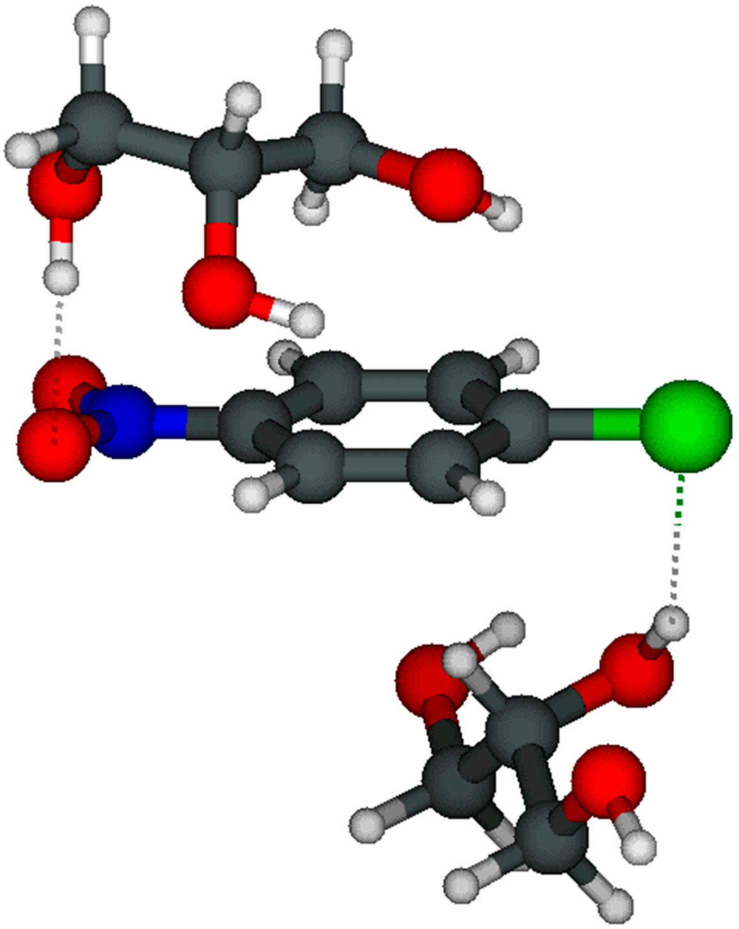

1	17	0	1.948787	0.394126	-2.035599
2	1	0	-1.789875	0.151450	3.677585
3	8	0	-1.258077	0.744243	4.246299
4	6	0	-0.265737	0.037118	4.944824
5	6	0	0.770661	-0.625274	4.064229
6	1	0	0.242798	0.759861	5.594343
7	1	0	-0.700795	-0.736430	5.596492
8	6	0	1.352367	0.319565	3.038368
9	8	0	0.171583	-1.741296	3.428995
10	1	0	0.738645	-1.975218	2.683796
11	1	0	1.590936	-0.970330	4.719890
12	8	0	2.292026	-0.422294	2.273474
13	1	0	2.374907	-0.020709	1.404102
14	1	0	1.834148	1.166851	3.548003
15	1	0	0.550578	0.705029	2.395871
16	1	0	-2.009827	2.509539	-3.874703
17	8	0	-2.889182	2.165671	-3.677168
18	6	0	-2.915307	0.835798	-4.145224
19	6	0	-1.681861	0.076221	-3.719539
20	1	0	-2.988516	0.808719	-5.245176
21	1	0	-3.811150	0.358694	-3.736336
22	8	0	-0.579279	0.794529	-4.253309
23	1	0	0.233540	0.437824	-3.872997
24	1	0	-1.618352	0.072812	-2.617664
25	6	0	-1.701693	-1.347581	-4.215060
26	8	0	-0.527917	-1.979032	-3.742574
27	1	0	-0.447162	-2.842834	-4.154471
28	1	0	-1.734437	-1.346480	-5.315075
29	1	0	-2.606111	-1.851777	-3.842708
30	6	0	0.544546	0.520238	-0.980081
31	6	0	-0.100672	1.744697	-0.844935
32	6	0	-1.207234	1.843069	-0.023645
33	6	0	-1.660534	0.714711	0.684788
34	6	0	-1.000672	-0.517731	0.523502
35	6	0	0.095557	-0.612548	-0.309750
36	1	0	0.259816	2.612093	-1.389107
37	1	0	-1.725553	2.786058	0.099141
38	7	0	-2.732822	0.820995	1.561354
39	1	0	-1.354121	-1.377697	1.077538
40	1	0	0.607447	-1.561164	-0.440320
41	8	0	-3.033282	-0.186560	2.307043
42	8	0	-3.371098	1.918961	1.656219

14 Cpl Br(-) * 2 GL

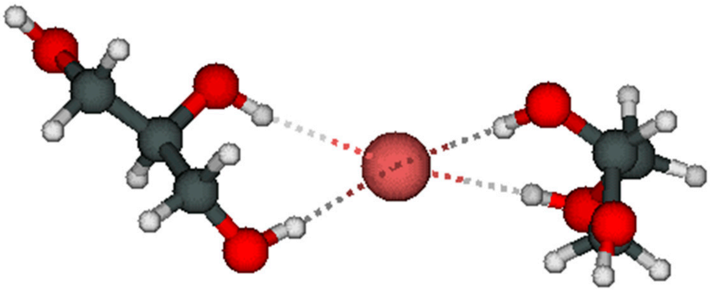

1	35	0	0.000000	0.000000	0.000000

2	1	0	0.000000	0.000000	2.392833
3	1	0	1.654393	0.000000	-1.748084
4	1	0	-2.016715	-0.081116	1.380764
5	1	0	0.973594	2.056101	-0.817006
6	8	0	-0.132811	-0.160091	3.342420
7	6	0	-1.286550	0.518751	3.785463
8	6	0	-2.576308	-0.098215	3.273950
9	1	0	-1.272137	0.471742	4.880352
10	1	0	-1.263109	1.581420	3.494026
11	8	0	-2.791546	0.180667	1.903425
12	1	0	-2.522444	-1.189280	3.437424
13	6	0	-3.759296	0.441538	4.036953
14	8	0	2.435559	0.107796	-2.315737
15	6	0	2.408511	1.382135	-2.924571
16	6	0	2.509239	2.543842	-1.950211
17	1	0	3.268109	1.414704	-3.603699
18	1	0	1.500212	1.517142	-3.530606
19	8	0	1.277578	2.835317	-1.314072
20	6	0	3.627456	2.336909	-0.952645
21	1	0	2.746321	3.440831	-2.540226
22	8	0	-4.919030	-0.269389	3.654115
23	1	0	-3.568751	0.329856	5.114305
24	1	0	-3.862281	1.516021	3.818226
25	1	0	-5.679151	0.124633	4.089308
26	8	0	3.886991	3.566452	-0.303655
27	1	0	3.332763	1.562498	-0.229004
28	1	0	4.520331	1.972077	-1.483817
29	1	0	4.508319	3.412880	0.412251

----

15 Cpl 4-NO2-C6H4-N2(+) * Br(-) * 4 GL

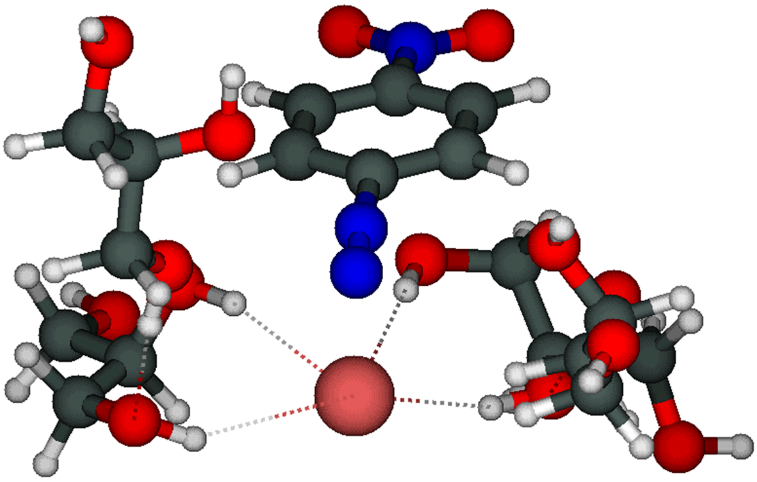

1	6	0	-0.044638	-0.064357	-0.017442
2	6	0	-0.016578	-0.031489	1.374740
3	6	0	1.229237	-0.045909	1.970871
4	6	0	2.350671	-0.109040	1.160959
5	6	0	2.306221	-0.099421	-0.224344
6	6	0	1.075380	-0.075044	-0.846657
7	1	0	-0.939990	-0.036573	1.945272
8	1	0	1.328885	-0.037055	3.049088
9	7	0	3.676329	-0.239408	1.813468
10	1	0	3.219643	-0.151064	-0.802591
11	1	0	0.951704	-0.115378	-1.925414
12	35	0	-0.346372	-3.537011	-0.633929

13	7	0	-1.294378	-0.185945	-0.609886
14	7	0	-2.296131	-0.325081	-1.037250
15	8	0	4.651339	-0.282714	1.096557
16	8	0	3.697671	-0.310817	3.023060
17	8	0	-0.379273	-0.641246	-3.414241
18	6	0	-1.155455	0.185956	-4.243879
19	6	0	-0.856595	1.620754	-3.895009
20	1	0	-0.925376	0.024944	-5.307191
21	1	0	-2.233265	-0.000785	-4.100704
22	6	0	-1.581605	2.578536	-4.810056
23	8	0	-1.243813	1.844777	-2.548189
24	1	0	0.230425	1.787384	-3.994757
25	1	0	-1.228463	2.800566	-2.413316
26	1	0	-0.368071	-1.568009	-3.722257
27	8	0	-1.287141	3.882198	-4.342827
28	1	0	-1.242920	2.436435	-5.845668
29	1	0	-2.662640	2.380369	-4.764318
30	1	0	-1.896149	4.510376	-4.738800
31	1	0	-0.063227	-4.403203	1.537591
32	8	0	-0.026504	-4.411035	2.511281
33	6	0	1.309901	-4.589408	2.966058
34	6	0	2.141042	-3.342648	2.727836
35	6	0	1.245524	-4.903440	4.439425
36	1	0	1.770056	-5.438169	2.436171
37	1	0	3.111824	-3.447229	3.224056
38	8	0	2.415341	-3.091282	1.368116
39	1	0	1.619837	-2.485979	3.193491
40	1	0	1.586785	-3.076825	0.858886
41	1	0	2.264629	-4.894821	4.851590
42	1	0	0.669892	-4.109853	4.943712
43	8	0	0.636789	-6.164040	4.613532
44	1	0	0.536786	-6.331321	5.554138
45	1	0	-0.190312	-3.764189	-3.129016
46	8	0	-0.025986	-3.318324	-3.976511
47	6	0	1.292883	-3.581964	-4.423062
48	6	0	2.304844	-3.664056	-3.292277
49	1	0	1.320114	-4.516394	-5.001128
50	1	0	1.562726	-2.758658	-5.097877
51	6	0	3.695022	-3.801676	-3.858565
52	8	0	2.265940	-2.514467	-2.471733
53	1	0	2.089939	-4.560274	-2.683995
54	1	0	1.519967	-2.621034	-1.858158
55	8	0	4.574521	-4.192440	-2.825529
56	1	0	3.683886	-4.543723	-4.670781
57	1	0	3.990347	-2.832748	-4.289747
58	1	0	5.473137	-4.186781	-3.164635
59	8	0	-1.524210	-2.133560	3.153354
60	6	0	-2.657640	-2.432668	2.371420
61	6	0	-3.641158	-1.297871	2.453409
62	1	0	-2.377808	-2.589902	1.314455
63	1	0	-3.153065	-3.351903	2.722498
64	6	0	-4.943119	-1.647170	1.771846
65	1	0	-3.843090	-1.074005	3.514509
66	8	0	-3.067582	-0.155716	1.832318
67	1	0	-3.766362	0.507047	1.764830
68	8	0	-5.757618	-0.491630	1.843283
69	1	0	-4.743020	-1.923114	0.724880
70	1	0	-5.413969	-2.502605	2.275496
71	1	0	-6.500527	-0.584526	1.241870

72	1	0	-0.903193	-2.876500	3.039820

16 TS_SN 4-NO2-C6H4-N2(+) * Br(-) * 4 GL

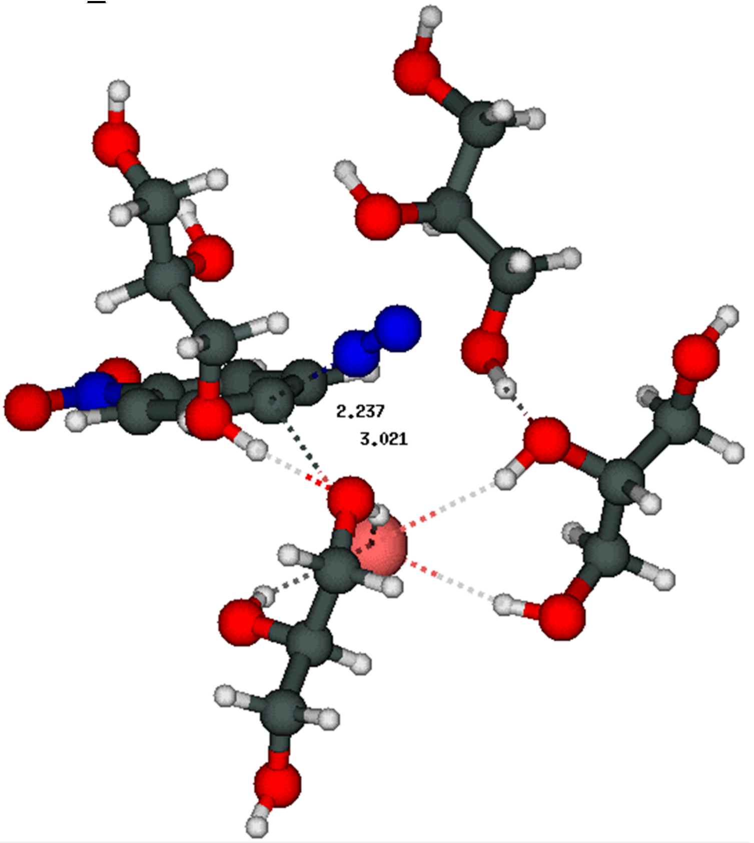

1	6	0	0.011920	0.004161	-0.007864
2	6	0	0.014065	0.007448	1.326568
3	6	0	1.349474	-0.003979	1.749566
4	6	0	2.369181	0.016074	0.806249
5	6	0	2.148599	0.051664	-0.564612
6	6	0	0.836045	0.065063	-1.055104
7	1	0	-0.873076	0.011291	1.965573
8	1	0	1.572093	-0.026398	2.810083
9	7	0	3.762302	0.012678	1.286976
10	1	0	2.977969	0.079068	-1.261866
11	1	0	0.535744	0.132479	-2.105260
12	35	0	-1.708596	-2.404899	-0.608006
13	7	0	-1.961158	0.815969	-0.680726
14	7	0	-2.917839	1.260351	-0.970809
15	8	0	4.644144	0.033240	0.454339
16	8	0	3.942827	-0.009567	2.486313
17	8	0	-0.584948	0.468403	-3.664035
18	6	0	-1.020947	1.770654	-3.984772
19	6	0	-0.062121	2.781785	-3.412318
20	1	0	-1.072234	1.905374	-5.076167
21	1	0	-2.025725	1.960879	-3.576613
22	6	0	-0.322726	4.162520	-3.967759
23	8	0	-0.198517	2.788429	-2.002341
24	1	0	0.966019	2.481310	-3.684189
25	1	0	0.286523	3.558538	-1.681470
26	1	0	-1.358120	-0.129066	-3.629893
27	8	0	0.573778	5.038074	-3.309152
28	1	0	-0.161276	4.166142	-5.054568

29	1	0	-1.367068	4.445749	-3.767463
30	1	0	0.304050	5.947391	-3.459688
31	1	0	-3.991310	-1.558634	0.043342
32	8	0	-4.513457	-1.069527	0.700005
33	6	0	-5.439281	-1.944409	1.339286
34	6	0	-4.762055	-3.222853	1.794710
35	6	0	-6.033764	-1.205400	2.510611
36	1	0	-6.242801	-2.200980	0.630644
37	1	0	-5.472383	-3.814588	2.383050
38	8	0	-4.347690	-4.023008	0.710461
39	1	0	-3.913050	-2.968329	2.450050
40	1	0	-3.485217	-3.713387	0.390843
41	1	0	-6.691477	-1.893987	3.060616
42	1	0	-5.223588	-0.898372	3.192157
43	8	0	-6.748781	-0.086374	2.033442
44	1	0	-7.068680	0.419299	2.785087
45	1	0	-2.874843	-1.518068	-2.611760
46	8	0	-2.855269	-1.076876	-3.478008
47	6	0	-2.914251	-2.047250	-4.510151
48	6	0	-2.085209	-3.283926	-4.203752
49	1	0	-3.955240	-2.341619	-4.701722
50	1	0	-2.528383	-1.558772	-5.414724
51	6	0	-2.037712	-4.178837	-5.415173
52	8	0	-0.762243	-2.942215	-3.842088
53	1	0	-2.563232	-3.840994	-3.379911
54	1	0	-0.758626	-2.720742	-2.898005
55	8	0	-1.551784	-5.446947	-5.030153
56	1	0	-3.046649	-4.255118	-5.847504
57	1	0	-1.382787	-3.710620	-6.166126
58	1	0	-1.450099	-5.991428	-5.814889
59	8	0	-2.767575	0.138442	2.458215
60	6	0	-3.354001	1.406364	2.663259
61	6	0	-2.277017	2.436346	2.881486
62	1	0	-3.961268	1.699604	1.793096
63	1	0	-4.016920	1.389465	3.543097
64	6	0	-2.857124	3.748371	3.355510
65	1	0	-1.582281	2.060065	3.654529
66	8	0	-1.578934	2.620858	1.663312
67	1	0	-1.023203	3.401370	1.778801
68	8	0	-1.774838	4.655702	3.452491
69	1	0	-3.602566	4.101930	2.627405
70	1	0	-3.353476	3.610680	4.326074
71	1	0	-2.112245	5.551688	3.527164
72	1	0	-3.339682	-0.367950	1.849197

----

17 Cpl 4-NO2-C6H4-Br * N2 * 4 GL

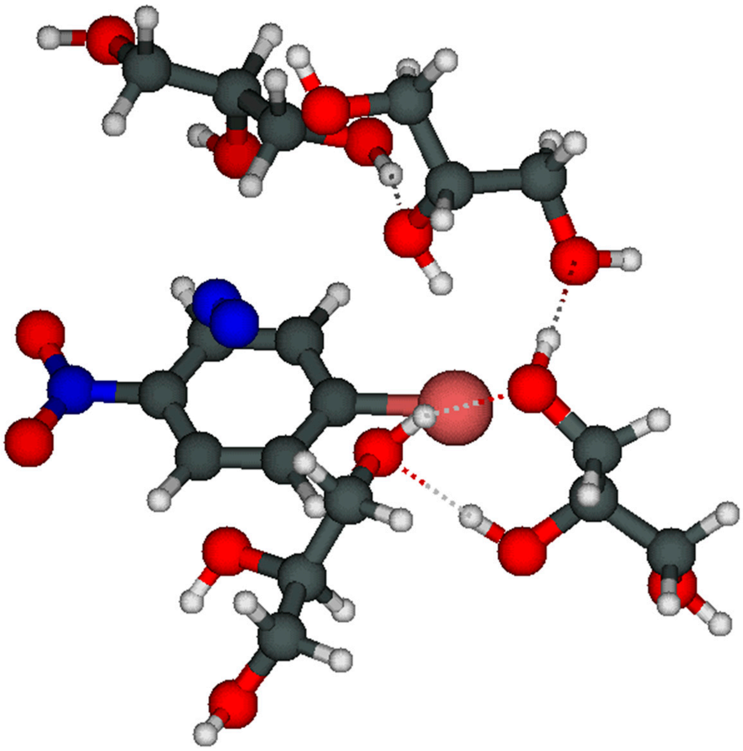

1	6	0	0.407647	0.587730	-1.594175
2	6	0	1.770647	0.377294	-1.753411
3	6	0	2.649695	1.395230	-1.424751
4	6	0	2.135203	2.591674	-0.950199
5	6	0	0.774571	2.799644	-0.780025
6	6	0	-0.104207	1.782376	-1.106494
7	1	0	2.156667	-0.574454	-2.103906
8	1	0	3.717460	1.234728	-1.523752
9	7	0	3.065852	3.665136	-0.586160
10	1	0	0.403142	3.733394	-0.376940
11	1	0	-1.167493	1.910384	-0.939321
12	35	0	-0.793355	-0.814616	-2.044889
13	7	0	1.192412	1.100233	1.940047
14	7	0	0.331282	1.117068	2.615739
15	8	0	2.599970	4.711367	-0.174142
16	8	0	4.258390	3.456747	-0.711417
17	8	0	-2.211110	0.660084	0.983736
18	6	0	-2.975777	1.541028	1.780730
19	6	0	-3.129671	2.864023	1.079394
20	1	0	-3.977464	1.120418	1.965477
21	1	0	-2.489147	1.708308	2.753921
22	6	0	-4.128330	3.742818	1.798230
23	8	0	-1.868330	3.503907	1.024682
24	1	0	-3.498959	2.680588	0.053230
25	1	0	-2.043429	4.425621	0.795677
26	1	0	-2.410581	-0.247282	1.281476
27	8	0	-4.123247	4.990734	1.129614
28	1	0	-5.123612	3.278200	1.772679
29	1	0	-3.822344	3.859725	2.848560
30	1	0	-4.548584	5.652503	1.680468
31	1	0	-0.170969	-1.839473	0.235675
32	8	0	0.574451	-1.954873	0.843598
33	6	0	0.200254	-2.899702	1.835802

34	6	0	-0.390796	-4.131099	1.188173
35	6	0	1.418885	-3.256965	2.645714
36	1	0	-0.548518	-2.444238	2.506898
37	1	0	-0.715947	-4.845190	1.955059
38	8	0	-1.490169	-3.692207	0.401154
39	1	0	0.364839	-4.611353	0.550599
40	1	0	-1.774746	-4.390572	-0.195504
41	1	0	1.169541	-4.105844	3.299251
42	1	0	2.218188	-3.582472	1.960383
43	8	0	1.813254	-2.130221	3.395011
44	1	0	2.618413	-2.343376	3.873991
45	1	0	-2.678906	-2.526596	1.097228
46	8	0	-3.306729	-1.827312	1.359488
47	6	0	-4.482360	-2.004519	0.584819
48	6	0	-4.316109	-1.516414	-0.844525
49	1	0	-4.773017	-3.064544	0.585550
50	1	0	-5.282423	-1.433294	1.070971
51	6	0	-5.503565	-1.917827	-1.682431
52	8	0	-4.191191	-0.112372	-0.909169
53	1	0	-3.415583	-2.001142	-1.269428
54	1	0	-3.385151	0.163890	-0.438965
55	8	0	-5.221276	-1.640988	-3.038484
56	1	0	-5.703055	-2.988977	-1.533408
57	1	0	-6.385288	-1.356082	-1.336385
58	1	0	-6.005113	-1.828188	-3.561045
59	8	0	3.012415	-2.448039	-0.421205
60	6	0	3.792373	-1.550124	0.331249
61	6	0	5.142295	-1.382340	-0.314551
62	1	0	3.299115	-0.566905	0.404225
63	1	0	3.941922	-1.916046	1.361338
64	6	0	6.045065	-0.522450	0.539326
65	1	0	5.606777	-2.377912	-0.426970
66	8	0	4.978804	-0.789158	-1.591183
67	1	0	5.861983	-0.543616	-1.892510
68	8	0	7.264973	-0.387196	-0.166850
69	1	0	5.569711	0.459207	0.691812
70	1	0	6.200360	-0.992759	1.520336
71	1	0	7.775620	0.333097	0.210807
72	1	0	2.100620	-2.371266	-0.089643

----

18 4-NO2-C6H4-Br

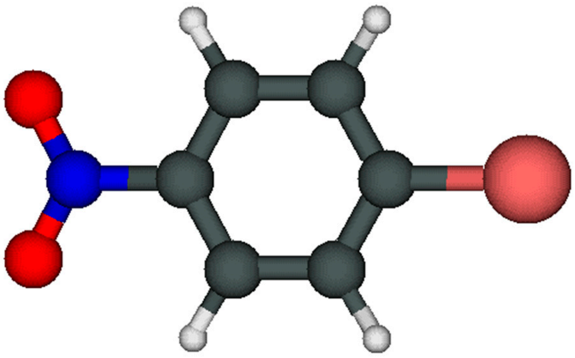

1	6	0	-0.000000	0.000000	0.000000
2	6	0	0.000000	0.000000	1.386614
3	6	0	1.212197	-0.000000	2.053284
4	6	0	2.390894	-0.000000	1.317044
5	6	0	2.383299	-0.000000	-0.072673
6	6	0	1.172014	0.000000	-0.740999
7	1	0	-0.936120	0.000000	1.931002
8	1	0	1.240688	-0.000000	3.136651
9	35	0	4.048031	-0.000000	2.229893
10	1	0	3.314224	-0.000000	-0.627539
11	1	0	1.131892	0.000000	-1.823159
12	7	0	-1.285581	0.000000	-0.708173
13	8	0	-1.266548	0.000000	-1.924207
14	8	0	-2.303244	0.000000	-0.042244

r07 Cpl 4-NO2-C6H4 Rad. Br(-) * 2 GL

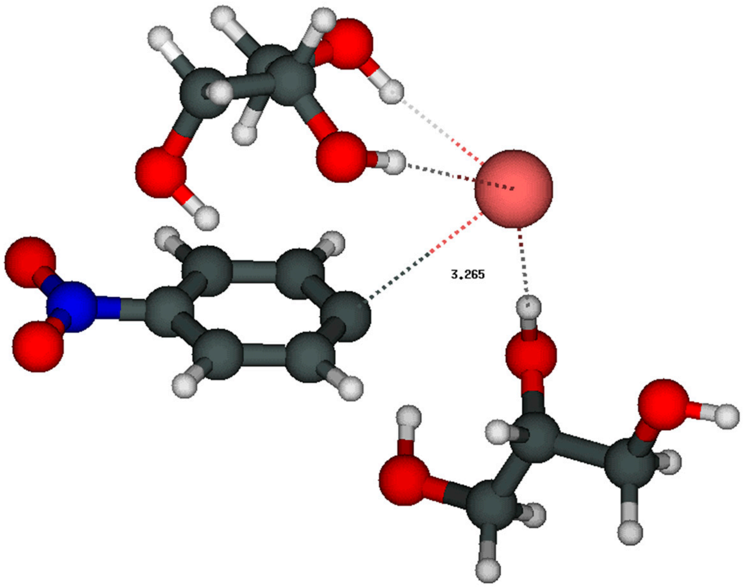

1	35	0	0.212747	-0.090340	-0.221186
2	1	0	-0.148585	0.006014	4.935951
3	8	0	0.603412	-0.052250	5.539619
4	6	0	1.393943	-1.127708	5.071175
5	6	0	1.613221	-1.046637	3.575065
6	1	0	2.351927	-1.087044	5.598963
7	1	0	0.918618	-2.091114	5.307875
8	6	0	2.406995	0.186731	3.186429
9	8	0	0.312642	-1.066074	3.007920

10	1	0	0.331317	-0.826653	2.064584
11	1	0	2.185383	-1.935267	3.257471
12	8	0	2.831486	0.182976	1.840683
13	1	0	2.063462	0.270095	1.249231
14	1	0	3.315515	0.219863	3.799338
15	1	0	1.818185	1.087285	3.415448
16	1	0	-1.357048	4.442106	0.022081
17	8	0	-2.270077	4.723964	0.165956
18	6	0	-3.023526	4.123882	-0.861616
19	6	0	-2.598521	2.688920	-1.093807
20	1	0	-2.911352	4.682608	-1.806554
21	1	0	-4.077936	4.165565	-0.571553
22	8	0	-1.205445	2.719890	-1.347334
23	1	0	-0.807695	1.878084	-1.056607
24	1	0	-2.793036	2.098139	-0.181355
25	6	0	-3.368756	2.087391	-2.243648
26	8	0	-3.064229	0.710729	-2.321296
27	1	0	-3.457636	0.348604	-3.119093
28	1	0	-3.083542	2.610332	-3.169178
29	1	0	-4.446068	2.247345	-2.080032
30	6	0	-0.701227	2.314914	1.788894
31	6	0	0.411414	3.091856	1.993431
32	6	0	0.678019	3.484856	3.299884
33	6	0	-0.189921	3.075437	4.305594
34	6	0	-1.314252	2.293494	4.063374
35	6	0	-1.576366	1.893124	2.759388
36	1	0	1.069545	3.382047	1.178600
37	1	0	1.542612	4.090921	3.544462
38	7	0	0.108212	3.459722	5.688258
39	1	0	-1.958962	2.004190	4.885952
40	1	0	-2.434928	1.267863	2.531933
41	8	0	-0.699813	3.169850	6.550869
42	8	0	1.151641	4.047275	5.907437

----

r08 TS_CX

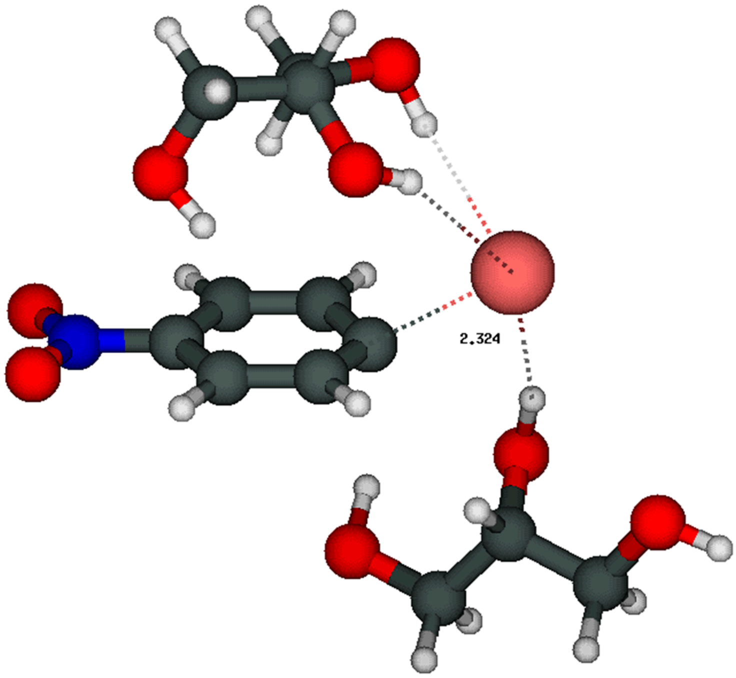

1	35	0	0.018002	0.007169	-0.007599
2	1	0	0.004553	0.007702	4.880474
3	8	0	0.740913	0.004499	5.506479
4	6	0	1.527445	-1.129219	5.200724
5	6	0	1.772944	-1.262858	3.713512
6	1	0	2.479724	-1.021390	5.730138
7	1	0	1.044517	-2.049331	5.561915
8	6	0	2.540974	-0.081448	3.154317
9	8	0	0.487076	-1.401896	3.126980
10	1	0	0.521949	-1.223947	2.176326
11	1	0	2.370675	-2.173543	3.538807
12	8	0	2.898395	-0.260037	1.797245
13	1	0	2.155429	-0.011103	1.227802
14	1	0	3.474014	0.022667	3.720630
15	1	0	1.960922	0.841875	3.290621
16	1	0	-1.386327	4.297483	0.333075
17	8	0	-2.296194	4.565386	0.518016
18	6	0	-3.066374	4.138943	-0.583261
19	6	0	-2.639926	2.766565	-1.053138
20	1	0	-2.973552	4.846278	-1.424784
21	1	0	-4.115499	4.125175	-0.271221
22	8	0	-1.270254	2.873551	-1.410233
23	1	0	-0.837339	2.028959	-1.218317
24	1	0	-2.744352	2.053370	-0.218241
25	6	0	-3.474360	2.289954	-2.215392
26	8	0	-3.091043	0.962864	-2.514778
27	1	0	-3.525524	0.686955	-3.325598
28	1	0	-3.303376	2.955919	-3.074773
29	1	0	-4.540251	2.345486	-1.945806
30	6	0	-0.515260	1.727942	1.461148
31	6	0	0.525096	2.629538	1.672754
32	6	0	0.629892	3.259114	2.896331
33	6	0	-0.276306	2.933501	3.914293
34	6	0	-1.283292	1.978396	3.708890
35	6	0	-1.378227	1.348666	2.486205
36	1	0	1.220891	2.872821	0.871999

37	1	0	1.384878	4.016222	3.076368
38	7	0	-0.173459	3.580118	5.179130
39	1	0	-1.987466	1.767906	4.506968
40	1	0	-2.147045	0.598440	2.315668
41	8	0	-0.967457	3.269608	6.072777
42	8	0	0.708361	4.428876	5.346423

----

r09 Cpl 4-NO2-C6H4-Br(*-) * 2 GL

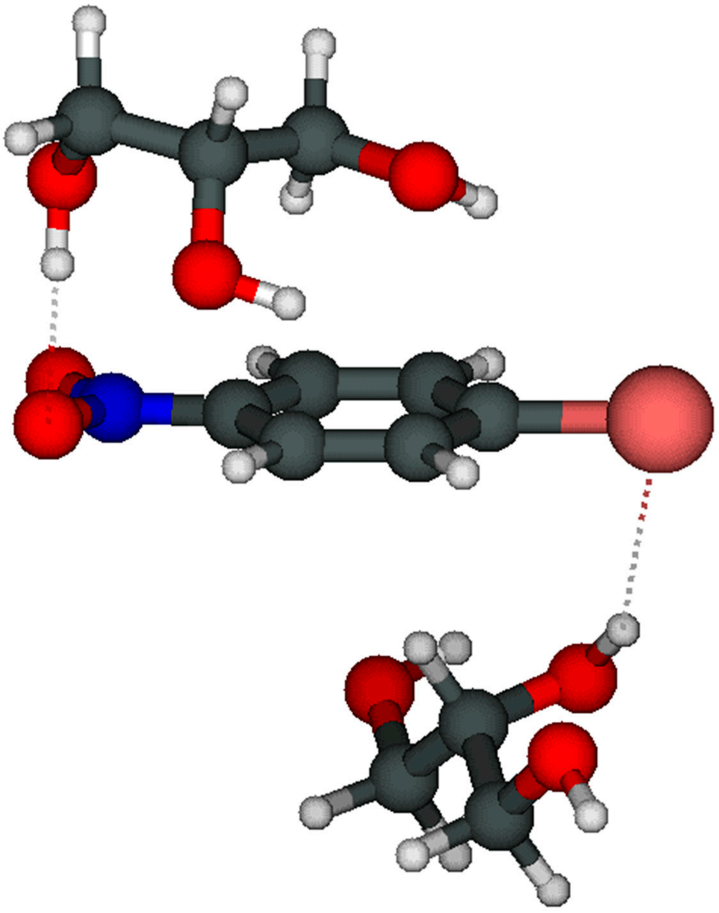

1	35	0	0.123405	0.076216	-0.160685
2	1	0	-0.014968	0.026483	6.839032
3	8	0	0.946052	-0.000849	7.023268
4	6	0	1.410822	-1.322918	7.111613
5	6	0	1.267302	-2.122038	5.835761
6	1	0	2.475216	-1.272180	7.371151
7	1	0	0.905356	-1.877407	7.917529
8	6	0	1.845324	-1.411850	4.633881
9	8	0	-0.107735	-2.398654	5.634029
10	1	0	-0.205226	-2.662056	4.710522
11	1	0	1.819167	-3.069735	5.973995
12	8	0	1.631735	-2.256219	3.511817
13	1	0	1.627063	-1.722258	2.712670
14	1	0	2.917342	-1.225902	4.794904
15	1	0	1.337161	-0.449088	4.494796
16	1	0	-2.194169	4.410897	0.664132
17	8	0	-2.858022	4.494216	1.359013
18	6	0	-3.889778	3.587778	1.039343
19	6	0	-3.338454	2.231014	0.673022
20	1	0	-4.496623	3.964771	0.199362

21	1	0	-4.540732	3.505403	1.914961
22	8	0	-2.475576	2.446888	-0.433953
23	1	0	-1.981760	1.633596	-0.600613
24	1	0	-2.754403	1.834757	1.521260
25	6	0	-4.434370	1.253540	0.331204
26	8	0	-3.820573	0.015709	0.029607
27	1	0	-4.477557	-0.580270	-0.338376
28	1	0	-5.000974	1.634448	-0.531827
29	1	0	-5.123386	1.162391	1.184396
30	6	0	-0.271473	0.606545	1.628560
31	6	0	0.117960	1.867382	2.072171
32	6	0	-0.173246	2.256598	3.365308
33	6	0	-0.847782	1.375927	4.231433
34	6	0	-1.245654	0.110589	3.761545
35	6	0	-0.959065	-0.268430	2.465332
36	1	0	0.643432	2.543709	1.404924
37	1	0	0.122914	3.232136	3.730623
38	7	0	-1.093898	1.741439	5.548698
39	1	0	-1.752267	-0.565394	4.438376
40	1	0	-1.263238	-1.246310	2.104100
41	8	0	-1.605782	0.875767	6.354659
42	8	0	-0.771544	2.903798	5.957349

19 Cpl I(-) * 2 GL

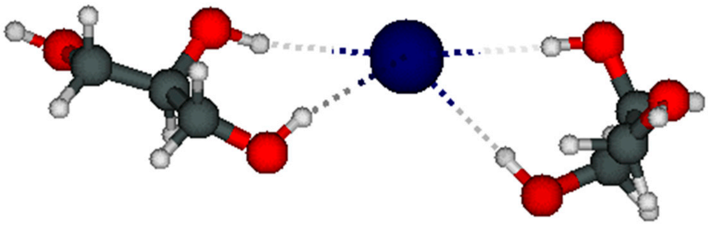

1	53	0	0.136430	-0.653893	-0.358380
2	1	0	2.131566	-0.547708	1.419163
3	8	0	2.875601	-0.265661	1.975802
4	6	0	4.087187	-0.765783	1.448343
5	6	0	4.452153	-0.203631	0.085526
6	1	0	4.869001	-0.491715	2.165748
7	1	0	4.069944	-1.863157	1.376781
8	6	0	4.339564	1.304131	0.053419
9	8	0	3.723878	-0.812732	-0.967404
10	1	0	2.780223	-0.604497	-0.872870
11	1	0	5.502164	-0.468694	-0.103396
12	8	0	4.991625	1.781064	-1.106913
13	1	0	4.817732	2.721434	-1.194701
14	1	0	4.797348	1.717336	0.965541
15	1	0	3.277609	1.592382	0.056761
16	1	0	-1.651033	1.347361	0.141600
17	8	0	-2.252886	1.907789	0.656169
18	6	0	-3.569557	1.783324	0.166880
19	6	0	-4.123301	0.377062	0.301972
20	1	0	-3.634871	2.089020	-0.889926
21	1	0	-4.181624	2.475760	0.755242
22	8	0	-3.481315	-0.518600	-0.589143
23	1	0	-2.566364	-0.671030	-0.308260
24	1	0	-3.981536	0.043170	1.344003

25	6	0	-5.596727	0.354378	-0.015314
26	8	0	-6.122568	-0.910990	0.327766
27	1	0	-7.044980	-0.943891	0.062375
28	1	0	-5.729593	0.562253	-1.088531
29	1	0	-6.096938	1.154466	0.549449

---

20 Cpl 4-NO2-C6H4-N2(+) * I(-) * 4 GL

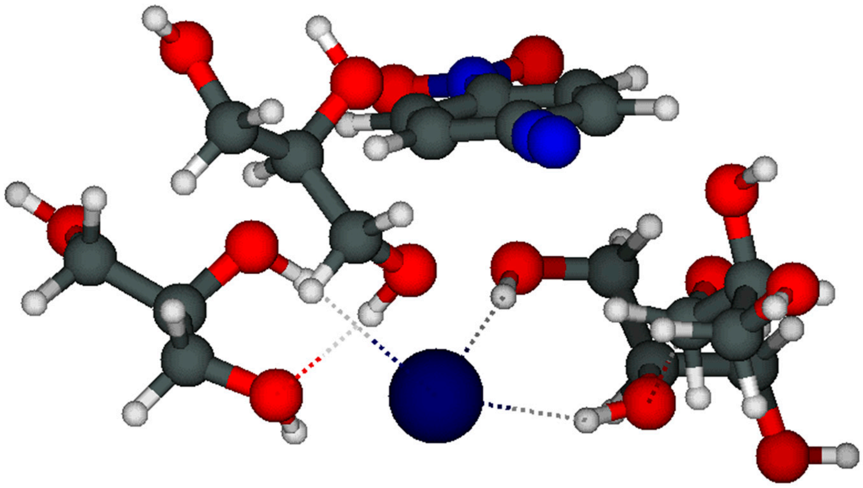

1	6	0	0.178219	0.202461	0.228870
2	6	0	0.201596	0.039195	1.614172
3	6	0	1.442041	-0.099912	2.204200
4	6	0	2.568626	-0.093952	1.397494
5	6	0	2.527587	0.034207	0.017978
6	6	0	1.301882	0.194284	-0.595915
7	1	0	-0.723619	0.011062	2.178151
8	1	0	1.536997	-0.207275	3.277269
9	7	0	3.896699	-0.227560	2.047952
10	1	0	3.439303	0.012581	-0.565953
11	1	0	1.198411	0.317162	-1.667084
12	53	0	1.542976	-3.735146	-1.118955
13	7	0	-1.057870	0.417236	-0.359539
14	7	0	-2.050530	0.605848	-0.790148
15	8	0	4.868757	-0.260368	1.328038
16	8	0	3.921287	-0.282123	3.257652
17	8	0	-0.823102	-0.914045	-2.845075
18	6	0	-1.124439	-0.024502	-3.897564
19	6	0	-0.128041	1.107593	-3.994145
20	1	0	-1.170043	-0.555605	-4.859624
21	1	0	-2.120184	0.399087	-3.708700
22	6	0	-0.375827	1.966893	-5.209375
23	8	0	-0.234728	1.884713	-2.809834
24	1	0	0.890801	0.683653	-4.059341
25	1	0	0.254866	2.701827	-2.966547
26	1	0	-0.199042	-1.571617	-3.202155
27	8	0	0.550588	3.035465	-5.145507
28	1	0	-0.237349	1.369062	-6.122106
29	1	0	-1.410390	2.341343	-5.191573
30	1	0	0.299038	3.717076	-5.773789
31	1	0	1.353343	-4.822812	1.303564
32	8	0	1.031301	-4.798146	2.219918
33	6	0	2.117290	-4.678129	3.135287
34	6	0	2.692284	-3.274895	3.111120
35	6	0	1.586899	-5.007657	4.507072

36	1	0	2.904396	-5.399623	2.868253
37	1	0	3.453237	-3.174061	3.892121
38	8	0	3.323425	-2.948089	1.894947
39	1	0	1.881874	-2.560161	3.342200
40	1	0	2.707185	-3.066570	1.151298
41	1	0	2.349164	-4.749980	5.256374
42	1	0	0.699132	-4.383551	4.699616
43	8	0	1.267023	-6.380579	4.556649
44	1	0	0.850399	-6.571082	5.400939
45	1	0	1.140122	-3.343274	-3.960978
46	8	0	0.786844	-2.576758	-4.435359
47	6	0	1.818130	-1.945036	-5.170165
48	6	0	3.038259	-1.619402	-4.321226
49	1	0	2.123035	-2.563132	-6.025728
50	1	0	1.384334	-1.018183	-5.568641
51	6	0	3.969379	-0.723862	-5.096323
52	8	0	2.677996	-0.962697	-3.122154
53	1	0	3.570712	-2.556385	-4.081853
54	1	0	2.350101	-1.637220	-2.503260
55	8	0	5.218198	-0.679052	-4.442077
56	1	0	4.070845	-1.110473	-6.121554
57	1	0	3.512020	0.277066	-5.157145
58	1	0	5.788314	-0.059554	-4.904541
59	8	0	-0.841166	-2.713926	2.385775
60	6	0	-1.703862	-2.877817	1.287555
61	6	0	-3.015235	-2.193286	1.566830
62	1	0	-1.259436	-2.457362	0.365624
63	1	0	-1.896782	-3.943348	1.088182
64	6	0	-4.026731	-2.464662	0.477319
65	1	0	-3.413839	-2.569431	2.523780
66	8	0	-2.782414	-0.795195	1.670731
67	1	0	-3.649039	-0.371851	1.713912
68	8	0	-5.178039	-1.707635	0.801184
69	1	0	-3.613567	-2.155350	-0.495366
70	1	0	-4.251070	-3.539422	0.433391
71	1	0	-5.765677	-1.673317	0.042373
72	1	0	-0.157616	-3.406774	2.333811

----

21 TS_SN 4-NO2-C6H4-N2(+) * I(-) * 4 GL

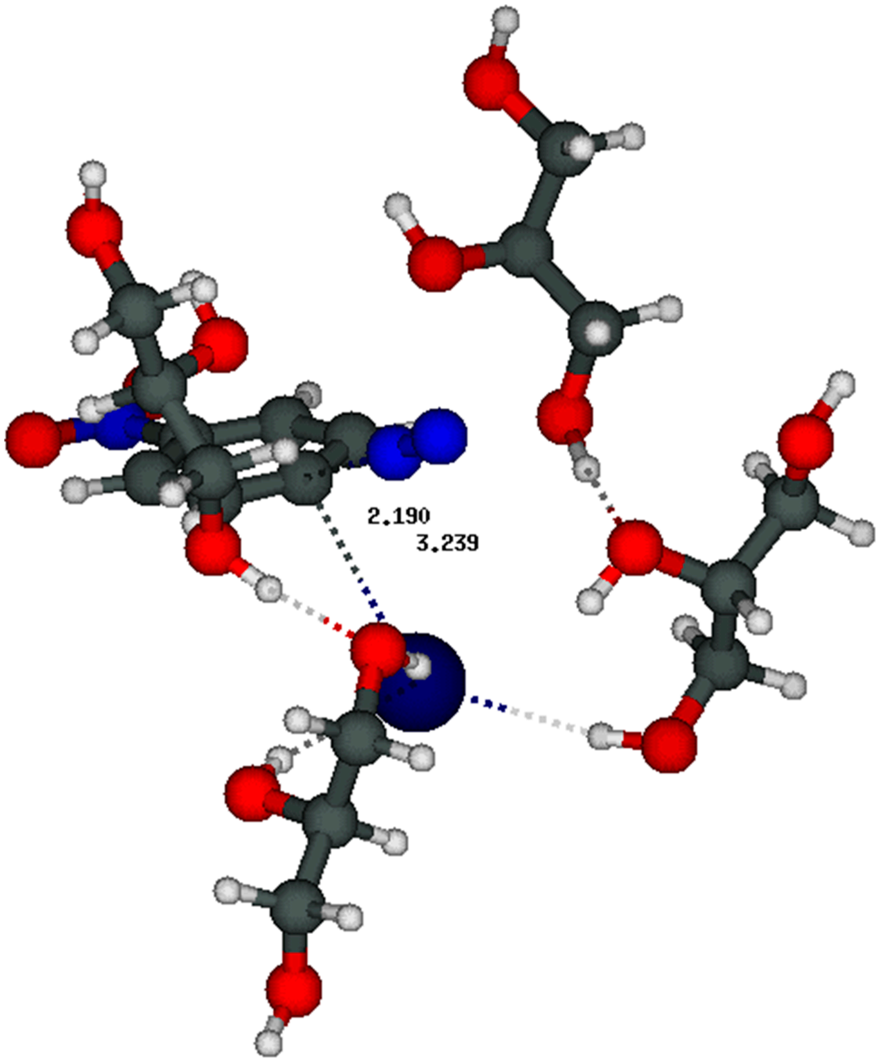

1	6	0	0.125986	0.069579	0.101049
2	6	0	0.097236	0.103248	1.438353
3	6	0	1.415250	0.142770	1.906304
4	6	0	2.465475	0.182727	0.997657
5	6	0	2.290198	0.191470	-0.380327
6	6	0	0.996193	0.153253	-0.911445
7	1	0	-0.806793	0.085348	2.049426
8	1	0	1.603592	0.143784	2.973529
9	7	0	3.840213	0.231727	1.524838
10	1	0	3.142003	0.235154	-1.048898
11	1	0	0.729981	0.186481	-1.970297
12	53	0	-1.477592	-2.668707	-0.550334
13	7	0	-1.811930	0.771079	-0.640771
14	7	0	-2.774624	1.165489	-0.979182
15	8	0	4.749743	0.262768	0.722389
16	8	0	3.980602	0.239059	2.730026
17	8	0	-0.442307	0.449138	-3.536334
18	6	0	-0.848585	1.739632	-3.934689
19	6	0	0.084086	2.766539	-3.347431
20	1	0	-0.836529	1.830123	-5.031908
21	1	0	-1.873172	1.953200	-3.592295
22	6	0	-0.127894	4.125015	-3.973761
23	8	0	-0.137903	2.831888	-1.949995

24	1	0	1.123967	2.447667	-3.543585
25	1	0	0.335596	3.610004	-1.631213
26	1	0	-1.219712	-0.141580	-3.536864
27	8	0	0.735729	5.020407	-3.298088
28	1	0	0.099594	4.082087	-5.047872
29	1	0	-1.179467	4.424528	-3.849772
30	1	0	0.480695	5.924558	-3.497390
31	1	0	-4.140148	-1.611772	0.012574
32	8	0	-4.572375	-1.082161	0.699144
33	6	0	-5.539962	-1.883450	1.374107
34	6	0	-4.930445	-3.190614	1.840529
35	6	0	-6.069243	-1.088581	2.538555
36	1	0	-6.369862	-2.103897	0.684103
37	1	0	-5.670202	-3.743010	2.431150
38	8	0	-4.556617	-4.007817	0.752973
39	1	0	-4.068712	-2.977217	2.493114
40	1	0	-3.643838	-3.806602	0.495082
41	1	0	-6.740168	-1.733839	3.124150
42	1	0	-5.229130	-0.800378	3.191026
43	8	0	-6.749002	0.046070	2.048197
44	1	0	-7.018254	0.591354	2.791961
45	1	0	-2.876670	-1.552282	-2.684881
46	8	0	-2.779068	-1.024309	-3.493470
47	6	0	-2.917138	-1.861653	-4.629672
48	6	0	-2.153536	-3.167502	-4.488939
49	1	0	-3.977262	-2.072513	-4.825371
50	1	0	-2.520796	-1.290824	-5.479516
51	6	0	-2.210902	-3.937348	-5.783334
52	8	0	-0.797476	-2.939075	-4.162470
53	1	0	-2.629499	-3.778136	-3.701904
54	1	0	-0.733151	-2.794712	-3.206268
55	8	0	-1.779633	-5.260660	-5.549129
56	1	0	-3.240894	-3.918131	-6.169603
57	1	0	-1.565295	-3.429949	-6.516654
58	1	0	-1.741046	-5.727120	-6.387877
59	8	0	-2.767628	0.157762	2.399362
60	6	0	-3.372886	1.419338	2.587800
61	6	0	-2.312787	2.455339	2.854145
62	1	0	-3.945182	1.715823	1.695579
63	1	0	-4.071811	1.393814	3.439465
64	6	0	-2.919597	3.758035	3.320750
65	1	0	-1.641901	2.077730	3.647429
66	8	0	-1.575117	2.658309	1.662453
67	1	0	-1.032014	3.443420	1.804258
68	8	0	-1.848631	4.673269	3.459773
69	1	0	-3.644598	4.111716	2.572392
70	1	0	-3.445067	3.607547	4.273917
71	1	0	-2.195381	5.566047	3.529811
72	1	0	-3.335752	-0.370314	1.806564

----

22 Cpl 4-NO2-C6H4-I * N2 * 4 GL

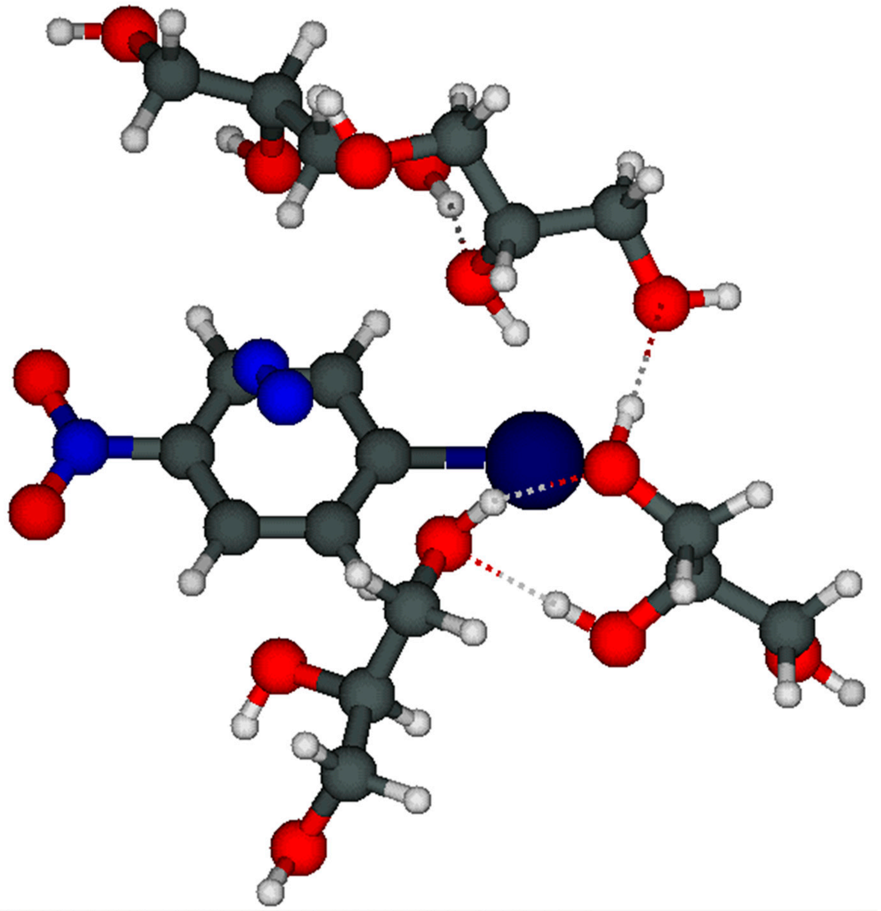

1	6	0	0.555897	0.660672	-1.551556
2	6	0	1.928320	0.456861	-1.646138
3	6	0	2.790441	1.472046	-1.266922
4	6	0	2.255671	2.663196	-0.802474
5	6	0	0.888697	2.870620	-0.696301
6	6	0	0.028005	1.856176	-1.076695
7	1	0	2.341488	-0.487674	-1.985757
8	1	0	3.861471	1.315877	-1.324465
9	7	0	3.169389	3.730241	-0.381711
10	1	0	0.495500	3.799128	-0.301589
11	1	0	-1.041348	1.993128	-0.965388
12	53	0	-0.759963	-0.887148	-2.126322
13	7	0	1.251088	1.121659	2.043631
14	7	0	0.381139	1.143101	2.707713
15	8	0	2.686730	4.781901	-0.004098
16	8	0	4.366009	3.511360	-0.427636
17	8	0	-2.143111	0.823611	1.027578
18	6	0	-2.883812	1.759001	1.784811
19	6	0	-3.077771	3.022745	0.990394
20	1	0	-3.873065	1.347567	2.042193
21	1	0	-2.360496	2.003619	2.722105
22	6	0	-4.079509	3.934273	1.662844
23	8	0	-1.831034	3.680909	0.861430
24	1	0	-3.462233	2.755451	-0.011412
25	1	0	-2.029385	4.581037	0.572922
26	1	0	-2.315349	-0.055788	1.412369
27	8	0	-4.108219	5.130447	0.906225
28	1	0	-5.066406	3.452125	1.688647

29	1	0	-3.758135	4.131802	2.696456
30	1	0	-4.534946	5.823419	1.416008
31	1	0	-0.203243	-1.847622	0.457596
32	8	0	0.574052	-1.914268	1.032071
33	6	0	0.240762	-2.740679	2.136875
34	6	0	-0.398973	-4.020895	1.651130
35	6	0	1.494746	-3.024637	2.919975
36	1	0	-0.468470	-2.204675	2.791140
37	1	0	-0.693506	-4.647833	2.501932
38	8	0	-1.532225	-3.639472	0.881275
39	1	0	0.314889	-4.576414	1.026981
40	1	0	-1.863104	-4.387812	0.376080
41	1	0	1.267627	-3.781892	3.684730
42	1	0	2.251076	-3.449386	2.239710
43	8	0	1.944651	-1.822359	3.501791
44	1	0	2.758204	-1.998592	3.981697
45	1	0	-2.641643	-2.351735	1.496420
46	8	0	-3.242207	-1.609843	1.696689
47	6	0	-4.450623	-1.850409	0.991575
48	6	0	-4.339641	-1.499011	-0.482015
49	1	0	-4.743214	-2.904097	1.102903
50	1	0	-5.228679	-1.231687	1.454692
51	6	0	-5.552434	-1.975381	-1.239845
52	8	0	-4.220970	-0.107593	-0.683153
53	1	0	-3.452937	-2.022227	-0.890261
54	1	0	-3.391357	0.208905	-0.284677
55	8	0	-5.309176	-1.826596	-2.623391
56	1	0	-5.746006	-3.027558	-0.985434
57	1	0	-6.424144	-1.382067	-0.922350
58	1	0	-6.106643	-2.061398	-3.104449
59	8	0	2.968623	-2.436720	-0.316260
60	6	0	3.810932	-1.578287	0.414040
61	6	0	5.142593	-1.461021	-0.279643
62	1	0	3.360760	-0.576789	0.515078
63	1	0	3.985383	-1.960065	1.434295
64	6	0	6.108697	-0.640178	0.542966
65	1	0	5.562770	-2.474107	-0.409227
66	8	0	4.958440	-0.858785	-1.549176
67	1	0	5.839551	-0.648127	-1.881147
68	8	0	7.308916	-0.556335	-0.203844
69	1	0	5.680918	0.360752	0.710350
70	1	0	6.276616	-1.117193	1.518643
71	1	0	7.863841	0.138505	0.158696
72	1	0	2.075139	-2.343347	0.057988

----

23 4-NO2-C6H4-I

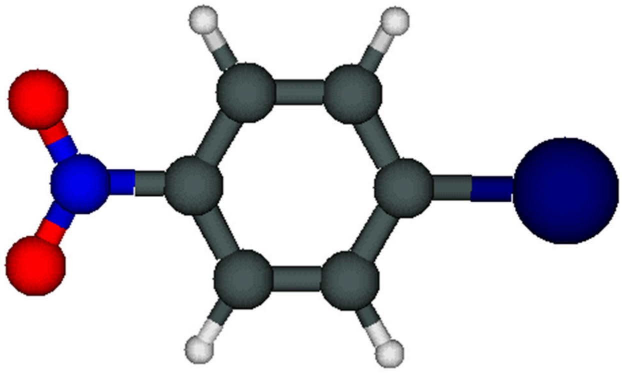

1	6	0	0.000000	0.000000	0.000000
2	6	0	0.000000	-0.000000	1.386320
3	6	0	1.212680	-0.000000	2.052850
4	6	0	2.397211	-0.000000	1.321238
5	6	0	2.383225	0.000000	-0.070946
6	6	0	1.172106	0.000000	-0.740309
7	1	0	-0.936275	-0.000000	1.930790
8	1	0	1.231986	-0.000000	3.136790
9	53	0	4.242274	-0.000000	2.338155
10	1	0	3.309983	0.000000	-0.633456
11	1	0	1.132467	0.000000	-1.822660
12	7	0	-1.285284	0.000000	-0.708394
13	8	0	-1.266056	0.000000	-1.924548
14	8	0	-2.303248	-0.000000	-0.042703

r10 Cpl 4-NO2-C6H4 Rad. I(-) * 2 GL

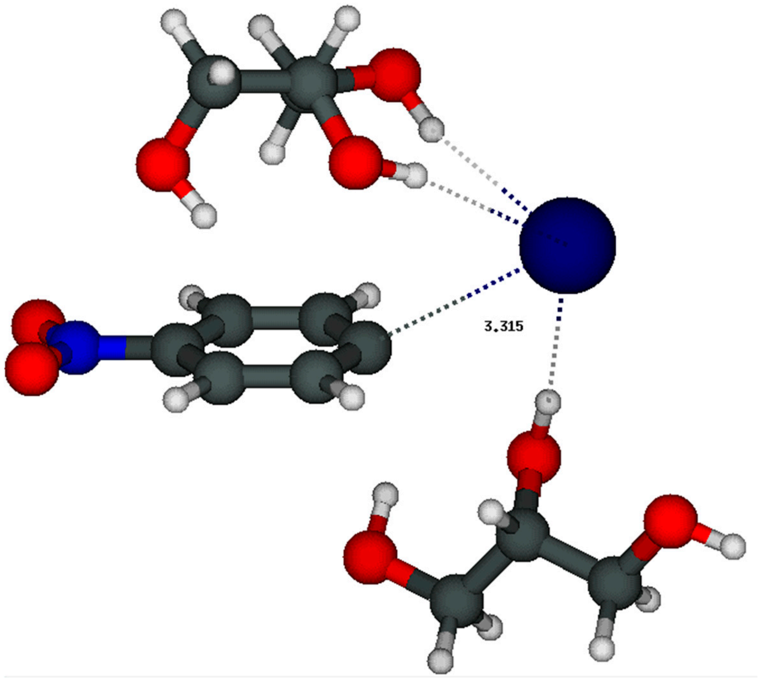

1	53	0	0.085325	-0.176082	-0.187334
2	1	0	0.006646	0.142251	5.263851
3	8	0	0.781778	0.157722	5.839619
4	6	0	1.624176	-0.890389	5.401025
5	6	0	1.813133	-0.870701	3.899203
6	1	0	2.588551	-0.767521	5.903973
7	1	0	1.213855	-1.868254	5.692929
8	6	0	2.501313	0.397565	3.432404
9	8	0	0.510217	-1.016059	3.354948
10	1	0	0.511898	-0.874208	2.394046
11	1	0	2.445715	-1.729341	3.616612
12	8	0	2.860931	0.366661	2.066951
13	1	0	2.067619	0.428000	1.509778
14	1	0	3.433732	0.513625	3.997099
15	1	0	1.864645	1.265552	3.656459
16	1	0	-1.696236	4.426508	0.954119
17	8	0	-2.610959	4.651082	1.169320
18	6	0	-3.383650	4.261838	0.057238
19	6	0	-2.953297	2.913054	-0.478650

20	1	0	-3.303862	5.004898	-0.754344
21	1	0	-4.430198	4.225378	0.375401
22	8	0	-1.575950	3.027780	-0.794595
23	1	0	-1.157279	2.153916	-0.712177
24	1	0	-3.088282	2.147910	0.305612
25	6	0	-3.772907	2.530566	-1.686627
26	8	0	-3.425636	1.214873	-2.064195
27	1	0	-3.846016	1.008634	-2.902712
28	1	0	-3.561236	3.245153	-2.496451
29	1	0	-4.842857	2.603695	-1.437113
30	6	0	-0.664547	2.114177	2.089403
31	6	0	0.295722	3.081597	2.253644
32	6	0	0.415941	3.653584	3.515787
33	6	0	-0.430473	3.211104	4.525064
34	6	0	-1.396207	2.228694	4.328183
35	6	0	-1.512529	1.656324	3.068966
36	1	0	0.943860	3.392307	1.437845
37	1	0	1.153561	4.419726	3.724494
38	7	0	-0.282820	3.785433	5.865860
39	1	0	-2.032322	1.924337	5.152044
40	1	0	-2.243991	0.876015	2.877649
41	8	0	-0.973956	3.335048	6.760935
42	8	0	0.526741	4.681211	6.020739

----

r11 TS_CX

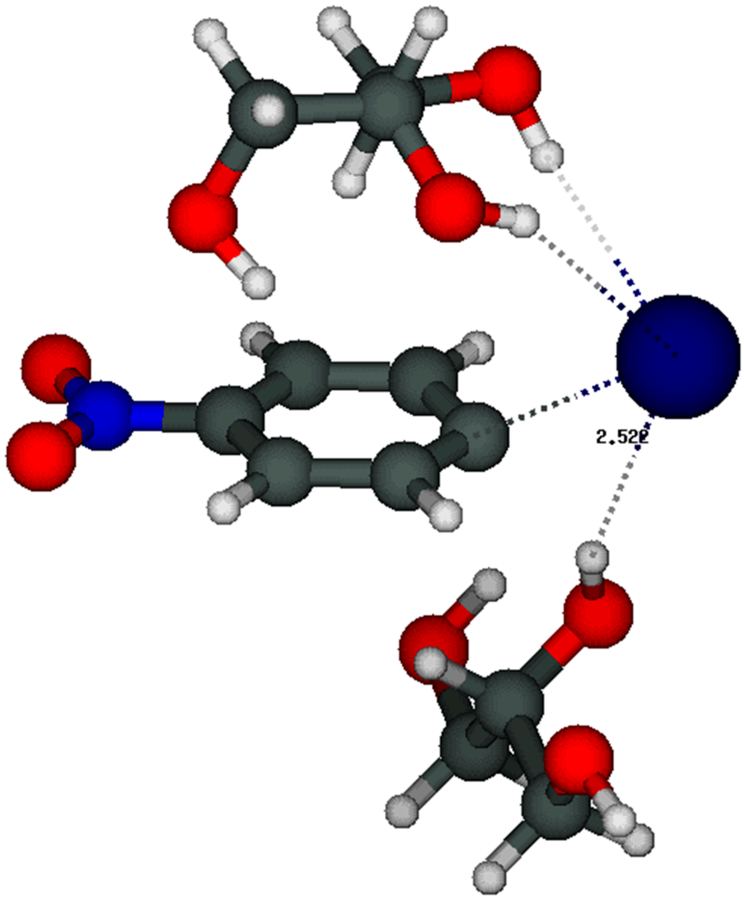

1	53	0	-0.070143	-0.016165	0.033965
2	1	0	0.034202	0.010653	5.148682
3	8	0	0.767307	0.015777	5.778166
4	6	0	1.563151	-1.116758	5.493129
5	6	0	1.826059	-1.269375	4.011276
6	1	0	2.509365	-0.995562	6.030395
7	1	0	1.083126	-2.035459	5.861482
8	6	0	2.578190	-0.084300	3.438704
9	8	0	0.550147	-1.441214	3.412054
10	1	0	0.603115	-1.307984	2.455044
11	1	0	2.441475	-2.172070	3.858300
12	8	0	2.941381	-0.281588	2.085074
13	1	0	2.209502	-0.019327	1.507751
14	1	0	3.507647	0.042542	4.006378
15	1	0	1.984646	0.832862	3.556573
16	1	0	-0.249906	5.424917	-0.050301
17	8	0	-0.791304	6.106205	0.368718
18	6	0	-2.130282	5.760325	0.094269
19	6	0	-2.365384	4.284923	0.325785
20	1	0	-2.393341	6.005915	-0.948303
21	1	0	-2.770397	6.356554	0.751676
22	8	0	-1.444969	3.598177	-0.506529
23	1	0	-1.214183	2.762599	-0.069540
24	1	0	-2.148562	4.048691	1.381951
25	6	0	-3.786046	3.886717	0.013531
26	8	0	-3.941005	2.521409	0.344782
27	1	0	-4.795764	2.218415	0.028696
28	1	0	-3.975688	4.058809	-1.056852

29	1	0	-4.478666	4.518866	0.590432
30	6	0	-0.447443	1.812241	1.729808
31	6	0	0.630913	2.675279	1.931014
32	6	0	0.807480	3.282272	3.158901
33	6	0	-0.077023	2.980801	4.199197
34	6	0	-1.129408	2.075857	4.017454
35	6	0	-1.291269	1.470733	2.787620
36	1	0	1.310904	2.908475	1.113079
37	1	0	1.602079	4.001378	3.323942
38	7	0	0.096580	3.608448	5.474308
39	1	0	-1.812545	1.880440	4.837410
40	1	0	-2.099664	0.757848	2.640881
41	8	0	-0.699062	3.345494	6.376658
42	8	0	1.036912	4.389282	5.627254

---

r12 Cpl 4-NO2-C6H4-I(*-) * 2 GL

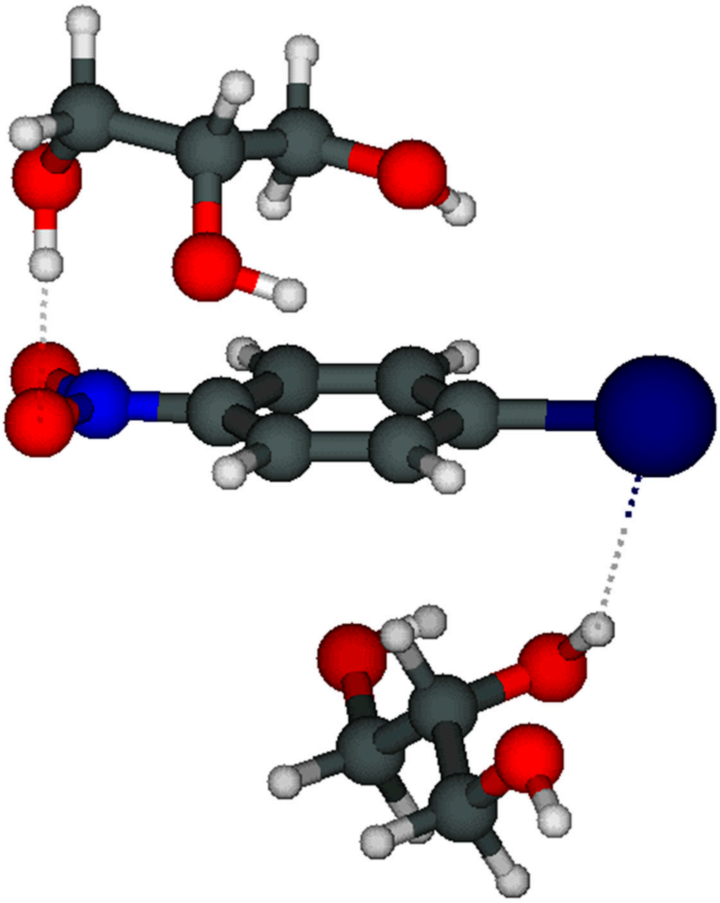

1	53	0	0.199451	-0.131828	-0.304224
2	1	0	-0.107691	-0.044898	6.933344
3	8	0	0.840012	0.002959	7.169006
4	6	0	1.425629	-1.273426	7.200008
5	6	0	1.369141	-2.017473	5.884767
6	1	0	2.477527	-1.134973	7.477320
7	1	0	0.966336	-1.911754	7.970738
8	6	0	1.876275	-1.191290	4.725494
9	8	0	0.029250	-2.420288	5.660398
10	1	0	-0.044984	-2.633741	4.722075
11	1	0	2.013256	-2.910633	5.979636
12	8	0	1.762376	-1.992048	3.557246

13	1	0	1.634343	-1.417854	2.796697
14	1	0	2.921623	-0.902138	4.908156
15	1	0	1.272179	-0.280285	4.628678
16	1	0	-2.088104	4.388972	0.612377
17	8	0	-2.729952	4.506897	1.322783
18	6	0	-3.808510	3.643886	1.038329
19	6	0	-3.324267	2.263525	0.666804
20	1	0	-4.422368	4.041651	0.213200
21	1	0	-4.437118	3.594374	1.932594
22	8	0	-2.486629	2.438069	-0.466823
23	1	0	-2.043737	1.600000	-0.654361
24	1	0	-2.731324	1.852357	1.501143
25	6	0	-4.465467	1.325343	0.367479
26	8	0	-3.906324	0.060286	0.071570
27	1	0	-4.594061	-0.517027	-0.269249
28	1	0	-5.038861	1.714039	-0.487521
29	1	0	-5.135405	1.273402	1.238830
30	6	0	-0.301034	0.471860	1.659135
31	6	0	0.072918	1.736717	2.116448
32	6	0	-0.253612	2.131682	3.399113
33	6	0	-0.947448	1.253216	4.252231
34	6	0	-1.338245	-0.011445	3.773769
35	6	0	-1.017535	-0.394424	2.487040
36	1	0	0.616985	2.417033	1.467904
37	1	0	0.031337	3.109604	3.767514
38	7	0	-1.216046	1.618512	5.564661
39	1	0	-1.871687	-0.681231	4.436244
40	1	0	-1.322103	-1.372820	2.126360
41	8	0	-1.733328	0.751454	6.363442
42	8	0	-0.907126	2.783170	5.975314

----

24 TS_Solv 4-NO2-C6H4-N2(+) * 3 GL

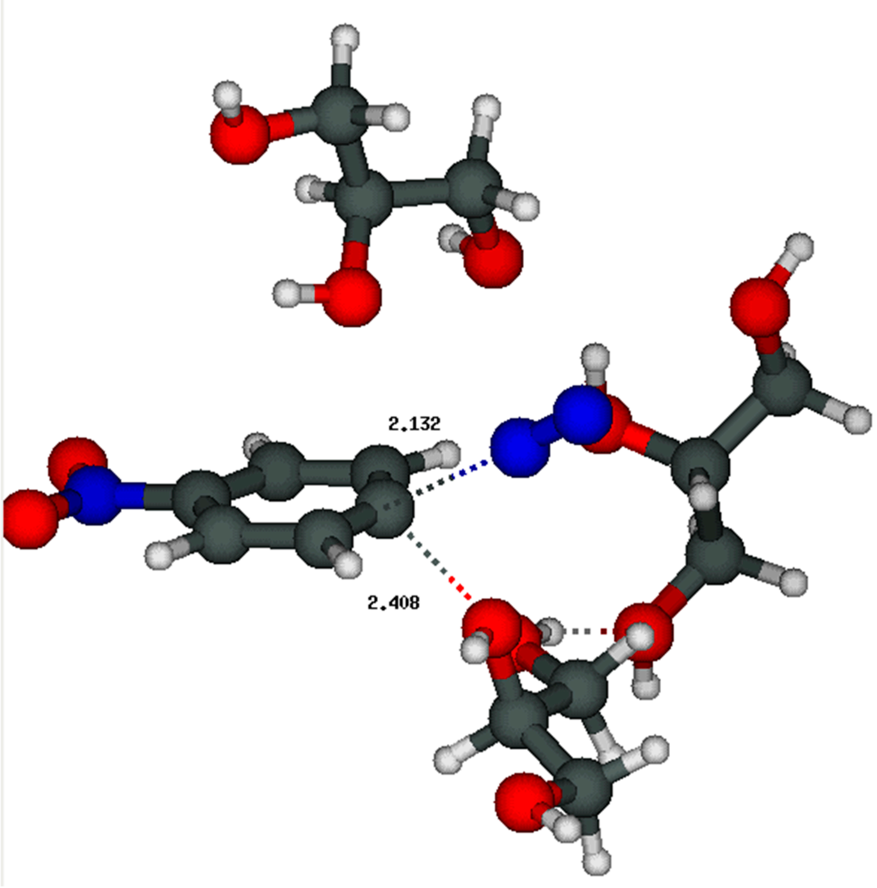

1	6	0	0.019773	0.122886	0.225945
2	6	0	-0.071354	0.111109	1.560686
3	6	0	1.215905	0.022508	2.097219
4	6	0	2.309200	-0.048407	1.244034
5	6	0	2.206606	-0.013840	-0.139870
6	6	0	0.945045	0.075037	-0.737749
7	1	0	-0.999191	0.186661	2.119758
8	1	0	1.347865	0.007072	3.172759
9	7	0	3.652988	-0.157403	1.840679
10	1	0	3.092133	-0.055279	-0.763294
11	1	0	0.736646	0.114448	-1.804090
12	7	0	-1.799689	-0.677679	-0.543529
13	7	0	-2.750330	-1.153308	-0.801131
14	8	0	4.600998	-0.195806	1.085422
15	8	0	3.727487	-0.204273	3.050147
16	8	0	-1.288229	2.034504	-0.431015
17	6	0	-2.618547	2.437513	-0.168808
18	6	0	-2.898365	2.347783	1.306919
19	1	0	-2.764573	3.474986	-0.502410
20	1	0	-3.343041	1.808050	-0.711391
21	1	0	-1.107482	2.208913	-1.377646
22	6	0	-4.196179	3.042662	1.653226
23	8	0	-2.971084	0.979464	1.679808
24	1	0	-2.073187	2.835229	1.854430
25	8	0	-4.437300	2.777220	3.021533
26	1	0	-4.105822	4.120018	1.460753
27	1	0	-5.006112	2.640642	1.026413
28	1	0	-3.442300	0.952660	2.523929
29	1	0	-5.349698	2.987817	3.235177

30	8	0	-1.151096	2.741922	-3.080041
31	6	0	-1.531032	1.987879	-4.221012
32	6	0	-1.886575	0.598572	-3.768087
33	1	0	-2.397505	2.446398	-4.715568
34	1	0	-0.703047	1.932948	-4.940877
35	6	0	-2.481923	-0.216382	-4.891798
36	8	0	-0.718020	-0.015288	-3.269499
37	1	0	-2.643651	0.678664	-2.964670
38	8	0	-2.758152	-1.495734	-4.356373
39	1	0	-3.394874	0.270389	-5.262586
40	1	0	-1.759052	-0.280337	-5.718615
41	1	0	-0.869951	-0.969815	-3.152137
42	1	0	-2.939988	-2.109989	-5.071947
43	1	0	-0.820465	3.603429	-3.349304
44	8	0	-0.093121	-2.525840	-2.306267
45	6	0	-0.487863	-3.723735	-1.663138
46	6	0	0.216142	-3.930240	-0.344240
47	1	0	-1.567478	-3.644604	-1.491509
48	1	0	-0.314427	-4.592850	-2.312402
49	6	0	-0.101538	-5.290408	0.233985
50	1	0	1.308457	-3.867721	-0.508357
51	8	0	-0.187260	-2.911273	0.549615
52	1	0	0.109084	-3.182907	1.427589
53	8	0	0.531128	-5.346177	1.498492
54	1	0	-1.192004	-5.397193	0.334790
55	1	0	0.267282	-6.079589	-0.435623
56	1	0	0.176383	-6.081890	2.003825
57	1	0	0.830233	-2.604452	-2.568236

IRC from TS_Solv 4-NO2-C6H4-N2(+) * 3 GL

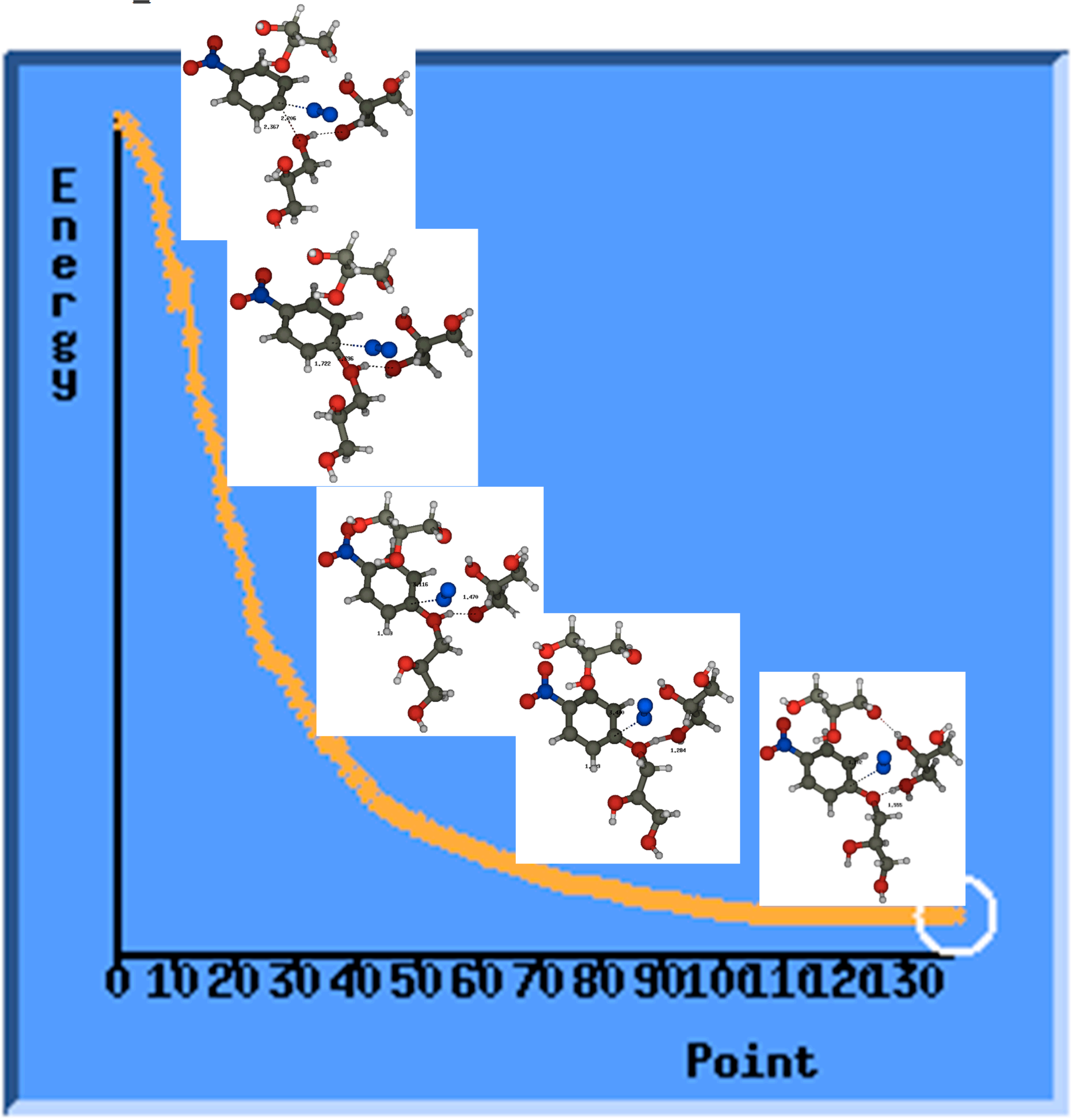

25 Cpl 4-NO2-C6H4-O-C3H7O2 * H(+)GL * GL * N2

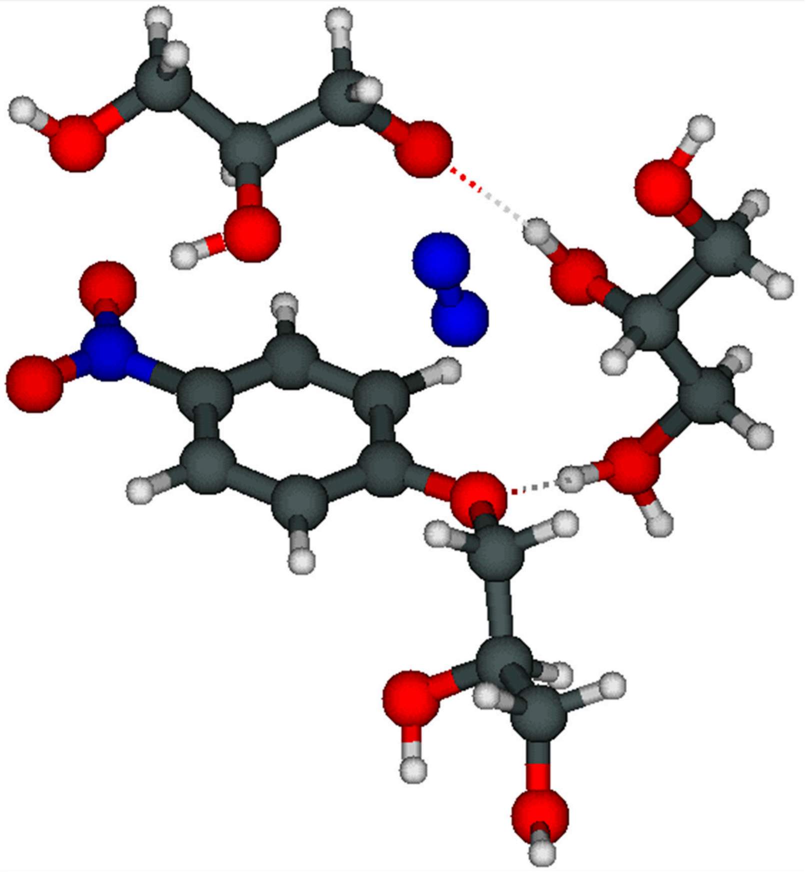

1	6	0	0.636699	-1.086643	-0.571191
2	6	0	0.189950	-2.179965	0.164014
3	6	0	-1.102126	-2.633866	-0.025577
4	6	0	-1.917353	-2.004477	-0.952961
5	6	0	-1.463618	-0.935628	-1.714410
6	6	0	-0.180259	-0.472859	-1.518665
7	1	0	0.845725	-2.698829	0.850805
8	1	0	-1.476666	-3.478842	0.539156
9	7	0	-3.292633	-2.465551	-1.119489
10	1	0	-2.117250	-0.469128	-2.442048
11	1	0	0.184315	0.389066	-2.066458
12	7	0	0.383185	1.201666	1.795960
13	7	0	-0.204531	1.646654	2.604550
14	8	0	-3.999296	-1.883177	-1.923475
15	8	0	-3.670583	-3.403772	-0.440755
16	8	0	1.891155	-0.551583	-0.428311
17	6	0	2.720344	-0.860128	0.708428
18	6	0	3.755826	-1.902089	0.378828
19	1	0	3.213431	0.079995	0.978098
20	1	0	2.091496	-1.165436	1.549250
21	1	0	2.505860	0.440394	-1.456155
22	6	0	4.741190	-2.038928	1.520170
23	8	0	3.103495	-3.127004	0.136427
24	1	0	4.309963	-1.583288	-0.521652
25	8	0	5.623023	-3.077689	1.147816
26	1	0	5.274451	-1.090876	1.675456
27	1	0	4.198517	-2.292081	2.442709
28	1	0	3.786003	-3.809982	0.127316
29	1	0	6.098931	-3.389825	1.921637

30	8	0	2.887784	1.103005	-2.133467
31	6	0	3.068454	2.497040	-1.667059
32	6	0	1.995073	2.779010	-0.656373
33	1	0	4.068391	2.585416	-1.241986
34	1	0	2.963065	3.108615	-2.563958
35	6	0	2.121551	4.193775	-0.132658
36	8	0	0.763138	2.555343	-1.282420
37	1	0	2.124303	2.096211	0.201787
38	8	0	1.132126	4.322615	0.863361
39	1	0	3.130209	4.358274	0.273314
40	1	0	1.956618	4.899523	-0.959470
41	1	0	0.036655	2.650696	-0.640802
42	1	0	1.025135	5.250704	1.087019
43	1	0	3.650772	0.738156	-2.610281
44	8	0	-1.829241	2.471318	-0.320644
45	6	0	-2.952294	2.337117	0.534251
46	6	0	-3.461977	0.917967	0.611582
47	1	0	-2.631345	2.660906	1.529776
48	1	0	-3.763653	3.002076	0.206699
49	6	0	-4.843494	0.845566	1.219409
50	1	0	-3.530369	0.511963	-0.416646
51	8	0	-2.556310	0.145696	1.371027
52	1	0	-3.007003	-0.683301	1.576470
53	8	0	-5.166000	-0.532095	1.279390
54	1	0	-4.829766	1.293051	2.224522
55	1	0	-5.560008	1.399467	0.597541
56	1	0	-5.925876	-0.663067	1.852066
57	1	0	-2.106185	2.264143	-1.219631

----

26 4-NO2-C6H4-O-C3H7O2

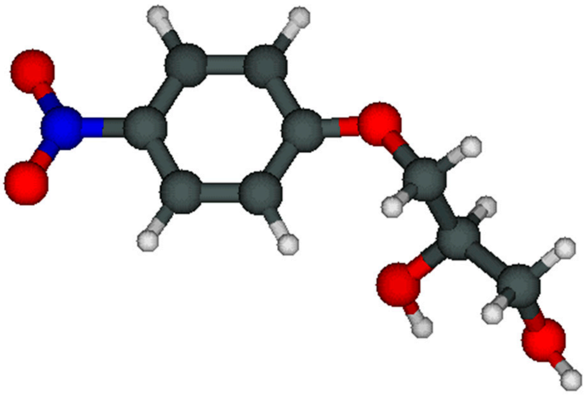

1	6	0	-0.040495	-0.087627	0.036125
2	6	0	0.004293	-0.142872	1.432264
3	6	0	1.224527	-0.093276	2.076819
4	6	0	2.392207	0.021617	1.334646
5	6	0	2.360491	0.093272	-0.055731
6	6	0	1.146707	0.036489	-0.698390
7	1	0	-0.905566	-0.187249	2.015851
8	1	0	1.276390	-0.131271	3.158189
9	7	0	3.669527	0.077828	2.021786
10	1	0	3.283678	0.185889	-0.614506
11	1	0	1.083787	0.080572	-1.780165
12	8	0	4.681925	0.197529	1.350348
13	8	0	3.676442	0.002137	3.240094
14	8	0	-1.158151	-0.144941	-0.702881

15	6	0	-2.426409	-0.444505	-0.128646
16	6	0	-3.220556	0.803924	0.155532
17	1	0	-2.316043	-1.053936	0.775408
18	1	0	-2.950326	-1.044208	-0.878864
19	6	0	-4.652586	0.459100	0.499309
20	8	0	-2.609156	1.497895	1.222686
21	1	0	-3.221401	2.194573	1.489334
22	1	0	-3.219804	1.431961	-0.751836
23	8	0	-5.291648	1.683854	0.801145
24	1	0	-6.121845	1.511052	1.252049
25	1	0	-5.132496	-0.046978	-0.349847
26	1	0	-4.668250	-0.218006	1.366139

27 H(+)GL * GL

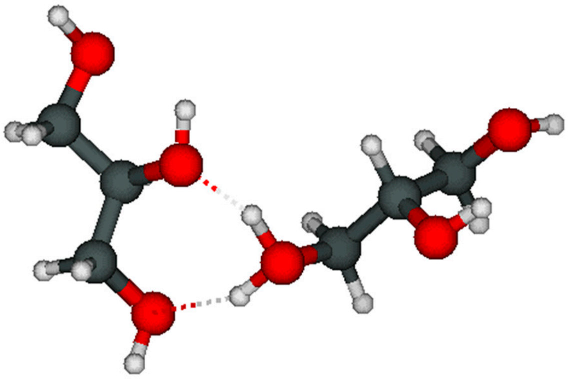

1	8	0	0.334382	0.663636	-0.366258
2	6	0	0.490372	0.810906	1.046786
3	6	0	0.496972	-0.549546	1.688024
4	1	0	-0.772233	-0.363001	-0.923335
5	1	0	0.392993	1.523591	-0.795829
6	1	0	1.442093	1.308815	1.262917
7	1	0	-0.328229	1.411573	1.462370
8	6	0	0.891243	-0.472159	3.144428
9	8	0	-0.820385	-1.114290	1.604628
10	1	0	-0.867436	-1.830080	2.257820
11	1	0	1.199379	-1.206949	1.152505
12	8	0	0.689103	-1.768725	3.666728
13	1	0	0.660601	-1.733058	4.626437
14	1	0	0.259641	0.265849	3.658795
15	1	0	1.939083	-0.156441	3.225805
16	8	0	-1.410749	-1.143903	-0.841491
17	6	0	-1.002271	-2.300689	-1.640061
18	1	0	-1.344167	-1.321048	0.164888
19	6	0	-1.992913	-3.406513	-1.424200
20	1	0	0.006916	-2.597533	-1.339582
21	1	0	-1.005656	-1.960617	-2.676661
22	6	0	-1.515953	-4.672935	-2.104752
23	8	0	-3.233157	-2.992633	-1.941381
24	1	0	-3.795390	-3.776700	-1.979071
25	1	0	-2.077364	-3.612880	-0.341552
26	8	0	-2.544608	-5.619707	-1.911182
27	1	0	-2.428408	-6.352408	-2.521362
28	1	0	-1.354661	-4.474595	-3.174273
29	1	0	-0.567624	-5.005777	-1.660987

Pictures and **Cartesian coordinates** for the reaction of **1f'** in glycerol (see labels in **Table 4**).

28 Cpl 4-CH3O-C6H4-N2(+) * 3 GL

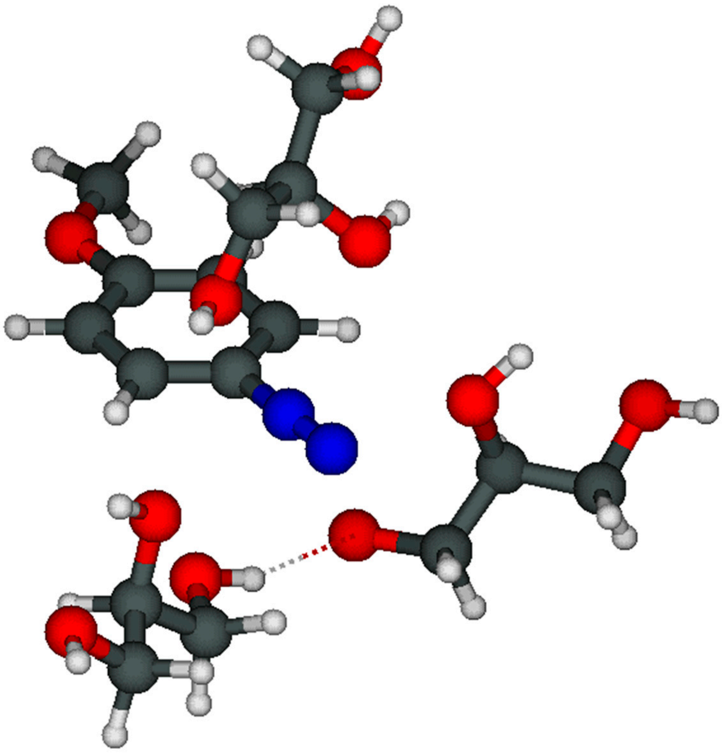

1	6	0	-0.002088	0.016084	-0.034163
2	6	0	0.056437	0.041852	1.366477
3	6	0	1.293203	-0.004461	1.948778
4	6	0	2.453132	-0.072177	1.154762
5	6	0	2.365032	-0.079819	-0.247227
6	6	0	1.130198	-0.033196	-0.850222
7	1	0	-0.868391	0.102812	1.930520
8	1	0	1.406900	0.010564	3.026750
9	8	0	3.597903	-0.122294	1.823608
10	1	0	3.254157	-0.120418	-0.863164
11	1	0	1.005137	-0.032760	-1.928285
12	7	0	-1.227102	0.013167	-0.630135
13	7	0	-2.218184	-0.006085	-1.113213
14	6	0	4.826132	-0.199274	1.106506
15	8	0	-1.603546	2.641161	0.640051
16	6	0	-3.006757	2.667516	0.735598
17	6	0	-3.427352	1.864254	1.938188
18	1	0	-3.384735	3.696513	0.842552
19	1	0	-3.477628	2.232858	-0.162001
20	6	0	-4.900655	2.029982	2.226620
21	8	0	-3.128282	0.498155	1.696626
22	1	0	-2.853286	2.216955	2.813058
23	8	0	-5.197698	1.170575	3.311810
24	1	0	-5.118371	3.078338	2.472898
25	1	0	-5.482143	1.751272	1.334964
26	1	0	-3.597125	-0.009835	2.370745
27	1	0	-6.149679	1.074835	3.394959
28	1	0	-1.331901	2.838044	-0.271422
29	8	0	-1.833986	-2.470288	0.778434
30	6	0	-1.339186	-3.667099	0.211472
31	6	0	-0.115663	-3.337216	-0.599472

32	1	0	-1.067861	-4.394233	0.991491
33	1	0	-2.085918	-4.134283	-0.447560
34	6	0	0.607984	-4.582562	-1.054025
35	8	0	-0.510146	-2.564757	-1.718891
36	1	0	0.575143	-2.747838	0.032420
37	8	0	1.680829	-4.139746	-1.865895
38	1	0	0.968645	-5.147397	-0.183732
39	1	0	-0.080589	-5.221327	-1.626837
40	1	0	2.036676	-4.881227	-2.361943
41	1	0	-2.686678	-2.644484	1.184997
42	8	0	-0.579289	2.620929	-1.964629
43	6	0	-1.194926	2.538473	-3.239161
44	6	0	-0.507056	1.547913	-4.145912
45	1	0	-2.227374	2.215301	-3.062869
46	1	0	-1.226651	3.522877	-3.725101
47	6	0	-1.127391	1.556113	-5.524510
48	1	0	0.559345	1.824871	-4.239350
49	8	0	-0.611268	0.258633	-3.570196
50	1	0	-0.414380	-0.373959	-4.273472
51	8	0	-0.489108	0.526918	-6.255879
52	1	0	-2.207437	1.365101	-5.438361
53	1	0	-0.981622	2.537266	-5.996446
54	1	0	-0.996223	0.332537	-7.048114
55	1	0	0.302453	2.996710	-2.060097
56	1	0	0.228363	-2.586248	-2.341231
57	1	0	5.610072	-0.234988	1.860060
58	1	0	4.863603	-1.106342	0.496137
59	1	0	4.964528	0.684123	0.476409

29 Cpl 4-CH3O-C6H4-N2(+) * F(-) * 4 GL

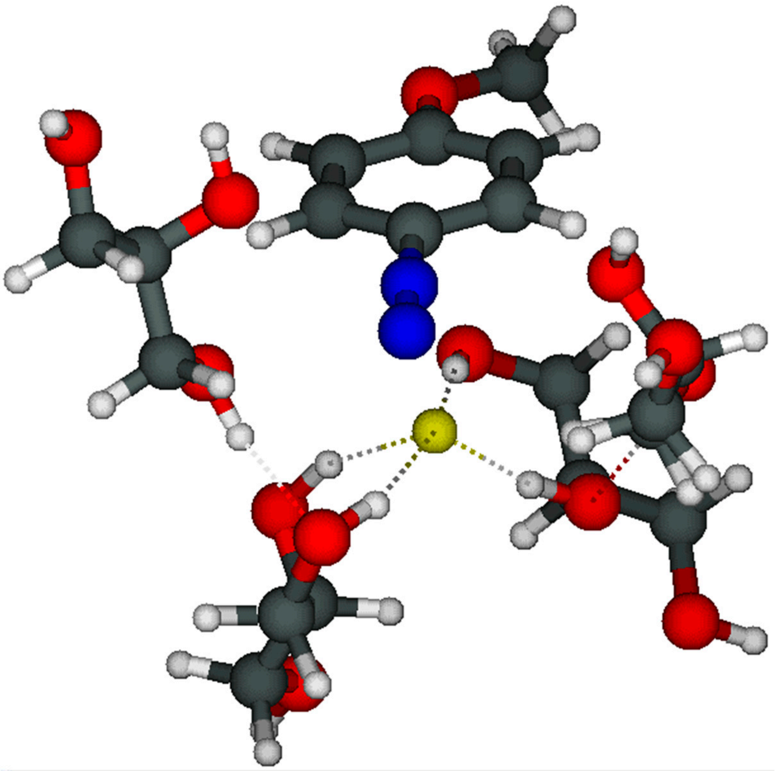

1	6	0	-0.020012	0.041737	0.034904
2	6	0	0.027843	-0.009714	1.432633
3	6	0	1.261106	-0.011273	2.036283

4	6	0	2.421723	0.014623	1.247026
5	6	0	2.339729	0.096832	-0.155604
6	6	0	1.123647	0.096170	-0.778006
7	1	0	-0.896347	-0.073792	1.999854
8	1	0	1.320527	-0.064004	3.116129
9	8	0	3.651187	-0.043667	1.739064
10	1	0	3.261777	0.122229	-0.725114
11	1	0	1.006606	0.101908	-1.855791
12	9	0	-0.563350	-2.748233	-0.244657
13	7	0	-1.235422	-0.079720	-0.561348
14	7	0	-2.232603	-0.194810	-1.021846
15	6	0	3.833728	-0.224940	3.139717
16	8	0	-0.379637	-0.956496	-3.368354
17	6	0	-1.207574	-0.141331	-4.160385
18	6	0	-0.682294	1.269777	-4.131420
19	1	0	-1.233821	-0.489248	-5.205370
20	1	0	-2.245063	-0.141482	-3.785760
21	6	0	-1.395675	2.147401	-5.132494
22	8	0	-0.850450	1.787045	-2.821920
23	1	0	0.392967	1.247965	-4.383850
24	1	0	-0.674218	2.734311	-2.873457
25	1	0	-0.857264	-1.782913	-3.155013
26	8	0	-0.877434	3.454502	-4.965738
27	1	0	-1.220859	1.773529	-6.150788
28	1	0	-2.477394	2.125546	-4.931871
29	1	0	-1.443174	4.084521	-5.418786
30	1	0	-0.794613	-3.850822	0.929034
31	8	0	-0.845087	-4.374383	1.764014
32	6	0	0.447282	-4.848793	2.101752
33	6	0	1.422128	-3.701345	2.312114
34	6	0	0.324653	-5.676681	3.355348
35	1	0	0.831525	-5.488676	1.286984
36	1	0	2.351605	-4.086426	2.747860
37	8	0	1.771800	-3.040946	1.121300
38	1	0	0.978433	-2.997787	3.042107
39	1	0	0.953987	-2.766424	0.661945
40	1	0	1.330856	-5.953671	3.701680
41	1	0	-0.147183	-5.061664	4.139144
42	8	0	-0.448498	-6.825462	3.076374
43	1	0	-0.577108	-7.315982	3.892025
44	1	0	-1.529709	-3.046122	-1.567375
45	8	0	-1.832707	-3.136843	-2.500147
46	6	0	-1.404832	-4.381571	-3.020451
47	6	0	-0.217696	-4.963560	-2.267759
48	1	0	-2.232076	-5.106070	-3.002624
49	1	0	-1.123676	-4.221895	-4.072018
50	6	0	0.290454	-6.192137	-2.975568
51	8	0	0.835121	-4.029661	-2.161348
52	1	0	-0.550184	-5.263824	-1.257495
53	1	0	0.561722	-3.396548	-1.471773
54	8	0	1.162038	-6.892211	-2.111095
55	1	0	-0.563712	-6.820737	-3.268968
56	1	0	0.809440	-5.875514	-3.893500
57	1	0	1.536701	-7.637390	-2.587372
58	8	0	-2.057751	-2.176784	2.970134
59	6	0	-3.116498	-2.154010	2.041768
60	6	0	-3.907049	-0.885753	2.213366
61	1	0	-2.731765	-2.205208	1.008076
62	1	0	-3.792911	-3.011898	2.187332

63	6	0	-5.185765	-0.916885	1.409111
64	1	0	-4.165164	-0.771682	3.280241
65	8	0	-3.102428	0.208879	1.801945
66	1	0	-3.677506	0.983575	1.774708
67	8	0	-5.801964	0.346079	1.582277
68	1	0	-4.950102	-1.096990	0.349211
69	1	0	-5.832133	-1.730778	1.765727
70	1	0	-6.493412	0.459346	0.925554
71	1	0	-1.523751	-2.963138	2.752520
72	1	0	4.908558	-0.290667	3.295284
73	1	0	3.434829	0.626034	3.699488
74	1	0	3.356379	-1.152053	3.471410

30 TS_SN 4-CH3O-C6H4-N2(+) * F(-) * 4 GL

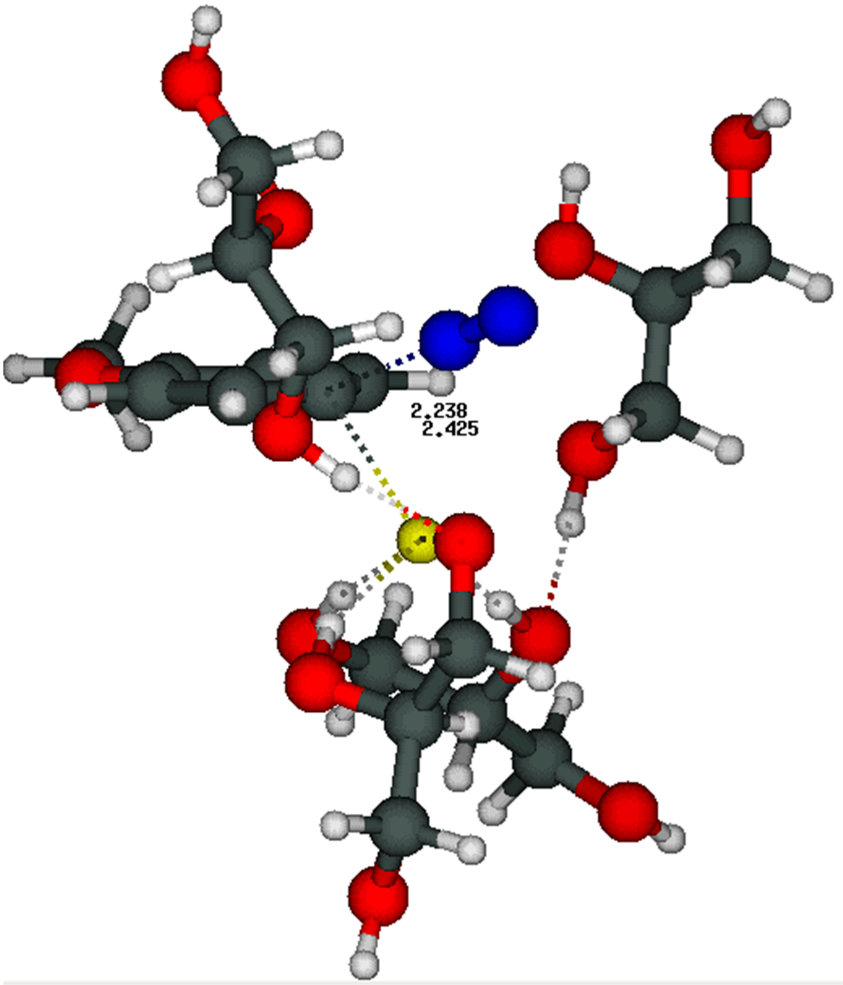

1	6	0	0.025252	0.031304	0.070669
2	6	0	0.007685	0.027786	1.398814
3	6	0	1.343340	0.020167	1.849916
4	6	0	2.407884	0.015170	0.945331
5	6	0	2.176392	0.015289	-0.438241
6	6	0	0.879886	0.024916	-0.957167
7	1	0	-0.865469	0.004152	2.051732
8	1	0	1.499781	0.008535	2.922160
9	8	0	3.700108	0.008712	1.310309
10	1	0	3.019180	0.005795	-1.121821
11	1	0	0.601294	0.022740	-2.012605
12	9	0	-1.443912	-1.805954	-0.520079

13	7	0	-1.929154	0.779805	-0.721747
14	7	0	-2.863533	1.158283	-1.145848
15	6	0	4.009468	-0.005372	2.693678
16	8	0	-0.613389	-0.164762	-3.642931
17	6	0	-1.113524	1.002427	-4.253602
18	6	0	-0.193467	2.159322	-3.964193
19	1	0	-1.190684	0.872230	-5.344385
20	1	0	-2.118664	1.245718	-3.874713
21	6	0	-0.540224	3.363813	-4.807570
22	8	0	-0.293059	2.479611	-2.588274
23	1	0	0.841191	1.851089	-4.200102
24	1	0	0.173685	3.314906	-2.464671
25	1	0	-1.364845	-0.748556	-3.415482
26	8	0	0.331032	4.404694	-4.404766
27	1	0	-0.414001	3.125244	-5.872733
28	1	0	-1.590710	3.641192	-4.632543
29	1	0	0.010973	5.242612	-4.747882
30	1	0	-2.130972	-2.748945	0.658064
31	8	0	-2.394772	-3.230219	1.475026
32	6	0	-1.450305	-4.250217	1.764189
33	6	0	-0.049515	-3.682464	1.920056
34	6	0	-1.890231	-4.938721	3.030931
35	1	0	-1.439434	-4.987649	0.942353
36	1	0	0.615641	-4.456135	2.320113
37	8	0	0.510889	-3.249819	0.704740
38	1	0	-0.084283	-2.860105	2.658861
39	1	0	-0.103498	-2.621532	0.282443
40	1	0	-1.114335	-5.656138	3.334397
41	1	0	-1.985775	-4.184995	3.830031
42	8	0	-3.120699	-5.590660	2.797771
43	1	0	-3.419938	-5.987686	3.619573
44	1	0	-2.486831	-1.682160	-1.848414
45	8	0	-2.788356	-1.630811	-2.781840
46	6	0	-2.914734	-2.938164	-3.309091
47	6	0	-2.016072	-3.942959	-2.605704
48	1	0	-3.958401	-3.278055	-3.242723
49	1	0	-2.644563	-2.890430	-4.373679
50	6	0	-2.059235	-5.268480	-3.318933
51	8	0	-0.678172	-3.492106	-2.562474
52	1	0	-2.388310	-4.094950	-1.576556
53	1	0	-0.644581	-2.816627	-1.864726
54	8	0	-1.493856	-6.256971	-2.482612
55	1	0	-3.102957	-5.511461	-3.568811
56	1	0	-1.498384	-5.174605	-4.261585
57	1	0	-1.447803	-7.084192	-2.968511
58	8	0	-2.533184	-0.949210	3.024879
59	6	0	-3.626272	-0.370519	2.348004
60	6	0	-3.688410	1.098394	2.667663
61	1	0	-3.517682	-0.496973	1.257333
62	1	0	-4.575254	-0.842753	2.645909
63	6	0	-4.937218	1.734181	2.102744
64	1	0	-3.697679	1.220348	3.764958
65	8	0	-2.534508	1.719379	2.129114
66	1	0	-2.672137	2.671891	2.196195
67	8	0	-4.858711	3.115213	2.404696
68	1	0	-4.971910	1.572246	1.014893
69	1	0	-5.828622	1.275224	2.552698
70	1	0	-5.503587	3.595252	1.879415
71	1	0	-2.419719	-1.842535	2.645448

72	1	0	5.095875	-0.010607	2.762936
73	1	0	3.618742	0.887517	3.193955
74	1	0	3.611108	-0.903985	3.177365

---

31 Cpl 4-CH3O-C6H4-F * N2 * 4 GL

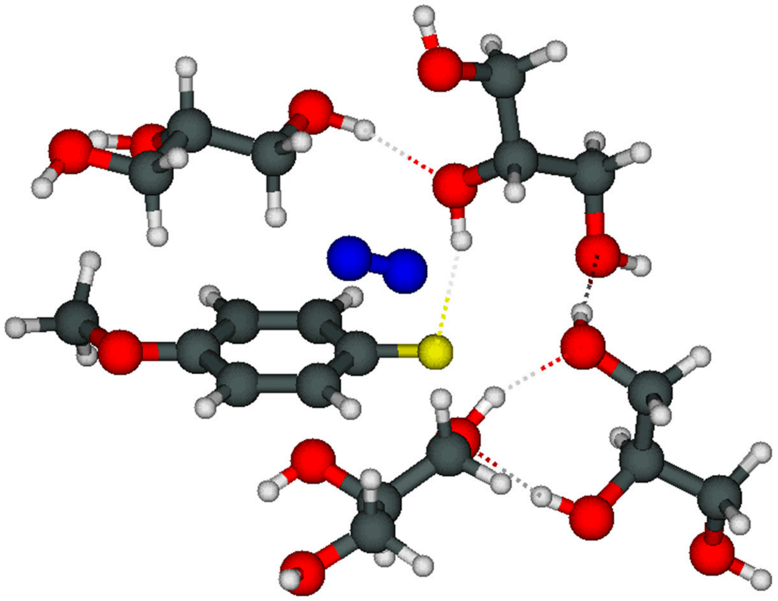

1	6	0	-0.034370	0.002658	0.160698
2	6	0	-0.020950	0.020171	1.536313
3	6	0	1.204340	0.044560	2.196098
4	6	0	2.386016	0.059869	1.459075
5	6	0	2.335008	0.026723	0.063922
6	6	0	1.120588	-0.006335	-0.596564
7	1	0	-0.953767	0.027166	2.091231
8	1	0	1.218689	0.075943	3.278694
9	8	0	3.625815	0.112399	2.003460
10	1	0	3.264873	0.034331	-0.497404
11	1	0	1.062873	-0.023953	-1.681479
12	9	0	-1.236075	0.010905	-0.483941
13	7	0	1.231416	3.099138	-2.098658
14	7	0	0.237891	2.658025	-2.229682
15	6	0	3.730715	0.118069	3.413978
16	8	0	-0.025767	-0.043613	-3.625777
17	6	0	0.733758	0.346329	-4.749221
18	6	0	2.080426	-0.321869	-4.687028
19	1	0	0.226471	0.060837	-5.685737
20	1	0	0.879168	1.437512	-4.761913
21	6	0	2.867955	-0.084329	-5.954283
22	8	0	2.780381	0.191156	-3.568462
23	1	0	1.929915	-1.409103	-4.565050
24	1	0	3.693938	-0.106820	-3.655490
25	1	0	-0.787713	0.563627	-3.540739
26	8	0	4.133764	-0.689019	-5.763094
27	1	0	2.339864	-0.522473	-6.812654
28	1	0	2.971914	0.998137	-6.122009
29	1	0	4.744477	-0.373151	-6.433514
30	1	0	-1.964203	1.827495	-0.112733
31	8	0	-1.985147	2.756350	0.156896
32	6	0	-2.684798	3.501504	-0.825270
33	6	0	-4.087543	2.967374	-0.988920

34	6	0	-2.677574	4.947379	-0.409471
35	1	0	-2.161514	3.417904	-1.793441
36	1	0	-4.628836	3.550218	-1.745582
37	8	0	-3.953922	1.610005	-1.393087
38	1	0	-4.627982	3.031817	-0.035081
39	1	0	-4.800918	1.159011	-1.331831
40	1	0	-3.395708	5.505431	-1.028271
41	1	0	-3.007007	5.015864	0.638779
42	8	0	-1.366574	5.445133	-0.570008
43	1	0	-1.304838	6.302226	-0.140802
44	1	0	-2.882022	1.343175	-2.838080
45	8	0	-2.440057	1.191318	-3.694794
46	6	0	-3.329733	0.426444	-4.492731
47	6	0	-3.341642	-1.044379	-4.109025
48	1	0	-4.345030	0.841003	-4.417233
49	1	0	-2.998518	0.523516	-5.534048
50	6	0	-4.493504	-1.749494	-4.780487
51	8	0	-2.151809	-1.702066	-4.489084
52	1	0	-3.485145	-1.113409	-3.013363
53	1	0	-1.384178	-1.258585	-4.087230
54	8	0	-4.587000	-3.064223	-4.273493
55	1	0	-5.418716	-1.187439	-4.587268
56	1	0	-4.318058	-1.753921	-5.867889
57	1	0	-5.279948	-3.529058	-4.749084
58	8	0	-0.210055	3.767862	2.088521
59	6	0	0.938953	3.592164	1.295353
60	6	0	2.169099	4.022439	2.046478
61	1	0	1.056129	2.536187	1.002798
62	1	0	0.868342	4.191679	0.373665
63	6	0	3.393177	3.868377	1.170908
64	1	0	2.065738	5.085563	2.328981
65	8	0	2.311625	3.236009	3.215398
66	1	0	3.199486	3.413640	3.549347
67	8	0	4.514081	4.211207	1.967937
68	1	0	3.461819	2.823194	0.829862
69	1	0	3.313900	4.523068	0.291292
70	1	0	5.315794	3.892962	1.545936
71	1	0	-0.955589	3.484617	1.533641
72	1	0	4.794919	0.171102	3.643102
73	1	0	3.220338	0.987922	3.841966
74	1	0	3.315929	-0.800906	3.845989

----

32 4-CH3O-C6H4-F

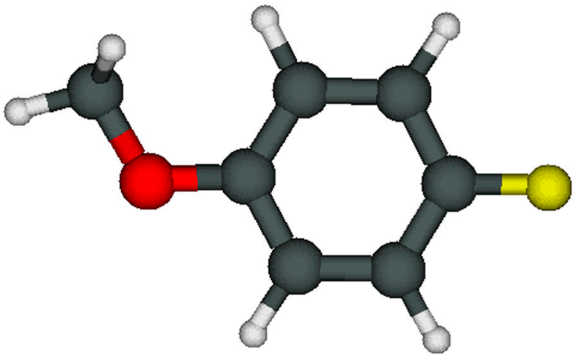

1	6	0	-0.018881	0.000000	-0.002541
2	6	0	0.024605	0.000000	1.379980
3	6	0	1.257204	0.000000	2.006254

4	6	0	2.433898	-0.000000	1.254917
5	6	0	2.365424	-0.000000	-0.135813
6	6	0	1.125107	-0.000000	-0.767430
7	1	0	-0.897683	0.000000	1.950920
8	1	0	1.329485	0.000000	3.089041
9	8	0	3.588133	-0.000000	1.964721
10	1	0	3.263110	-0.000000	-0.742295
11	1	0	1.051322	-0.000000	-1.849700
12	9	0	-1.220459	0.000000	-0.617487
13	6	0	4.807288	-0.000000	1.251778
14	1	0	5.601261	-0.000000	1.997829
15	1	0	4.904137	-0.894131	0.624385
16	1	0	4.904137	0.894131	0.624385

33 Cpl 4-CH3O-C6H4-N2(+) * Cl(-) * 4 GL

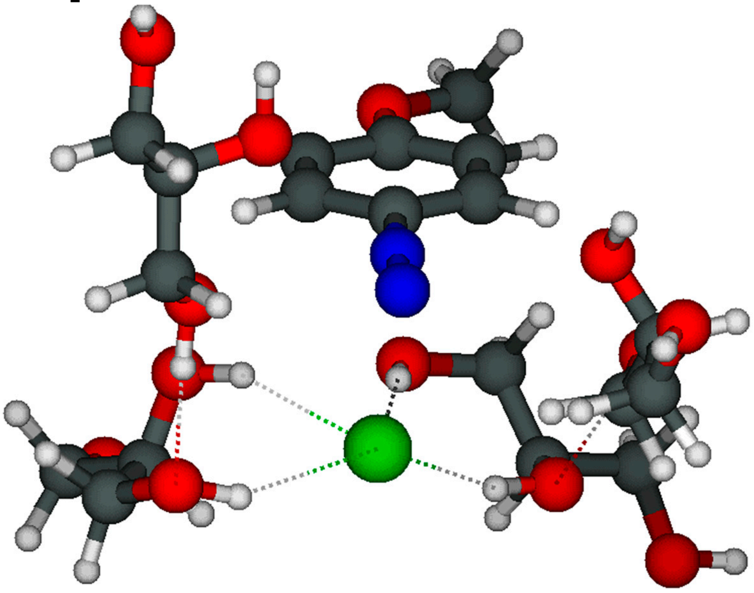

1	6	0	0.041636	-0.020315	0.055284
2	6	0	0.096867	-0.070089	1.452422
3	6	0	1.328912	0.017090	2.055048
4	6	0	2.484315	0.129381	1.266026
5	6	0	2.393241	0.191739	-0.137376
6	6	0	1.178832	0.115375	-0.758621
7	1	0	-0.816969	-0.195517	2.024971
8	1	0	1.389482	-0.018247	3.135801
9	8	0	3.714106	0.179915	1.757611
10	1	0	3.309528	0.277872	-0.710558
11	1	0	1.060975	0.121082	-1.837770
12	17	0	-0.270432	-3.350770	-0.601860
13	7	0	-1.174016	-0.197201	-0.534083
14	7	0	-2.170756	-0.367040	-0.977328
15	6	0	3.909501	0.075575	3.164653
16	8	0	-0.227525	-0.545192	-3.395413
17	6	0	-1.120628	0.221129	-4.162589
18	6	0	-0.859117	1.678833	-3.886965
19	1	0	-0.991351	0.036426	-5.239808
20	1	0	-2.169664	-0.005585	-3.907079
21	6	0	-1.681367	2.568509	-4.789158
22	8	0	-1.166796	1.942971	-2.528365

23	1	0	0.211174	1.882520	-4.067802
24	1	0	-1.169386	2.902768	-2.428452
25	1	0	-0.284627	-1.494436	-3.619159
26	8	0	-1.414362	3.901961	-4.394364
27	1	0	-1.403949	2.397792	-5.838625
28	1	0	-2.747981	2.329137	-4.664994
29	1	0	-2.075393	4.487040	-4.772443
30	1	0	-0.045762	-4.392742	1.332802
31	8	0	-0.030263	-4.527765	2.298719
32	6	0	1.304745	-4.676099	2.764978
33	6	0	2.113788	-3.410438	2.544777
34	6	0	1.233302	-5.006735	4.234206
35	1	0	1.792263	-5.508887	2.232968
36	1	0	3.076204	-3.503253	3.061157
37	8	0	2.408973	-3.153990	1.192951
38	1	0	1.566683	-2.565047	3.001395
39	1	0	1.583498	-3.053042	0.687950
40	1	0	2.249383	-4.988528	4.653674
41	1	0	0.642859	-4.227339	4.743752
42	8	0	0.641450	-6.278027	4.391453
43	1	0	0.542537	-6.458626	5.329692
44	1	0	-0.284905	-3.609248	-2.857551
45	8	0	-0.124721	-3.286068	-3.762543
46	6	0	1.146303	-3.717869	-4.213811
47	6	0	2.191402	-3.760227	-3.110711
48	1	0	1.069962	-4.712017	-4.677148
49	1	0	1.461995	-3.007429	-4.989771
50	6	0	3.541816	-4.082121	-3.696811
51	8	0	2.281772	-2.525397	-2.430550
52	1	0	1.925769	-4.557985	-2.395260
53	1	0	1.535845	-2.480918	-1.810496
54	8	0	4.424651	-4.431181	-2.651147
55	1	0	3.436917	-4.906850	-4.417496
56	1	0	3.902107	-3.197902	-4.244877
57	1	0	5.305666	-4.547683	-3.015558
58	8	0	-1.576030	-2.334574	3.109139
59	6	0	-2.659614	-2.537314	2.231056
60	6	0	-3.634609	-1.399555	2.362698
61	1	0	-2.311407	-2.596933	1.185170
62	1	0	-3.186612	-3.477872	2.459205
63	6	0	-4.896720	-1.662935	1.575284
64	1	0	-3.899612	-1.277220	3.426930
65	8	0	-3.013509	-0.213169	1.891125
66	1	0	-3.702267	0.461119	1.840277
67	8	0	-5.706025	-0.509058	1.711752
68	1	0	-4.637351	-1.837786	0.519845
69	1	0	-5.403968	-2.556764	1.963903
70	1	0	-6.405099	-0.527656	1.053609
71	1	0	-0.947568	-3.061321	2.944937
72	1	0	4.985419	0.115252	3.320897
73	1	0	3.432675	0.909675	3.687536
74	1	0	3.519735	-0.875857	3.539515

----

34 TS_SN 4-CH3O-C6H4-N2(+) * Cl(-) * 4 GL

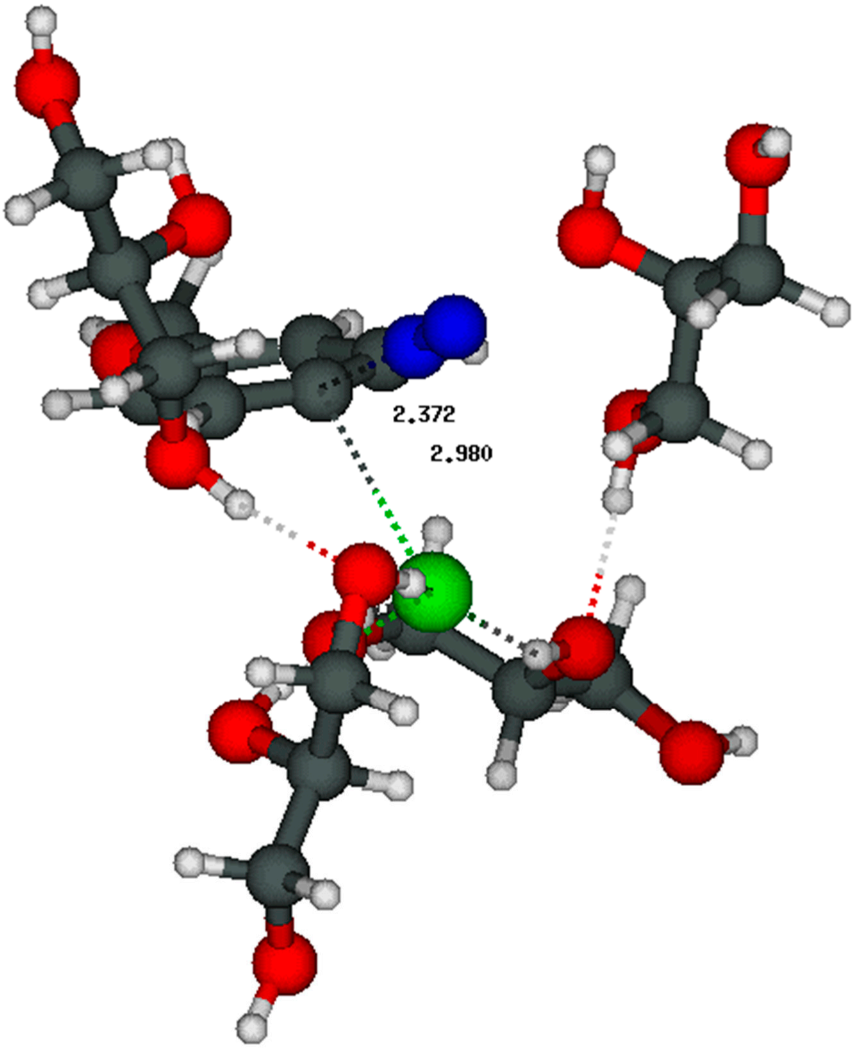

1	6	0	-0.281094	-0.300511	-0.018027
2	6	0	-0.294562	-0.203291	1.303028
3	6	0	1.047748	-0.281820	1.735936
4	6	0	2.094979	-0.424821	0.822682
5	6	0	1.843069	-0.478150	-0.556566
6	6	0	0.541442	-0.402340	-1.062188
7	1	0	-1.149812	-0.127629	1.975781
8	1	0	1.215654	-0.237847	2.805943
9	8	0	3.385746	-0.518766	1.175456
10	1	0	2.670563	-0.581576	-1.251285
11	1	0	0.253641	-0.418852	-2.117122
12	17	0	-2.220330	-2.499178	-0.552373
13	7	0	-2.280372	0.609533	-0.913340
14	7	0	-3.187026	1.020807	-1.366888
15	6	0	3.703972	-0.550847	2.557167
16	8	0	-0.790222	-0.349299	-3.786077
17	6	0	-1.096285	0.894204	-4.375406
18	6	0	-0.100238	1.928435	-3.920855
19	1	0	-1.062669	0.823471	-5.473789
20	1	0	-2.107013	1.226309	-4.092021
21	6	0	-0.209064	3.195265	-4.737063
22	8	0	-0.333164	2.206098	-2.552340
23	1	0	0.916648	1.514389	-4.048521

24	1	0	0.197887	2.981769	-2.334673
25	1	0	-1.615154	-0.863714	-3.683473
26	8	0	0.710071	4.116259	-4.178869
27	1	0	0.024079	2.984909	-5.790049
28	1	0	-1.237784	3.581149	-4.676186
29	1	0	0.518549	4.998815	-4.505262
30	1	0	-2.370734	-3.627254	1.390999
31	8	0	-2.365736	-3.734697	2.358949
32	6	0	-1.201061	-4.434595	2.783470
33	6	0	0.065499	-3.685046	2.411184
34	6	0	-1.298861	-4.601350	4.278255
35	1	0	-1.178593	-5.430334	2.312485
36	1	0	0.925282	-4.168793	2.888148
37	8	0	0.326174	-3.678549	1.028176
38	1	0	-0.002011	-2.658083	2.812118
39	1	0	-0.376946	-3.199002	0.558557
40	1	0	-0.352535	-5.017729	4.652266
41	1	0	-1.436422	-3.608831	4.737959
42	8	0	-2.382497	-5.455058	4.575113
43	1	0	-2.480265	-5.509963	5.529136
44	1	0	-3.264472	-1.879674	-2.506941
45	8	0	-3.220997	-1.604065	-3.439245
46	6	0	-3.381948	-2.733348	-4.280369
47	6	0	-2.697640	-3.977297	-3.737004
48	1	0	-4.448570	-2.943838	-4.440782
49	1	0	-2.941174	-2.463763	-5.249540
50	6	0	-2.753324	-5.078901	-4.763821
51	8	0	-1.345317	-3.725160	-3.415401
52	1	0	-3.235985	-4.316069	-2.835364
53	1	0	-1.323394	-3.326146	-2.530436
54	8	0	-2.415139	-6.301942	-4.144535
55	1	0	-3.764591	-5.119922	-5.195602
56	1	0	-2.049592	-4.833360	-5.574105
57	1	0	-2.380327	-6.989053	-4.814739
58	8	0	-2.660049	-1.077298	3.155873
59	6	0	-3.791029	-0.683434	2.408222
60	6	0	-3.986883	0.800198	2.561825
61	1	0	-3.650936	-0.922883	1.339794
62	1	0	-4.697894	-1.201835	2.755953
63	6	0	-5.266612	1.258894	1.902680
64	1	0	-4.039058	1.042535	3.637729
65	8	0	-2.873564	1.456605	1.980711
66	1	0	-3.095263	2.394283	1.931898
67	8	0	-5.307381	2.667749	2.039422
68	1	0	-5.258412	0.967853	0.841490
69	1	0	-6.130108	0.783401	2.388448
70	1	0	-5.977324	3.028163	1.453347
71	1	0	-2.511097	-2.024891	2.973211
72	1	0	4.784274	-0.669650	2.618451
73	1	0	3.413747	0.382611	3.051552
74	1	0	3.215998	-1.399175	3.051044

----

35 Cpl 4-CH3O-C6H4-Cl * N2 * 4 GL

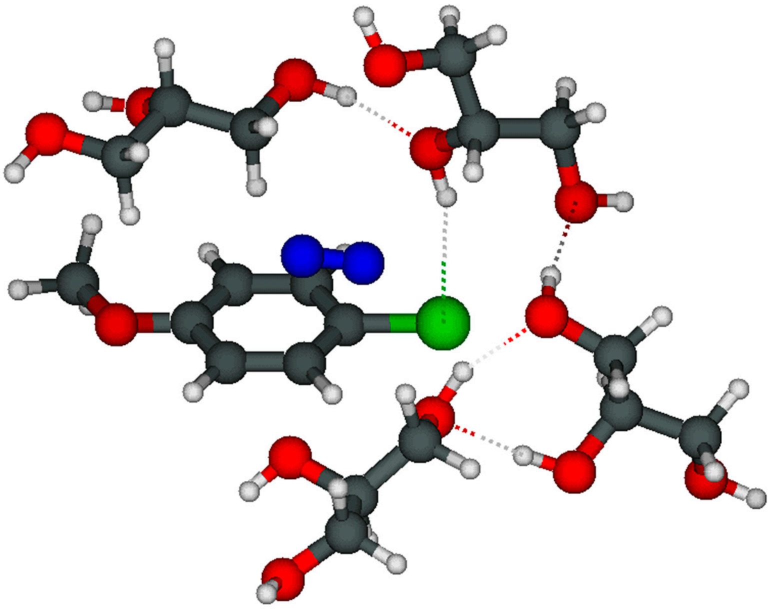

1	6	0	0.505444	0.230299	-1.907953
2	6	0	1.593701	-0.267045	-2.599359
3	6	0	2.788024	0.445788	-2.597089
4	6	0	2.876138	1.643659	-1.891225
5	6	0	1.760275	2.130035	-1.207513
6	6	0	0.568606	1.430176	-1.214271
7	1	0	1.522264	-1.209041	-3.133788
8	1	0	3.642160	0.042329	-3.127437
9	8	0	3.997165	2.395198	-1.799118
10	1	0	1.844302	3.063851	-0.658967
11	1	0	-0.294735	1.799637	-0.667458
12	17	0	-1.000815	-0.691037	-1.896731
13	7	0	0.908572	0.578562	1.993548
14	7	0	-0.013165	0.058379	1.713420
15	6	0	5.166400	1.930402	-2.449101
16	8	0	-2.313791	1.471116	0.248666
17	6	0	-2.630023	2.277015	1.363041
18	6	0	-2.148000	3.679183	1.106154
19	1	0	-3.717986	2.293172	1.542307
20	1	0	-2.142508	1.892236	2.271968
21	6	0	-2.627789	4.628779	2.178783
22	8	0	-0.733235	3.663635	1.059697
23	1	0	-2.548380	4.016159	0.133571
24	1	0	-0.447676	4.585046	1.079201
25	1	0	-2.375796	0.535172	0.522746
26	8	0	-2.064073	5.893030	1.881002
27	1	0	-3.725929	4.669876	2.180722
28	1	0	-2.288564	4.269917	3.162127
29	1	0	-2.146632	6.469414	2.644699
30	1	0	-0.081741	-2.372324	-0.475157
31	8	0	0.480961	-2.831263	0.165754
32	6	0	-0.320991	-3.280695	1.245660
33	6	0	-1.418670	-4.189914	0.747180
34	6	0	0.573883	-3.981660	2.232510
35	1	0	-0.783269	-2.412919	1.748012
36	1	0	-2.023301	-4.548733	1.590356
37	8	0	-2.207532	-3.417751	-0.149410
38	1	0	-0.982360	-5.054008	0.228425

39	1	0	-2.817371	-3.985349	-0.629603
40	1	0	-0.047080	-4.520039	2.963582
41	1	0	1.181782	-4.723684	1.692221
42	8	0	1.384649	-3.014516	2.863457
43	1	0	2.083083	-3.461994	3.347803
44	1	0	-2.751738	-1.810609	0.536363
45	8	0	-3.188640	-1.025383	0.915307
46	6	0	-4.564964	-1.121627	0.583885
47	6	0	-4.848908	-0.706035	-0.850399
48	1	0	-4.915091	-2.150234	0.750524
49	1	0	-5.113843	-0.461295	1.266598
50	6	0	-6.251479	-1.099111	-1.241294
51	8	0	-4.722606	0.687390	-1.037171
52	1	0	-4.143370	-1.247800	-1.510093
53	1	0	-3.845182	0.987766	-0.741428
54	8	0	-6.425241	-0.866514	-2.623328
55	1	0	-6.405773	-2.160717	-0.999458
56	1	0	-6.963334	-0.506435	-0.645309
57	1	0	-7.335799	-1.063875	-2.856382
58	8	0	3.276868	-2.677571	0.089561
59	6	0	3.349352	-1.358236	0.574899
60	6	0	4.785159	-0.931255	0.713153
61	1	0	2.835828	-0.660065	-0.107404
62	1	0	2.867320	-1.283054	1.562435
63	6	0	4.864780	0.477802	1.256934
64	1	0	5.296304	-1.612320	1.416758
65	8	0	5.421098	-0.998028	-0.551051
66	1	0	6.285468	-0.584012	-0.439922
67	8	0	6.238372	0.825031	1.299578
68	1	0	4.312724	1.156201	0.587586
69	1	0	4.409290	0.523205	2.256811
70	1	0	6.324293	1.777400	1.388132
71	1	0	2.331776	-2.902574	0.076786
72	1	0	5.950957	2.651368	-2.220569
73	1	0	5.453638	0.941782	-2.073154
74	1	0	5.024669	1.885244	-3.535469

----

36 4-CH3O-C6H4-Cl

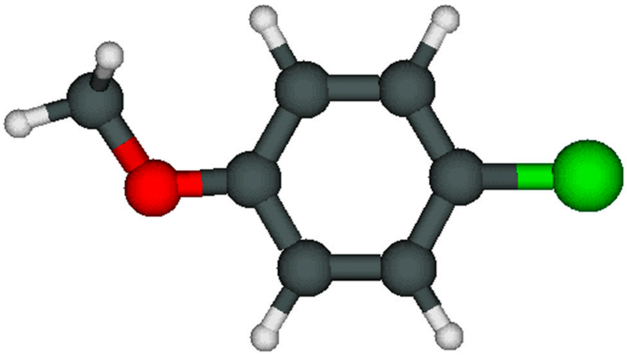

1	6	0	-0.010553	0.000000	-0.005306
2	6	0	0.002382	-0.000000	1.383857
3	6	0	1.213612	-0.000000	2.047996
4	6	0	2.413092	-0.000000	1.333439
5	6	0	2.387570	0.000000	-0.058522
6	6	0	1.167787	0.000000	-0.727181
7	1	0	-0.929933	-0.000000	1.938347
8	1	0	1.252078	-0.000000	3.132611

9	8	0	3.542355	-0.000000	2.077689
10	1	0	3.303144	0.000000	-0.637864
11	1	0	1.143044	0.000000	-1.811766
12	17	0	-1.547078	0.000000	-0.853120
13	6	0	4.785867	-0.000000	1.405876
14	1	0	5.553397	-0.000000	2.178932
15	1	0	4.903151	-0.894270	0.782686
16	1	0	4.903151	0.894270	0.782686

37 Cpl 4-CH3O-C6H4-N2(+) * Br(-) * 4 GL

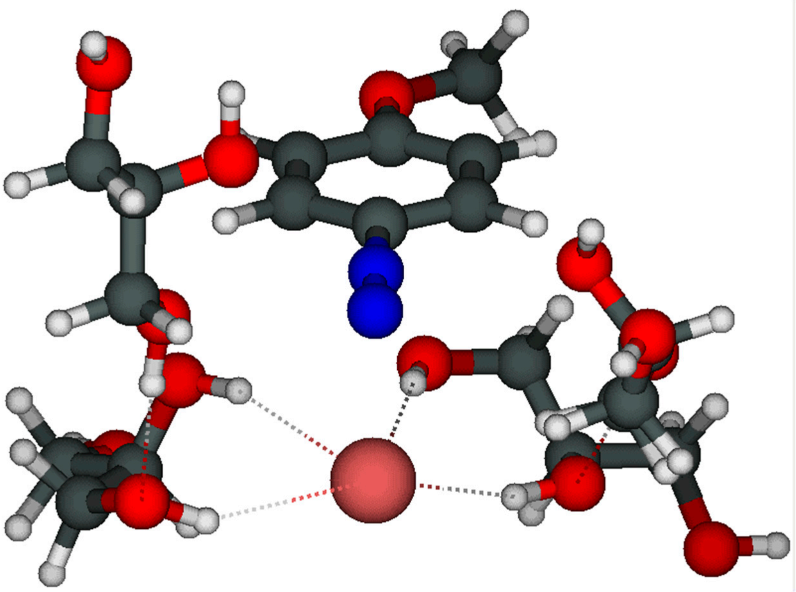

1	6	0	0.040555	-0.137522	-0.006618
2	6	0	0.071076	-0.161841	1.392920
3	6	0	1.294134	-0.080190	2.013563
4	6	0	2.464638	0.001357	1.242496
5	6	0	2.398614	0.034671	-0.162776
6	6	0	1.193643	-0.034118	-0.803330
7	1	0	-0.853242	-0.256162	1.955724
8	1	0	1.336616	-0.093277	3.095739
9	8	0	3.686224	0.049423	1.754381
10	1	0	3.325440	0.098187	-0.721672
11	1	0	1.093992	-0.041422	-1.884501
12	35	0	-0.622650	-3.640936	-0.643663
13	7	0	-1.169536	-0.273566	-0.618594
14	7	0	-2.160652	-0.400658	-1.088022
15	6	0	3.856854	0.002597	3.167717
16	8	0	-0.145739	-0.681831	-3.470278
17	6	0	-0.998456	0.105233	-4.262266
18	6	0	-0.693380	1.556446	-3.997348
19	1	0	-0.856114	-0.096219	-5.334669
20	1	0	-2.058897	-0.083588	-4.023057
21	6	0	-1.471317	2.463414	-4.921359
22	8	0	-1.013884	1.843941	-2.646544
23	1	0	0.385799	1.722999	-4.162585
24	1	0	-0.983677	2.804053	-2.554716
25	1	0	-0.207534	-1.627406	-3.707049
26	8	0	-1.166335	3.791427	-4.535809
27	1	0	-1.183356	2.272629	-5.964555

28	1	0	-2.547025	2.260746	-4.811821
29	1	0	-1.804504	4.393388	-4.926423
30	1	0	-0.441794	-4.466699	1.558308
31	8	0	-0.434340	-4.476759	2.532833
32	6	0	0.883644	-4.693174	3.023392
33	6	0	1.775136	-3.495609	2.750990
34	6	0	0.775410	-4.945459	4.505725
35	1	0	1.317113	-5.581973	2.537988
36	1	0	2.727979	-3.625617	3.276352
37	8	0	2.091549	-3.322921	1.390198
38	1	0	1.283212	-2.596141	3.163893
39	1	0	1.277566	-3.208199	0.871574
40	1	0	1.784998	-4.958328	4.940723
41	1	0	0.219630	-4.111865	4.965791
42	8	0	0.116238	-6.175219	4.715184
43	1	0	-0.013483	-6.299610	5.658774
44	1	0	-0.327242	-3.814321	-3.080968
45	8	0	-0.056599	-3.409478	-3.922824
46	6	0	1.253719	-3.826583	-4.263844
47	6	0	2.171245	-3.959153	-3.059479
48	1	0	1.221827	-4.784248	-4.802603
49	1	0	1.653853	-3.066628	-4.948153
50	6	0	3.574277	-4.267233	-3.515855
51	8	0	2.200253	-2.772855	-2.293296
52	1	0	1.819544	-4.797284	-2.432518
53	1	0	1.406422	-2.765442	-1.734220
54	8	0	4.335186	-4.691890	-2.404775
55	1	0	3.539906	-5.045660	-4.292694
56	1	0	3.999874	-3.357647	-3.966975
57	1	0	5.250461	-4.794575	-2.677074
58	8	0	-1.832588	-2.133214	3.172008
59	6	0	-2.914969	-2.317567	2.288382
60	6	0	-3.784527	-1.090198	2.292924
61	1	0	-2.556086	-2.501451	1.260475
62	1	0	-3.528474	-3.183665	2.584501
63	6	0	-5.041968	-1.305133	1.483732
64	1	0	-4.067488	-0.855599	3.333453
65	8	0	-3.043013	-0.008606	1.749428
66	1	0	-3.663934	0.718984	1.620617
67	8	0	-5.747556	-0.077674	1.500425
68	1	0	-4.767706	-1.586365	0.455199
69	1	0	-5.638673	-2.117058	1.921923
70	1	0	-6.430070	-0.090391	0.824956
71	1	0	-1.256338	-2.912536	3.069571
72	1	0	4.930238	0.042640	3.340459
73	1	0	3.376455	0.860782	3.646531
74	1	0	3.455533	-0.929420	3.576713

----

38 TS_SN 4-CH3O-C6H4-N2(+) * Br(-) * 4 GL

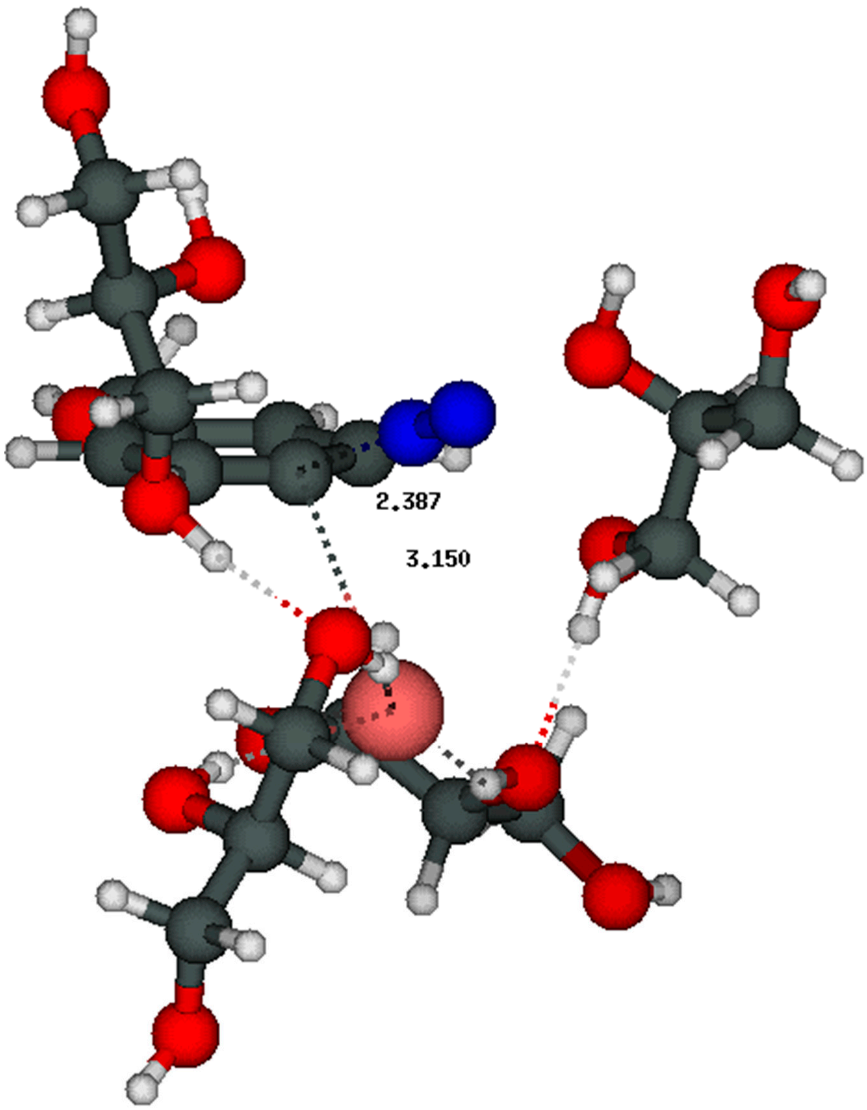

1	6	0	-0.031310	-0.093265	-0.011985
2	6	0	-0.037975	-0.063994	1.312543
3	6	0	1.311100	-0.055345	1.731511
4	6	0	2.356798	-0.063204	0.805414
5	6	0	2.095231	-0.055311	-0.573053
6	6	0	0.786159	-0.056630	-1.064614
7	1	0	-0.886733	-0.088299	1.997314
8	1	0	1.485953	-0.053973	2.801418
9	8	0	3.654211	-0.074610	1.144929
10	1	0	2.920709	-0.047740	-1.277837
11	1	0	0.489984	-0.024829	-2.116888
12	35	0	-1.835828	-2.584076	-0.689862
13	7	0	-2.114467	0.711822	-0.854510
14	7	0	-3.063995	1.090089	-1.244746
15	6	0	3.986937	-0.177186	2.519927
16	8	0	-0.555523	0.123172	-3.773269
17	6	0	-0.927095	1.392035	-4.263701
18	6	0	-0.009114	2.444774	-3.699531
19	1	0	-0.865820	1.415773	-5.362885
20	1	0	-1.962884	1.634738	-3.980953
21	6	0	-0.175134	3.763714	-4.417521
22	8	0	-0.292854	2.598243	-2.321034

23	1	0	1.034478	2.103915	-3.828795
24	1	0	0.184824	3.383431	-2.027168
25	1	0	-1.354272	-0.434858	-3.705222
26	8	0	0.672379	4.689854	-3.762931
27	1	0	0.096150	3.651317	-5.476343
28	1	0	-1.226610	4.082642	-4.356951
29	1	0	0.441378	5.582350	-4.031767
30	1	0	-1.737787	-3.739730	1.443375
31	8	0	-1.675356	-3.792992	2.412582
32	6	0	-0.438558	-4.377660	2.809259
33	6	0	0.738912	-3.509660	2.405179
34	6	0	-0.483690	-4.546854	4.306283
35	1	0	-0.333411	-5.367876	2.338687
36	1	0	1.655536	-3.913101	2.849504
37	8	0	0.953703	-3.469614	1.014673
38	1	0	0.587336	-2.496554	2.817706
39	1	0	0.193542	-3.061542	0.568387
40	1	0	0.509395	-4.861076	4.658252
41	1	0	-0.712066	-3.571842	4.767233
42	8	0	-1.465682	-5.506422	4.630320
43	1	0	-1.538494	-5.563560	5.586406
44	1	0	-3.010805	-1.677378	-2.690880
45	8	0	-2.938496	-1.257108	-3.564035
46	6	0	-3.066853	-2.234531	-4.582372
47	6	0	-2.333669	-3.526092	-4.257561
48	1	0	-4.126372	-2.452526	-4.775321
49	1	0	-2.641938	-1.787779	-5.491160
50	6	0	-2.355038	-4.437728	-5.457513
51	8	0	-0.989090	-3.282201	-3.900273
52	1	0	-2.853286	-4.033695	-3.426638
53	1	0	-0.967544	-3.037769	-2.961073
54	8	0	-1.963565	-5.733655	-5.057543
55	1	0	-3.367426	-4.444966	-5.888588
56	1	0	-1.668358	-4.028625	-6.214577
57	1	0	-1.890821	-6.290137	-5.837025
58	8	0	-2.239782	-1.184604	3.238639
59	6	0	-3.427808	-0.916231	2.525087
60	6	0	-3.769711	0.541880	2.667366
61	1	0	-3.297792	-1.153159	1.454543
62	1	0	-4.266583	-1.519016	2.906310
63	6	0	-5.094644	0.856588	2.012042
64	1	0	-3.836833	0.791269	3.740795
65	8	0	-2.734344	1.300468	2.067928
66	1	0	-3.059071	2.205777	1.990586
67	8	0	-5.271290	2.258125	2.108948
68	1	0	-5.067354	0.536804	0.959457
69	1	0	-5.903235	0.313628	2.520846
70	1	0	-5.977805	2.534116	1.519927
71	1	0	-1.991008	-2.107797	3.042506
72	1	0	5.073475	-0.221747	2.568240
73	1	0	3.635333	0.697089	3.078340
74	1	0	3.565054	-1.090330	2.955973

----

39 Cpl 4-CH3O-C6H4-Br * N2 * 4 GL

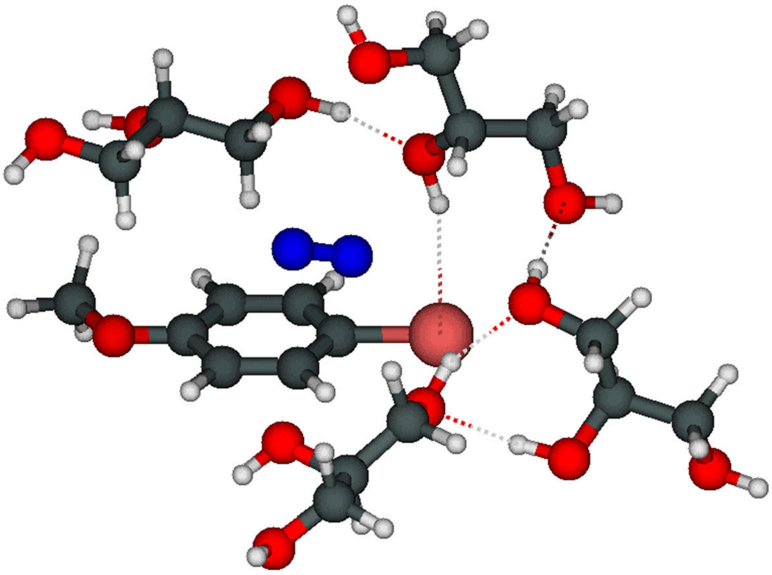

1	6	0	-0.665903	0.281590	1.918453
2	6	0	-1.776607	-0.211526	2.579480
3	6	0	-2.982150	0.478362	2.505701
4	6	0	-3.061510	1.651553	1.758666
5	6	0	-1.926656	2.137058	1.106561
6	6	0	-0.724407	1.459141	1.184296
7	1	0	-1.716970	-1.133916	3.148491
8	1	0	-3.850363	0.076225	3.013727
9	8	0	-4.190937	2.377901	1.596647
10	1	0	-2.003632	3.051295	0.524822
11	1	0	0.150776	1.834744	0.660254
12	35	0	0.979053	-0.689531	2.000030
13	7	0	-0.967689	0.630589	-2.077175
14	7	0	-0.074497	0.113913	-1.711162
15	6	0	-5.376267	1.915341	2.218690
16	8	0	2.223115	1.572145	-0.232404
17	6	0	2.485737	2.374292	-1.364061
18	6	0	1.980164	3.768058	-1.107344
19	1	0	3.567073	2.411141	-1.577234
20	1	0	1.979527	1.968704	-2.253475
21	6	0	2.410308	4.716656	-2.201623
22	8	0	0.567663	3.721843	-1.022558
23	1	0	2.398788	4.123602	-0.149214
24	1	0	0.261249	4.636530	-1.040582
25	1	0	2.270323	0.637868	-0.513623
26	8	0	1.827469	5.971464	-1.901088
27	1	0	3.506895	4.781038	-2.233254
28	1	0	2.052890	4.340467	-3.172077
29	1	0	1.889445	6.546178	-2.668003
30	1	0	0.101421	-2.362256	0.370046
31	8	0	-0.456594	-2.831774	-0.268301
32	6	0	0.333916	-3.226802	-1.377335
33	6	0	1.474470	-4.109135	-0.929137
34	6	0	-0.560842	-3.932944	-2.360596
35	1	0	0.752280	-2.332213	-1.869911
36	1	0	2.066314	-4.428870	-1.796750
37	8	0	2.263994	-3.332667	-0.036528
38	1	0	1.081140	-4.998704	-0.419152
39	1	0	2.900738	-3.892102	0.417642

40	1	0	0.059268	-4.440870	-3.113837
41	1	0	-1.139416	-4.700216	-1.823698
42	8	0	-1.407507	-2.974513	-2.957282
43	1	0	-2.111343	-3.429655	-3.426491
44	1	0	2.732844	-1.690811	-0.678569
45	8	0	3.100416	-0.872790	-1.062515
46	6	0	4.507146	-0.926138	-0.891978
47	6	0	4.935161	-0.578506	0.524009
48	1	0	4.877193	-1.926394	-1.158315
49	1	0	4.948606	-0.203372	-1.589500
50	6	0	6.392362	-0.911526	0.725091
51	8	0	4.761032	0.792333	0.811868
52	1	0	4.340668	-1.197861	1.224166
53	1	0	3.848005	1.065769	0.615902
54	8	0	6.719836	-0.741613	2.088278
55	1	0	6.569077	-1.948708	0.404962
56	1	0	6.996455	-0.249393	0.084932
57	1	0	7.661836	-0.892250	2.200109
58	8	0	-3.248379	-2.682366	-0.159465
59	6	0	-3.359945	-1.372110	-0.661537
60	6	0	-4.808039	-1.011492	-0.853725
61	1	0	-2.899444	-0.644941	0.028932
62	1	0	-2.848856	-1.287854	-1.633417
63	6	0	-4.933703	0.386042	-1.418381
64	1	0	-5.261889	-1.723595	-1.565953
65	8	0	-5.484758	-1.094740	0.388130
66	1	0	-6.359067	-0.712838	0.244467
67	8	0	-6.319591	0.672090	-1.502448
68	1	0	-4.429614	1.097403	-0.745363
69	1	0	-4.454793	0.437679	-2.406830
70	1	0	-6.444264	1.618033	-1.611166
71	1	0	-2.298455	-2.887246	-0.158911
72	1	0	-6.163294	2.613897	1.935576
73	1	0	-5.633517	0.909260	1.868106
74	1	0	-5.274933	1.910312	3.310449

---

40 4-CH3O-C6H4-Br

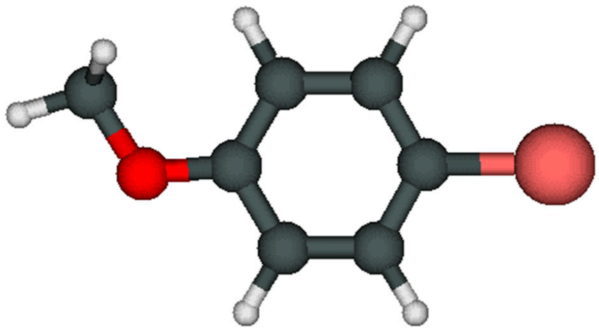

1	6	0	-0.001609	0.000000	-0.001352
2	6	0	0.000363	0.000000	1.389031
3	6	0	1.204965	0.000000	2.065544
4	6	0	2.411697	-0.000000	1.362853
5	6	0	2.399455	-0.000000	-0.029479
6	6	0	1.185986	-0.000000	-0.709916
7	1	0	-0.934420	0.000000	1.939715
8	1	0	1.232783	0.000000	3.150611
9	8	0	3.533757	-0.000000	2.116497
10	1	0	3.320605	-0.000000	-0.599986

11	1	0	1.177780	-0.000000	-1.794903
12	35	0	-1.660436	0.000000	-0.937042
13	6	0	4.783500	-0.000000	1.454903
14	1	0	5.544421	-0.000000	2.234371
15	1	0	4.905355	-0.894180	0.832702
16	1	0	4.905355	0.894180	0.832702

----

41 TS_Solv 4-CH3O-C6H4-N2(+) * 3 GL

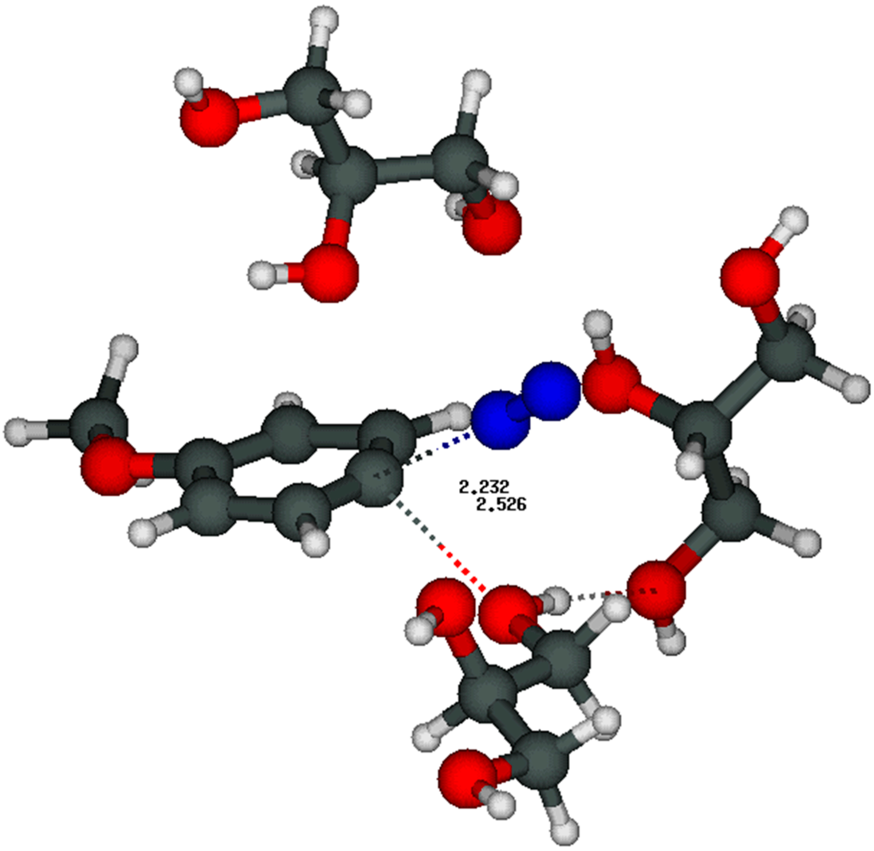

1	6	0	0.089609	0.023891	-0.041028
2	6	0	0.098864	0.042332	1.294563
3	6	0	1.429491	0.052779	1.715859
4	6	0	2.487119	0.027303	0.794261
5	6	0	2.239028	0.010861	-0.581135
6	6	0	0.920164	-0.001829	-1.075840
7	1	0	-0.778992	0.080246	1.930914
8	1	0	1.645681	0.087436	2.778739
9	8	0	3.715891	0.031022	1.333068
10	1	0	3.040158	0.010046	-1.310690
11	1	0	0.630253	-0.014766	-2.123085
12	7	0	-1.833341	-0.913462	-0.677213
13	7	0	-2.773448	-1.457758	-0.807027
14	6	0	4.835163	-0.001464	0.462696
15	8	0	-1.536915	1.874643	-0.596189
16	6	0	-2.872917	2.150365	-0.238864
17	6	0	-3.022681	2.064857	1.256768
18	1	0	-3.157242	3.160570	-0.570144
19	1	0	-3.572214	1.438046	-0.708802
20	1	0	-1.437170	2.044832	-1.552956
21	6	0	-4.363003	2.608222	1.698181
22	8	0	-2.897271	0.709154	1.658412

23	1	0	-2.221545	2.662493	1.725511
24	8	0	-4.457984	2.361308	3.088492
25	1	0	-4.423449	3.681888	1.474935
26	1	0	-5.165884	2.090518	1.152455
27	1	0	-3.282262	0.653582	2.543431
28	1	0	-5.371034	2.455755	3.370911
29	8	0	-1.509208	2.494862	-3.290555
30	6	0	-1.963205	1.700498	-4.375123
31	6	0	-2.189109	0.300726	-3.872566
32	1	0	-2.901811	2.100161	-4.781557
33	1	0	-1.213926	1.679518	-5.178537
34	6	0	-2.866730	-0.556345	-4.916085
35	8	0	-0.940984	-0.245701	-3.508815
36	1	0	-2.852668	0.353777	-2.988438
37	8	0	-3.018561	-1.841806	-4.346799
38	1	0	-3.838816	-0.118833	-5.184270
39	1	0	-2.239196	-0.594415	-5.819006
40	1	0	-1.040250	-1.195180	-3.319007
41	1	0	-3.252488	-2.470312	-5.034213
42	1	0	-1.293205	3.379377	-3.598377
43	8	0	-0.135589	-2.710963	-2.514254
44	6	0	-0.428940	-3.911277	-1.824402
45	6	0	0.364608	-4.059017	-0.548803
46	1	0	-1.498577	-3.880212	-1.587703
47	1	0	-0.252639	-4.785356	-2.466787
48	6	0	0.162062	-5.427558	0.061215
49	1	0	1.439780	-3.940636	-0.782533
50	8	0	-0.037835	-3.051809	0.356680
51	1	0	0.357675	-3.271282	1.209209
52	8	0	0.888596	-5.437294	1.275482
53	1	0	-0.911056	-5.589568	0.242898
54	1	0	0.521556	-6.204098	-0.628222
55	1	0	0.615542	-6.188505	1.807845
56	1	0	0.784705	-2.731751	-2.797219
57	1	0	5.716821	0.000837	1.101105
58	1	0	4.832235	-0.910587	-0.148637
59	1	0	4.855377	0.881303	-0.185871

----

42 Cpl 4-CH3O-C6H4-O-C3H7O2 * H(+)GL * GL * N2

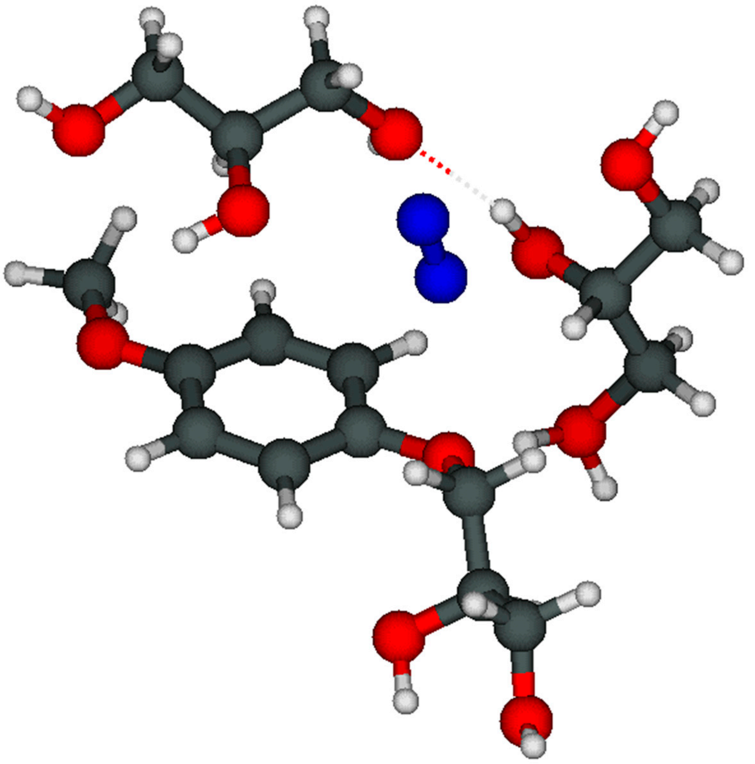

1	6	0	0.088178	0.078901	-0.035012
2	6	0	0.099259	0.155918	1.350789
3	6	0	1.312879	0.154210	2.015249
4	6	0	2.513852	0.099201	1.310733
5	6	0	2.489172	0.059306	-0.079866
6	6	0	1.271756	0.034247	-0.749187
7	1	0	-0.823741	0.247018	1.910663
8	1	0	1.345811	0.207949	3.098964
9	8	0	3.643044	0.083535	2.061234
10	1	0	3.405685	0.028787	-0.658560
11	1	0	1.248851	-0.049904	-1.831293
12	7	0	-0.792117	-3.258186	-0.499582
13	7	0	-0.589871	-4.293783	-0.209946
14	6	0	4.887651	0.106800	1.393757
15	8	0	-1.095558	0.070019	-0.775377
16	6	0	-2.328289	-0.391830	-0.200505
17	6	0	-3.261142	0.747849	0.113719
18	1	0	-2.793777	-1.049946	-0.944057
19	1	0	-2.110457	-0.989414	0.689526
20	1	0	-1.093262	0.336232	-2.183067
21	6	0	-4.628527	0.213708	0.483309
22	8	0	-2.720028	1.501592	1.174481
23	1	0	-3.367024	1.381216	-0.785261
24	8	0	-5.409436	1.339778	0.827361
25	1	0	-5.061243	-0.334573	-0.365002
26	1	0	-4.532746	-0.476043	1.334820
27	1	0	-3.418032	2.094260	1.480406
28	1	0	-6.191994	1.054815	1.306130
29	8	0	-1.078958	0.540063	-3.217011
30	6	0	-1.435092	-0.559840	-4.129544
31	6	0	-0.969264	-1.845155	-3.507317
32	1	0	-2.515806	-0.547962	-4.278745
33	1	0	-0.909483	-0.345603	-5.061711

34	6	0	-1.327010	-3.019687	-4.393067
35	8	0	0.412503	-1.739430	-3.306310
36	1	0	-1.488825	-1.987934	-2.544107
37	8	0	-0.920435	-4.172435	-3.689584
38	1	0	-2.408582	-3.027449	-4.592119
39	1	0	-0.795111	-2.923066	-5.350555
40	1	0	0.735793	-2.507190	-2.803088
41	1	0	-0.937987	-4.928570	-4.281812
42	1	0	-1.513661	1.380383	-3.429204
43	8	0	2.222016	-3.275575	-1.884543
44	6	0	2.813228	-4.145786	-0.934708
45	6	0	3.166816	-3.453214	0.360553
46	1	0	2.081908	-4.935729	-0.733390
47	1	0	3.711920	-4.617234	-1.356655
48	6	0	4.122237	-4.280176	1.190282
49	1	0	3.668124	-2.495187	0.120047
50	8	0	1.979321	-3.199223	1.077538
51	1	0	2.235848	-2.796635	1.915878
52	8	0	4.354550	-3.550665	2.380047
53	1	0	3.666544	-5.258287	1.406784
54	1	0	5.055572	-4.445125	0.633338
55	1	0	4.783581	-4.117728	3.025539
56	1	0	2.860960	-2.589285	-2.105938
57	1	0	5.653319	0.122189	2.168793
58	1	0	5.029650	-0.787037	0.773392
59	1	0	4.988503	1.001597	0.768037

----

43 4-CH3O-C6H4-O-C3H7O2

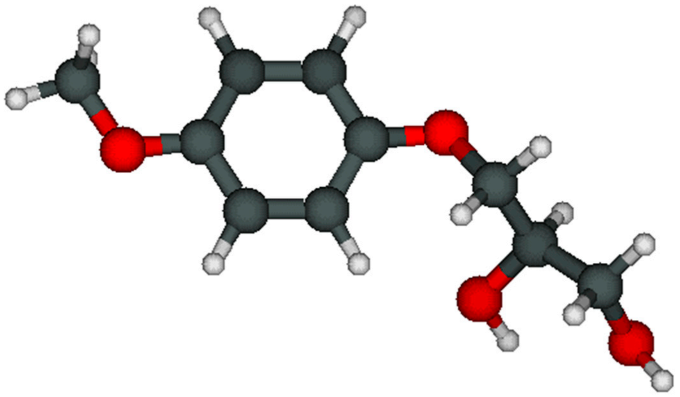

1	6	0	0.045410	0.227651	-0.062085
2	6	0	0.074175	0.345586	1.326466
3	6	0	1.280534	0.254129	2.000294
4	6	0	2.474409	0.061569	1.309535
5	6	0	2.447669	-0.040039	-0.078469
6	6	0	1.234747	0.037860	-0.752157
7	1	0	-0.838905	0.539224	1.877680
8	1	0	1.314603	0.346427	3.081824
9	8	0	3.601497	-0.009188	2.067339
10	1	0	3.357079	-0.188712	-0.649277
11	1	0	1.202973	-0.051568	-1.833792
12	6	0	4.832113	-0.195217	1.403302
13	8	0	-1.087902	0.323302	-0.818837
14	6	0	-2.339146	-0.053798	-0.270974
15	6	0	-3.219262	1.138423	0.006785
16	1	0	-2.205308	-0.648256	0.641957

17	1	0	-2.831094	-0.689309	-1.017333
18	6	0	-4.634371	0.700352	0.310361
19	8	0	-2.685647	1.862103	1.097666
20	1	0	-3.356645	2.504560	1.358933
21	1	0	-3.236097	1.776573	-0.893654
22	8	0	-5.359579	1.876300	0.616376
23	1	0	-6.188560	1.641666	1.040691
24	1	0	-5.061653	0.179534	-0.557828
25	1	0	-4.628045	0.008542	1.165946
26	1	0	5.601070	-0.218759	2.175174
27	1	0	4.851131	-1.142068	0.849749
28	1	0	5.043914	0.629190	0.711316

Pictures and **Cartesian coordinates** for the intramolecular fluorination of **1a** (see **Table 5**).

44 TS_SNi 4-NO2-C6H4-N2(+) * BF4(-)

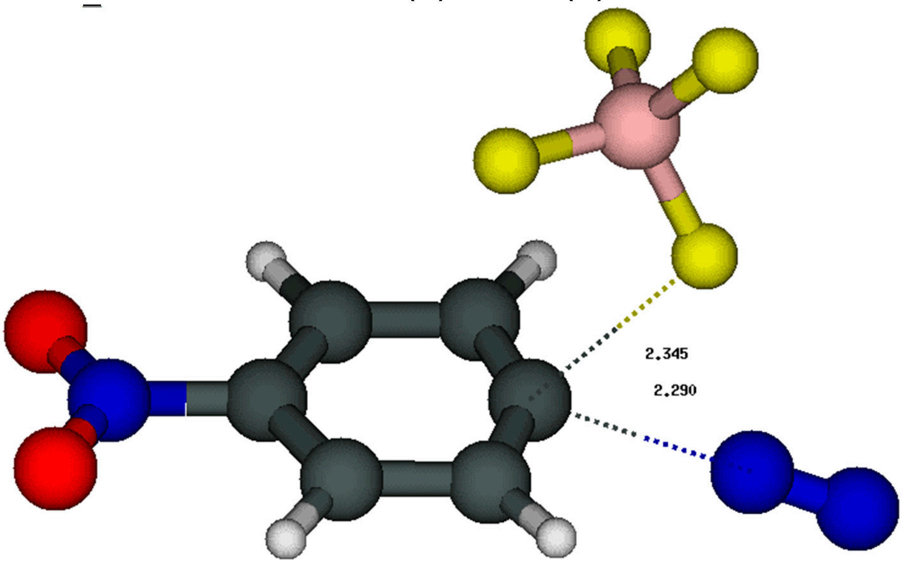

1	6	0	0.005080	0.004401	-0.015877
2	6	0	-0.007159	-0.007630	1.316879
3	6	0	1.327521	-0.006441	1.744350
4	6	0	2.350900	0.011761	0.808578
5	6	0	2.138237	0.033504	-0.561330
6	6	0	0.828715	0.034363	-1.063093
7	1	0	-0.864172	-0.039738	1.974863
8	1	0	1.539558	-0.025725	2.807469
9	7	0	3.744670	0.009303	1.298399
10	1	0	2.968261	0.048418	-1.258800
11	1	0	0.571385	0.034746	-2.112956
12	9	0	-1.493560	-1.718286	-0.548952
13	5	0	-0.727989	-2.915841	-0.757074
14	9	0	-0.151940	-2.845309	-2.023595
15	9	0	0.266482	-2.967676	0.220968
16	9	0	-1.578569	-4.006430	-0.656643
17	7	0	-2.004695	0.860479	-0.704515
18	7	0	-2.959891	1.284294	-1.024853
19	8	0	4.625979	0.035998	0.468235
20	8	0	3.912655	-0.019235	2.497415

45 Cpl 4-NO2-C6H4-F * BF3 * N2

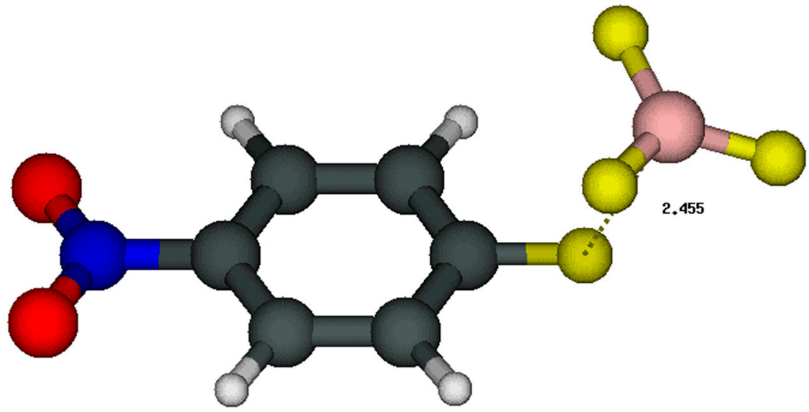

1	6	0	0.000000	0.000000	0.000000
2	6	0	0.000000	0.000000	1.381173
3	6	0	1.220580	0.000000	2.031262
4	6	0	2.382243	-0.001191	1.272494
5	6	0	2.367368	-0.004592	-0.114798
6	6	0	1.148243	-0.005042	-0.767442
7	1	0	-0.937331	-0.001655	1.924903
8	1	0	1.278057	0.000002	3.112430
9	7	0	3.677073	-0.002083	1.963569
10	1	0	3.297755	-0.007917	-0.668429
11	1	0	1.078018	-0.010779	-1.848819
12	9	0	-1.187551	-0.008135	-0.636459
13	5	0	-1.900672	-2.272065	-1.265743
14	9	0	-0.851696	-2.377925	-2.046728
15	9	0	-1.818323	-2.650546	-0.013040
16	9	0	-3.040040	-1.849399	-1.754087
17	7	0	-2.105832	0.408480	-3.805930
18	7	0	-1.019454	0.281341	-3.771999
19	8	0	4.685265	0.013450	1.284027
20	8	0	3.673997	-0.018419	3.179342

46 Cpl 4-NO2-C6H4-F * BF3

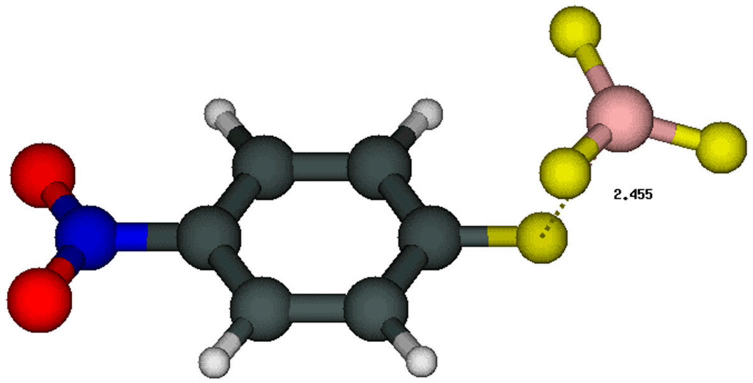

1	6	0	0.015018	0.039538	-0.017218
2	6	0	0.007357	0.013347	1.363860
3	6	0	1.223838	-0.006237	2.021109
4	6	0	2.390018	-0.001182	1.269417
5	6	0	2.383289	0.021872	-0.117819
6	6	0	1.168193	0.042466	-0.777658
7	1	0	-0.933169	0.007060	1.902183
8	1	0	1.274443	-0.026294	3.102426
9	7	0	3.680093	-0.025094	1.968648
10	1	0	3.316993	0.022722	-0.665896
11	1	0	1.105724	0.059229	-1.859369
12	9	0	-1.168968	0.052414	-0.658527
13	5	0	-2.149882	-2.172223	-0.998143
14	9	0	-1.125619	-2.521534	-1.739283
15	9	0	-2.115382	-2.380858	0.296381
16	9	0	-3.228599	-1.682644	-1.556622
17	8	0	4.692893	-0.029190	1.295788
18	8	0	3.668862	-0.039804	3.184454

Physical and NMR data of aryl halides 2, 4, 5.

1-Bromo-4-nitrobenzene (2a): 290 mg (72% yield; Method A); 330 mg (82% yield; Method B). Pale yellow solid, mp 124–125°C (crystallized from MeOH. Lit.[1] 123–125°C). ^{1}H-NMR ($CDCl_3$, 600 MHz): 8.09 (d, 2H, J = 8.4 Hz), 7.68 (d, 2H, J = 8.4 Hz). ^{13}C-NMR ($CDCl_3$, 150 MHz): 147.1, 132.7, 130.1, 125.1. MS: *m/z* 201 (M^+, 100), 203 (M^++ 2, 100).

4-Bromobenzonitrile (2b): 256 mg (70% yield; Method A); 290 mg (82% yield; Method B). White solid, mp 113–114°C (crystallized from MeOH. Lit.[2] 111–112°C). ^{1}H-NMR ($CDCl_3$, 600 MHz): 7.62 (d, 2H, J = 8.4 Hz), 7.51 (d, 2H, J = 8.4 Hz). ^{13}C-NMR ($CDCl_3$, 150 MHz): 133.5, 132.7, 128.1, 118.4, 111.3. MS: *m/z* 181 (M^+, 100), 183 (M^++ 2, 100).

1,4-Dibromobenzene (2c): 317 mg (67% yield; Method A); 318 mg (67% yield; Method B). Grey solid, mp 86–87°C (crystallized from MeOH. Lit.[3] 83–86°C). ^{1}H-NMR ($CDCl_3$, 600 MHz): 7.33 (s, 4H). ^{13}C-NMR ($CDCl_3$, 150 MHz): 132.2, 121.1. MS: *m/z* 234 (M^+, 48), 236 (M^++ 2, 100), 238 (M^++ 4, 49).

Methyl 4-bromobenzoate (2d): 278 mg (70% yield; Method A); 272 mg (63% yield; Method B). Yellow solid, mp 74–75°C (crystallized from MeOH. Lit.[4] 75–76°C) ^{1}H-NMR ($CDCl_3$, 600 MHz): 7.88 (d, 2H, J = 8.4 Hz), 7.56 (d, 2H, J = 8.4 Hz), 3.89 (s, 3H). ^{13}C-NMR ($CDCl_3$, 150 MHz): 166.4, 131.8, 131.2, 129.1, 128.1, 52.6. MS: *m/z* 214 (M^+, 80), 216 (M^++ 2, 75).

4-Bromotoluene (2e): 68 mg (20% yield; Method A); 108 mg (32% yield; Method B). Pale yellow waxy solid. ^{1}H-NMR ($CDCl_3$, 600 MHz): 7.38 (d, 2H, J = 7.8 Hz), 7.02 (d, 2H, J = 7.8 Hz), 2.33 (s, 3H). ^{13}C-NMR ($CDCl_3$, 150 MHz): 135.5, 131.3, 130.6, 119.6, 21.1. MS: m/z 170 (M^+, 100), 172 (M^++ 2, 100).

1-Bromo-4-methoxybenzene (2f): 95 mg (25% yield; Method A); 112 mg (30% yield; Method B). Viscous pale yellow oil. ^{1}H-NMR ($CDCl_3$, 600 MHz): 7.41 (d, 2H, J = 7.8 Hz), 6.76 (d, 2H, J = 8.4 Hz), 3.80 (s, 3H). ^{13}C-NMR ($CDCl_3$, 150 MHz): 158.9, 132.3, 114.1, 112.0, 56.0. MS: m/z 186 (M^+, 100), 188 (M^++ 2, 100).

1-Bromo-2-nitrobenzene (2g): 215 mg (53% yield; Method A); 179 mg (44% yield; Method B). Pale yellow solid, mp 41–42°C (crystallized from MeOH. Lit.[5] 40–42°C). ^{1}H-NMR ($CDCl_3$, 600 MHz): 7.84–7.81 (m, 1H), 7.74–7.72 (m, 1H), 7.47–7.41 (m, 2H). ^{13}C-NMR ($CDCl_3$, 150 MHz): 150.5, 135.4, 133.3, 128.4, 125.7, 114.4. MS: *m/z* 201 (M^+, 100), 203 (M^++ 2, 100).

1-Bromo-3-nitrobenzene (2h): 280 mg (69% yield; Method A); 275 mg (68% yield; Method B). Pale yellow solid, mp 51–52°C (crystallized from MeOH. Lit.[6] 52–53°C). ^{1}H-NMR ($CDCl_3$, 600 MHz): 8.34–8.33 (m, 1H), 8.15–8.13 (m, 1H), 7.82–7.80 (m, 1H), 7.44–7.41 (m, 1H). ^{13}C-NMR ($CDCl_3$, 150 MHz): 148.8, 137.7, 130.7, 126.8, 123.0, 122.2. MS: *m/z* 201 (M^+, 100), 203 (M^++ 2, 100).

1-Bromo-2,4-dinitrobenzene (2i): 450 mg (91% yield; Method A); 451 mg (92% yield; Method B). Pale brown solid, mp 72–73°C (crystallized from MeOH. Lit.[7] 72–74°C). ^{1}H-NMR ($CDCl_3$, 600 MHz): 8.68 (s, 1H), 8.27 (dd, 1H, J_1 = 8.4 Hz, J_2 = 2.4 Hz), 7.98 (d, 1H, , J = 9.0 Hz). ^{13}C-NMR ($CDCl_3$, 150 MHz): 149.9, 147.0, 136.6, 127.2, 122.0, 121.0. MS: *m/z* 246 (M^+, 100), 248 (M^++ 2, 100).

2-Bromo-5-nitrobenzonitrile (2j): 380 mg (85% yield; Method A); 365 mg (80% yield; Method B). Pale brown solid, mp 122–123°C (crystallized from MeOH. Lit.[8] 121°C). ^{1}H-NMR ($CDCl_3$, 600 MHz): 8.51 (d, 1H, J = 2.4 Hz), 8.29 (dd, 1H, J_1 = 9.0 Hz, J_2 = 2.4 Hz), 7.91 (d, 1H, J = 8.4 Hz). ^{13}C-NMR ($CDCl_3$, 150 MHz): 147.1, 134.7, 132.8, 129.3, 128.2, 117.6, 115.3. MS: *m/z* 226 (M^+, 100), 228 (M^++ 2, 100).

1-Bromo-2-nitro-4-methoxybenzene (2k): 161 mg (35% yield; Method A); 185 mg (40% yield; Method B). Pale brown waxy solid. ^{1}H-NMR ($CDCl_3$, 600 MHz): 7.57 (d, 1H, J = 9.0 Hz), 7.35 (d, 1H, J = 3.0 Hz), 6.96 (dd, 1H, J_1 = 9.0 Hz, J_2 = 3.0 Hz). ^{13}C-NMR ($CDCl_3$, 150 MHz): 159.3, 150.2, 135.7, 120.0, 111.3, 104.9, 56.2. MS: *m/z* 231 (M^+, 100), 233 (M^++ 2, 100).

Methyl-3-bromothiophene-2-carboxylate (2l): 155 mg (35% yield; Method A); 179 mg (40% yield; Method B). Pale grey solid, mp 45–46°C (crystallyzed from MeOH. Lit.[9] 46–48°C). ^{1}H-NMR (CDCl$_3$, 600 MHz): 7.43 (d, 1H, *J* = 5.4 Hz), 7.06 (d, 1H, *J* = 5.4 Hz), 3.88 (s, 3H). ^{13}C-NMR (CDCl$_3$, 150 MHz): 161.3, 133.0, 131.6, 127.8, 117.4, 1, 52.2. MS: *m/z* 220 (M$^+$, 45), 222 (M$^+$+ 2, 45).

1-Chloro-4-nitrobenzene (4a): 290 mg (72% yield; Method A); 330 mg (82% yield; Method B). Pale yellow solid, mp 83–84°C (crystallized from MeOH. Lit.[10] 81–83°C). ^{1}H-NMR (CDCl$_3$, 600 MHz): 8.09 (d, 2H, *J* = 7.8 Hz), 7.55 (d, 2H, *J* = 7.8 Hz). ^{13}C-NMR (CDCl$_3$, 150 MHz): 146.2, 140.5, 129.0, 126.1. MS: *m/z* 157 (M$^+$, 100).

1-Chloro-2,4-dinitrobenzene (4i): 404 mg (100% yield; Method A); 404 mg (100% yield; Method B). White solid mp 52–53°C (crystallized from MeOH. Lit.[11] 52–53°C). ^{1}H-NMR (CDCl$_3$, 600 MHz): 8.72 (d, 2H, *J* = 2.4 Hz), 8.38 (dd, 1H, J_1 = 9.0 Hz, J_2 = 3.0 Hz), 7.80 (d, 2H, *J* = 9.0 Hz), ^{13}C-NMR (CDCl$_3$, 150 MHz): 147.8, 146.4, 134.0, 133.3, 127.4, 121.2. MS: *m/z* 202 (M$^+$, 100).

2-Chloro-4-nitrobenzonitrile (4j): 344 mg (94% yield; Method A); 337 mg (92% yield; Method B). White solid, mp 110–111°C (crystallized from MeOH. Lit.[12] 107–109°C). ^{1}H-NMR (CDCl$_3$, 600 MHz): 8.53 (d, 2H, *J* = 2.4 Hz), 8.38 (dd, 1H, J_1 = 9.0 Hz, J_2 = 2.4 Hz), 7.73 (d, 2H, *J* = 9.0 Hz). ^{13}C-NMR (CDCl$_3$, 150 MHz): 146.4, 143.6, 131.4, 129.1, 128.4, 114.9, 114.0. MS: *m/z* 182 (M$^+$, 100).

1-Chloro-4-fluorobenzene (4m): 91 mg (35% yield; Method A); 88 mg (33% yield; Method B). Pale brown oil. ^{1}H-NMR (CDCl$_3$, 600 MHz): 7.48–7.47 (m, 2H), 7.13–7.09 (m, 2H).^{13}C-NMR (CDCl$_3$, 150 MHz): 162.5 (d, *J* = 244.5 Hz), 130.0 (d, *J* = 8.2 Hz), 128.6 (d, *J* = 7.8 Hz), 115.8 (d, *J* = 21.4 Hz). ^{19}F-NMR (CDCl$_3$, 564.5 MHz): -116.5. MS: *m/z* 130 (M$^+$, 100).

1-Iodo-4-nitrobenzene (5a): 498 mg (100% yield). Pale yellow solid, mp 171–172°C (crystallized from MeOH. Lit.[13] 170–172°C). ^{1}H-NMR (CDCl$_3$, 600 MHz): 7.93 (d, 2H, *J* = 9.0 Hz), 7.89 (d, 2H, *J* = 9.0 (Hz). ^{13}C-NMR (CDCl$_3$, 150 MHz): 147.9, 138.9, 124.9, 102.8. MS: *m/z* 249 (M$^+$, 100).

4-Iodotoluene (5e): 436 mg (100% yield). White waxy solid. ^{1}H-NMR (CDCl$_3$, 600 MHz): 7.55 (d, 2H, *J* = 9.0 Hz), 6.92 (d, 2H, *J* = 9.0 Hz), 2.29 (s, 3H). ^{13}C-NMR (CDCl$_3$, 150 MHz): 137.5, 137.3, 131.3, 90.3, 21.2. MS: m/z 218 (M$^+$, 100).

1-Iodo-2-nitrobenzene (5g): 498 mg (100% yield). Pale orange solid, mp 51–52°C (crystallized from MeOH. Lit.[14] 50–52°C). ^{1}H-NMR (CDCl$_3$, 600 MHz): 7.99–7.98 (m, 1H), 7.81–7.79 (m, 1H), 7.47–7.44 (m, 1H), 7.26–7.23 (m, 1H). ^{13}C-NMR (CDCl$_3$, 150 MHz): 153.0, 142.1, 133.8, 129.3, 125.5, 86.3. MS: *m/z* 249 (M$^+$, 100).

1-Iodo-2,4-dinitrobenzene (5i): 587 mg (100% yield). White solid, mp 89–90°C (crystallized from MeOH. Lit.[13] 88–89°C). ^{1}H-NMR (CDCl$_3$, 600 MHz): 8.62 (d, 2H, *J* = 2.4 Hz), 8.30 (d, 2H, *J* = 9.0 Hz), 8.09 (dd, 1H, J_1 = 8.4 Hz, J_2 = 2.4 Hz). ^{13}C-NMR (CDCl$_3$, 150 MHz): 153.2, 148.1, 143.6, 127.1, 120.5, 95.1. MS: *m/z* 294 (M$^+$, 100).

1-Iodo-2-nitro-4-methoxybenzene (5k): 555 mg (100% yield). White solid, mp 59–60°C (crystallized from MeOH. Lit.[15] 61–62°C). ^{1}H-NMR (CDCl$_3$, 600 MHz): 7.78 (d, 1H, *J* = 8.4 Hz), 7.35 (d, 1H, *J* = 3.0 Hz), 6.81 (dd, 1H, J_1 = 8.4 Hz, J_2 = 2.4 Hz). ^{13}C-NMR (CDCl$_3$, 150 MHz): 160.7, 153.5, 142.1, 120.4, 111.2, 74.5, 56.. MS: *m/z* 279 (M$^+$, 100).

References:

1. M. Zarei, E. Noroozizadeh, A. R. Moosavi-Zare, M. A. Zolfigol, *J. Org. Chem.,* **2018**, *83*, 3645−3650.
2. C. Zhu, F. Chen, C. Liu, H. Zeng, Z. Yang, W. Wu, H. Jiang, *J. Org. Chem.,* **2018**, *83*, 14713−14722.
3. S. Mukhopadhyay, S. Batra, *Chem. Eur. J.,* **2018**, *24*, 14622−14626.
4. H.-L. Xin, B. Pang, J. Choi, W. Akkad, H. Morimoto, T. Ohshima, *J. Org. Chem.,* **2020**, *85*, 11592−11606.
5. T. Li, X. Cui, L. Sun, C. Li, *RSC Adv.,* **2014**, *4*, 33599 −33606.
6. H. Shao, D. W. Foley, S. Huang, A. Y. Abbas, F. Lam, P. Gershkovich, T. D. Bradshaw, C. Pepper, P. M. Fischer, S. Wang, *Eur. J. Med. Chem.*, **2021**, *214*, 113244.
7. U. Lerch, J. G. Moffatt, *J. Org. Chem.*, **1971**, *36*, 3861−3869.
8. H. P. Baudet, *Recl. Trav. Chim. Pays-Bas*, **1924**, *43*, 707−726.
9. J. Gianni, V. Pirovano, G. Abbiati, *Org. Biomol. Chem.*, **2018,** *16*, 3213−3219.
10. J. Liu, J. Li, J. Ren, B.-B. Zeng, *Tetrahedron Lett*., **2014**, *55*, 1581−1584.
11. D. Ramananda, J. Uchil, *J. Mol. Struct.,* **1994**, *319*, 193−196.
12. J.F.K. Wilshire*, Aust. J. Chem.,* **1967**, *20*, 1663−1670.
13. N. Sloan, S. K. Luthra, G. McRobbie, S. L. Pimlott, A. Sutherland, *Chem. Commun.*, **2017**, *53*, 11008−11011.
14. S. Gupta, A. Ansari, K. V. Sashidhara, *Tetrahedron Lett.,* **2019**, *60*, 151076.
15. Z. Fu, Z. Li, Y. Song, R. Yang, Y. Liu, H. Cai, *J. Org. Chem.*, **2016**, *81*, 2794−2803.

NMR spectra of aryl halides 2, 4, 5

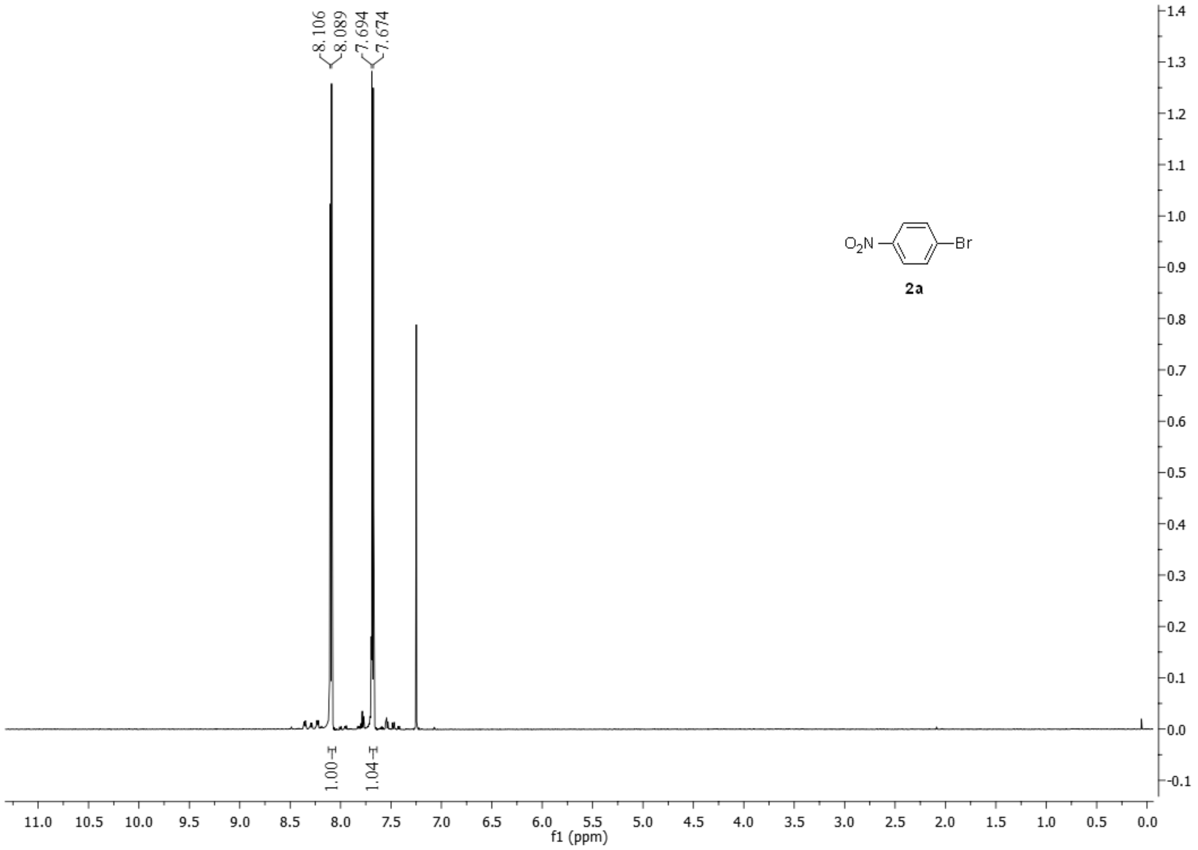

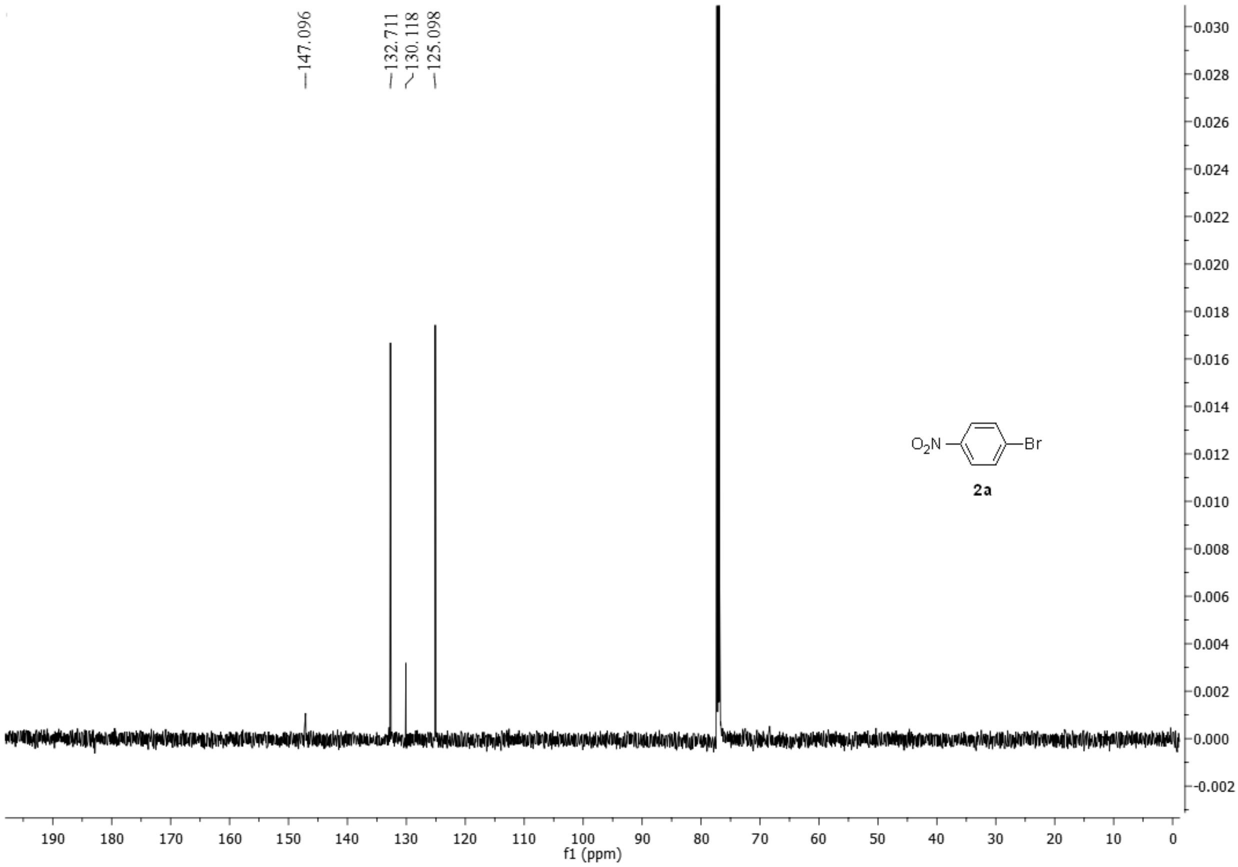

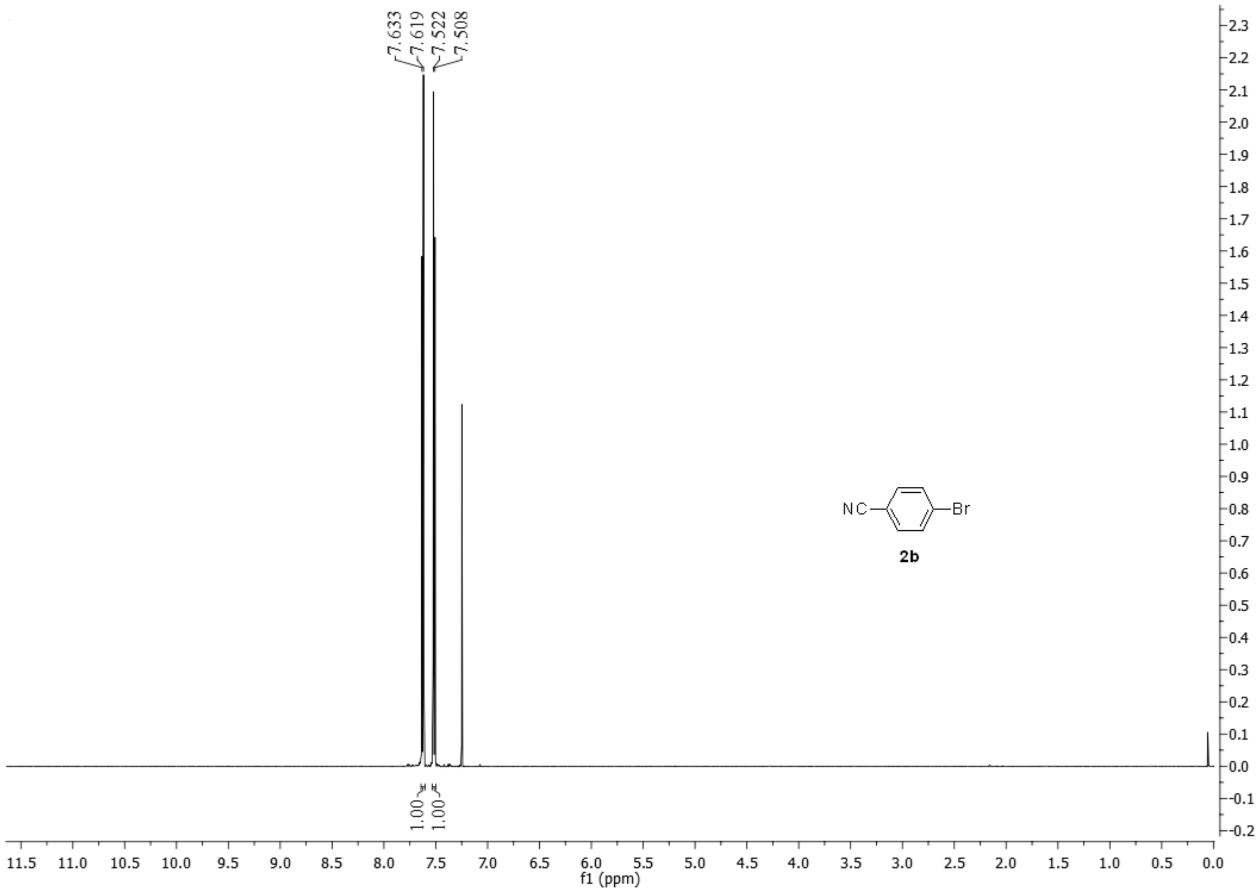
7.633
7.619
7.522
7.508
1.00
1.00
NC
Br
2b
f1 (ppm)

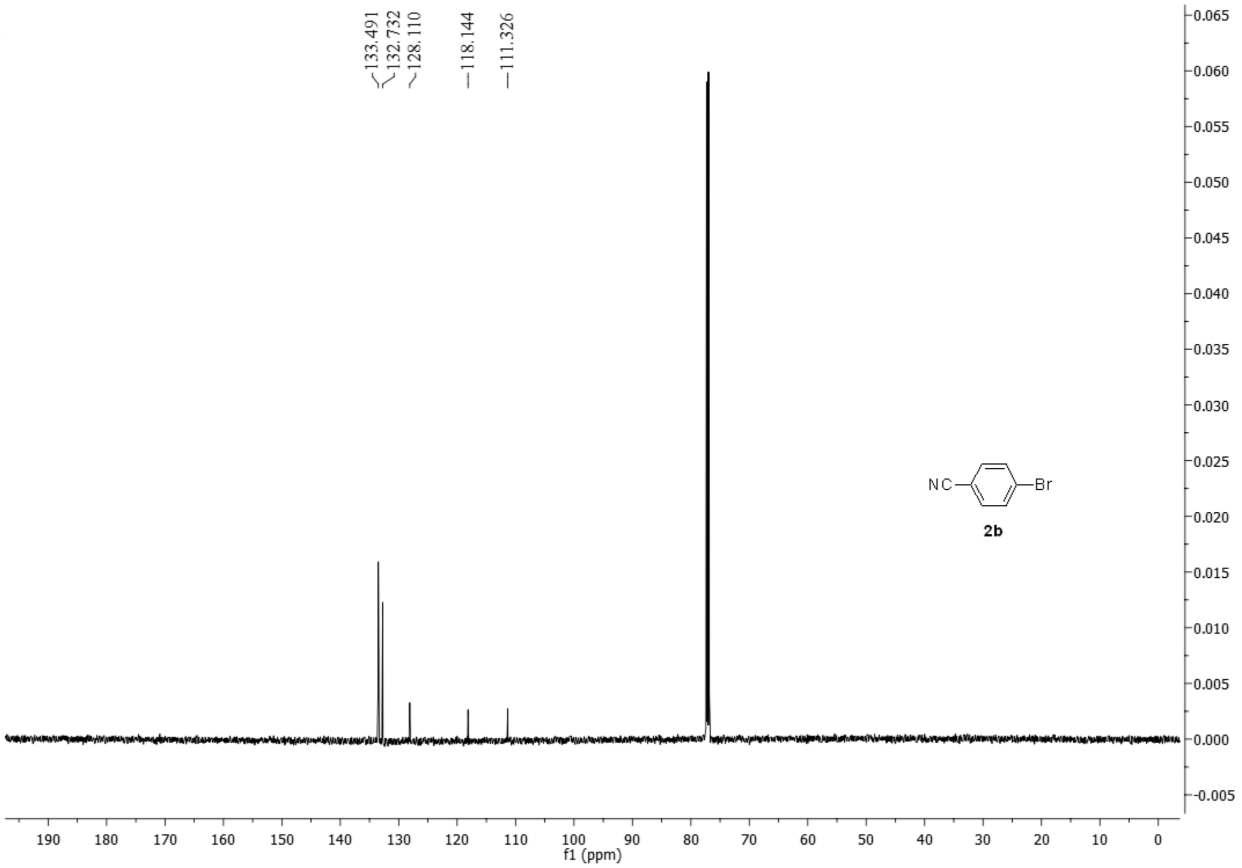
133.491
132.732
128.110
118.144
111.326
NC
Br
2b
f1 (ppm)

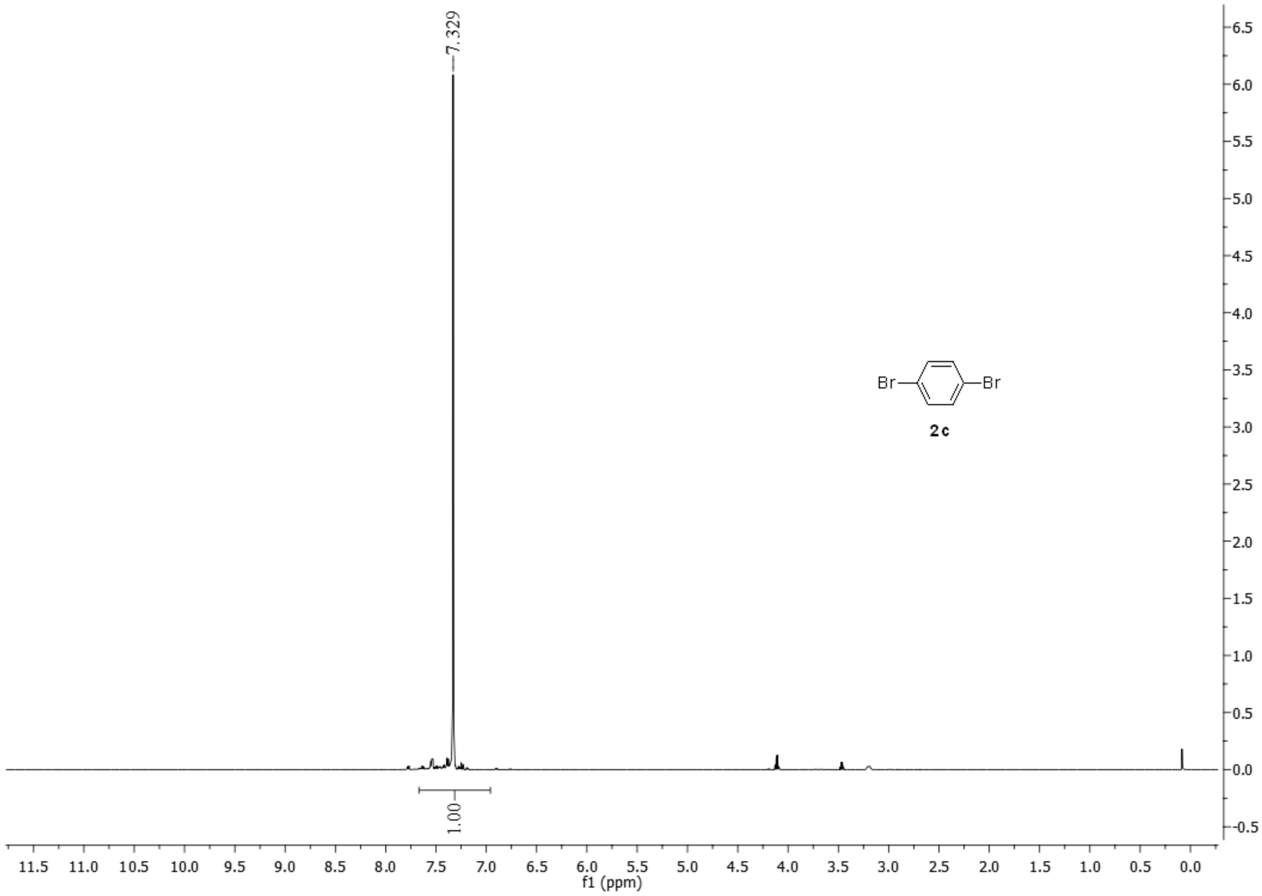
7.329
1.00
Br
Br
2c
f1 (ppm)

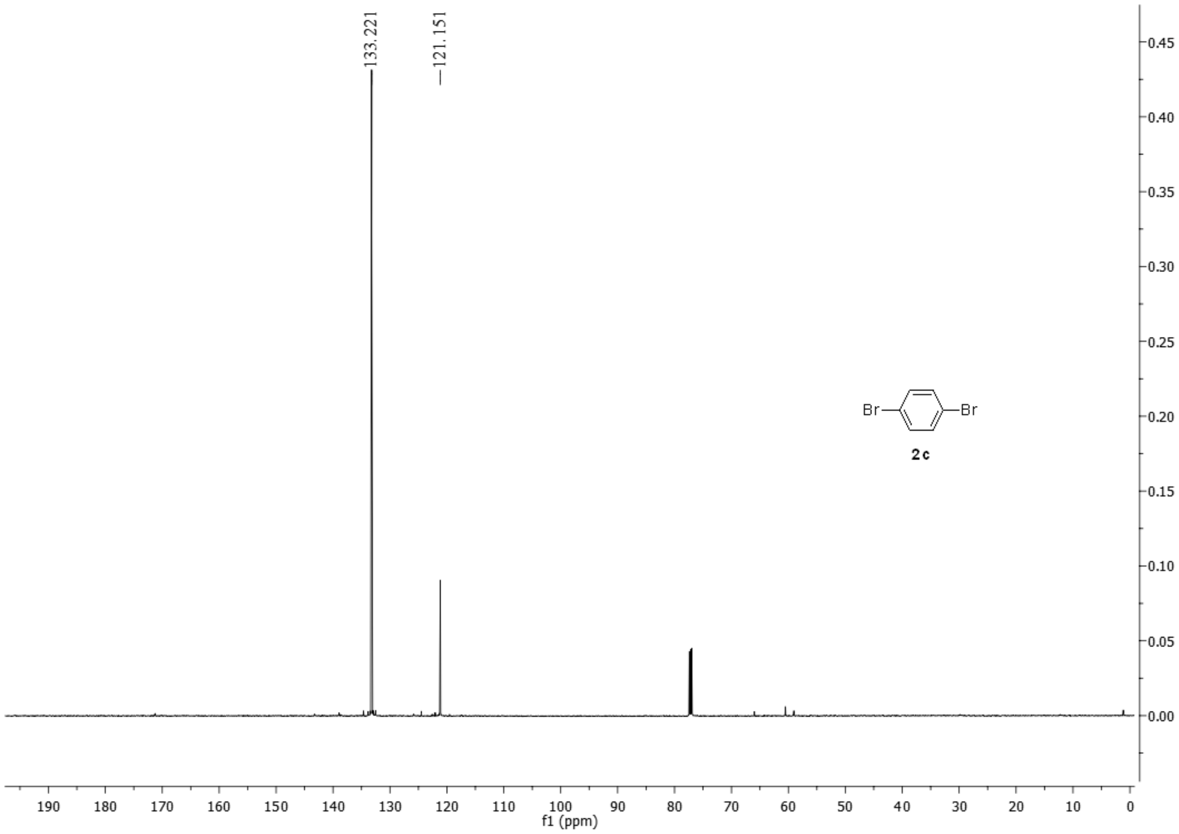
133.221
121.151
Br
Br
2c
f1 (ppm)

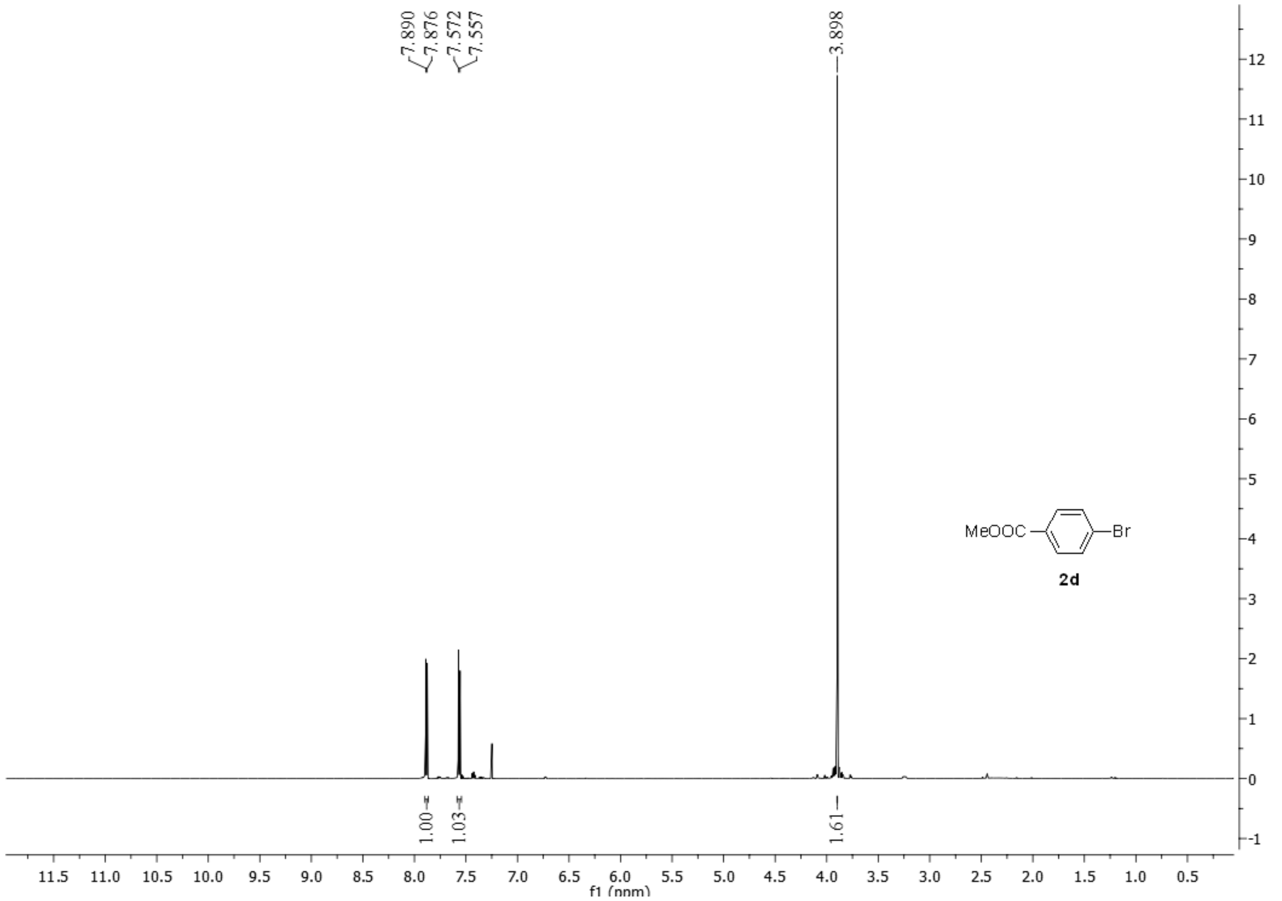
7.890
7.876
7.572
7.557
3.898
MeOOC
Br
2d
1.00
1.03
1.61
f1 (ppm)

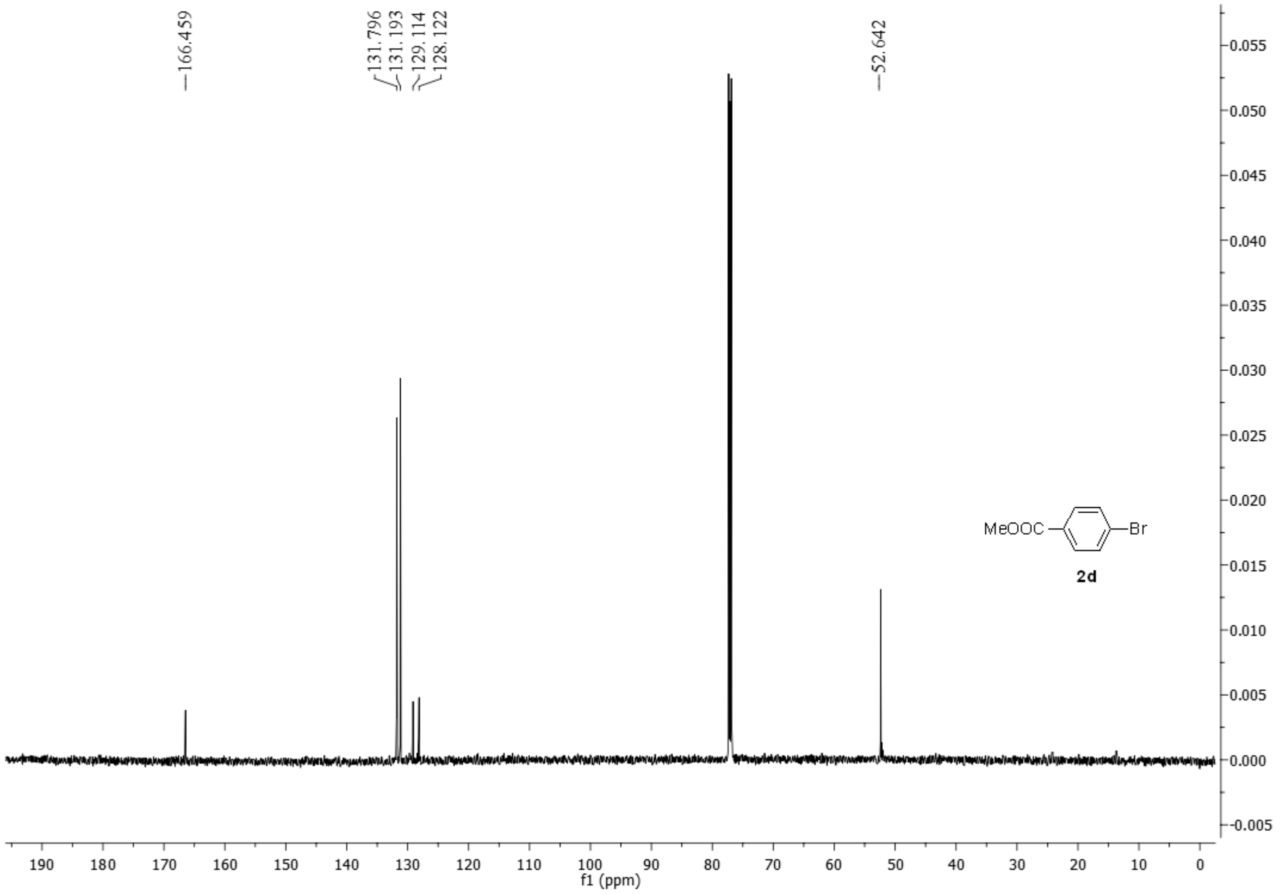
166.459
131.796
131.193
129.114
128.122
52.642
MeOOC
Br
2d
f1 (ppm)

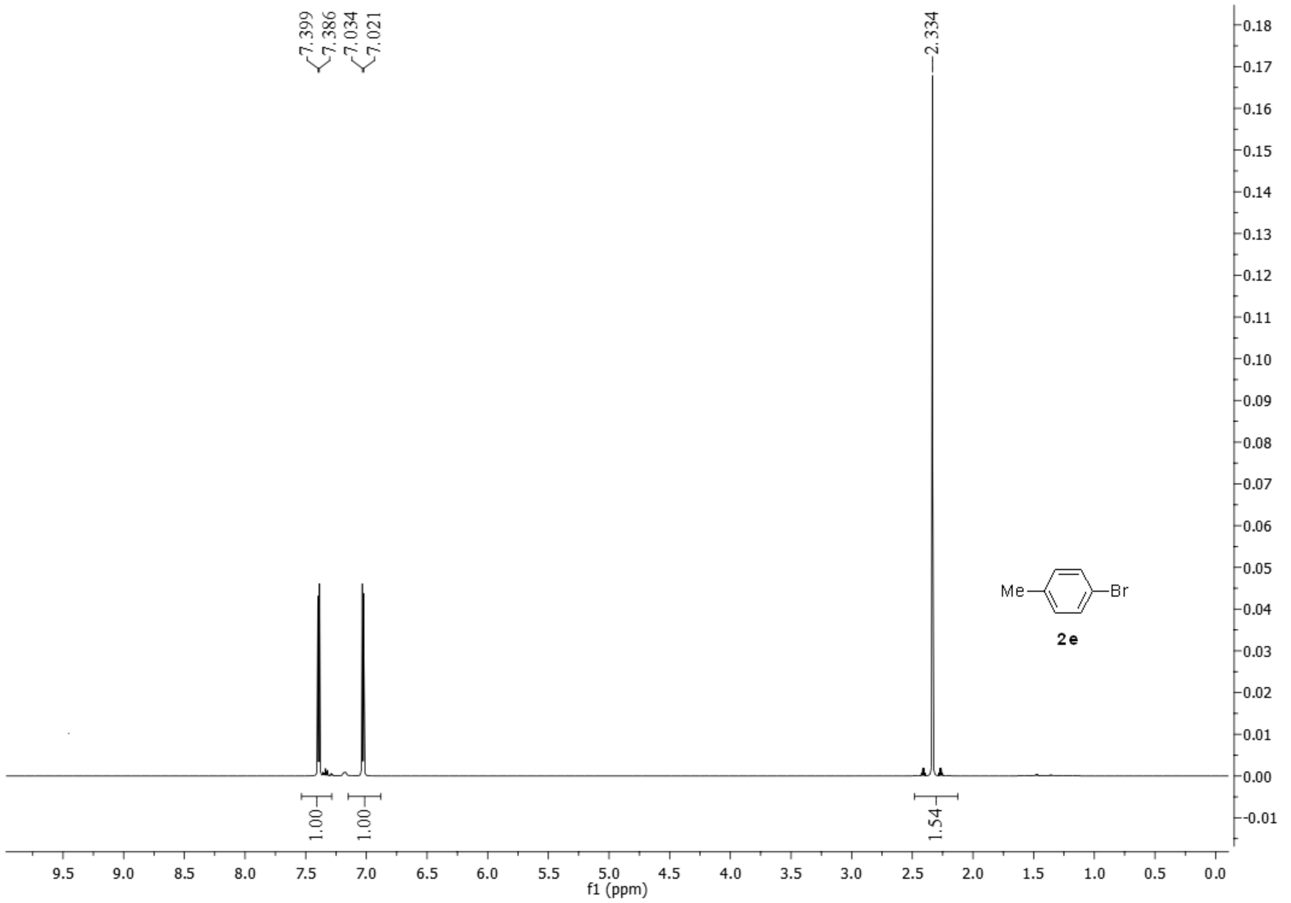
7.399
7.386
7.034
7.021
2.334
1.00
1.00
1.54
Me
Br
2e
9.5 9.0 8.5 8.0 7.5 7.0 6.5 6.0 5.5 5.0 4.5 4.0 3.5 3.0 2.5 2.0 1.5 1.0 0.5 0.0
f1 (ppm)
0.18 0.17 0.16 0.15 0.14 0.13 0.12 0.11 0.10 0.09 0.08 0.07 0.06 0.05 0.04 0.03 0.02 0.01 0.00 -0.01

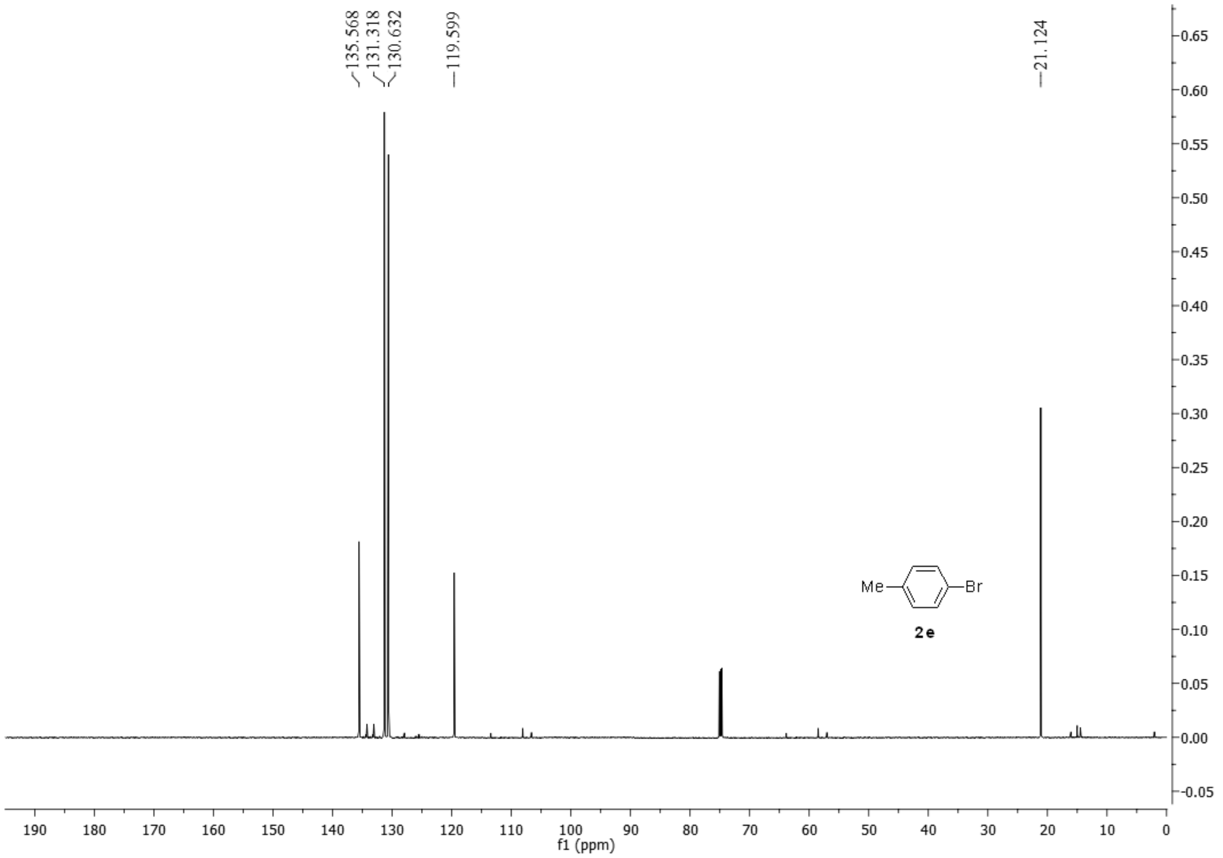
135.568
131.318
130.632
119.599
21.124
Me
Br
2e
190 180 170 160 150 140 130 120 110 100 90 80 70 60 50 40 30 20 10 0
f1 (ppm)
0.65 0.60 0.55 0.50 0.45 0.40 0.35 0.30 0.25 0.20 0.15 0.10 0.05 0.00 -0.05

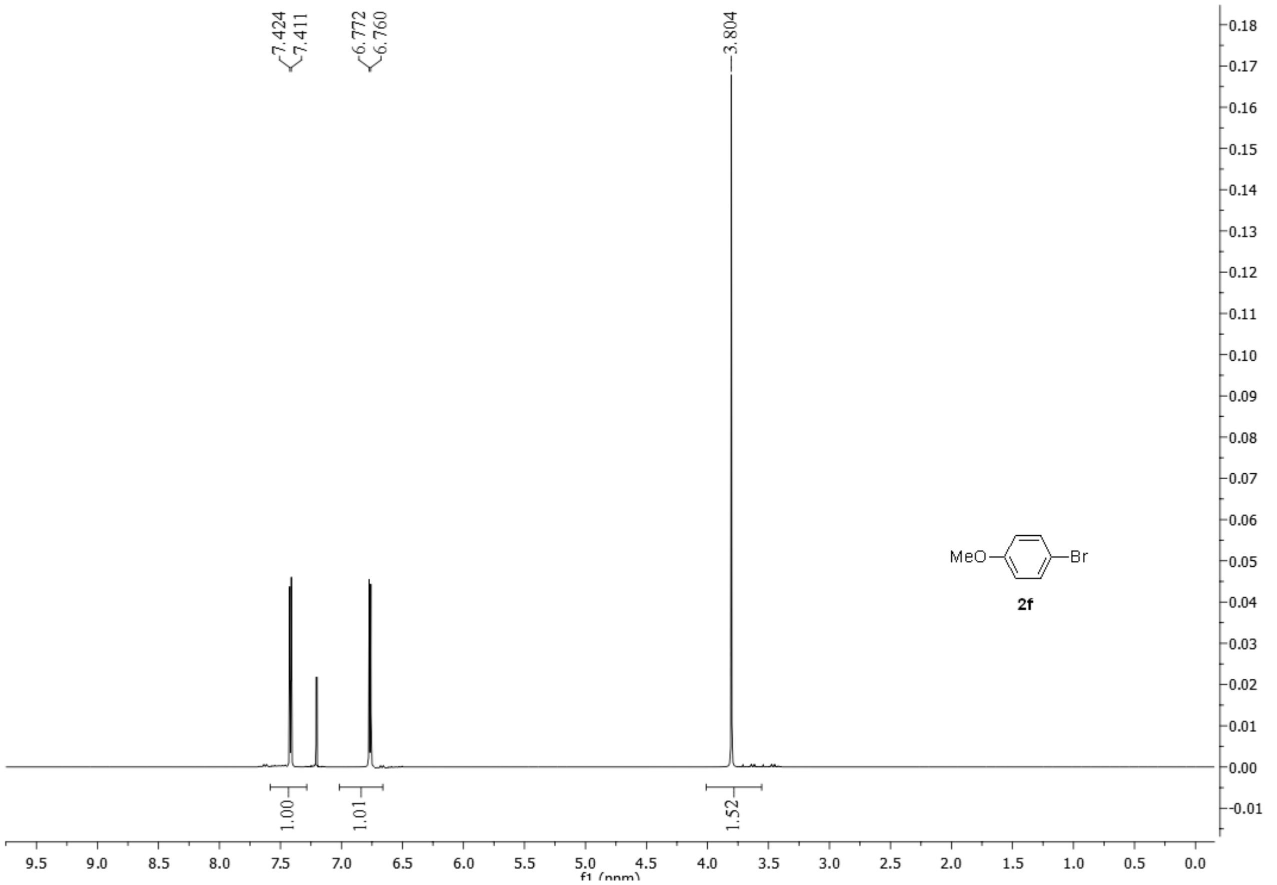

7.424
7.411
6.772
6.760
3.804
1.00
1.01
1.52
MeO
Br
2f
f1 (ppm)

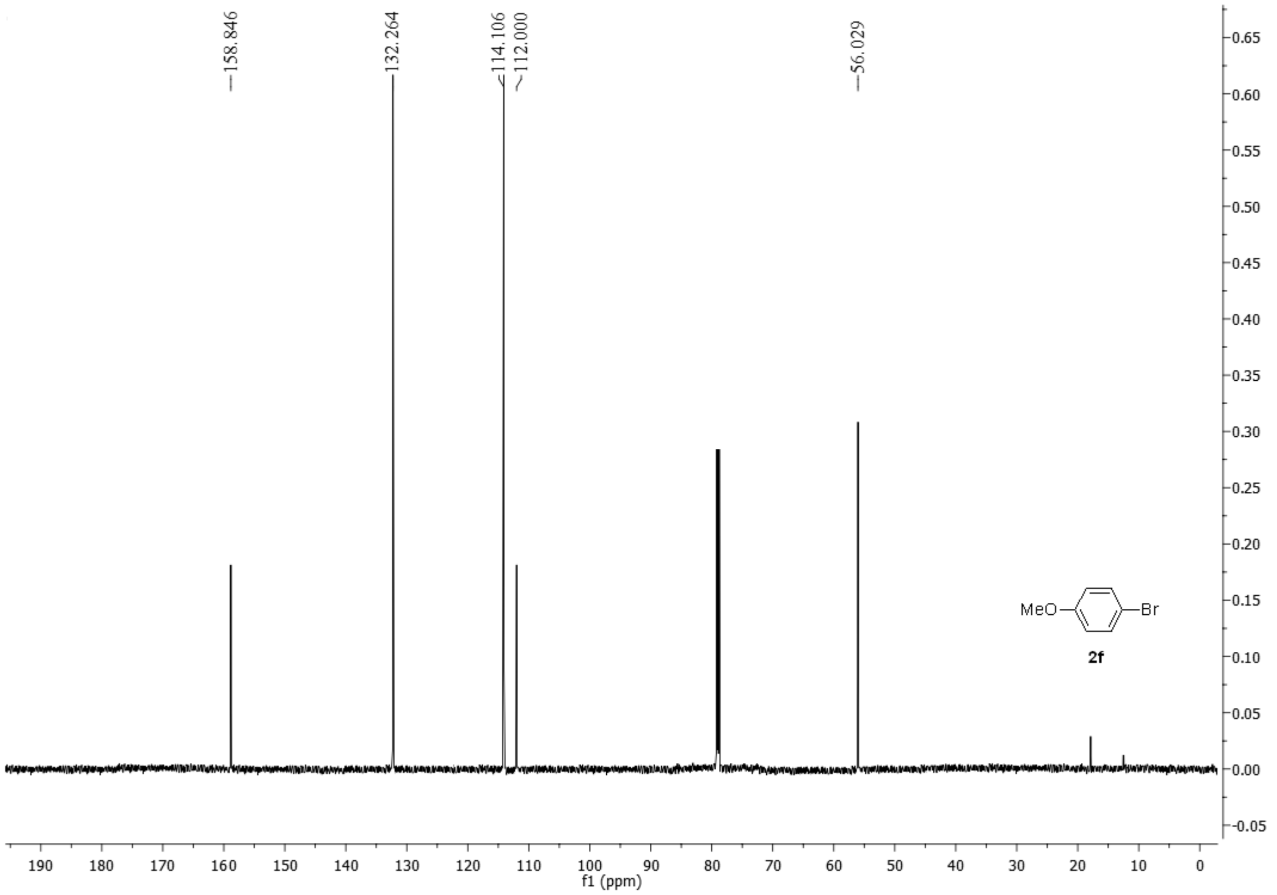

158.846
132.264
114.106
112.000
56.029
MeO
Br
2f
f1 (ppm)

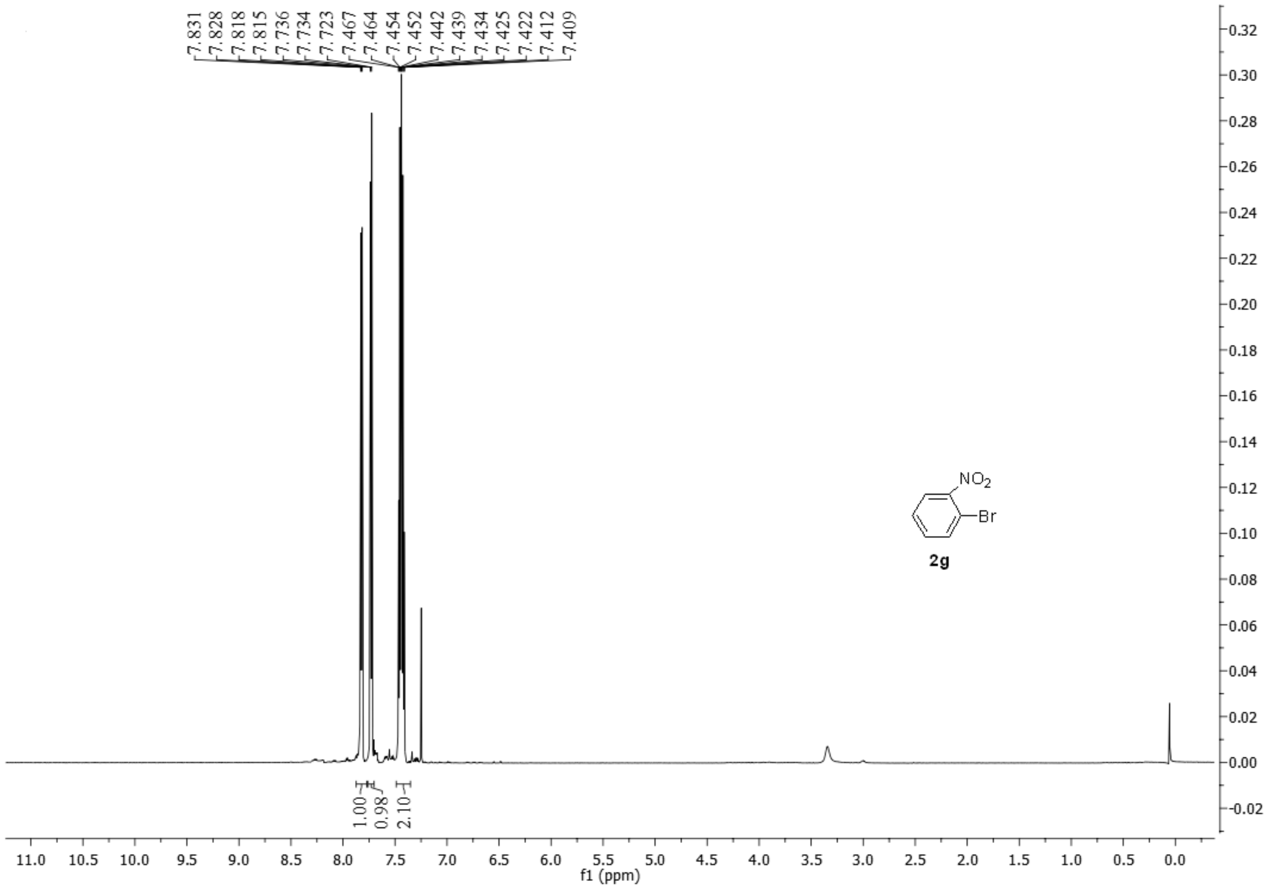
7.831
7.828
7.818
7.815
7.736
7.734
7.723
7.467
7.464
7.454
7.452
7.442
7.439
7.434
7.425
7.422
7.412
7.409
1.00
0.98
2.10
NO2
Br
2g
f1 (ppm)

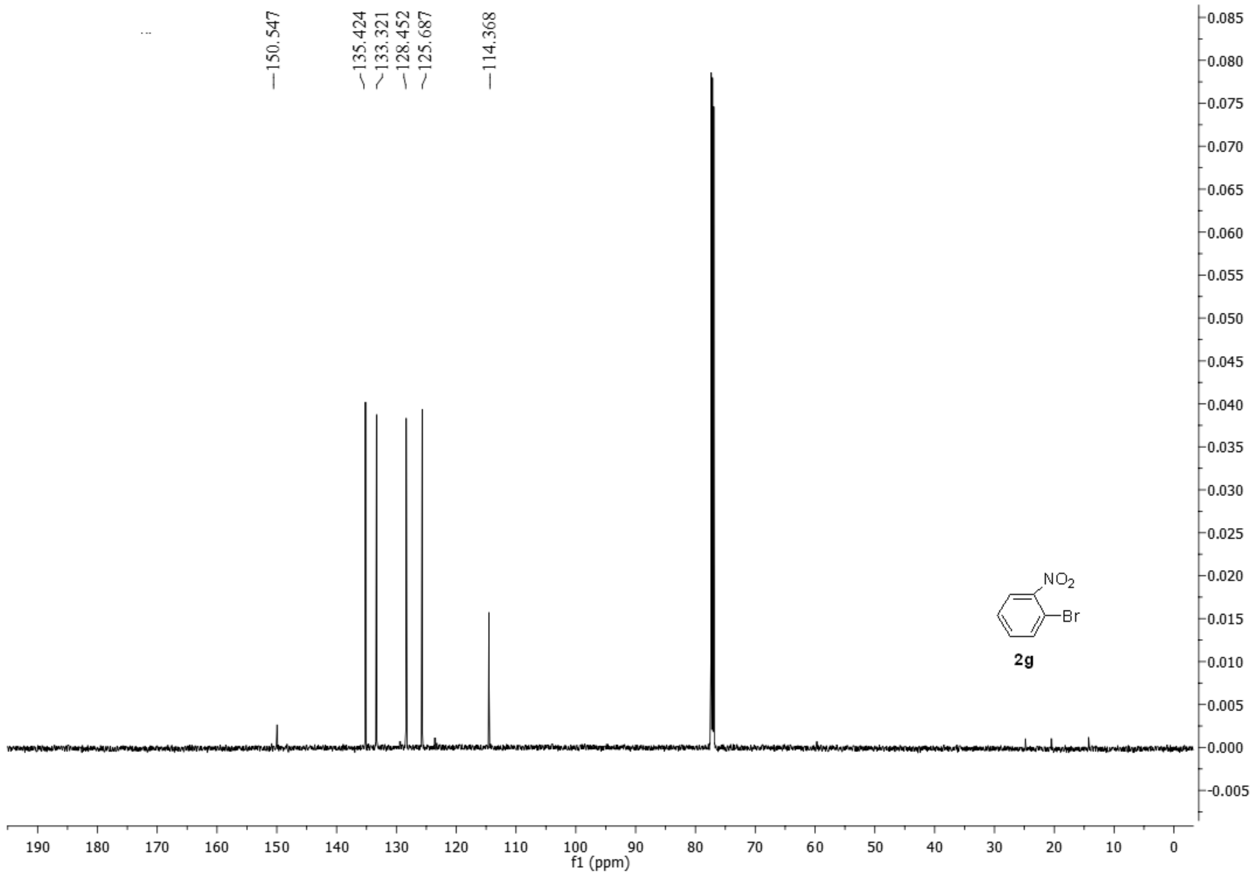
150.547
135.424
133.321
128.452
125.687
114.368
NO2
Br
2g
f1 (ppm)

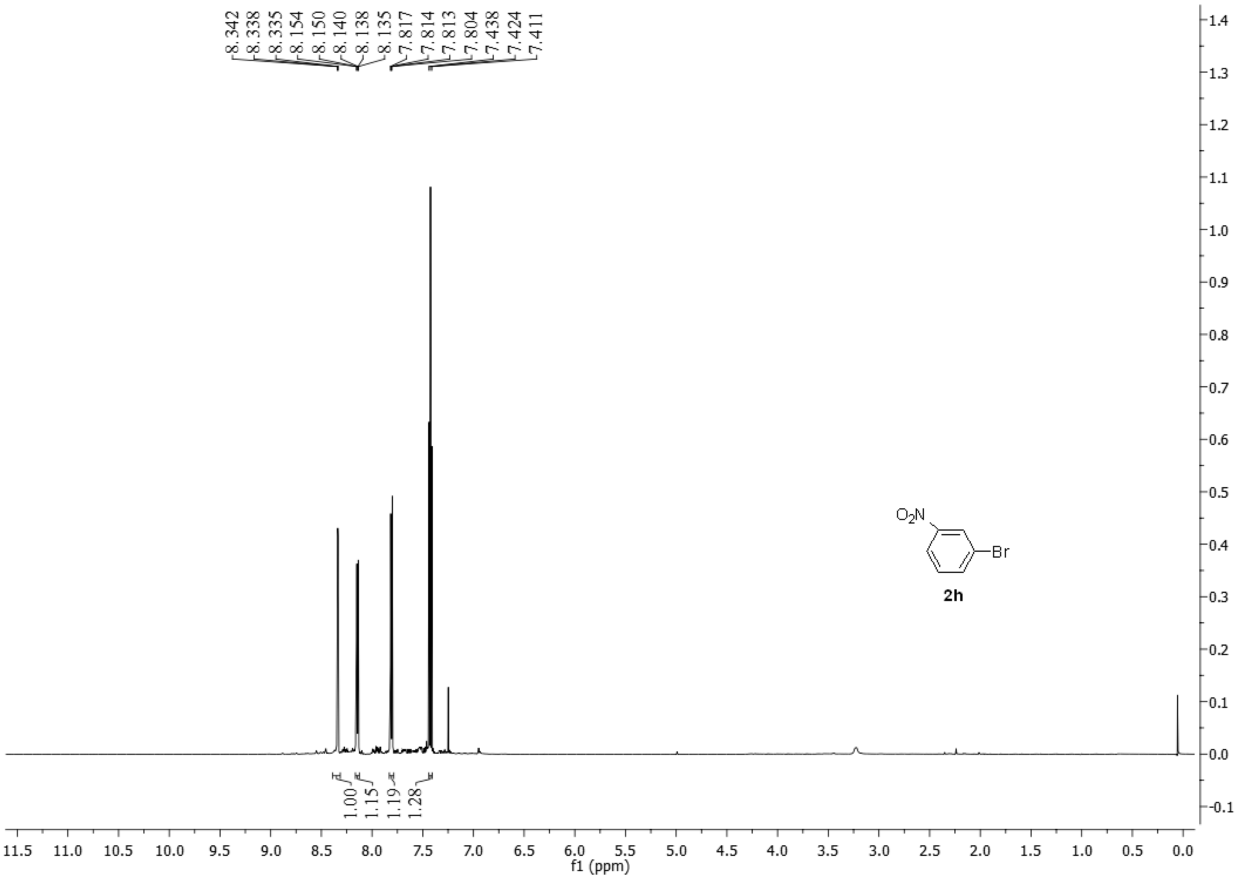

8.342
8.338
8.335
8.154
8.150
8.140
8.138
8.135
7.817
7.814
7.813
7.804
7.438
7.424
7.411
1.00
1.15
1.19
1.28
f1 (ppm)
O_2N
Br
2h

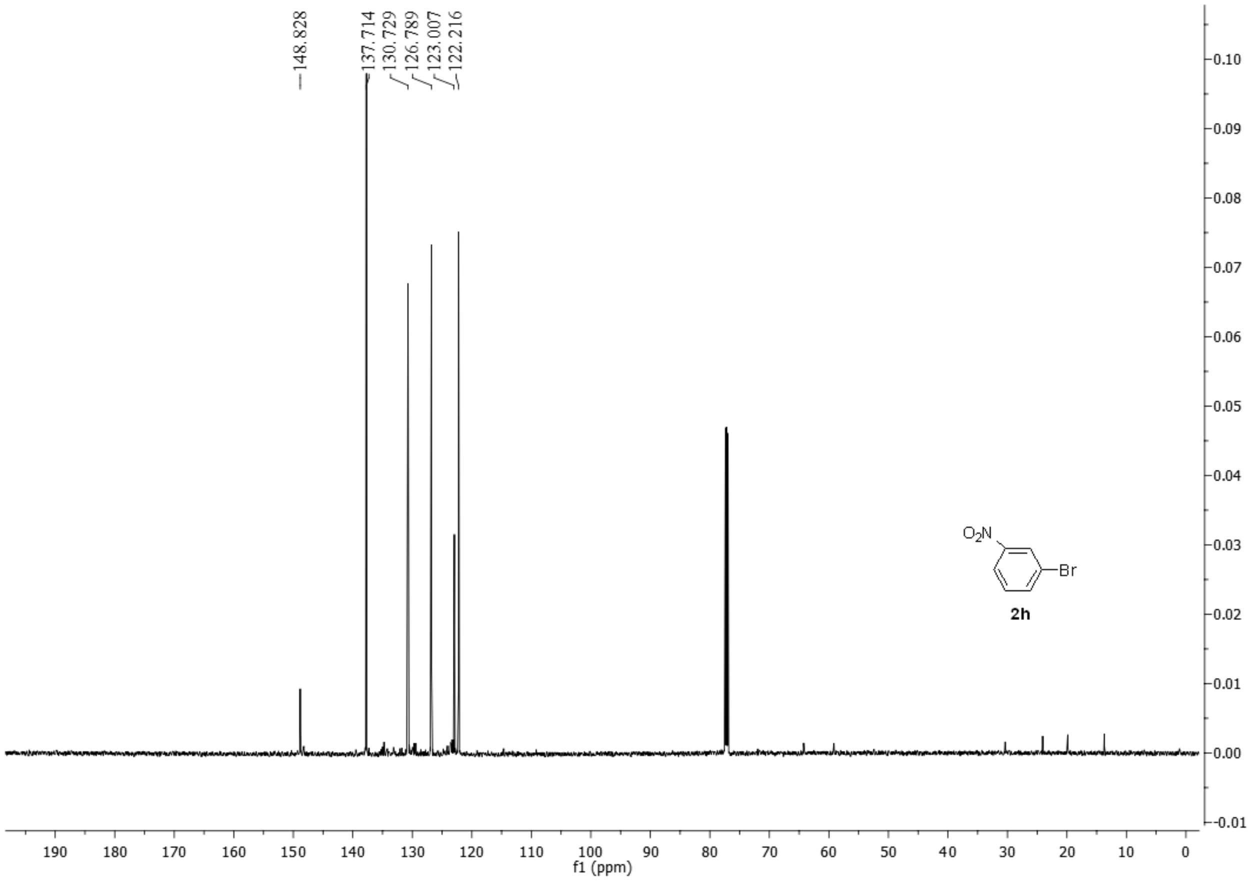

148.828
137.714
130.729
126.789
123.007
122.216
f1 (ppm)
O_2N
Br
2h

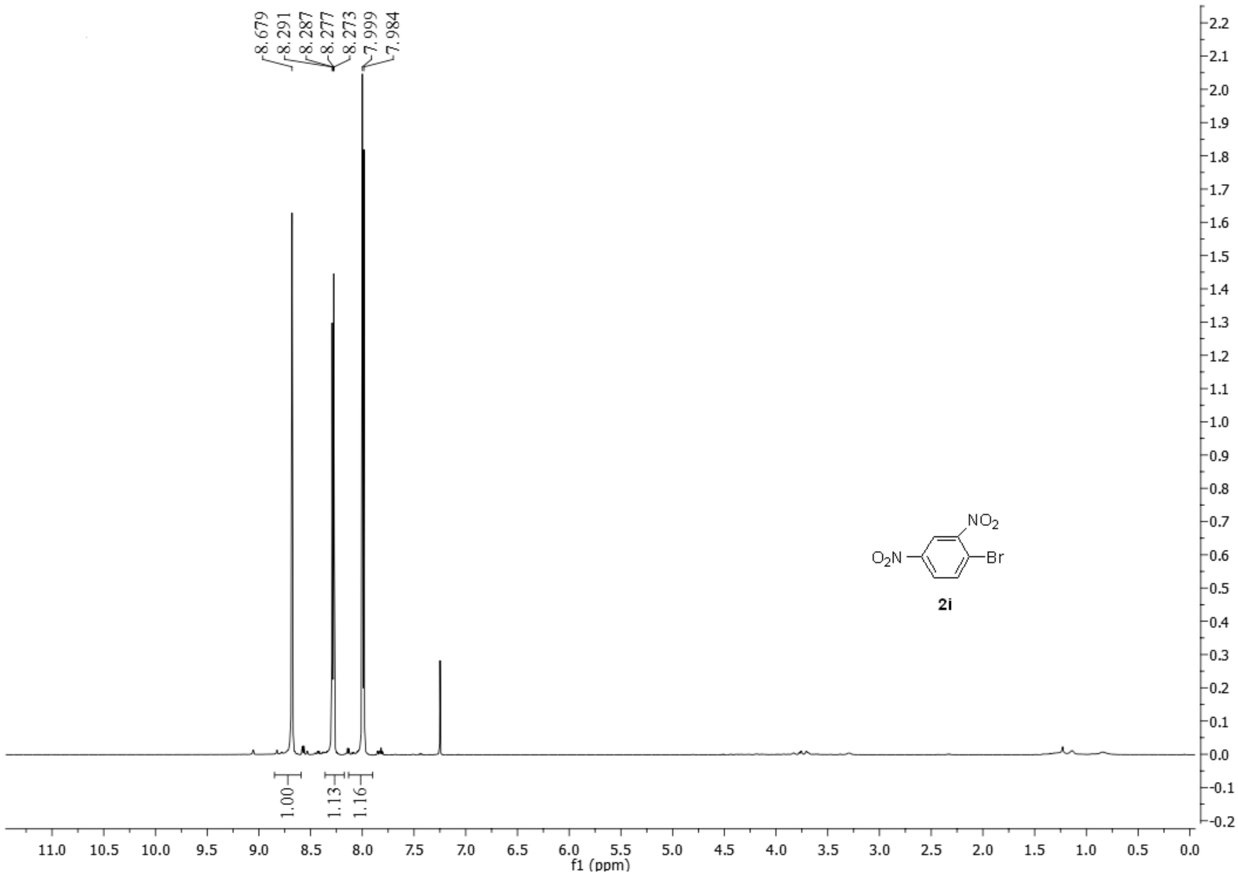

8.679
8.291
8.287
8.277
8.273
7.999
7.984
1.00
1.13
1.16
f1 (ppm)
NO2
O2N
Br
2i

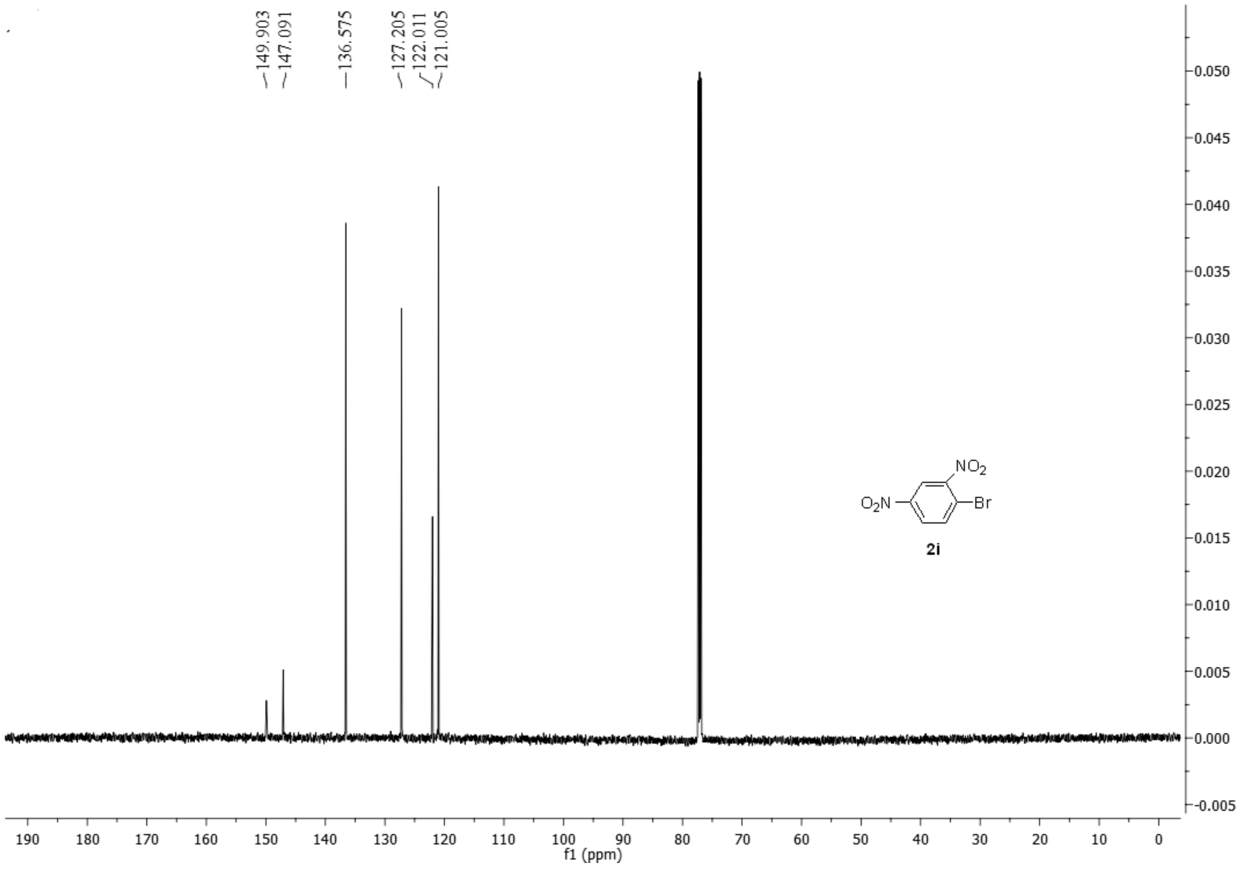

149.903
147.091
136.575
127.205
122.011
121.005
f1 (ppm)
NO2
O2N
Br
2i

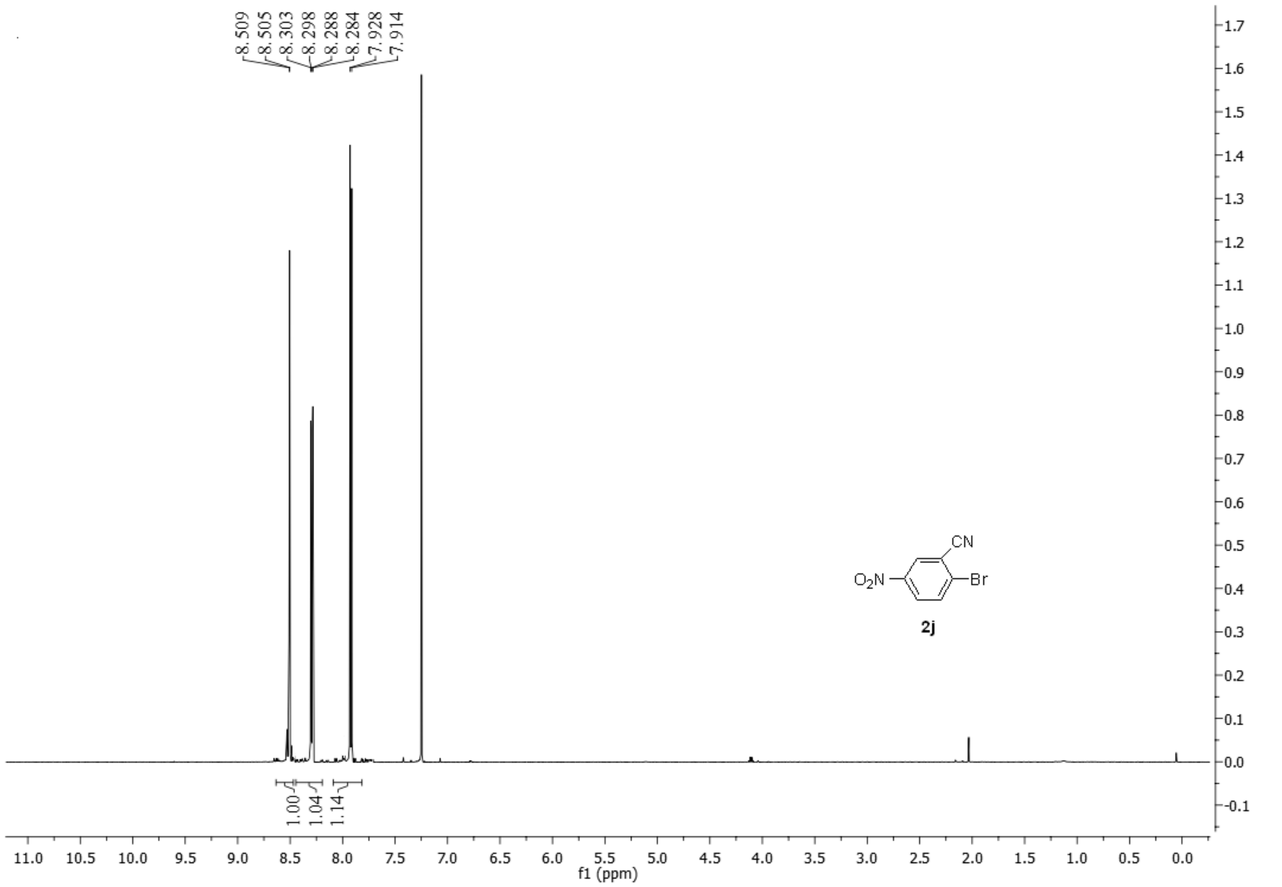
8.509
8.505
8.303
8.298
8.288
8.284
7.928
7.914
1.00
1.04
1.14
CN
O_2N
Br
2j
f1 (ppm)

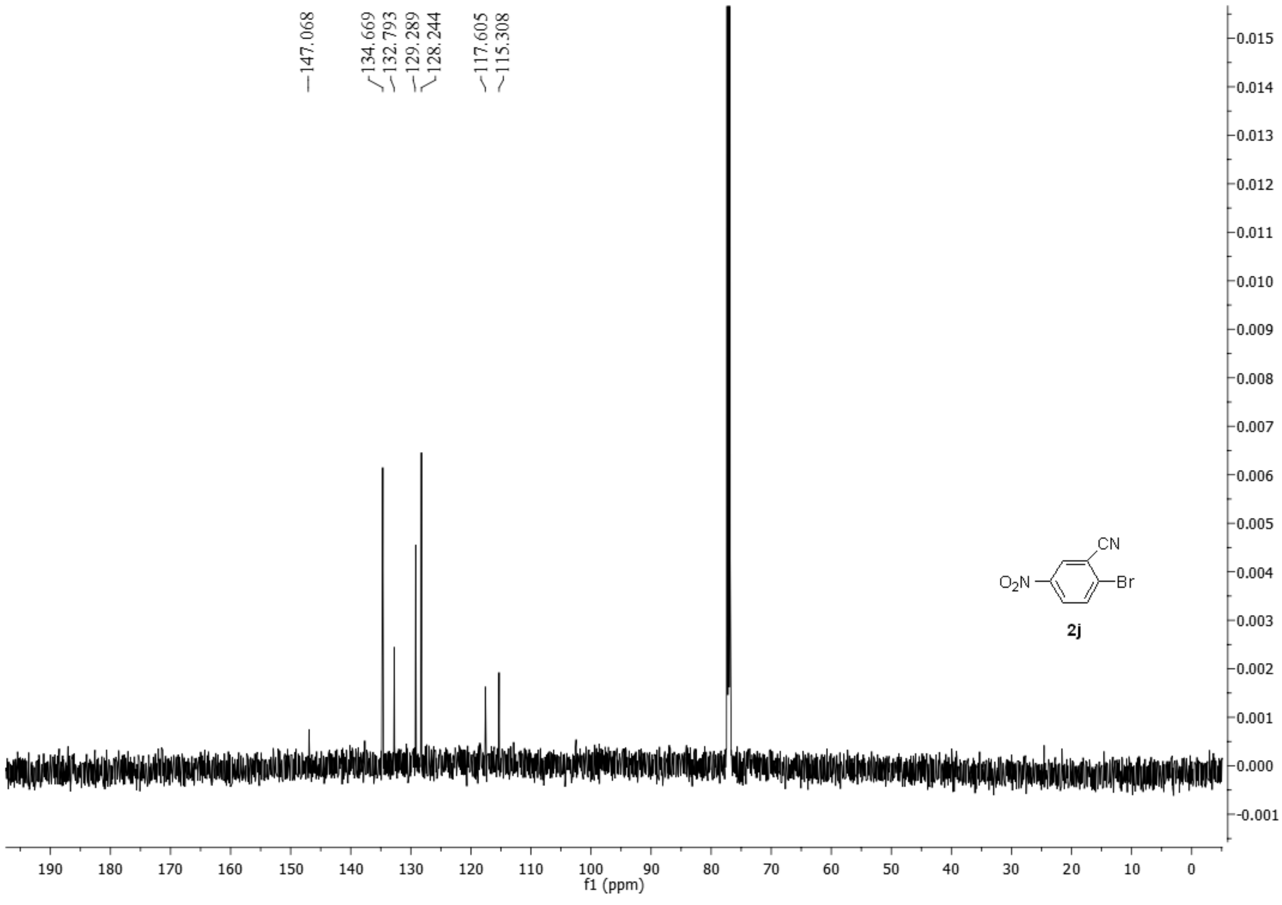
147.068
134.669
132.793
129.289
128.244
117.605
115.308
CN
O_2N
Br
2j
f1 (ppm)

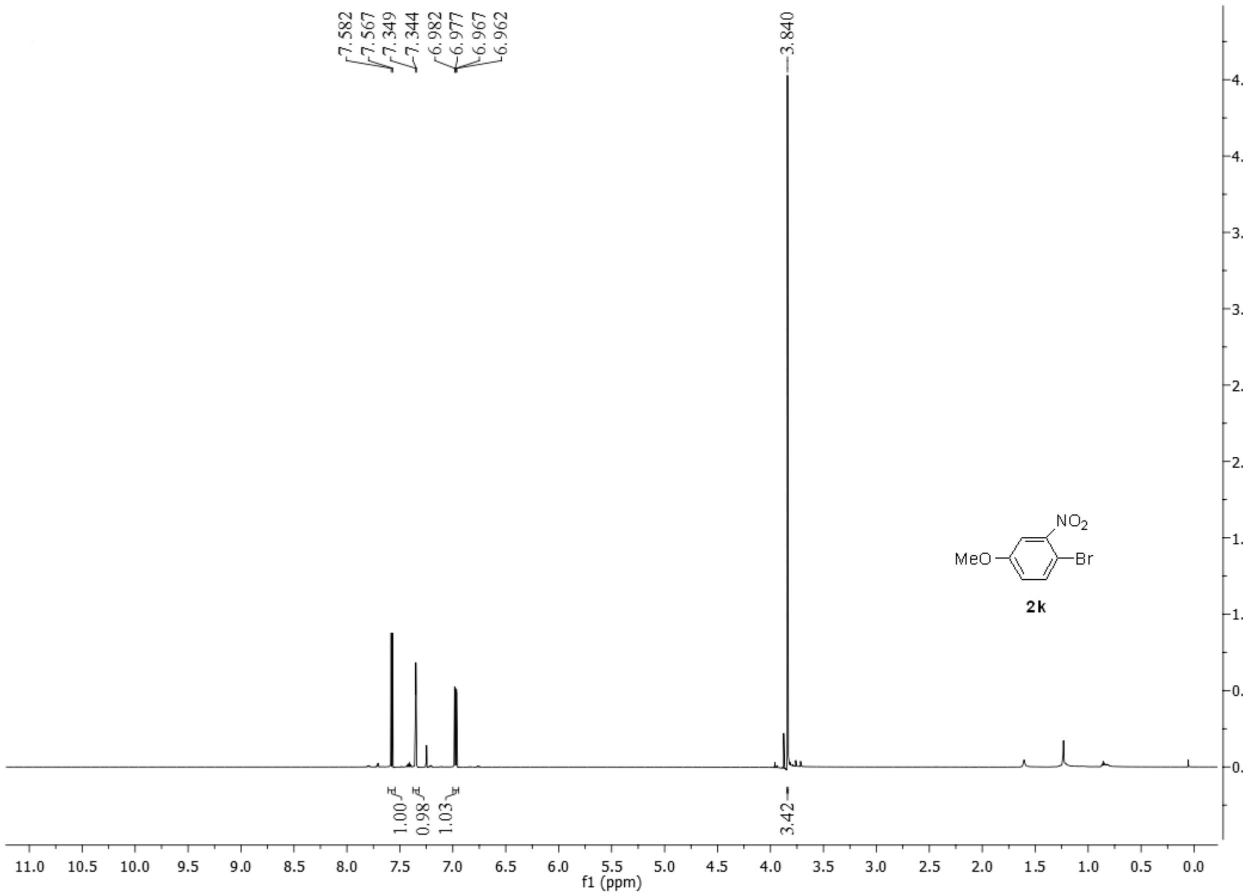
7.582
7.567
7.349
7.344
6.982
6.977
6.967
6.962
3.840
1.00
0.98
1.03
3.42
NO2
MeO
Br
2k
f1 (ppm)

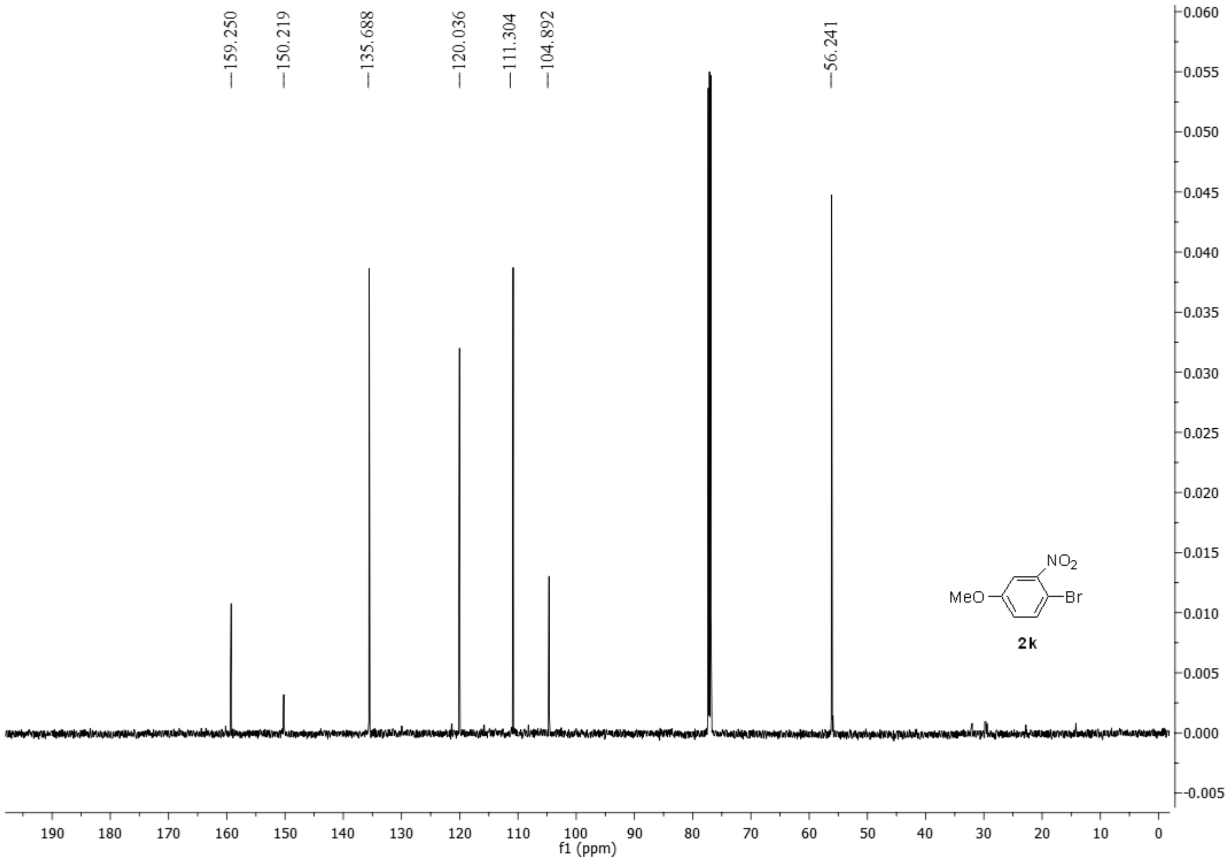
159.250
150.219
135.688
120.036
111.304
104.892
56.241
NO2
MeO
Br
2k
f1 (ppm)

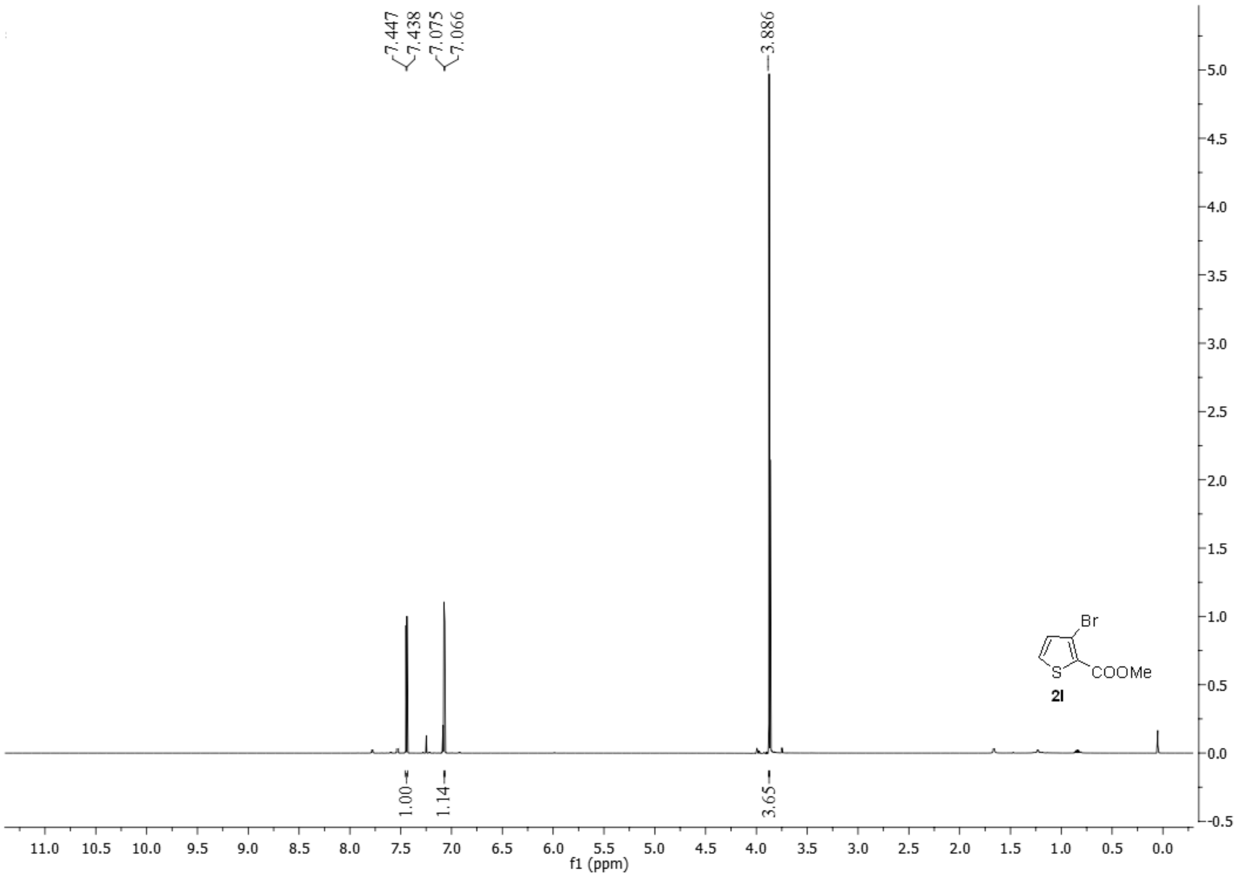
7.447
7.438
7.075
7.066
3.886
1.00
1.14
3.65
Br
S
COOMe
2l
f1 (ppm)

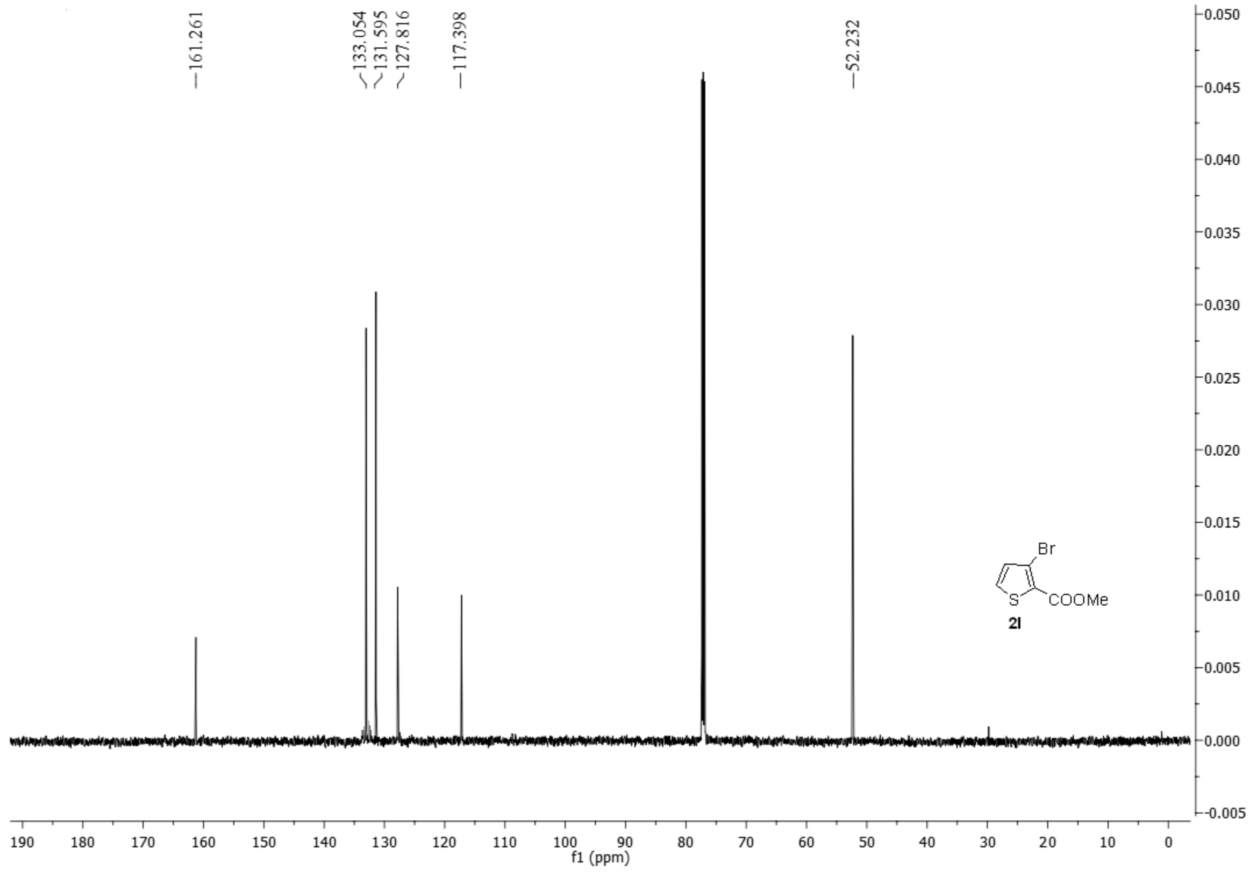
161.261
133.054
131.595
127.816
117.398
52.232
Br
S
COOMe
2l
f1 (ppm)

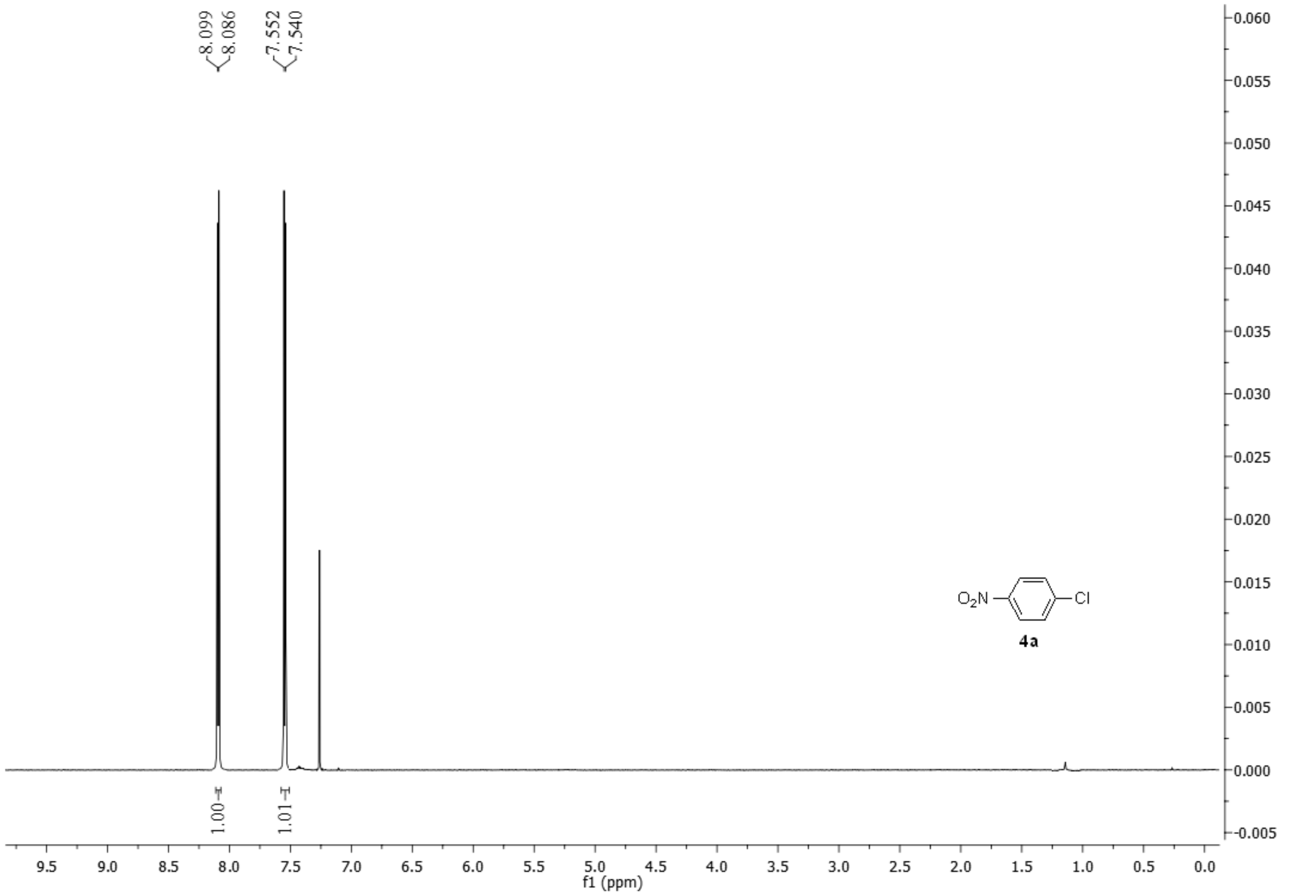
8.099
8.086
7.552
7.540
O2N Cl
4a
1.00
1.01
f1 (ppm)

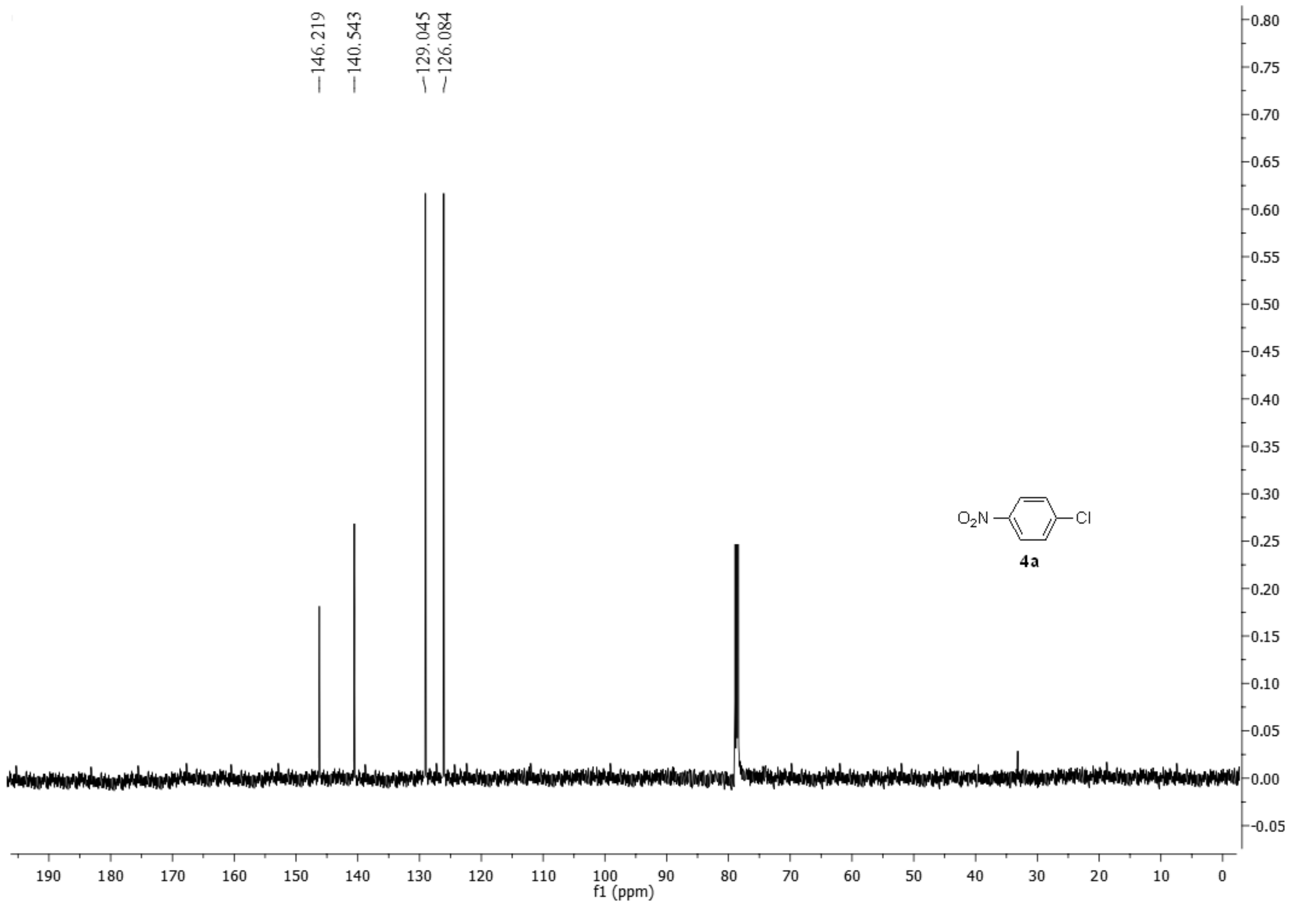
146.219
140.543
129.045
126.084
O2N Cl
4a
f1 (ppm)

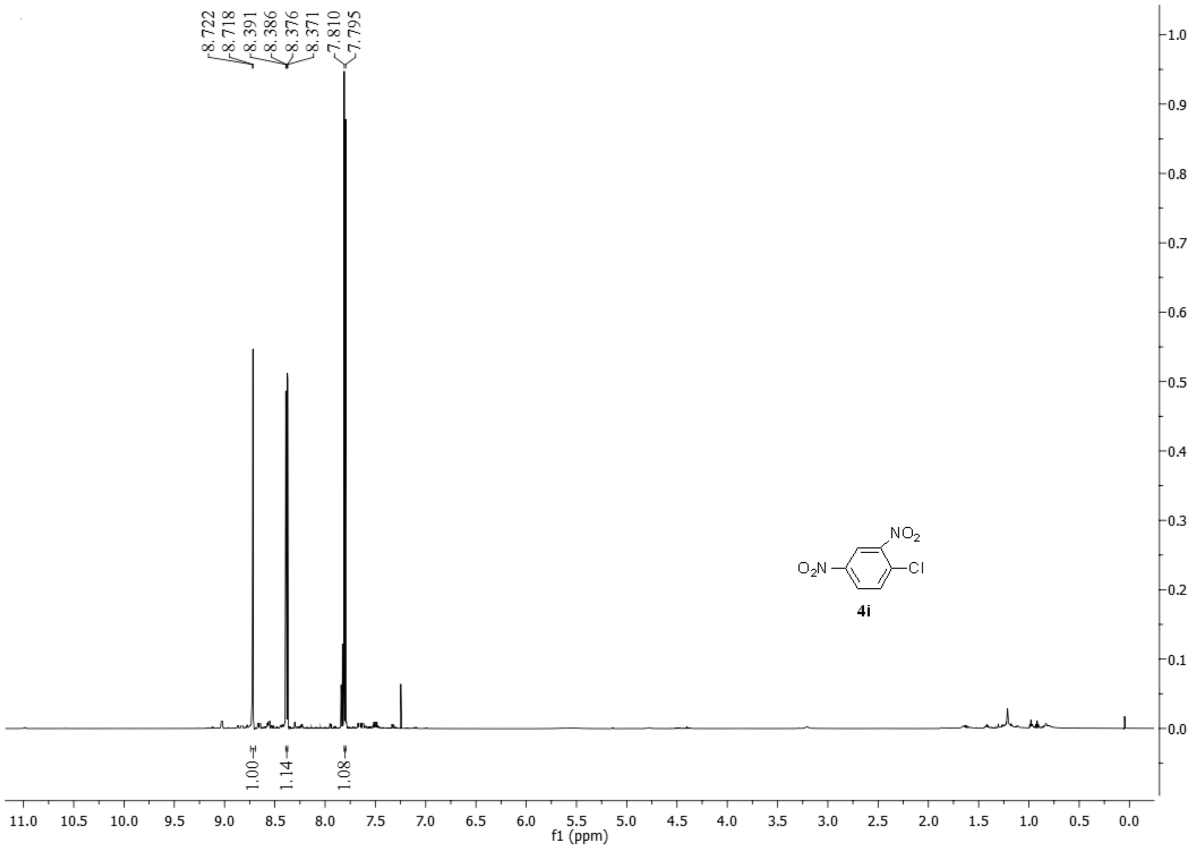

Highlight of the zone between 7.75 and 8.80 ppm

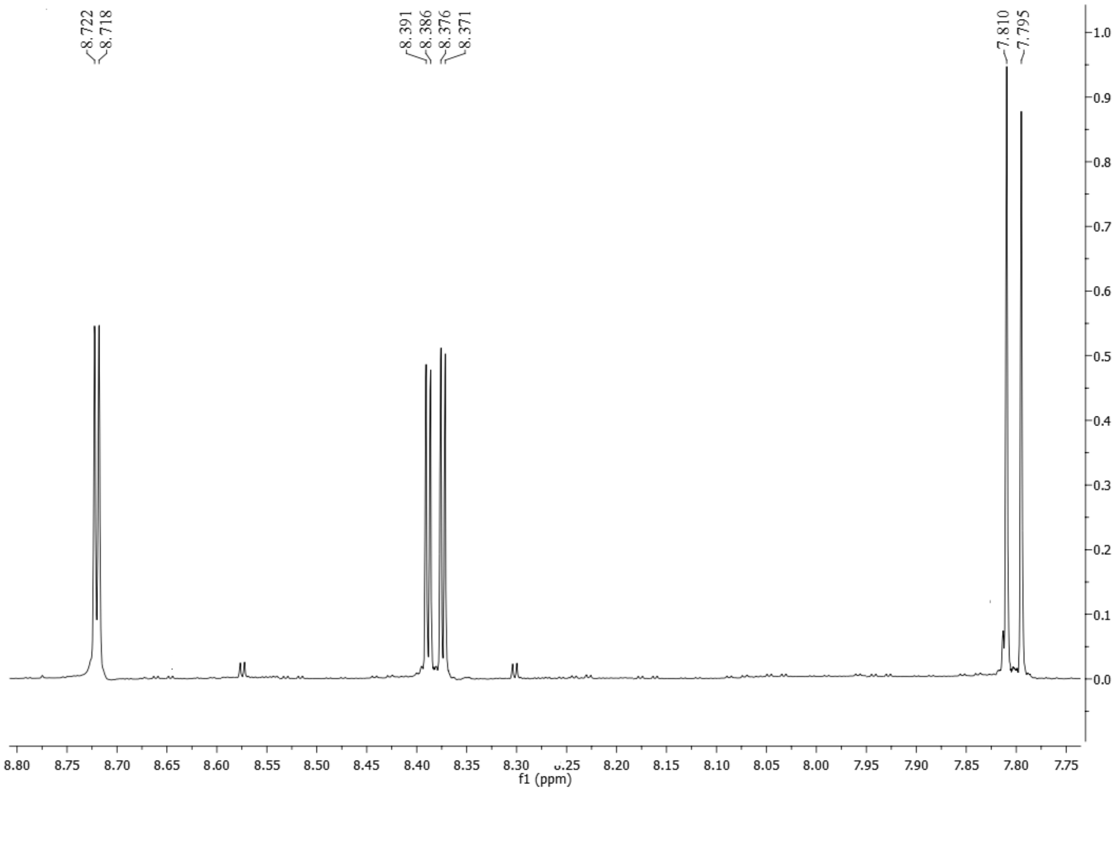

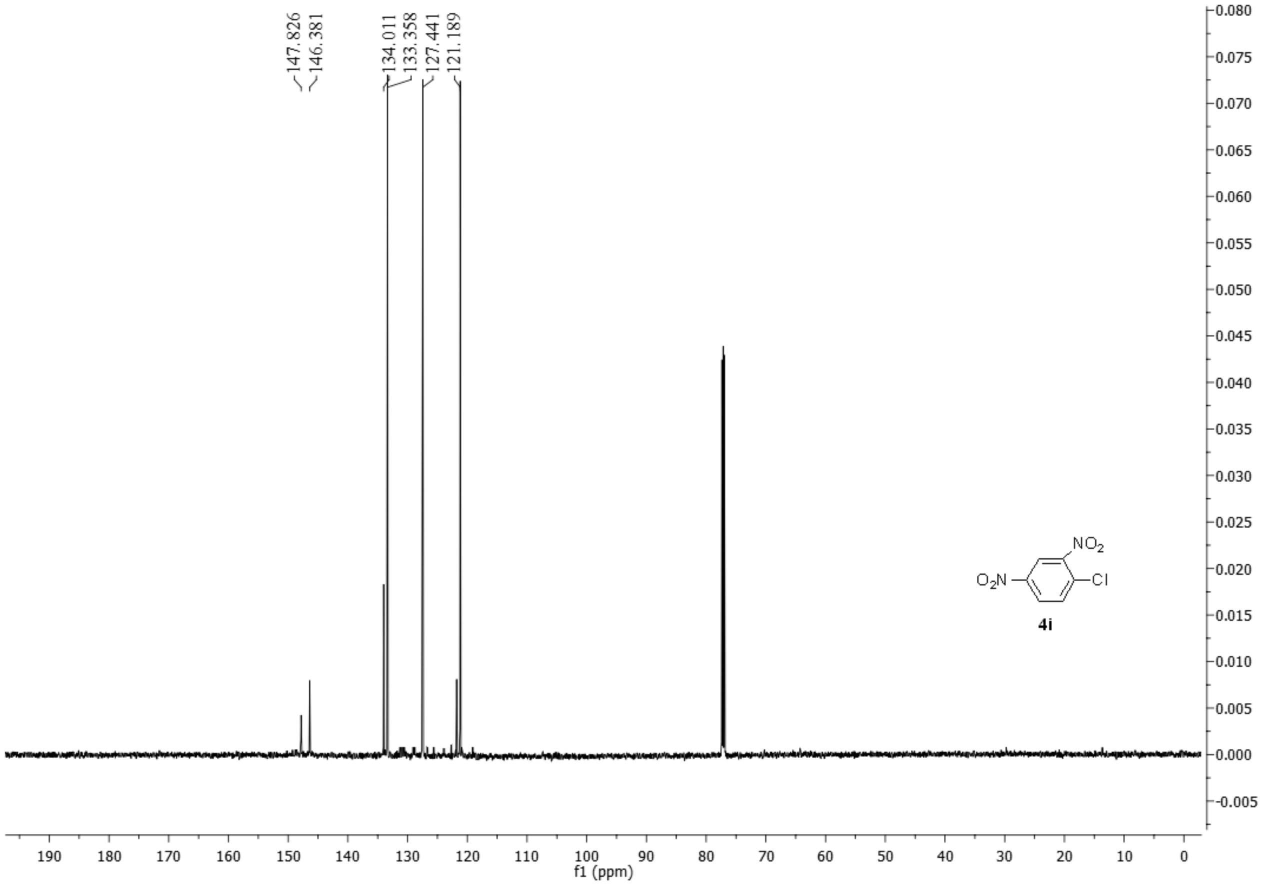

Highlight of the zone between 149 and 121 ppm

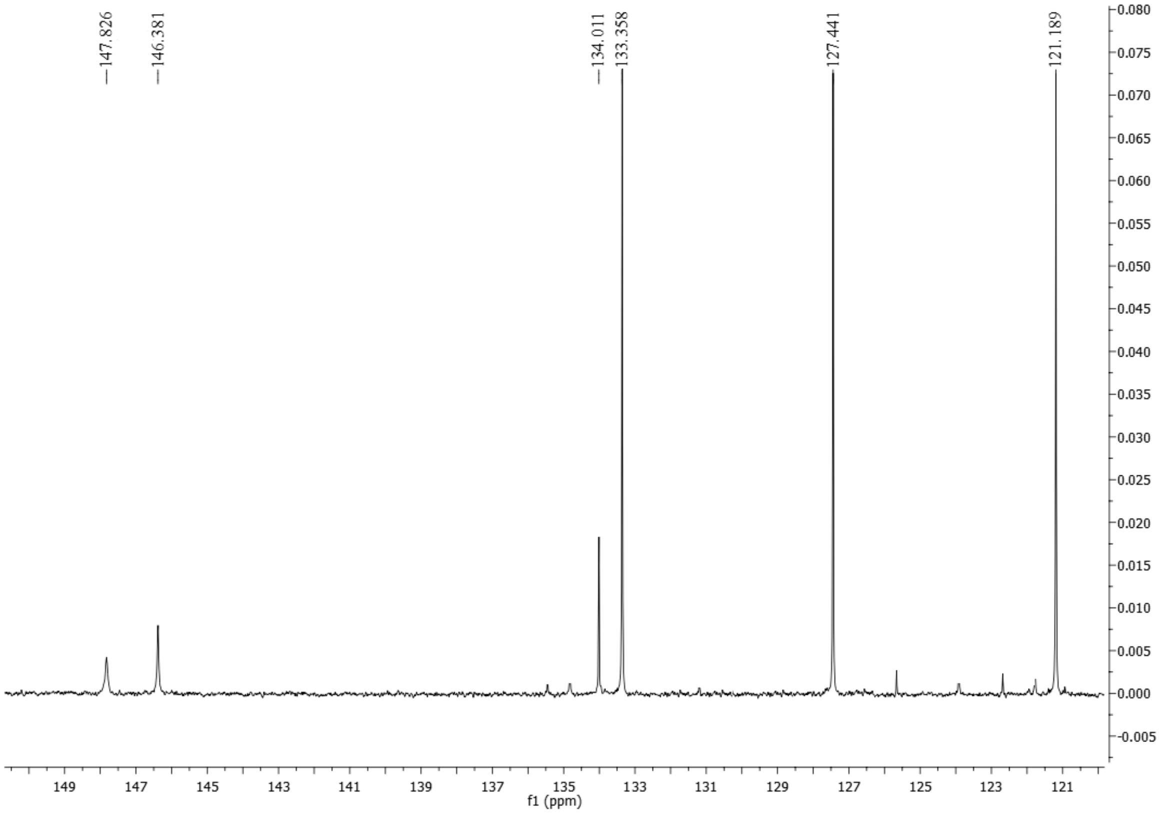

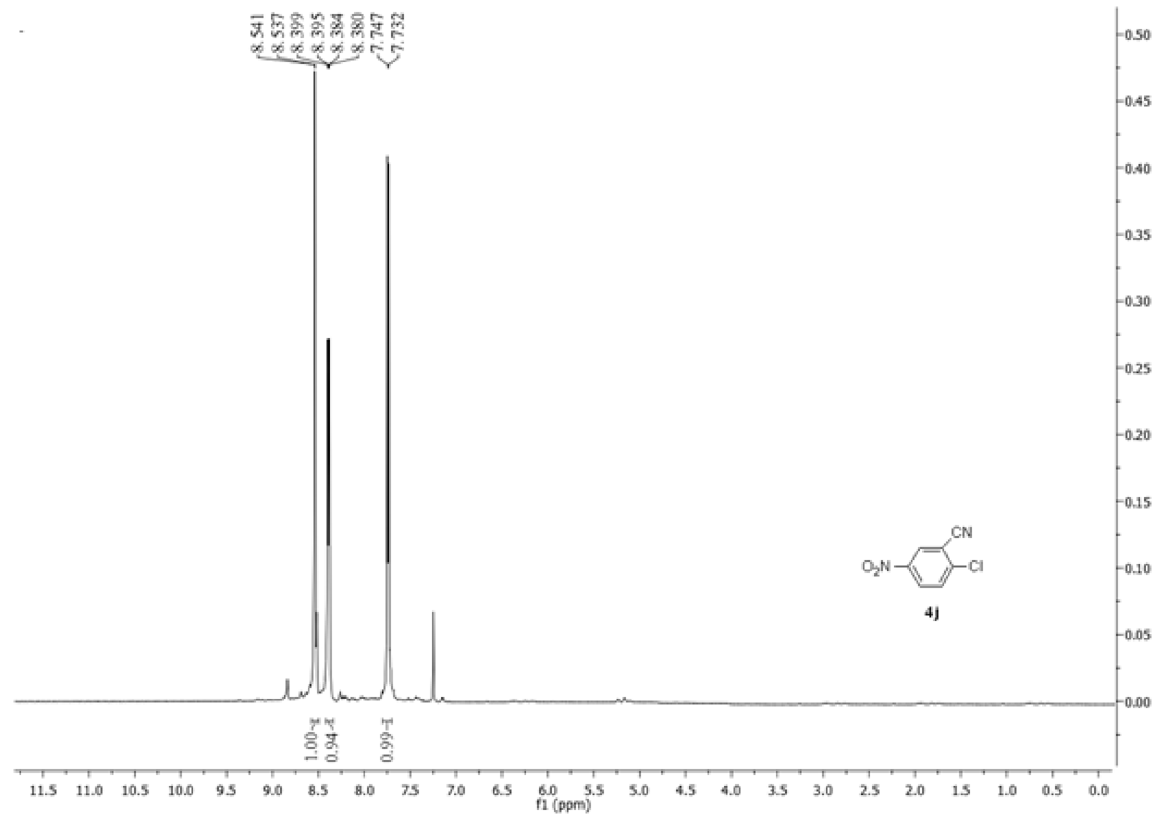
8.541
8.537
8.399
8.395
8.384
8.380
7.747
7.732
1.00
0.94
0.99
O2N
CN
Cl
4j
f1 (ppm)

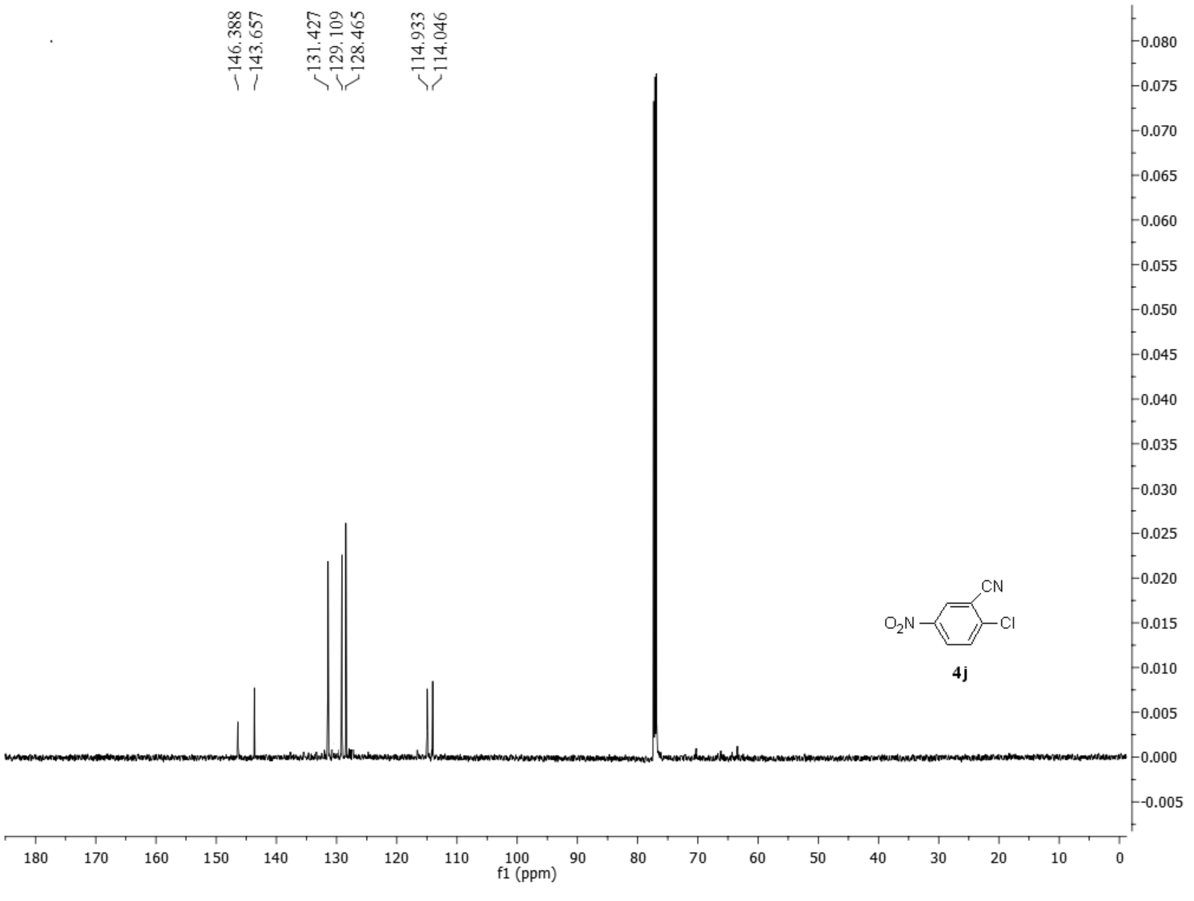
146.388
143.657
131.427
129.109
128.465
114.933
114.046
O2N
CN
Cl
4j
f1 (ppm)

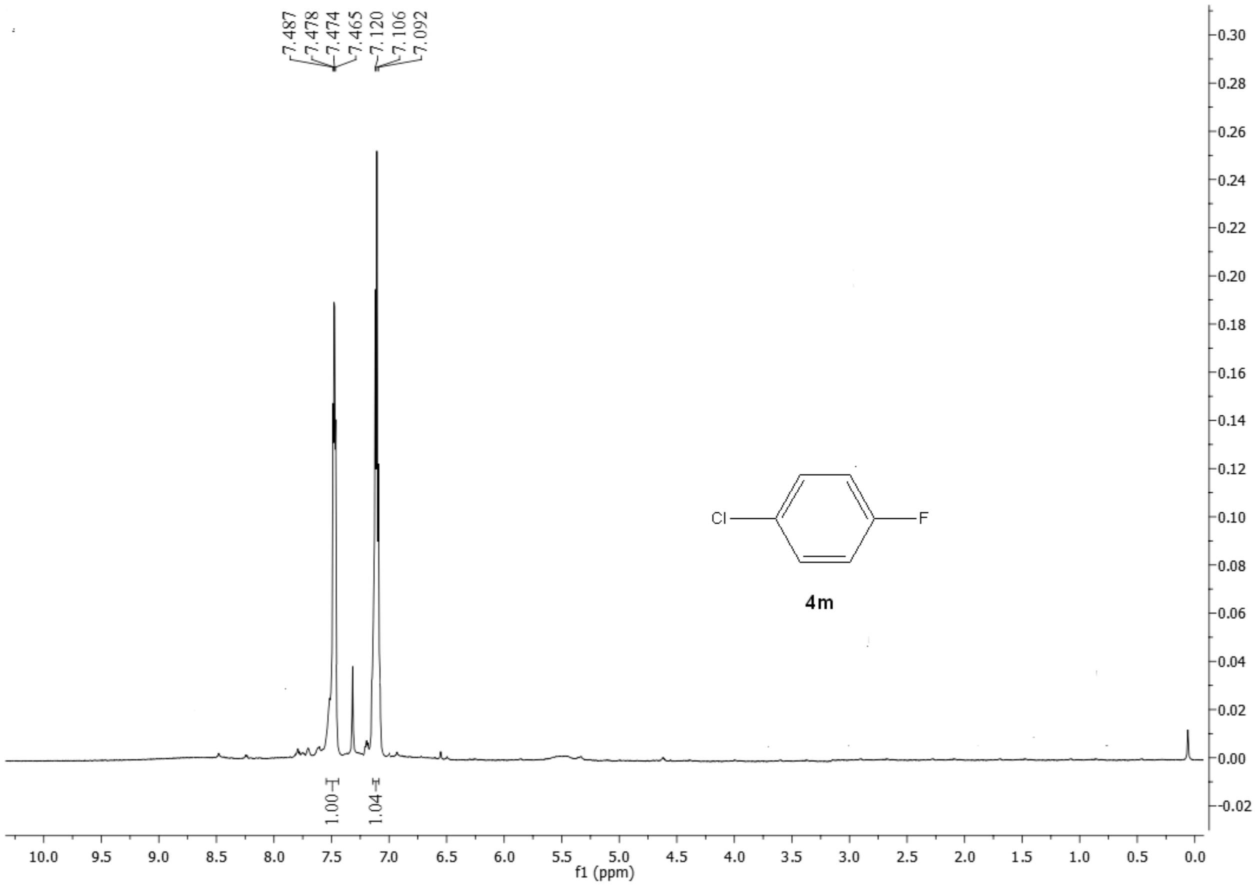
7.487
7.478
7.474
7.465
7.120
7.106
7.092
Cl
F
4m
1.00
1.04
f1 (ppm)

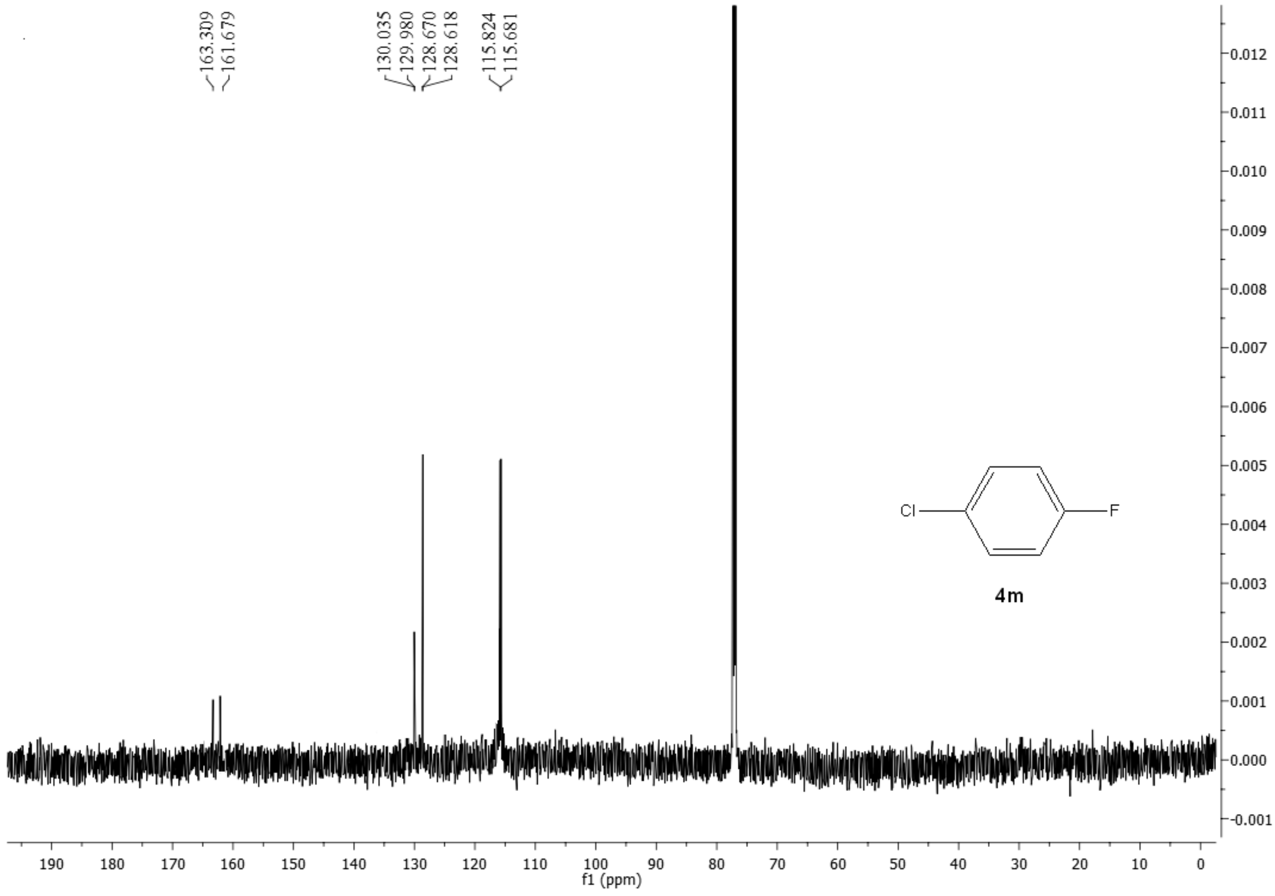
163.309
161.679
130.035
129.980
128.670
128.618
115.824
115.681
Cl
F
4m
f1 (ppm)

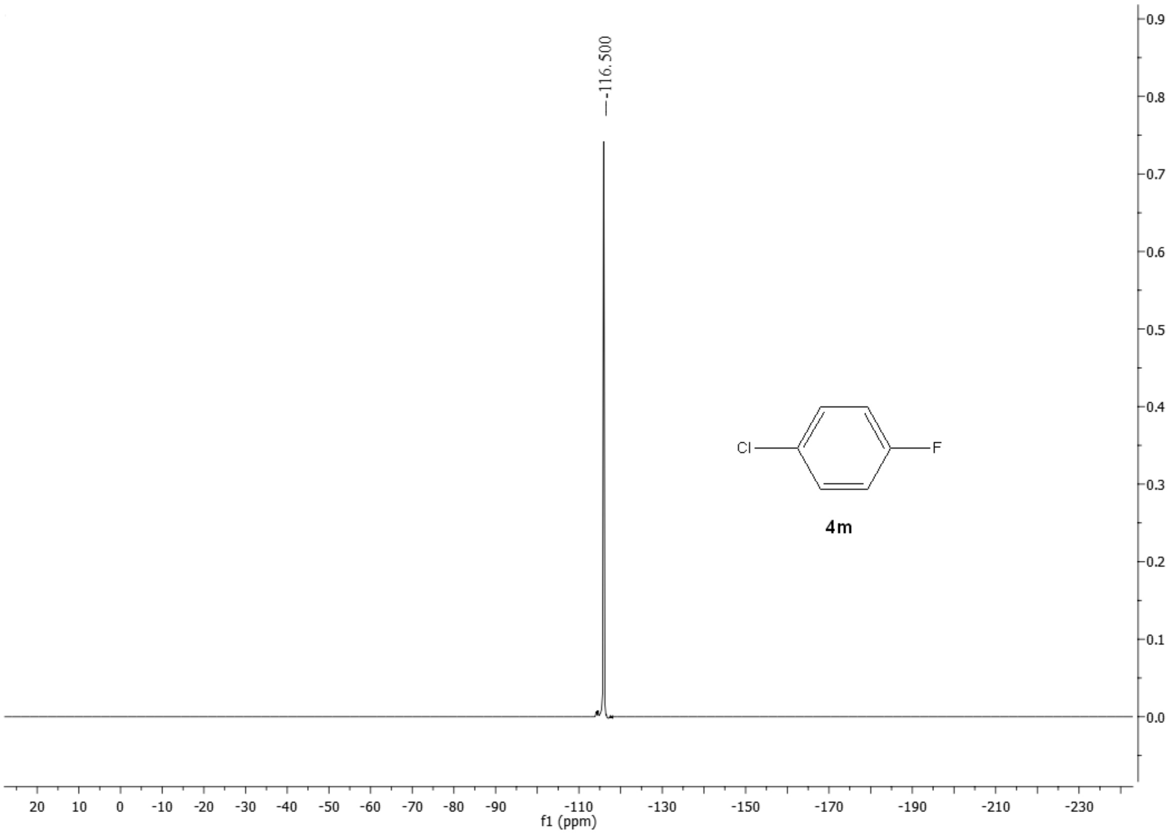

-116.500
Cl
F
4m
0.9
0.8
0.7
0.6
0.5
0.4
0.3
0.2
0.1
0.0
20 10 0 -10 -20 -30 -40 -50 -60 -70 -80 -90 -110 -130 -150 -170 -190 -210 -230
f1 (ppm)

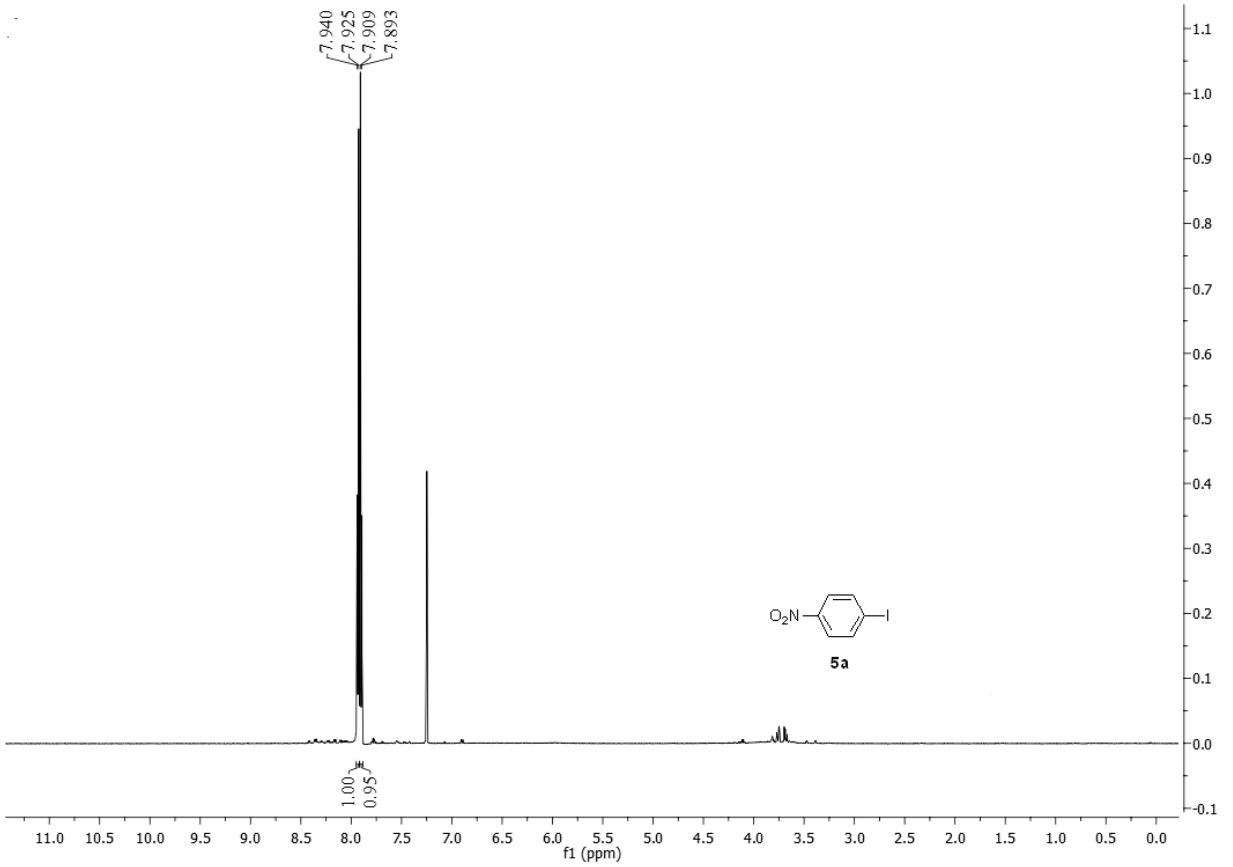
7.940
7.925
7.909
7.893
1.00
0.95
O2N
I
5a
f1 (ppm)

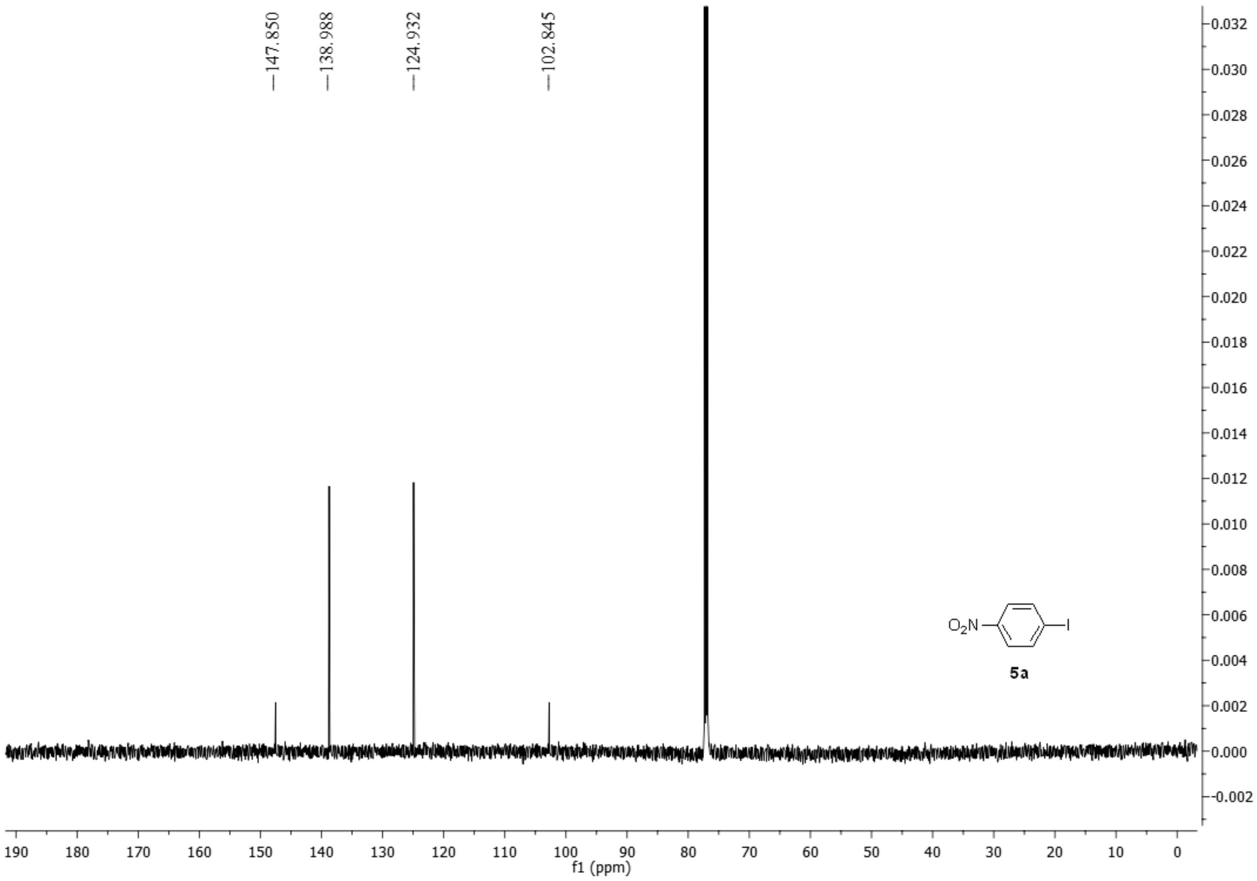
147.850
138.988
124.932
102.845
O2N
I
5a
f1 (ppm)

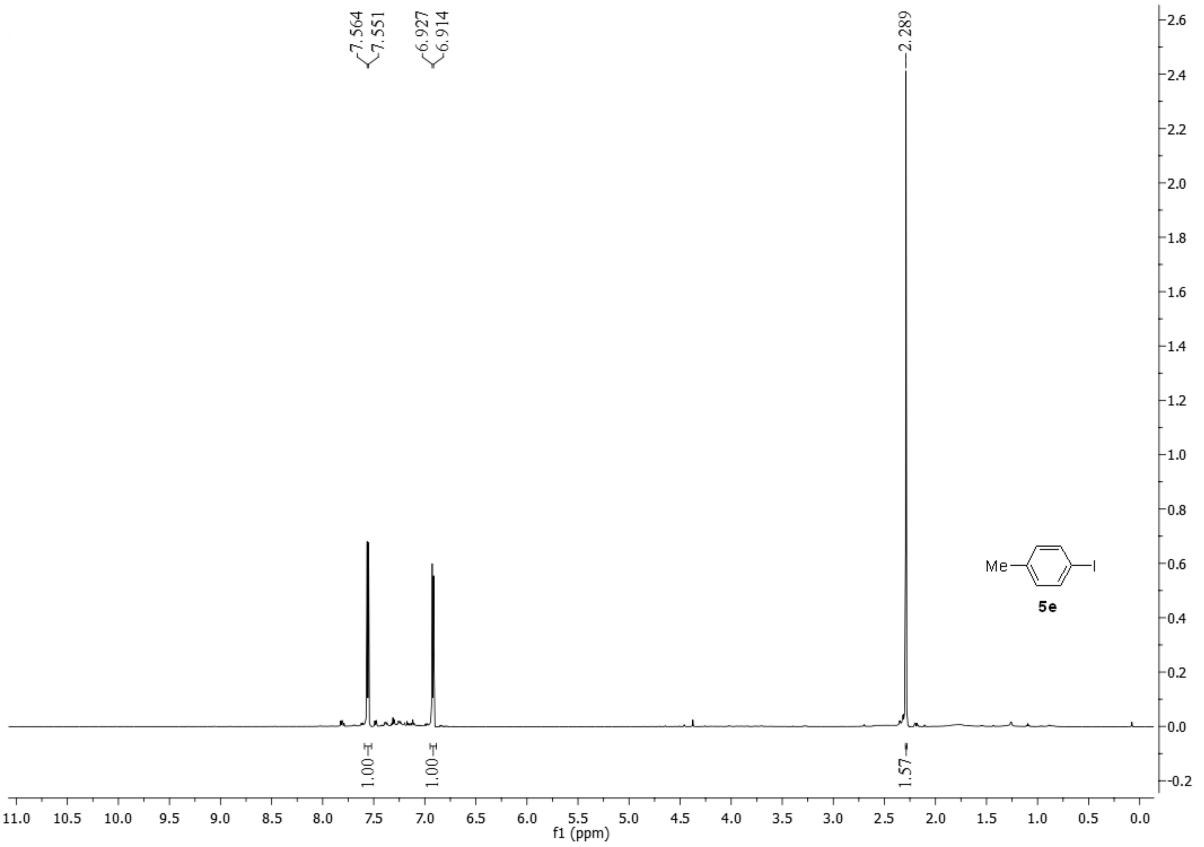
7.564
7.551
6.927
6.914
2.289
1.00
1.00
1.57
Me
I
5e
f1 (ppm)

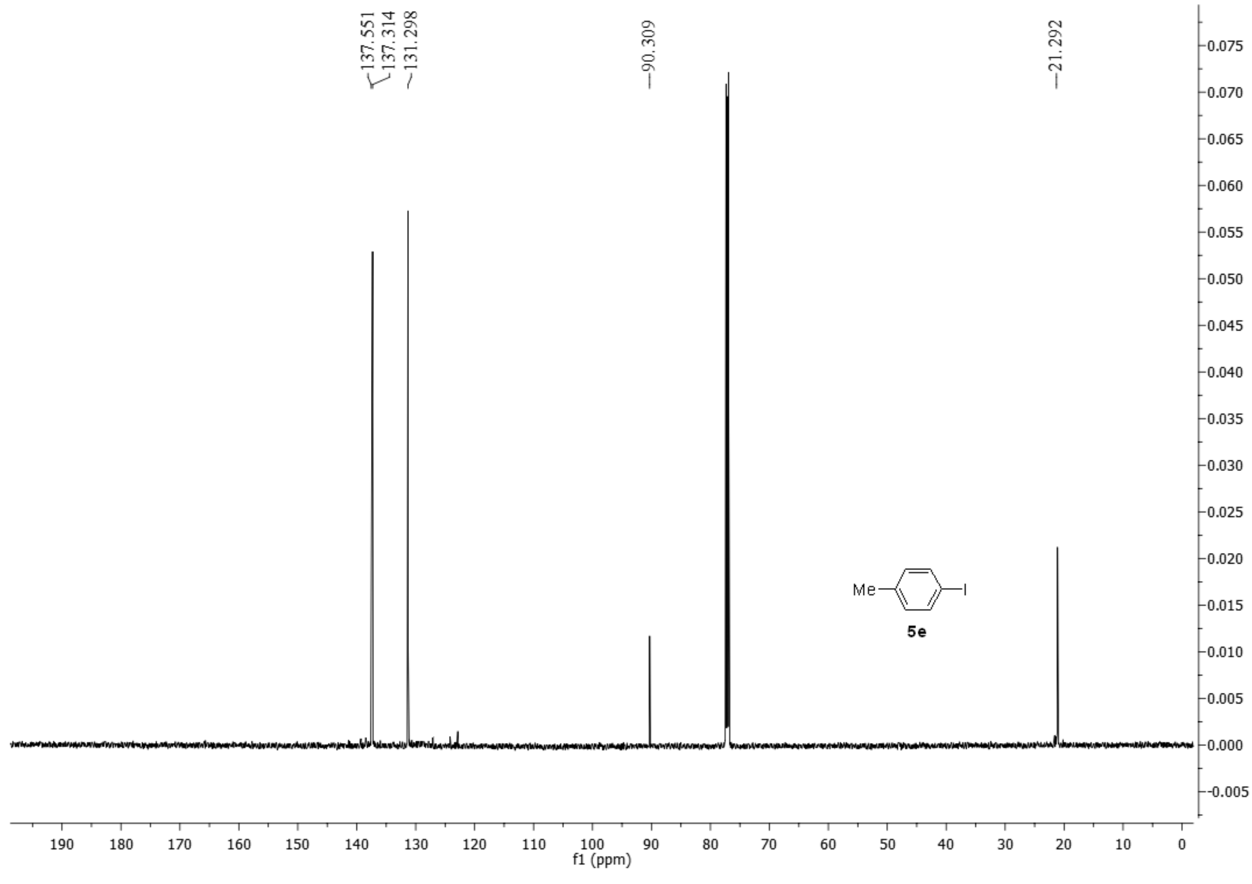
137.551
137.314
131.298
90.309
21.292
Me
I
5e
f1 (ppm)

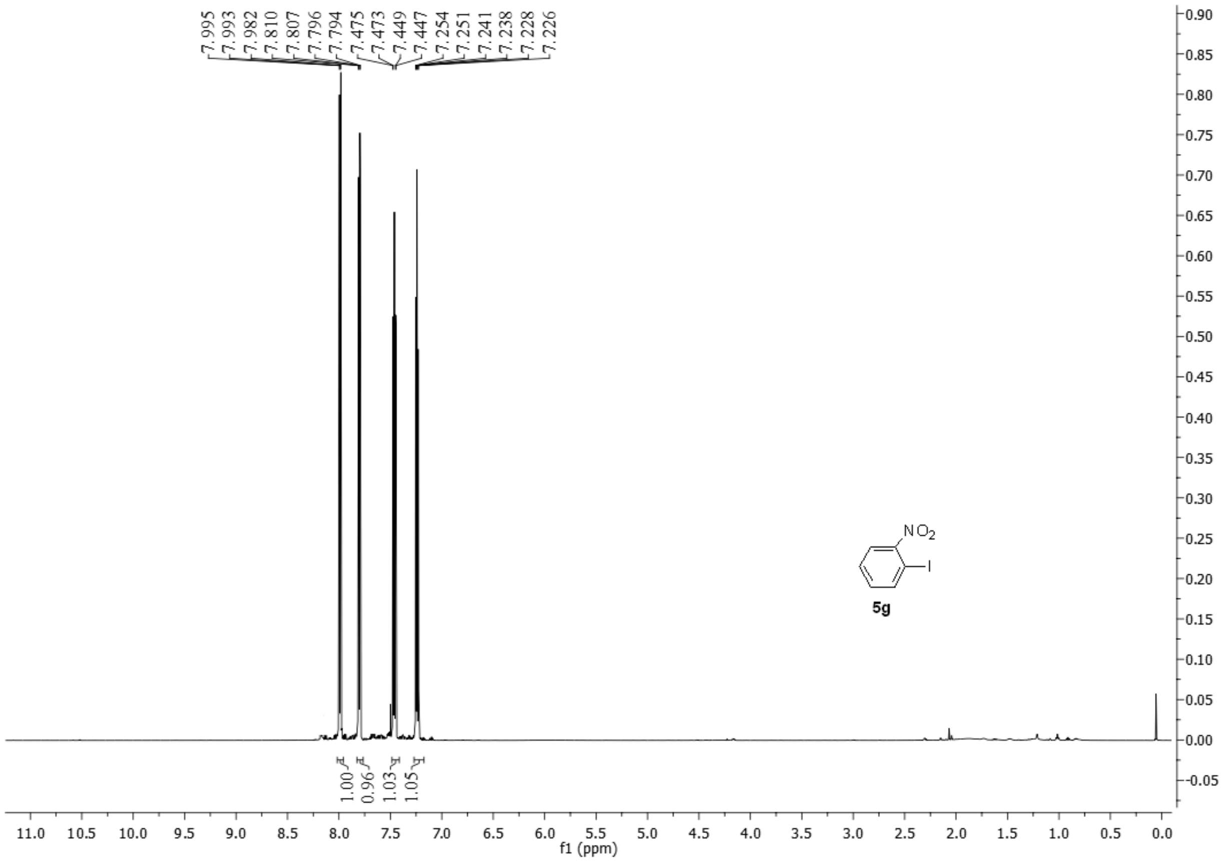
7.995
7.993
7.982
7.810
7.807
7.796
7.794
7.475
7.473
7.449
7.447
7.254
7.251
7.241
7.238
7.228
7.226
1.00
0.96
1.03
1.05
NO2
I
5g
f1 (ppm)

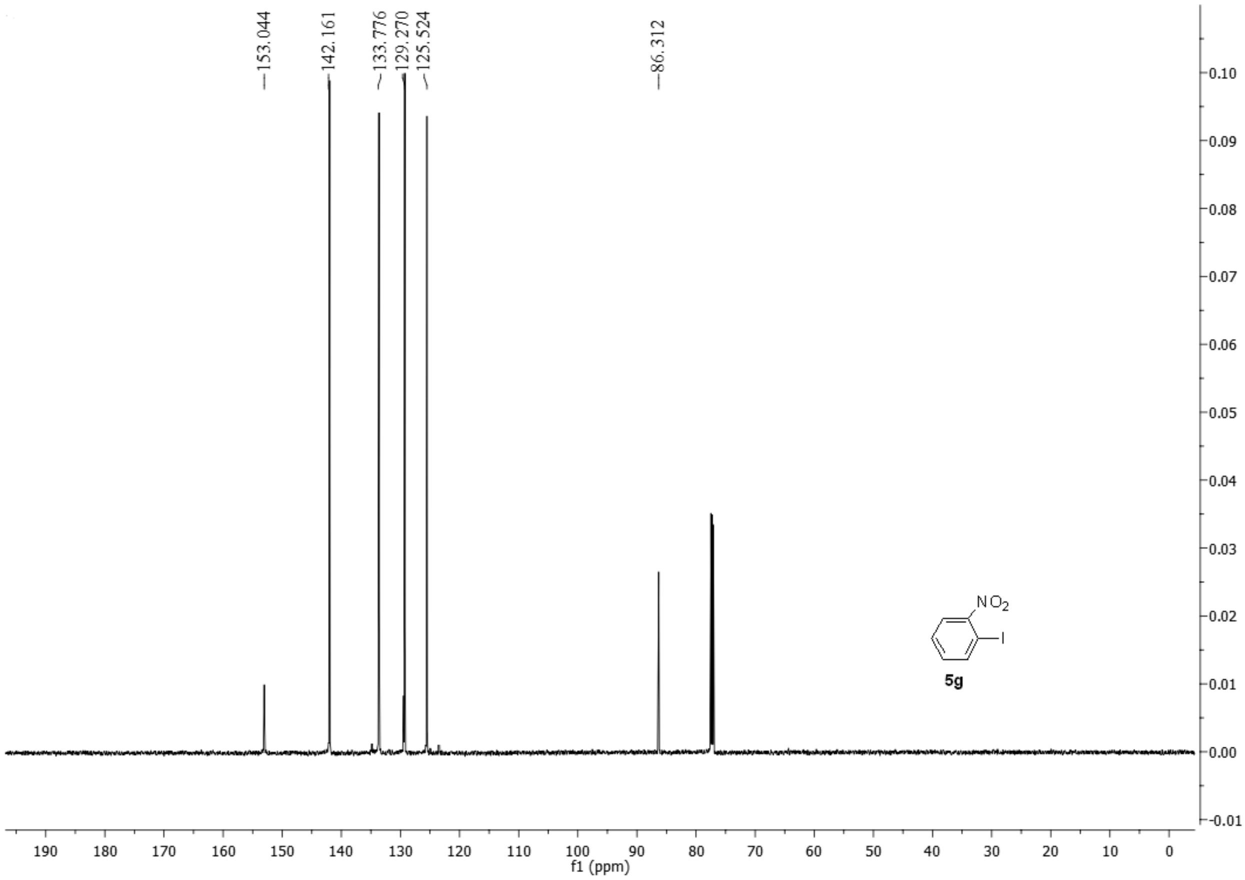
153.044
142.161
133.776
129.270
125.524
86.312
NO2
I
5g
f1 (ppm)

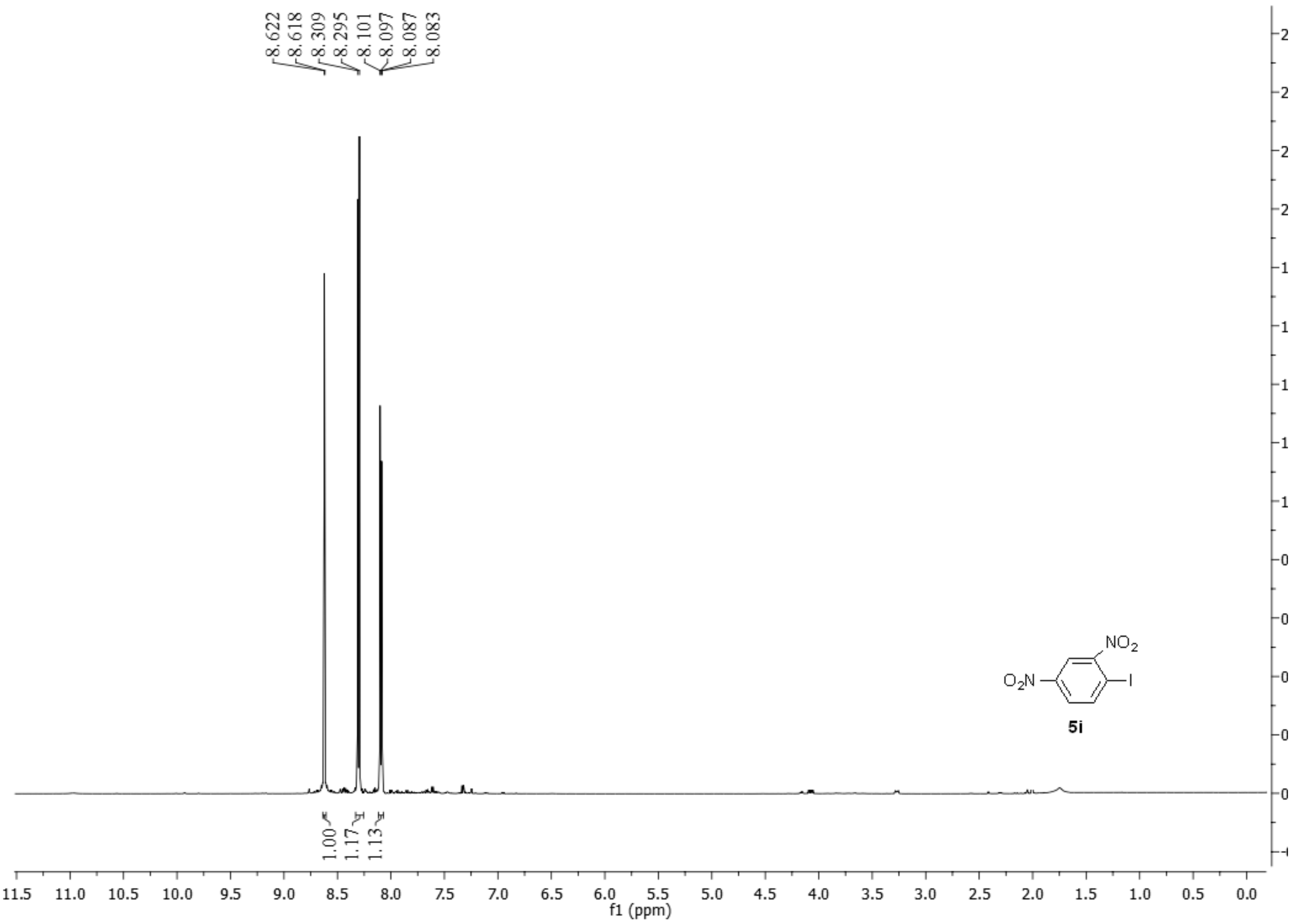
8.622
8.618
8.309
8.295
8.101
8.097
8.087
8.083
1.00
1.17
1.13
f1 (ppm)
5i

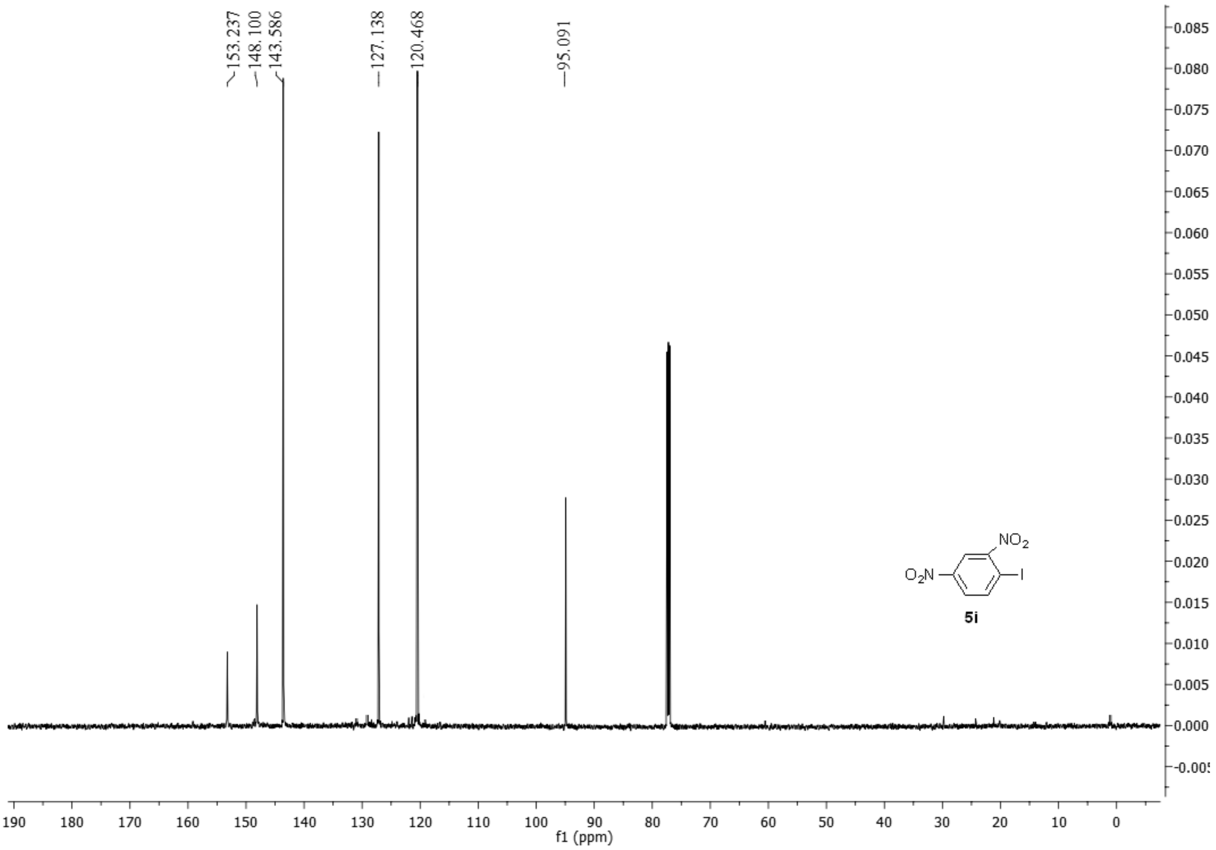
153.237
148.100
143.586
127.138
120.468
95.091
f1 (ppm)
5i

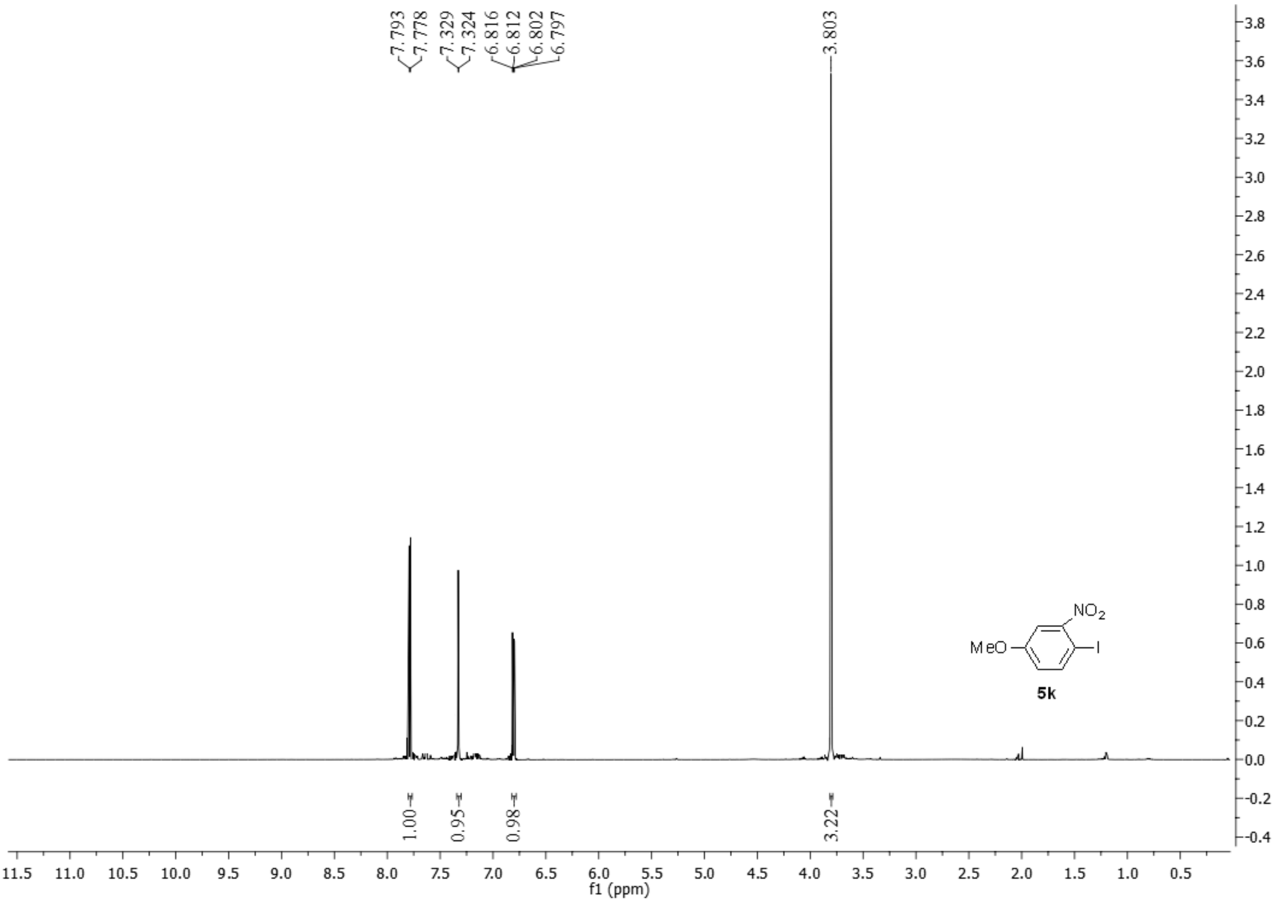
7.793
7.778
7.329
7.324
6.816
6.812
6.802
6.797
3.803
1.00
0.95
0.98
3.22
MeO
NO2
I
5k
f1 (ppm)

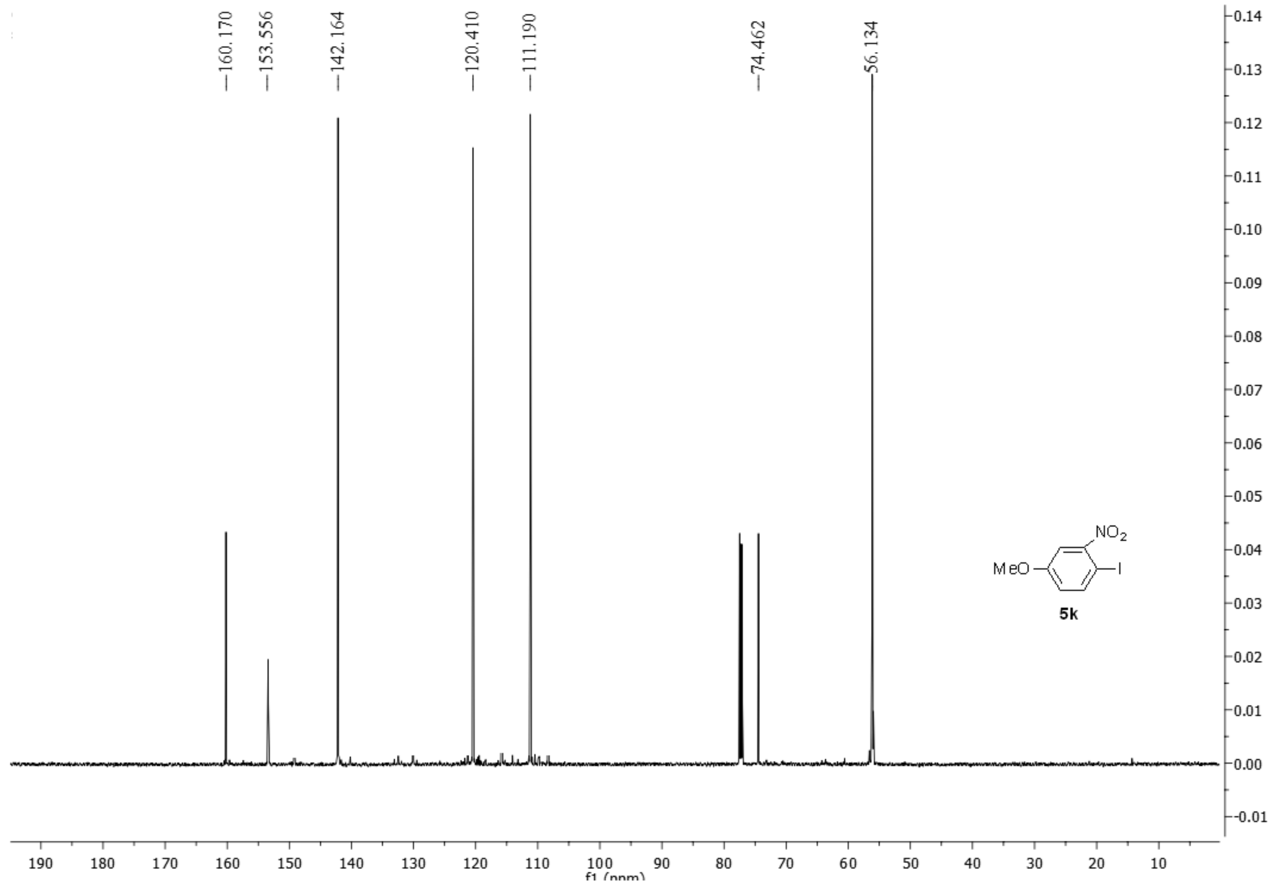
160.170
153.556
142.164
120.410
111.190
74.462
56.134
MeO
NO2
I
5k
f1 (ppm)

NMR Spectra of glycerol/KBr (6:1) solvent system

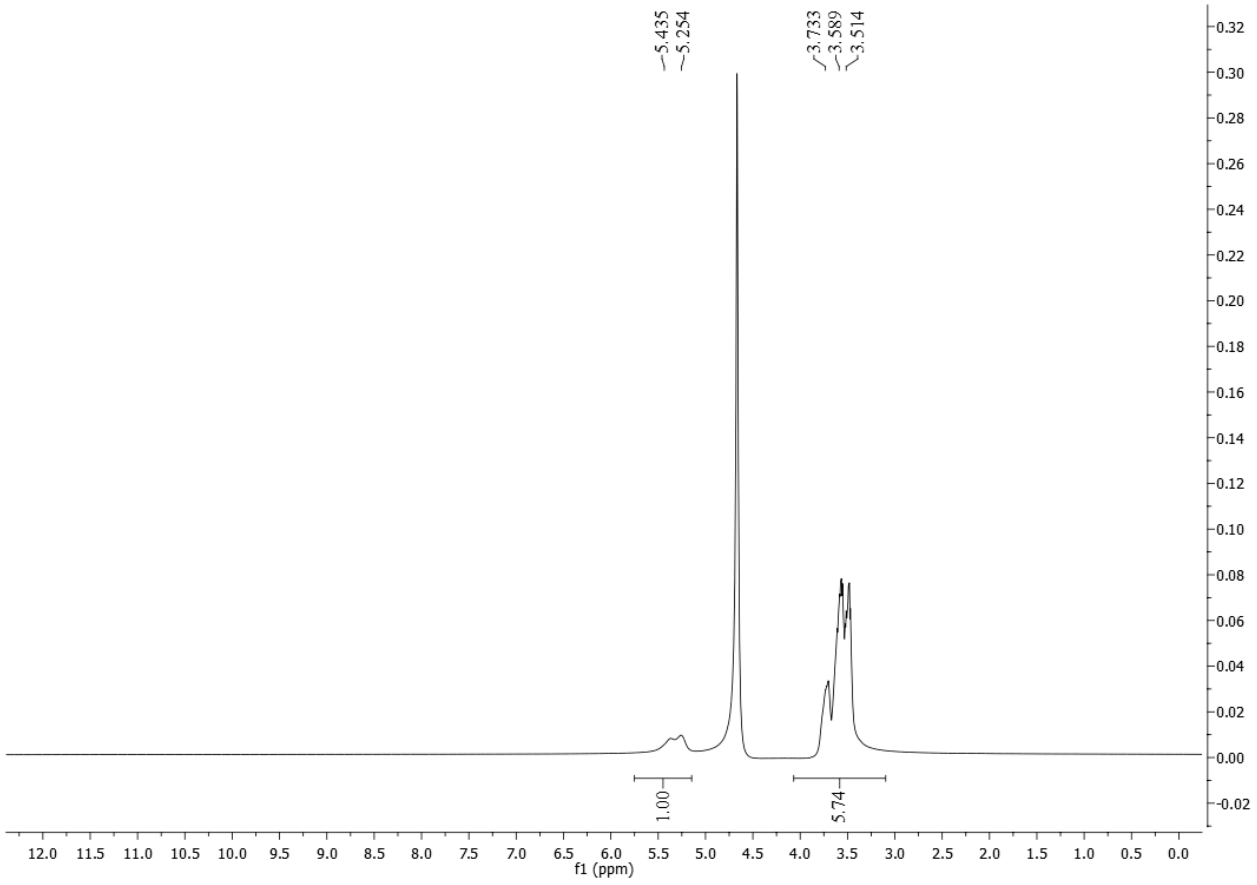

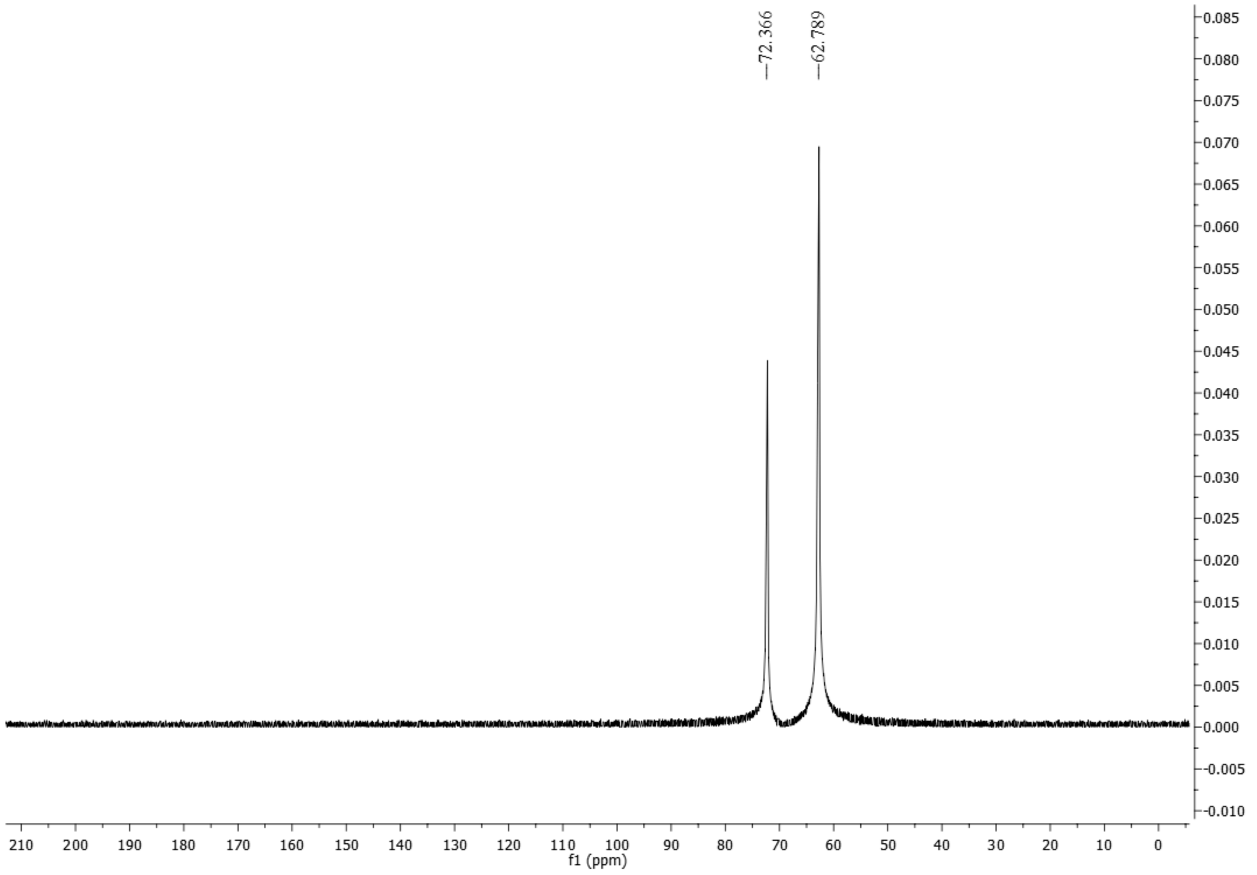

NMR spectra of recovered glycerol/KBr (6:1) solvent system after the fifth run

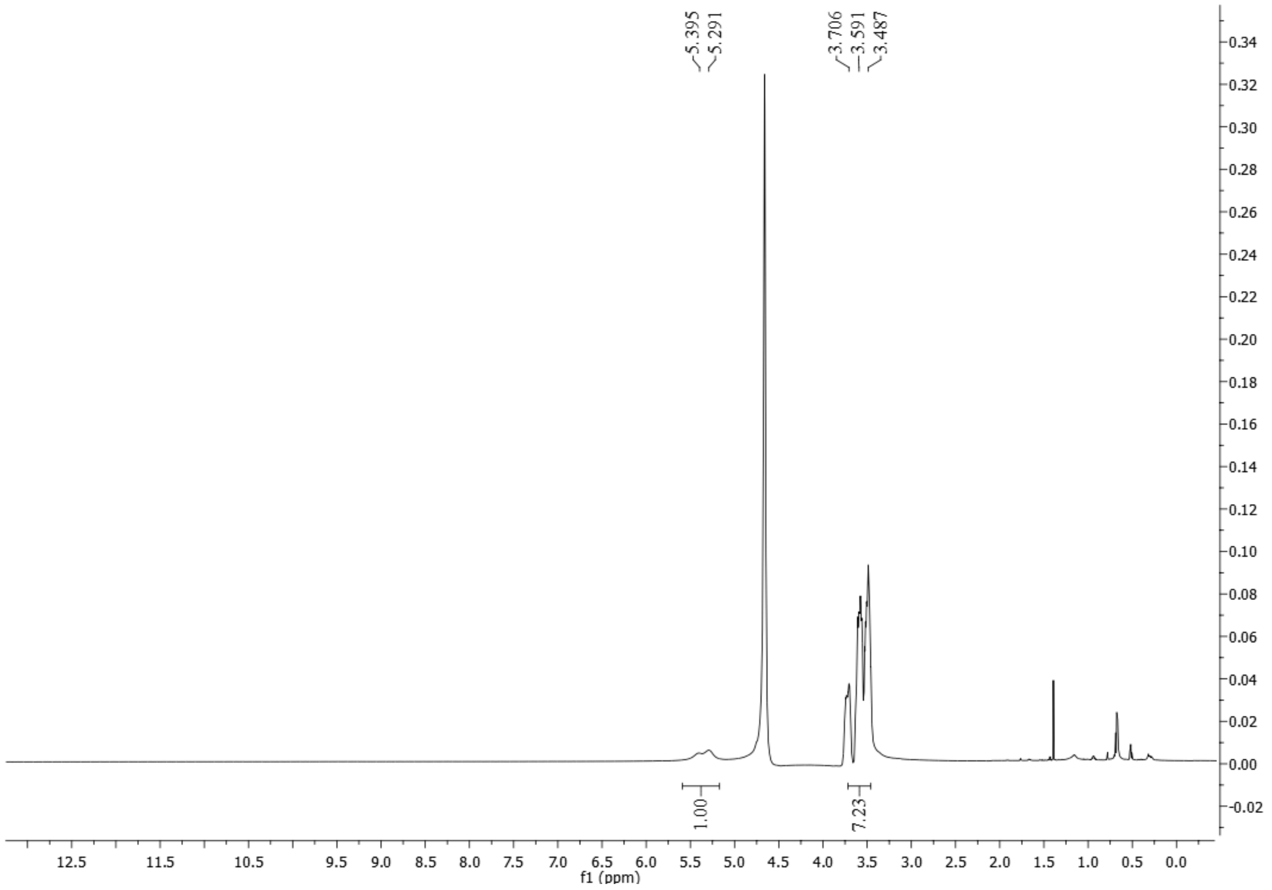

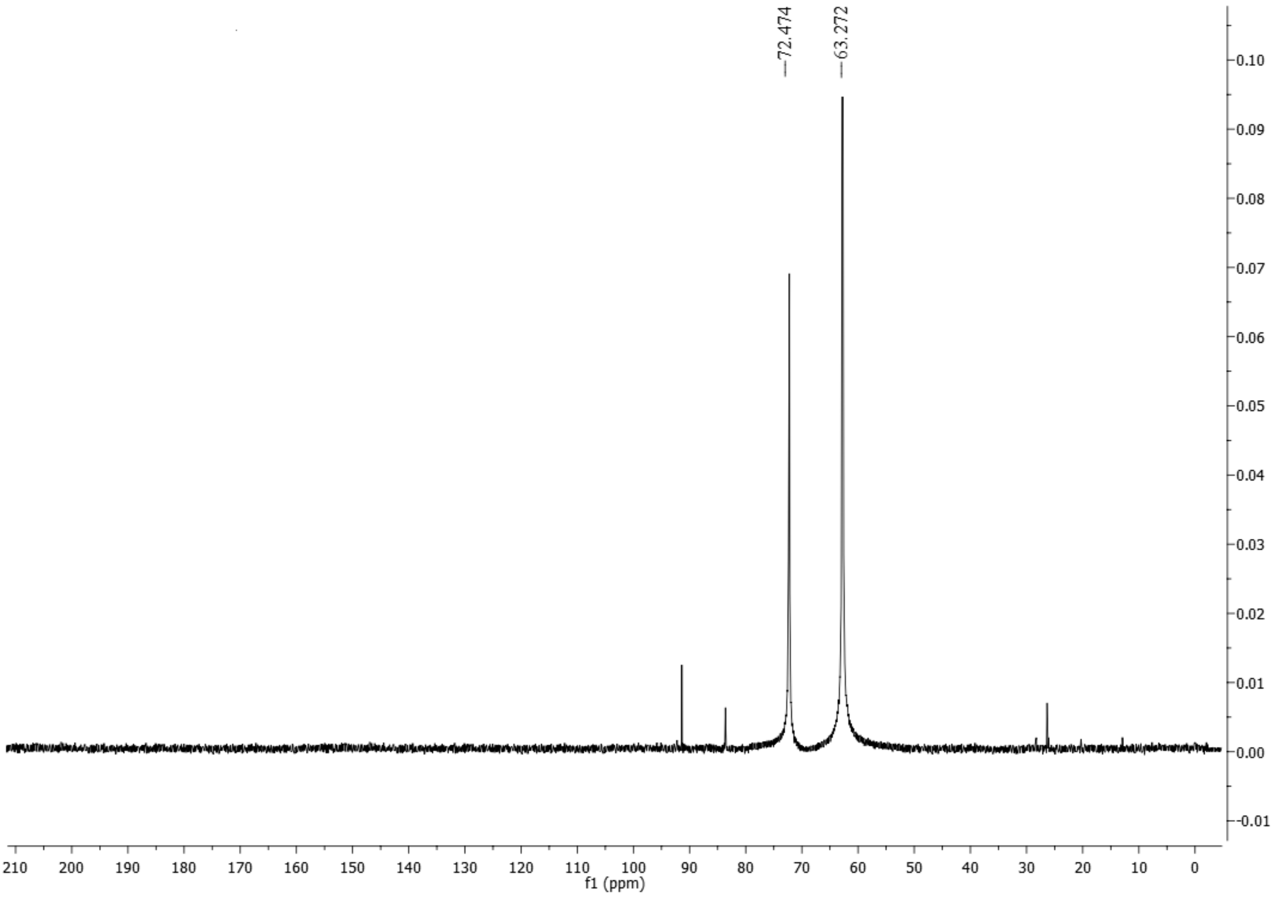

Differential Scanning Calorimetry tracks of **pure glycerol (in red)** and **Glycerol:KBr 6:1 DES-like mixture (in black).** Scan rate 5 °C/min from -80 °C to 80 °C and backwards.

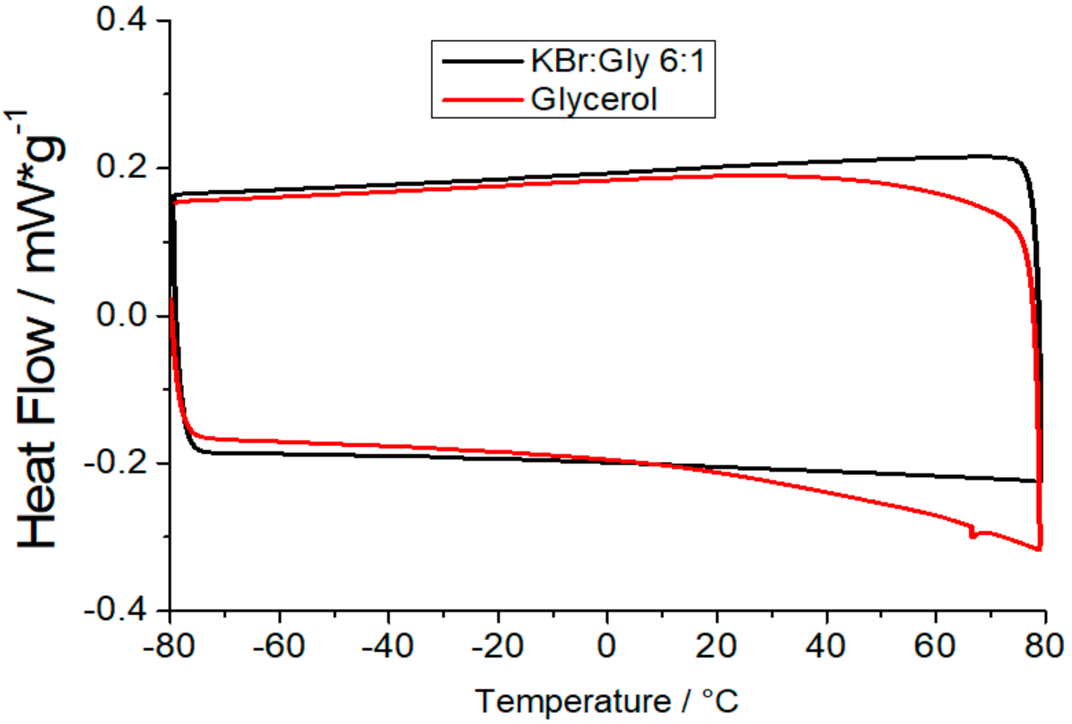

Supplement: Supplementary file 1 [file molecules-27-01909-s001.zip › molecules-1629013-supplementary.pdf]
